# The Genomic Architecture of Novel *Simulium damnosum Wolbachia* Prophage Sequence Elements and Implications for Onchocerciasis Epidemiology

**DOI:** 10.3389/fmicb.2017.00852

**Published:** 2017-05-29

**Authors:** James L. Crainey, Jacob Hurst, Poppy H. L. Lamberton, Robert A. Cheke, Claire E. Griffin, Michael D. Wilson, Cláudia P. Mendes de Araújo, María-Gloria Basáñez, Rory J. Post

**Affiliations:** ^1^Laboratório de Ecologia de Doenças Transmissíveis na Amazônia, Fundação Oswaldo Cruz, Instituto Leônidas e Maria DeaneManaus, Brazil; ^2^Oxford Martin School, Institute for Emerging Infections, University of OxfordOxford, UK; ^3^Institute of Biodiversity, Animal Health and Comparative Medicine, Wellcome Centre for Molecular Parasitology, University of GlasgowGlasgow, UK; ^4^Natural Resources Institute, University of Greenwich at MedwayChatham, UK; ^5^Department of Infectious Disease Epidemiology, Faculty of Medicine (St Mary's campus), London Centre for Neglected Tropical Disease Research, School of Public Health, Imperial College LondonLondon, UK; ^6^Core Research Laboratories Department, Molecular Biology Laboratories Division, Natural History MuseumLondon, UK; ^7^Noguchi Memorial Institute for Medical Research, University of GhanaAccra, Ghana; ^8^School of Natural Sciences and Psychology, Liverpool John Moores UniversityLiverpool, UK; ^9^Department of Disease Control, Faculty of Infectious Tropical Diseases, London School of Hygiene and Tropical MedicineLondon, UK

**Keywords:** *Wolbachia*, *Wolbachia* phages, serine recombinase, SpvB protein homolog, *Simulium squamosum* E, onchocerciasis

## Abstract

Research interest in *Wolbachia* is growing as new discoveries and technical advancements reveal the public health importance of both naturally occurring and artificial infections. Improved understanding of the *Wolbachia* bacteriophages (WOs) WOcauB2 and WOcauB3 [belonging to a sub-group of four WOs encoding serine recombinases group 1 (sr1WOs)], has enhanced the prospect of novel tools for the genetic manipulation of *Wolbachia*. The basic biology of sr1WOs, including host range and mode of genomic integration is, however, still poorly understood. Very few sr1WOs have been described, with two such elements putatively resulting from integrations at the same *Wolbachia* genome loci, about 2 kb downstream from the *FtsZ* cell-division gene. Here, we characterize the DNA sequence flanking the *FtsZ* gene of *w*Dam, a genetically distinct line of *Wolbachia* isolated from the West African onchocerciasis vector *Simulium squamosum* E. Using Roche 454 shot-gun and Sanger sequencing, we have resolved >32 kb of WO prophage sequence into three contigs representing three distinct prophage elements. Spanning ≥36 distinct WO open reading frame gene sequences, these prophage elements correspond roughly to three different WO modules: a serine recombinase and replication module (sr1RRM), a head and base-plate module and a tail module. The sr1RRM module contains replication genes and a Holliday junction recombinase and is unique to the sr1 group WOs. In the extreme terminal of the tail module there is a SpvB protein homolog—believed to have insecticidal properties and proposed to have a role in how *Wolbachia* parasitize their insect hosts. We propose that these *w*Dam prophage modules all derive from a single WO genome, which we have named here sr1WOdamA1. The best-match database sequence for all of our sr1WOdamA1-predicted gene sequences was annotated as of *Wolbachia* or *Wolbachia* phage sourced from an arthropod. Clear evidence of exchange between sr1WOdamA1 and other *Wolbachia* WO phage sequences was also detected. These findings provide insights into how *Wolbachia* could affect a medically important vector of onchocerciasis, with potential implications for future control methods, as well as supporting the hypothesis that *Wolbachia* phages do not follow the standard model of phage evolution.

## Introduction

It is estimated that *Wolbachia* naturally infect about 40% of arthropods, including many important disease vectors (Bourtzis et al., 2014; Zug and Hammerstein, 2015). As these infections have an impact on several epidemiologically-relevant aspects of disease vector biology, such as longevity, insecticide resistance, and refractoriness to infection, it has been argued that *Wolbachia* are likely to influence disease epidemiology (Echaubard et al., 2010; Slatko et al., 2014; Hoffmann et al., 2015). Much of the present public health interest in arthropod-infecting *Wolbachia* focuses on how artificial infections can be manipulated as tools for effective disease control (Bourtzis et al., 2014; Hoffmann et al., 2015; Jeffries and Walker, 2015).

*Wolbachia* bacteriophages (WOs) have received far less attention than their bacterial hosts, with some research focusing on how they could influence disease ecology and epidemiology (Tanaka et al., 2009; Metcalf and Bordenstein, 2012; LePage and Bordenstein, 2013; Wang et al., 2013) and, most commonly, how they might be utilized for disease control (Metcalf and Bordenstein, 2012; LePage and Bordenstein, 2013; Slatko et al., 2014). Several authors have advocated the possibility of developing artificial WO vectors for the genetic modification of *Wolbachia*. Despite the potential of WO-based tools and the growing interest in the use of *Wolbachia* for vector-borne disease control, there are presently no genetic manipulation tools available for the genetic engineering of *Wolbachia* (LePage and Bordenstein, 2013; Bourtzis et al., 2014; Slatko et al., 2014; Hoffmann et al., 2015; Jeffries and Walker, 2015). There is, thus, a growing need for a better understanding of the basic biology, diversity and distribution of naturally occurring WOs to assess the feasibility and potential utility of WO-based *Wolbachia* manipulation tools (Tanaka et al., 2009; LePage and Bordenstein, 2013; Wang et al., 2013). Similarly, there is also a pressing need to improve our understanding about how naturally occurring WOs influence vector-borne disease epidemiology and what risks (if any) they pose to the safety of using artificial *Wolbachia* infections for disease control (Bourtzis et al., 2014; Hoffmann et al., 2015; Jeffries and Walker, 2015; Caragata et al., 2016).

In previous studies, we identified a genetically isolated strain of *Wolbachia* from the West African onchocerciasis vector *Simulium squamosum* E (a member of the *S. damnosum* sensu lato [s.l.] species complex [Diptera: Simuliidae]) and identified bacterial artificial chromosomes (BACs) containing its *FtsZ* cell-division gene (Crainey et al., 2010a,b). As shown in Table 1, the *FtsZ* gene is part of a conserved block (spanning ~3 kb) immediately adjacent to where two closely-related prophages (WOcauB2 and a WOri phage relic) have been identified in two genetically-distinct *Wolbachia* genomes: *w*Cau and *w*Ri genomes (Tanaka et al., 2009; Kent et al., 2011a; Ellegaard et al., 2013). This six-gene block begins in both cases with superoxide dismutase and terminates with the magnesium chelatase-related protein, which occurs immediately adjacent to the prophages' serine recombinase gene. If these WOs belong to a group of site-specific bacteriophages, large cloned fragments of the *w*Dam genome, containing *FtsZ* gene sequences (Crainey et al., 2010a) could be expected also to contain *Wolbachia* prophage sequences. Similarly, if, as proposed, certain WOs have a role in male-killing (and male-killing is affecting the *S. damnosum* s.l. complex), any WOs might also be expected to harbor SpvB genes (Crainey et al., 2010a; Kent et al., 2011a; Metcalf and Bordenstein, 2012; LePage and Bordenstein, 2013). In this study, we have characterized the genomic DNA of *w*Dam flanking its *FtsZ*-gene and have recovered three WO phage sequence elements, including one that encodes a SpvB-like gene, that we propose all derive from a single WO prophage genome that we have named sr1WOdamA1.

**Table 1:** *****w***Dam shot-gun sequence contigs from matching known and predicted serine recombinase WO upstream integration sites**.

**Gene name**	**Distance (in nucleotides, nts) from WO phage terminal end**	**Gene length**	***w*Dam sequence match**	***w*Dam contig(s)**	***w*Dam contig length**
Mg chelatase-related protein *w*Cau protein ID: BAH22205 *w*Ri protein ID: ACN95503	158 nts from 5′ terminal end of WOcauB2 0 nts from 5′ terminal end of WOriRelic1	*w*Cau: 1,238 *w*Ri: 1,464	*w*Cau: co-ordinates: 261–891 Identity: 527/632 (83%) BLAST score: 587 bits (738) *w*Ri: co-ordinates: 259–888 Identity: 523/631(83%) BLAST score: 648 bits (718)	KY695242	632
Cell-division protein *FtsZ w*Cau protein ID: BAH22203 *w*Ri protein ID: ACN95501	2,263 nts from 5′ terminal end of WOcauB2 1,477 nts from 5′ terminal end of WOriRelic1	*w*Cau: 1,145 *w*Ri: 1,197	*w*Cau: co-ordinates: 18–1,145 Identity: 959/1,166 (82%) BLAST score: 1,166 bits (1,292) *w*Ri: co-ordinates: 1–1,193 Identity: 1,004/1,222 (82%) BLAST score: 1,048 bits (1,318)	KY695243	1,943
Hypothetical protein GF1gp18 *w*Cau protein ID: BAH22202 *w*Ri protein ID: ACN95500	3,390 nts from 5′ terminal end of WOcauB2 3,371 nts from 5′ terminal end of WOriRelic1	*w*Cau: 399 *w*Ri: 399	*w*Cau: co-ordinates: 1–161 Identity: 132/161 (82%) BLAST score:159 bits (176)	KY695244	729
			*w*Cau: co-ordinates: 202–399 Identity: 162/202 (80%) BLAST score: 157 bits (196)	KY695243	1,943
			*w*Ri: coordinates: 11–161 Identity: 124/151 (82%) BLAST score: 133 bits (166)	KY695244	729
			*w*Ri: coordinates: 200–399	KY695243	1,943
			Identity: 164/200 (82%) BLAST score: 199 bits (220)		
Hypothetical protein GF1gp17 *w*Cau protein ID: BAH22201 *w*Ri protein ID: ACN95499	3,782 nts from 5′ terminal end of WOcauB2 4,151 nts from 5′ terminal end of WOriRelic1	*w*Cau: 459 *w*Ri: 453	*w*Cau: co-ordinates: 40–459 Identity: 337/423 (80%) BLAST score: 327 bits (410) *w*Ri: co-ordinates: 14–453 Identity: 365/442 (83%) BLAST score: 394 bits (494)	KY695244	729
Peptidase, M16 family *w*Cau protein ID: BAH22189 *w*Ri protein ID: ACN95488	16,926 nts from 5′ terminal end of WOcauB2 17,238 nts from 5′ terminal end of WOriRelic1	*w*Cau: 1,275 *w*Ri: 1,275	*w*Cau: co-ordinates: 1–1,262 Identity: 1,022/1,262 (81%) BLAST score: 1,195 bits (1,324) *w*Ri: coordinates: 16–1,268 Identity: 1,004/1,255 (80%) BLAST score: 992 bits (1,248)	KY695245	1,970
Superoxide dismutase *w*Cau protein ID: BAH22188 *w*Ri protein ID: ACN95487	15,656 nts from 5′ terminal end of WOcauB2 18,514 nts from 5′ terminal end of WOriRelic1	*w*Cau: 609 *w*Ri: 618	*w*Cau: co-ordinates: 1–596 Identity: 486/614 (79%) BLAST score: 529 bits (586) *w*Ri: co-ordinates: 1–606 Identity: 480/616 (78%) BLAST score: 425 bits (534)	KY695245	1,970

*Quoted sequence matches are based on BLASTn sequence comparisons implementing the “align two or more sequences” function. The BLAST scores were calculated after the default search comparison parameters were modified to a word size of 7 and gap opening penalty system of 0 for existence and 4 for extension. Only significant matches with BLAST scores over 130 bits are shown*.

## Materials and methods

### Shotgun sequencing of the genomic DNA regions flanking the *w*Dam cell-division protein *FtsZ*

Large *Wolbachia*-DNA-containing BAC clone mini cultures from seven *FtsZ*-positive BACs (identified previously) were grown shaking over-night in BAC library growth media (Crainey et al., 2010b). Thick mini-culture preparations from each BAC colony were pooled and their BAC DNA was isolated in single preparations using a QIAGEN large-construct kit and protocol (https://www.qiagen.com/kr/resources/resourcedetail?id=8f67b644-6d21-4ef3-b33e-a60f32623785&lang=en). A 10-mg sample of purified BAC DNA was shot-gun sequenced commercially using a Roche 454 FLX system sequencer at the Cambridge University Biochemistry Department. Sequence reads were quality-checked using Phred Software (http://www.phrap.org/phredphrapconsed.html) and assembled into 8,238 contigs using Phrap (Ewing and Green, 1998; Ewing et al., 1998). Shot-gun sequence contigs were screened for the presence of WOcauB2 and WOcauB2-flanking sequences using BLAST (Basic Local Alignment Search Tool) homology searches (http://blast.ncbi.nlm.nih.gov/Blast.cgi) and the NCBI sequence deposits AB478515 and AB478516 as well as a library of previously proposed *Wolbachia* phage sequences (Supplementary File 1).

### *w*Dam *Wolbachia* prophage sequence assembly

Contigs showing significant matches were classified as being of bacteriophage origin if two of their three best matches in the NCBI's non-redundant sequence data bank were annotated as a *Wolbachia* phage sequence. Contigs identified as containing possible phage sequences were aligned to the WOcauB2 reference genome to identify putative gap sequences. Primers were designed to amplify predicted phage genome “gap” DNA sequences. All “gap-closing” PCRs that produced PCR products of the expected size had their PCR fragments Sanger sequenced in the forward and reverse directions (http://www.lifesciences.sourcebioscience.com/genomic-services/sanger-sequencing-service/). A full list of the primers used for this step is provided in Supplementary File 2. The primer design and PCR conditions used to amplify these “gap regions” followed an approach described previously (Post et al., 2009). “Gap-closing” Sanger-sequence reads were aligned to those generated from 454 sequence runs and used to extend the original contigs into a total of three large non-contiguous sequences, spanning what is proposed here to be a complete WO genome sequence (i.e., from its first gene sequence to its last).

### Confirmation of WO prophage sequence proximity to the *w*Dam *FtsZ* gene sequence

A BLAST search using the *S. squamosum* E *FtsZ* sequence (FN563974) confirmed that the Roche 454 sequence reads were from the targeted *S. squamosum* E *Wolbachia* described in Crainey et al. (2010a). To confirm that the WO prophage sequences occur adjacent to the *w*Dam *FtsZ* gene, all seven *FtsZ*-positive BAC colonies used in the shotgun sequence run were individually PCR-screened for the presence of WO genes using four primer sets (Supplementary File 2). Two of the primer sets targeted the serine recombinase gene (i.e., the phage's WOcauB2 gp1 paralog), which was predicted to occur at the 5′ end of the bacteriophage (as in WOcauB2 and WOcauB3) and the other two primer sets targeted the gene sequences from the tail end of the phage (corresponding to WOcauB2 gp32 and gp33 paralogous sequences).

### Phylogenetic classification of the *w*Dam *Wolbachia* prophage

The phylogenetic classification of the WO prophage sequences was performed using the minor capsid (sometimes referred to as the WO orf7 gene) and recombinase genes corresponding to WOcauB2 gp17 and gp1 paralogs, respectively. Clustal X (Thompson et al., 1997) was used to align the serine recombinase amino acid sequence of our WO gp1 paralog to the serine recombinases amino acid sequences of WO phage used in the recombinase analysis of Kent et al. (2011a). Clustal X was also used to align the nucleotide sequence of the minor capsid gene of our WO gp17 paralog to the minor capsid genes of the same WO phage, as well as the genes of three other *Wolbachia* prophages that lack integrase/recombinase genes.

The resulting alignments were used to construct maximum likelihood trees using the software from the PHYLIP package (Felsenstein, 2002). The robustness of the constructed trees' topologies was tested with 1000 pseudoreplicates (http://evolution.genetics.washington.edu/phylip.html). Final alignment files used in the tree construction are provided in Supplementary Files 3, 4.

## Results

### Identification and structural resolution of three *w*Dam prophage sequence elements and the proposed genomic architecture of the sr1WOdamA1 prophage genome

In total, 22 contigs were identified as containing putative bacteriophage sequences and showing homology with 33 WOcauB2 genes. In each case, only one allele for each phage gene was identified, which led to the hypothesis that the sequences recovered from the shot-gun sequence analysis had originated from just one phage genome sequence that might be resolvable by gap-closing PCR amplification and Sanger sequencing. Following gap-closing PCRs, the 22 original phage contigs were extended and assembled into three large contigs totalling 32.439 kb of unique sequence, which we propose here represents the near-complete genome of sr1WOdamA1 and which has been deposited at the NCBI with accession numbers KY695239–KY695241.

### The characterization of a *w*Dam WO serine recombinase replication and repair module (sr1RRM) prophage sequence element (sr1WOdamA1 contig number 1)

**Figure 1:**
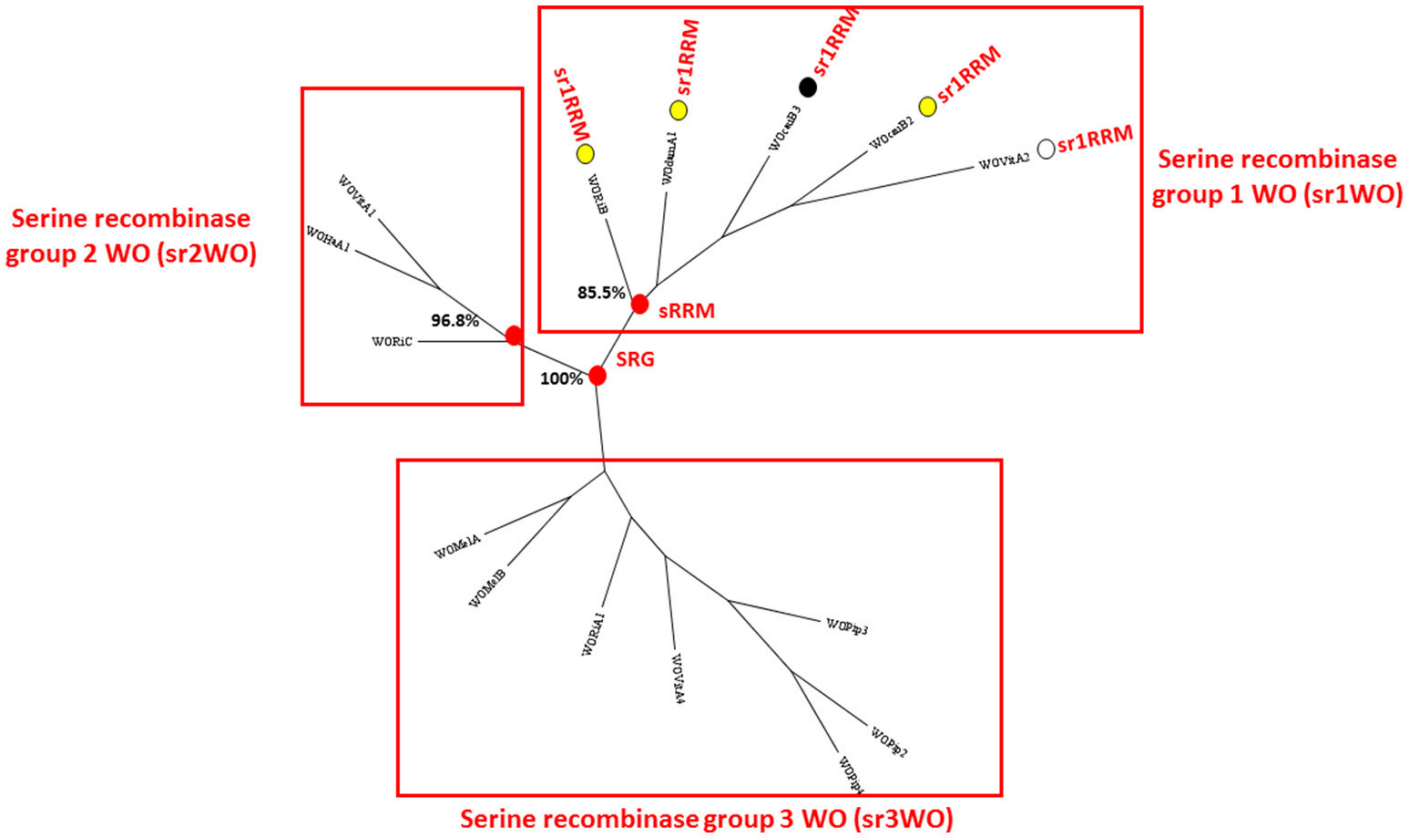
**Maximum likelihood consensus tree constructed from an alignment of ***Wolbachia*** phage recombinase amino acid sequences**. Three bootstrap-supported sequence clusters (labeled sr1WO-sr3WO) recovered in the analysis of Kent et al. (2011a) and Wang et al. (2013) were recovered in this analysis and are indicated. All the WOs known to have the structural group 1 serine recombinase replication module (sr1RRM) can be seen to occur in the sr1WO group. Sr1WO group recombinases are marked with a circle. When these circles are colored in yellow, the phage is known to occur adjacent to the *FtsZ* cell-division gene, white coloring indicates that the phage's genomic location is unknown and black is used to indicate that the phage does not appear to be located close to the *FtsZ* gene. The recombinase amino acid sequences are provided in Supplementary File 3.

**Figure 2:**
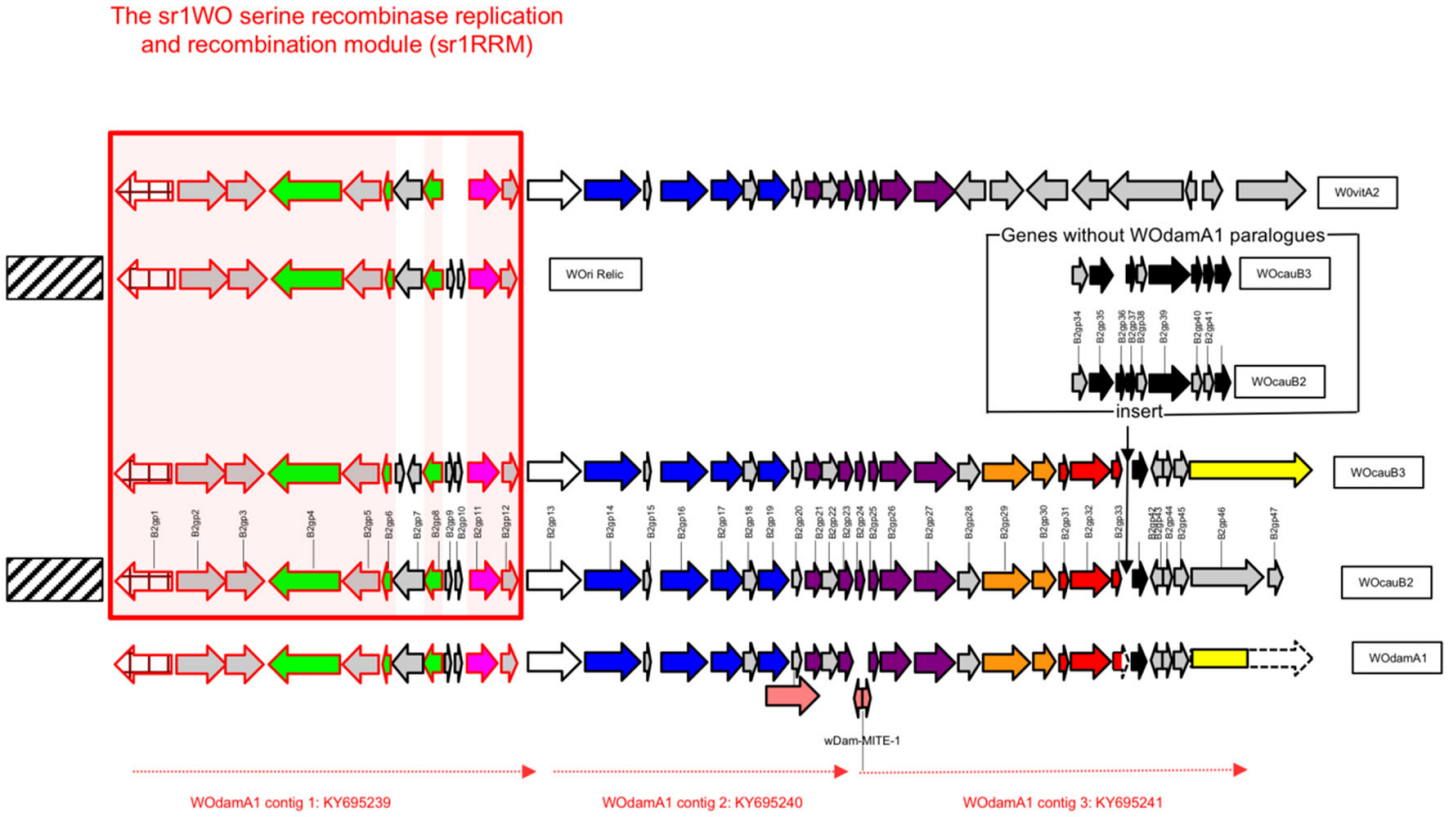
**Schematic representation of the predicted structural organization of sr1WOdamA1 in relation to all four other members of the sr1WO group bacteriophage group (sr1WO)**. Arrows are used to indicate the direction in which predicted gene sequences are encoded and shading is used to indicate functional homology. The gene sequences of the WOcauB2 reference genome (used in this paper and in Kent et al., 2011a) are annotated with numbers and the gene product (gp) abbreviation. A conserved block of genes spanning from WOcauB2 gp1 to WOcauB2 gp12 with functions involved in recombination and replication functions, and referred to in the main text as “sr1RMM,” is highlighted with a red box. See also Figure 1. Nine perfectly conserved genes found within this box are indicated with pink highlighting. Predicted gene protein functional groups have been colored following the classification of Kent et al. (2011a). Blue is used to show “head” proteins; purple is used for “base-plate” proteins; orange is used for virulence proteins, and black is used for “tail” proteins. Red coloring is used to highlight a block of three predicted gene proteins, which appears only to occur in sr1WOdamA1, WOcauB2, and WOcauB3, suggesting a special relationship between these phages. The inset with WOcauB2 and WOcauB3 genes that have no WOdamA1 paralogs represents that there is no non-coding DNA separating the 3′-truncated gp33 paralog and the gp42 paralog in sr1WOdamA1 contig 3. The two proposed transposable elements referred to in the main text are indicated with pink arrows; the *w*Dam-MITE-1 element is also labeled with its name.

*w*Dam WO prophage contig number 1 (NCBI accession number KY695239) is 11.689 kb in length and is predicted to contain a block of 12 genes that show high levels of sequence identity with the first 12 predicated genes in WOcauB2 [WOcauB2 gp1–gp12 (Table 2)]. The WOcauB2 gp1 paralog (sr1WOdamA1 gp1p) occurs at the extreme 5' terminal end of this contig and corresponds to the sr1WOdamA1 recombinase gene, whose phylogenetic analysis robustly groups with the four previously described WO group serine recombinases (Figure 1). The next 11 gene sequences occur in the same order and orientation as in WOcauB2, representing the conserved group 1 serine recombinase and replication module (sr1RRM) which is unique to and highly conserved among, the sr1WO group bacteriophages (Figure 2 and below). The extreme 3′ terminal end of contig 1 shows very high levels of sequence identity with the WOcauB2 predicated gene protein 13 (WOcauB2 gp13). The first 545 base pairs of WOcauB2 gp13 thus correspond with the last 469 nucleotides of contig 1 (Table 2). As the first 354 nucleotides of contig 2 correspond to the last 369 nucleotides of the same gene (WOcauB2 gp13), we assumed that the two contigs would be easily joined by PCR (Table 2). Despite repeated efforts (using eight different primer sets), we were unable to bridge what we predicted would correspond to a 584 base-pair (bp) gap of WOcauB2 gp13 paralog gene sequence, which we expected to occur between contigs 1 and 2 (Table 2). It is, thus, most probable that the sr1WOdamA1 genome is not orientated as the WOcauB2 genome (Figure 2 shows its similarity to the other serine recombinase WOs).

**Table 2:** **Conservation of gene content, synteny and sequence similarity in the sr1WO group ***Wolbachia*** phage, and gene-content inventory for the three sr1WOdamA1 ***Wolbachia*** phage contigs generated and characterized in this study**.

**Gene name^†^**	**sr1WOdamA1 contig #1 KY695239^§^**	**sr1WOdamA1 contig #2 KY695240^§^**	**sr1WOdamA1 contig #3 KY695241^§^**	**WOcauB2 AB478515^‡^ Coordinates Similarity (percent) Divergence (percent) BLAST score**	**WOcauB3 AB478516^‡^ Coordinates Similarity (percent) Divergence (percent) BLAST score**	**WOvitA HQ906663^‡^ Coordinates Similarity (percent) Divergence (percent) BLAST score**	**WOsimwRi CP001391^‡^ Coordinates Similarity (percent) Divergence (percent) BLAST score**
WOB2pgp1	34–1,565	–	–	20,032–21,575	16,816–18,347	89–1,632	81,7,261–81,8794
Recombinase				1,346/1,550 (87%)	1,318/1,538 (86%)	1,350/1,550 (87%)^*^	1,31,4/1,540 (85%)
				24/1,550 (1%)	24/1,538 (1%)	24/1,550 (1%)	24/1,540 (1%)
				1,633 bits (2,056)	1,551 bits (1,952)	1,649 bits (2,076)^*^	1,530 bits (1,926)
WOB2pgp2	1,906–3,104	–	–	21,893–23,090	18,700–19,897	1,950–3,1,47	819,117–820,31,3
				1,112/1,201 (93%)	1,108/1,201 (92%)	1,118/1,201 (93%)	1,132/1,200 (94%)^*^
				5/1,201 (0.4%)	5/1,201 (0.4%)	5/1,201 (0.4%)	5/1,200 (0.4%)
				1,551 bits (1,952)	1,535 bits (1,932)	1,574 bits (1,982)	1,632 bits (2,054)^*^
WOB2pgp3	3,070–4,179	–	–	23,056–24,1,65	19,863–20,972	3,1,13–4,222	820,279–821,388
				1,055/1,110 (95%)	1,058/1,110 (95%)	1,087/1,110 (98%)^*^	1,034/1,110 (93%)
				0/1,110 (0%)	0/1,110 (0%)	0/1,110 (0%)	0/1,110 (0%)
				1,544 bits (1,944)	1,557 bits (1,960)	1,671 bits (2,104)^*^	1,462bits (1,840)
WOB2pgp4	4,185–6,205	–	–	24,191–26,187	20,998–22,994	4,228–6,238	821,402–823,408
				1,882/1,998 (94%)	1,899/1,998 (95%)	1,903/2,012 (95%)	1,867/2,011 (93%)
				4/1,998 (0.2%)	4/1,998 (0.2%)	4/2,012 (0.2%)	11/2,011 (0.5%)
				2,709 bits (3,412)	2,776 bits (3,496)	2,758 bits (3,474)	2,612 bits (3,290)
WOB2pgp5	6,211–7,441	–	–	26,222–27,448	23,029–24,255	6,273–7,499	823,443–824,669
				1,190/1,233 (97%)	1,166/1,233 (95%)	1,191/1,233 (97%)^*^	1,180/1,233 (96%)
				8/1,233 (0.6%)	8/1,233 (0.6%)	8/1,233 (0.6%)	8/1,233 (0.6%)
				1,781 bits (2242)	1,686 bits (21,22)	1,785 bits (2248)^*^	1,741 bits (2,192)
WOB2pgp6	7,438–7,926	–	–	27,445–27,933	24,252–24,731	7,496–7,984	824,705–825,166
				459/489 (94%)	463/489 (95%)	461/489 (94%)	364/462 (79%)
				0/489 (0%)	9/489 (2%)	0/489 (0%)	1,2/462 (3%)
				659 bits (828)	667 bits (838)	667 bits (838)	337 bits (422)
WOB2pgp7	7,950–8,745	–	–	27,957–28,754	25,283–26,023	8,008–8,805	825,190–825,987
				771/798 (97%)	625/744 (84%)	771/798 (97%)^*^	753/799 (94%)
				2/798 (0.3%)	11/744 (1,.5%)	2/798 (0.3%)	4/799 (0.5%)
				1,159 bits (1,458)	702 bits (882)	1,159 bits (1,458)^*^	1,084 bits (1,364)
WOB2pgp8	8,797–9,288	–	–	28,806–29,297	25,537–26,021	8,857–9,348	826,039–826,530
				460/493 (93%)	448/486 (92%)	465/493 (94%)	475/492 (97%)^*^
				2/493 (0.4%)	2/486 (0.4%)	2/493 (0.4%)	0/492 (0%)
				651 bits (818)	621 bits (780)	671 bits (844)	714 bits (898)^*^
WOB2pgp9	9,336–9,493	–	–	29,343–29,501	26,071–26,220	9,394–9,552	826,578–826,727
				150/159 (94%)	144/150 (96%)	151/159 (95%)	148/150 (99%)^*^
				1/159 (0.6%)	1/150 (0.7%)	1/159 (0.6%)	1/150 (0.7%)
				218 bits (272)	214 bits (268)	221 bits (276)	230 bits (288)^*^
WOB2pgp10	9,642–9,799	–	–	29,650–29,713	26,370–26,430	9,701–9,764	826,877–826,937
				63/64 (98%)	61/61 (100%)	63/64 (98%)	60/61 (98%)
				0/64 (0%)	0/61 (0%)	0/64 (0%)	0/61 (0%)
				99.1 bits (122)	99.1 bits (122)	99.1 bits (122)	94.3 bits (116)
				29,647–29,814	26,367–26,534	9,698–9,865	826,874–827,041
				155/168 (92%)	155/168 (92%)	154/168 (92%)	154/168 (92%)
				7/168 (4%)	7/168 (4%)	7/168 (4%)	7/168 (4%)
				211 bits (264)	211 bits (264)	206 bits (258)	206 bits (258)
WOB2pgp11	10,128–10,594	–	–	30,170–30,639	26,891–27,351	10,222–10,691	827,414–827,883
				439/470 (93%)	431/470 (92%)	438/470 (93%)	444/471 (94%)
				4/470 (0.9%)	1,3/470 (3%)	4/470 (0.9%)	6/471 (1%)
				621 bits (780)	583 bits (732)	617 bits (776)	637 bits (800)
WOB2pgp12	10,727–11,203	–	–	30,774–31,244	27,485–27,942	10,825–11,295	828,029–828,499
				439/477 (92%)	423/464 (91%)	439/477 (92%)^*^	436/477 (91%)
				6/477 (1%)	6/464 (1%)	6/477 (1%)	6/477 (1%)
				603 bits (758)	570 bits (716)	603 bits (758)^*^	591 bits (742)
WOB2pgp13	11,220–11,689	–	–	31,261–31,457	27,966–28,160	11,312–11,508	828,51,6–828,712
				192/197 (97%)	181/195 (93%)	197/197 (100%)	188/197 (95%)
				0/197 (0%)	0/195 (0%)	0/197 (0%)	0/197 (0%)
				294 bits (368)	256 bits (320)	31,4 bits (394)	278 bits (348)
				31,523–31,808	28,238–28,509	11,574–11,859	828,790–828,858
				267/288 (93%)	243/273 (89%)	246/287 (86%)	66/69 (96%)
				6/288 (2%)	3/273 (1%)	4/287 (1%)	0/69 (0%)
				370 bits (464)	313 bits (392)	299 bits (374)	99.1 bits (122)
		1–354		32,392–32760	29,086–29,454	13,027–13,386	–
				341/369 (92%)	339/369 (92%)	334/360 (93%)	
				15/369 (4%)	15/369 (4%)	6/360 (2%)	
				464 bits (582)	456 bits (572)	465 bits (584)	
WOB2pgp14	–	348–21,78	–	32,754–34,575	29,448–31,278	13,380–15,200	–
				1,653/1,832 (90%)	1,664/1,840 (90%)	1,679/1,831 (92%)	
				19/1,832 (1%)	18/1,840 (1%)	22/1,831 (1%)	
				21,84 bits (2,750)	2,209 bits (2,782)	2,287 bits (2,880)	
WOB2pgp15	–	2,176–2,400	–	34,576–34,800	31,276–31,500	15,212–15,430	–
				172/225 (76%)	224/225 (99%)^*^	194/219 (89%)	
				0/225 (0%)	0/225 (0%)	0/219 (0%)	
				167 bits (184)	401 bits (444)^*^	282 bits (312)	
WOB2pgp16	–	2,401–3,785	–	34,801–36,228	31,501–32,928	16,129–16,829	–
Phage portal protein				1,215/1,439 (84%)	1,228/1,440 (85%)^*^	584/713 (82%)	
				65/1,439 (5%)	67/1,440 (5%)	15/713 (2%)	
				1,344 bits (1,692)	1,392 bits (1,752)^*^	610 bits (766)	
				–	–	16,880–17,508	
						540/650 (83%)	
						25/650 (4%)	
						576 bits (724)	
WOB2pgp17	–	3,818–4,888	–	36,255–37,331	32,955–33,967	17,551–18,566	–
Putative minor capsid protein				985/1,077 (91%)^*^	907/1,024 (89%)	809/1,030 (79%)	
				6/1,077 (1%)	12/1,024 (1%)	21/1,030 (2%)	
				1,528 bits (1,694)^*^	1,312 bits (1,454)	830 bits (920)	
WOB2pgp18	–	4,863–5,231	–	37,306–37,667	34,030–34,391	18,629–18,996	–
				331/362 (91%)^*^	299/367 (81%)	323/368 (88%)	
				3/362 (1%)	13/367 (4%)	0/368 (0%)	
				451 bits (566)^*^	303 bits (380)	406 bits (51,0)	
WOB2pgp19	–	5,273–6,280	–	37,715–38,715	34,430–35,429	19,031–20,015	–
				928/1,002 (93%)^*^	918/1,001 (92%)	910/986 (92%)	
				1/1,002 (0.1%)	1/1,001 (0.1%)	1/986 (0.1%)	
				1,297 bits (1632)^*^	1,260 bits (1586)	1,263 bits (1590)	
WOB2pgp20	–	7,485–7,805	–	38,807–39,1,26	35,51,4–35,833	20,1,29–20,193	–
				298/320 (93%)	295/320 (92%)	53/65 (82%)	
				0/320 (0%)	0/320 (0%)	0/65 (0%)	
				422 bits (530)	410 bits (51,4)	57.8 bits (70)	
WOB2pgp21	–	7,798–8,285	–	39,1,20–39,608	35,827–36,306	20,496–20,915	–
				467/489 (96%)	458/489 (94%)	300/422 (71%)	
				1/489 (0.2%)	1,0/489 (2%)	37/422 (9%)	
				689 bits (866)	646 bits (812)	157 bits (196)	
WOB2pgp22	–	8,266–8,698	–	39,589–40,021	36,287–36,761	20,952–21,359	–
				430/433 (99%)	419/478 (88%)	351/409 (86%)	
				0/433 (0%)	6/478 (1%)	2/409 (0.5%)	
				676 bits (850)	521 bits (654)	419 bits (526)	
WOB2pgp23	–	8,726–9,054	–	–	–	–	–
WOB2pgp25	–	–	316–651	40,773–41,08	37,871–38,206	–	–
				326/336 (97%)	328/336 (98%)		
				0/336 (0%)	0/336 (0%)		
				562 bits (622)	571 bits (632)		
WOB2pgp26	–	–	661–1,470	4,118–41,894	38,21,6–39,001	22,471–23,245	–
				739/789 (94%)	747/798 (94%)	535/799 (67%)	
				1,2/789 (2%)	12/798 (2%)	24/799 (3%)	
				1,204 bits (1,334)	1,216 bits (1,348)	250 bits (276)	
WOB2pgp27	–	–	–	41,922–42,083	39,020–39,181	23,439–24,361	–
				136/162 (84%)	136/162 (84%)	672/933 (72%)	
				0/162 (0%)	0/162 (0%)	20/933 (2%)	
				176 bits (194)	176 bits (194)	484 bits (536)	
				42,132–43,139	39,230–40,237	–	
				980/1,008 (97%)	978/1,008 (97%)		
				0/1,008 (0%)	0/1,008 (0%)		
				1,692 bits (1876)	1,683 bits (1866)		
WOB2pgp28	–	–	2,794–3,476	43,160–43,844	40,258–40,939	–	–
				61,7/685 (90%)	543/688 (79%)		
				4/685 (1%)	13/688 (2%)		
				816 bits (1,026)	508 bits (638)		
WOB2pgp29	–	–	3,492–5,414	43,863–45,796	40,958–42,910	–	–
				1,769/1,935 (91%)	1,742/1,953 (89%)		
				14/1,935 (1%)	30/1.953 (2%)		
				2,403 bits (3,026)	2,239 bits (2,820)		
WOB2pgp30	–	–	5,427–6,610	45,822–46,996	42,922–44,108	–	–
				1,076/1,175 (92%)	1,082/1,187 (91%)		
				1/1,175 (0.1%)	4/1,187 (0.3%)		
				1,473 bits (1,854)	1,465 bits (1,844)		
WOB2pgp31	–	–	6,615–6,811	47,001–47,195	44,124–44,310	–	–
				157/197 (80%)	145/191 (76%)		
				2/197 (1%)	6/191 (3%)		
				154 bits (192)	118 bits (146)		
WOB2pgp32	–	–	6,808–8,298	47,192–47,518	44,330–44,651	–	–
				302/327 (92%)	279/322 (87%)		
				0/327 (0%)	0/322 (0%)		
				477 bits (528)	387 bits (428)		
				47,607–48,612	44,740–45,719		
				926/1,018 (91%)	909/992 (92%)		
				12/1,018 (1%)	12/992 (1%)		
				1,424 bits (1578)	1,416 bits (1,570)		
WOB2pgp33	–	–	8,304–8,594	48,626–48,910	45,760–46,051	–	–
				225/286 (79%)	239/292 (82%)		
				3/286 (1%)	1/292 (0.3%)		
				211 bits (264)	254 bits (318)		
WOB2pgp42	–	–	8,593–9,193	55,791–56,398	51,536–52,143	–	–
				581/608 (96%)	581/608 (96%)		
				7/608 (1%)	7/608 (1%)		
				976 bits (1082)	976 bits (1,082)		
WOB2pgp43	–	–	9,121–9,692	56,319–56,900	52,064–52,645	–	–
				488/590 (83%)	488/590 (83%)		
				26/590 (4%)	26/590 (4%)		
				513 bits (644)	513 bits (644)		
WOB2pgp44	–	–	9,831–10,049	57,025–57,243	52,770–52,988	–	–
				216/219 (99%)	21,0/219 (96%)		
				0/219 (0%)	0/219 (0%)		
				337 bits (422)	313 bits (392)		
WOB2pgp45	–	–	10,023–10,922	57,217–58,116	52,962–53,866	–	–
				858/902 (95%)	848/905 (94%)		
				4/902 (0.4%)	5/905 (0.6%)		
				1,255 bits (1,580)	1,208 bits (1,520)		
WOB3pgp45	–	–	11,124–11,696	–	54,016–54,588	–	–
SpvB insect toxin					475/573 (83%)		
					0/573 (0%)		
					522 bits (656)		

† *Predicted gene products are named and numbered in the “gene name” column; homology-based predicted functional information is also provided. Gene names are based on their homology to gene products reported for the WO reference genome WOcauB2 (Kent et al., 2011a). WOcauB2 paralogs gene product is abbreviated as “WOB2pgp” followed by an identifying number; WOB2pgp13 is thus a paralogs sequence of WOcauB2 gene product 13*.

§ *NCBI accession numbers of the three contigs generated in this study are provided directly below their names: sr1WOdamA1 contig 1 to 3*.

‡ *GenBank accession numbers, gene co-ordinates and similarity values for all four of the other sr1WO phage genomes (for a schematic overview of shared sr1WO group genomic architecture see Figure 2). Divergence measurements from WOdamA1 predicted products are displayed for all the paralogs gene sequences that occur in these other four sr1WO genomes. Quoted sequence co-ordinates and similarity values were obtained from individual gene product BLASTn sequence searches of GenBank's non-redundant nucleotide sequence deposits using sr1WOdamA1 predicted gene products as queries and implementing the following search parameters: word size 7; gap opening penalty 0; extension penalty 4. Similarity values are displayed only if they were recovered from the top 10 most significant sequence matches (based on bit scores) found from GenBank's entire non-redundant sequence repository*.

* *Bold type face highlights BLASTn sequence similarity matches that were the most significant sequence matches found in all of GenBank's entire non-redundant sequence repository*.

### The characterization of a *w*Dam WO head and base-plate module prophage sequence element (sr1WOdamA1 contig number 2)

**Figure 3:**
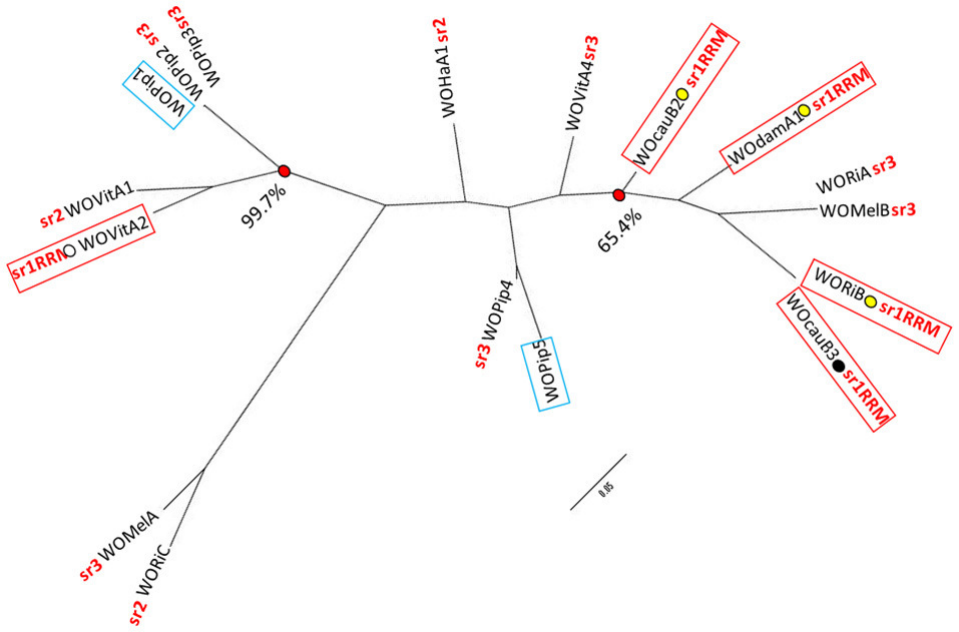
**Representative maximum likelihood tree constructed from a 1031 nucleotide position alignment of 17 WO minor capsid protein gene sequence**. The tree (historically used for WO classification) includes sequences from all the WOs used in Figure 1, as well as two additional WO sequences (from WOPip1 and WOPip5), the genomes of which lack integrase/recombinase genes (Kent et al., 2011a). The gene sequences of WOPip1 and WOPip5 are highlighted with blue boxing. Minor capsid gene sequences originating from WOs with the group 1 serine replication recombinase module (sr1RRM) referred to in the main text are indicated, as are their integration sites (labeled as in Figure 1). The serine recombinase-based phylogenetic classification of these WOs is also highlighted, using branch tip suffixes written in red (sr1–sr3). The bootstrap support for two monophyletic groups containing WO sequence representatives with varying integrase/recombinase gene sequences is also shown. The minor capsid protein sequences alignment is provided in Supplementary File 4.

*w*Dam WO prophage contig 2 (NCBI accession number KY695240) is 9.054 kb and spans from the 3′ end of our WOcauB2 gp13 paralog to the middle of our WOcauB2 gp23 paralog (Figure 2). It contains a block of 10 WOcauB2 gene sequences which, as shown in Figure 2, code for genes corresponding to what Kent et al. (2011a) defined as WO head and base-plate modules. It also includes gene sequence coding for the minor capsid (orf7) protein, which is a B2gp17 paralog and has been used to construct the phylogenetic tree shown in Figure 3. The 10 whole gene sequences that occur in contig 2 appear in the same order and orientation as their paralogs in the WOcauB2 genome. The synteny between the WOcauB2 and sr1WOdamA1 genomes is only interrupted by the existence of a transposable element-like sequence occurring between the sr1WOdamA1 WOcauB2 gene protein paralogs B2gp19 and B2gp20.

### The characterization of a *w*Dam WO tail module prophage sequence element (sr1WOdamA1 contig number 3)

*w*Dam WO prophage contig 3 (NCBI accession number KY695241) is 11.696 kb in length and contains 14 predicted gene sequences, spanning from a WOcauB2 gene protein 25 paralog at one end through to a WOcauB3 gp45 gene protein paralog at the other end (Table 2 and Figure 2). As can be seen in Figure 2, this contig contains the 3′ prime end of the phage base-plate gene modules as well as its virulence and tail regions (Kent et al., 2011a). The first predicted nine gene sequences in this contig correspond to paralogs of WOcauB2 gene protein sequences spanning from 25 to 33 (Table 2 and Figure 2). This *w*Dam WO prophage sequence element then appears to have a deletion. Thus, after this nine-gene block, there is a 3′-truncated gene protein sequence (a paralog of the WOcauB2 gene protein 33), which is immediately followed by a block of five gene sequences corresponding to paralogs of WOcauB2 gene proteins B2gp42 to B2gp45 (Table 2 and Figure 2). At the extreme 3′ end of this prophage sequence element and contig 3 (directly after the WOcauB2 gp45 paralog), there is a gene sequence matching the B3gp45 gene protein (see below) which has no paralog in the WOcauB2 genome. Repeated efforts to close a predicted gap between contigs 2 and 3 failed; it is, thus, unclear if there is a WOcauB2 gene protein 24 paralog within the genome of sr1WOdamA1 or not. The extreme 5′ end of contig 3 contains what we propose here is a 217-nucleotide transposable element sequence with all the features of a Miniature Inverted-repeat Transposable Element (MITE), including 24-nucleotide inverted terminal repeats (ITRs), as well as a 9-nucleotide target-site duplication (Delihas, 2011). This MITE—named here as *w*Dam-MITE-1—could be the beginning of a stretch of repetitive DNA lying between the head and base-plate and tail modules (i.e., contigs 2 and 3) of the sr1WOdamA1 genome that was too long for our PCR bridging efforts to gap-close.

### Preliminary characterization of the *w*Dam genomic DNA flanking the *FtsZ* cell-division gene: a possible *Wolbachia* genome target site for sr1 group WO integration

BLASTn and tBLASTx homology searches of the shot-gun sequence data using the 16,703 nucleotide sequence reported to be upstream of WOcauB3 did not identify any significant sequence matches. However, four shot-gun sequence contigs (representing 5.265 kb of unique DNA) showed significant levels of homology with the upstream sequence of WOcauB2. As shown in Table 1, these four contigs show high levels of sequence identity with six of the 16 genes found immediately upstream of the WOcauB2 and WOri relic serine recombinase prophage sequences. Importantly, the gene sequences in these contigs correspond to four of the five gene sequences that are closest to the WOcauB2 and WOri relic integration sites. Hence, our 454 sequence run recovered DNA sequence matching the 4,239 bp immediately upstream of the WOcauB2 prophage and the 3,769 bp sequence immediately upstream of the WOri relic prophage (Table 1). In contrast with the other four genes immediately upstream of the WOcauB2 and WOri relic prophages, BLAST searches revealed that the gene for which we did not recover a paralog (corresponding to ACN95502 in *w*Ri and BAH22204 in *w*Cau) is incompletely conserved among the *Wolbachia* strains. The absence of this gene from the shot-gun sequence reads could be a consequence of its absence from the *w*Dam genome. Sequence comparisons made between the *w*Dam *FtsZ* gene contig recovered from our shot-gun sequence reads and the previous report confirmed that the *FtsZ*-positive BACs used in this study were of the same origin as the *FtsZ* gene first reported in 2010 (Crainey et al., 2010a). The 454 sequence contig showed >99% sequence identity with the previously published *FtsZ* (FN563974) sequence.

PCR screening for sr1WOdamA1 gene sequences within the seven *FtsZ*-positive BACs used in the original shot-gun sequence run, identified three colonies as containing sr1WOdamA1 phage sequences. Consistent with the notion that a full-length sr1WOdamA1 prophage element occurs immediately adjacent to the *FtsZ* gene of *w*Dam, one *FtsZ*-positive BAC was confirmed (by two independent PCR reactions) as containing the sr1WOdamA1 serine recombinase gene (i.e., a WOcauB2 gp1 paralog) and also B2gp33 and B2gp34 paralogs (by two independent PCR reactions). As previous analysis of BAC clones from the *S. squamosum* E BAC library used in this study suggested that the average BAC contains around 128 kb of cloned DNA and the sr1WOdamA1 genomic sequence is over 32 kb, this strongly suggests that the sr1WOdamA1 prophage has integrated within 100 kb of the *w*Dam *FtsZ* gene. Unexpectedly, two BACs tested positive (in two independent PCR tests) for just the tail-end of sr1WOdamA1 (i.e., the gp31 and gp32 genes).

### All three *w*Dam prophage sequence elements likely derive from an inactive sr1WOdamA1 prophage relic

The three contigs recovered in this work can be seen to correspond, roughly, to three distinct (and non-overlapping) WO modules (1–3), that can be considered, when compared to the WOcauB2 reference genome, collectively to make-up a near complete sr1WO genome. *w*Dam WO contig 1, contains a replication and repair module thus far only associated with WOs that contain sr1 recombinases (sr1RRM); the *w*Dam WO contig 2 contains a head and base-plate module and some “virulence” genes, and *w*Dam WO contig 3 is a tail module (Figure 2). While the exact relationship between these *w*Dam WO prophage sequences is presently unclear, the gene sequence order and orientation within these contigs can be seen to be almost identical to those reported for the WOcauB2 and WOcauB3 phage genomes. Although we acknowledge that alternative explanations for our results may exist (see Section Discussion), we believe that the most parsimonious explanation is that all three of the *w*Dam WO contigs reported here derive from the same sr1WO genome, which we are proposing be named as sr1WOdamA1. In relation to the reference genome WOcauB2, the sr1WOdamA1 appears to be missing nine phage gene sequences corresponding to WOcauB2 gp24 and gp34–41 (Figure 2), but to contain paralogs for all other WOcauB2 genome sequences.

In WOcauB2, and the other serine recombinase phage genomes that have paralogs, the genes gp34–41 (and their paralogs) have been ascribed tail functions (Figure 2 and Table 2). As this deletion begins following a 3′-truncated gp33 paralog, our findings indicate that sr1WOdamA1 lacks paralogs for these sequences because of a recent deletion and, thus, that sr1WOdamA1 is missing genes that its progenitor contained. As, however, this part of the tail region is highly variable among WOs and some, including other sr1WO group bacteriophages like WOvitA2 (Figure 2), lack recognizable tail modules, it is possible that sr1WOdamA1 and/or its progenitor retained active functions in the absence of a gp34–41 section. On the other hand, the absence of a WOcauB2 gp24 paralog from the sr1WOdamA1 genome, a well conserved gene component of the highly preserved phage base-plate region, is likely to render sr1WOdamA1 immobile. Although our failure to detect this gene could be an artifact of its expected location occurring in the break between two of our three sr1WOdamA1 contigs (Figure 2 and above), we believe that it is more likely that the gene has been disrupted by a transposable element integration. Our analysis identified a *w*Dam-MITE-1 transposable element sequence at the extreme 5′-end of contig 3, immediately downstream of sr1WOdamA1's WOcauB2 gp25 gene protein paralog (where the WOcauB2 gp24 gene should occur). Because of this and because there was no trace of a WOcauB2 gp24 paralog detected from our initial shot-gun sequence run, we believe that the sr1WOdamA1 genome reported here is probably a dysfunctional prophage relic.

### *w*Dam WO prophage sequences are isolated from both non-*wolbachia*-infecting bacteriophage and other WOs

As shown in Table 2, most BLAST searches with the *w*Dam WO prophage sequences recovered in this work, returned best match paralogous sequences from the genomes of other sr1WO group prophages. In every search performed with our 36 predicted sr1WOdamA1 gene sequences, the paralogous sequence matches listed in Table 2 were among the top ten closest matches in the non-redundant NCBI sequence repository. In addition to this, every search returned a best match sequence annotated as deriving from a *Wolbachia* genome or WO. Moreover, when the search results returned five or more significant matches, the top five hits were always annotated as not just deriving from a *Wolbachia* or WO genome but to be also sourced from an arthropod.

Figure 1 presents a phylogenetic tree constructed using WO recombinase genes and shows that the *w*Dam WO phage sequence element recovered in contig 1 belongs to the same group of serine recombinase WOs to which WOcauB2 and WOcauB3 belong, and that was previously identified in the analysis of both Kent et al. (2011a) and Wang et al. (2013). As mentioned above, in addition to sharing closely-related recombinase genes, this group of five phages (WOcauB2, WOcauB3, WOvitA2, WOri relic, and now sr1WOdamA1) share an sr1RRM (spanning around 11 kb), which is not found in other (unrelated) WOs and corresponds almost exactly with contig 1 (Figure 2). Gene order and orientation is near perfectly conserved in the sr1RRM, with nine conserved WOcauB2 spanning gp1–6 paralog sequences recognizable in sr1WOdamA1 and all other serine recombinase WOs (Table 2 and Figure 2). Our first six predicted gene sequences have clear paralogs (appearing in the same order and orientation) and the last two gene sequences of the module (including a Holliday junction recombinase) have clear paralogs in all five phage genomes (Table 1 and Figure 2).

As shown in Table 2, BLAST searches (against the NCBI's entire non-redundant sequence database) with the 12 *w*Dam WO contig gene sequences from this module returned best match sequences deriving from another serine recombinase WO eight times. In most cases the *w*Dam WO-predicted gene sequences share similar levels of identity (>90%) with the other predicted phage gene sequences. For five out of eight of these genes, all differences between the sequences and their closest ones in the database are attributable to nucleotide substitutions, suggesting that these genes have been the subject of point mutation-based evolution. Indications of recombination, however, can be seen in Table 2. For example, most WOvitA2, WOri relic, WOcauB2, and WOcauB3 genes show similar levels of divergence from their sr1WOdamA1 paralogs, but the WOvitA2 B2gp3 paralog is markedly closer than the others. Similar signs of point-mutation-based WO evolution and of between-WO gene recombination are also evident from the BLAST search returns of *w*Dam WO prophage head base-plate gene sequences (contig 2) and tail module gene sequences (contig 3) (Table 2).

### The *w*Dam WO prophage tail-module sequence element harbors an SpvB protein homolog at its terminal end

In addition to the 36 *w*Dam prophage genes with paralogs in the WOcauB2 genome that were identified from the three prophage sequence elements recovered in this work, an SpvB-like protein was observed to occur at the terminal end of the *w*Dam WO prophage tail module element (contig 3). BLASTn searches with the last 377 nucleotides of contig 3, best match the first 378 nucleotides of the WOcauB3 gp45 protein which is annotated as coding for an SpvB-motif protein (BAH22314). The two sequences share 83% identity (315/378). The second-best (and only other significant) match is with the first 378 nucleotides of the *Wolbachia* phage wNo_WO4 “SpvB and TcdB toxin domain protein” (AGJ99401), which shares 79% identity (299/378). BLASTx searches also provided best matches with these gene sequences (83 and 85% identity across 110 residues, respectively), as well as support for this gene having an insecticidal toxin function. Thus, while there are presently no other close relatives to these proteins in the NCBI database, the next 17 best matches are all with bacterial proteins, which share between 50 and 60% amino acid level identity and have similar properties to those predicted for the BAH22314 and AGJ99401 proteins. All 10 of these hits that have functional annotation, are described as SpvB proteins and/or toxins or “insecticidal toxins.” As in the WOcauB3 genome, the SpvB-like protein appears to occur at the terminal tail end of the *w*Dam WO tail module contig.

## Discussion

In previous work we reported a novel *Wolbachia FtsZ* cell-division gene sequence and showed the genome of this *Wolbachia* to be well represented in a BAC library prepared from *S. squamosum* E blackfly larvae (Crainey et al., 2010a,b). In this work we have taken the first step toward characterizing this bacterium's genome and have provided evidence that it harbors *Wolbachia* prophage sequence elements close to its *FtsZ* cell-division gene. Following gap-closing PCRs, we have resolved >32 kb of WO prophage sequence elements, corresponding to three distinct WO functional modules, namely, an sr1RRM, a head and base-plate and a tail module. Although alternative explanations may exist (see below) as to why we recovered these three WO sequences from the pool of *FtsZ*-gene positive BACs sequenced, the most parsimonious explanation is that they all derive from a single WO prophage genome that occurs close to the *FtsZ* cell-division gene.

Alternative explanations may include, for instance, the generation of chimeric BAC clones, created during the cloning process by ligating WO and *w*Dam genomic fragments (with different origins and which do not occur close together in the *w*Dam genome in nature). This would have required these sequences co-incidentally being cloned into the same BAC vector. However, there are good reasons to doubt such an explanation. Firstly, BAC libraries have been widely used in genome research for over 30 years and reports of such chimeric ligation being generated by the cloning process are extremely rare. Second, our former characterization of the BAC library used for this work suggests that *w*Dam DNA represents only about 1% of the total cloned DNA (Crainey et al., 2010a). Hence, one would expect that there is about a 99% chance that any randomly created BAC chimera including a *w*Dam genomic fragment would be composed of *w*Dam and non-*w*Dam DNA (i.e., most likely *S. squamosum* genomic DNA). The PCRs we did on our BAC clones showed that three of our *FtsZ*-positive BACs contain both *w*Dam genomic DNA and WO phage sequences; thus, this would require three such events to have occurred (each with a 1% chance) ignoring that the cloning process only very rarely creates chimeric BAC clones. Therefore, the possibility that our results are explained by such a phenomenon is in the order of one in a million. We are, therefore, reasonably confident that all the *w*Dam WO sequences recovered are from the *w*Dam genome and thus of prophage origin. As a result, we are tentatively proposing that they are all from the same WO prophage genome, named here sr1WOdamA1. This sr1WOdamA1 is most similar to the *Wolbachia* phage from the almond moth *Cadra cautella*, WOcauB2 (Tanaka et al., 2009). Several similarities exist between our sequences and other WOs, as well as unique aspects which we discuss in relation to phage evolution, *Wolbachia*-based disease control programmes and the relevance of sr1WOdamA1 to onchocerciasis epidemiology below.

### The *Wolbachia* prophages of *w*Dam show indications of an integration-site preference

Traditional phylogenetic classification of *Wolbachia* phages has focused on the minor capsid or “orf7” gene (Bordenstein and Wernegreen, 2004; Gavotte et al., 2007; Chafee et al., 2010). More recently, however, WO researchers have begun performing phylogenetic analysis on the integrase/recombinase genes of WOs (Kent et al., 2011a; Wang et al., 2013). To classify our novel *w*DamWO prophage sequence elements, we used both approaches (Figures 1, 3). Using the serine recombinase gene of the *w*Dam WO sr1RRM prophage sequence module, our analysis resulted in the same four serine phylogenetic groupings generated by Kent et al. (2011a) and Wang et al. (2013), and showed that this *w*Dam WO prophage sequence element, at least, belongs to a cluster of four other serine recombinase phages (sr1WOs) that share several structural features (Figures 1, 2). Phylogenetic analysis with the minor capsid gene from the *w*Dam WO head and base-plate module prophage sequence element (Figure 3), by contrast, did not share the same degree of congruence with WO structural features or agree well with the phylogeny constructed using the serine recombinase genes. Supporting the notion that our *w*Dam WO sr1RMM prophage element and our *w*Dam WO head and base-plate modules derive from the same (sr1WOdamA1) genome, the minor capsid gene phylogeny shown in Figure 3, clustered the *w*Dam prophage minor capsid gene in a bootstrap-supported monophyletic group with the genes of three other sr1 group WOs. This group, however, also contained two non-sr1 group WOs and excluded the sr1group WOvitA2 bacteriophage, supporting previous reports that this gene has been exchanged between WO families via recombination and suggesting that this is not a reliable gene for WO classification. As the conservation of the sr1RRM probably reflects a fundamental difference in phage life-cycle and serine recombinase-based phylogeny grouped all of the WOs that share this feature together, we think that classifying and naming our phage based on this feature (rather than by its minor capsid protein) has more biological meaning.

With the inclusion of sr1WOdamA1 in the sr1WO group, the latter can be considered as, currently, having five members, namely sr1WOdamA1, sr1WOvitA2, WOcauB2, WOcauB3, and WOri relic (Figures 1, 2). While we have been unable to resolve completely the modular organization of sr1WOdamA1 recorded here, we have been able to resolve most of its within-modular structure, and from this it is apparent that gene sequence identity, gene sequence order and orientation are all well preserved among this group (Figure 2 and Table 2). Our results do, however, suggest that modular architecture of sr1WO group bacteriophages may vary as for many other WO families (Klasson et al., 2008; Kent et al., 2011a). Thus, while the occurrence of the B2gp13 homolog gene sequence—corresponding to the first portion of the gene—at the end of contig 1, and the occurrence of the B2gp13 homolog gene sequence—corresponding to the end of the gene—in contig 2, strongly suggest that they are from the same WO genome, the fact that we were unable to bridge the gap by PCR suggests that they may not be orientated in the same way as the WOcauB2, WOcauB3, and WOvitA2 genomes (Figure 2). Consistent with the idea of variant modular architectures occurring within the sr1WO group, the “terminal” end of the WOri relic (like the end of the sr1WOdamA1 contig 1) corresponds to the 5′-end of a B2gp13 paralog (Klasson et al., 2009). The sr1RRM of the sr1WO phage group may have become separated from the head and base-plate modules (and therefore not occur in sr1WOdamA1 as they do in WOcauB2, WOcauB3, and WOvitA2 genomes) in the progenitors of this relic and the sr1WOdamA1 prophage. A variant modular organization of sr1WOdamA1 may also help to explain the non-joining of contigs 2 and 3 (and thus the sr1WOdamA1 head and tail modules).

In addition to shared sequence and structural features, some of the sr1WO bacteriophages also share a common integration preference. The occurrence of the sr1WOdamA1 genome within BACs that contain four of the five genes immediately upstream of two other sr1 group prophages (WOcauB2 and a WOri relic) suggest that the sr1WOdamA1 prophage belongs to a group of *Wolbachia* prophages with a target site preference. This observation and the fact that most of the sr1RRM genes do not have clear paralogs in the genomes of other *Wolbachia* phages, suggest that the sr1RRM prophage may be involved in a common and targeted genomic integration method. However, it should be noted that while the WOri relic prophage, WOcauB2 and sr1WOdamA1 all appear to have integrated close to the *Wolbachia FtsZ* gene, the WOcauB3 appears to have integrated at a different genomic location (Klasson et al., 2008; Tanaka et al., 2009). As more serine recombinase *Wolbachia* phages have their genome sequences and integration sites resolved, the nature of this apparent genomic targeting will become better understood. Our observation that the tail end of sr1WOdamA1 appears to be closer to the *FtsZ* gene than the recombinase gene, suggests that the integration process may not require the bacteriophage to be integrated in a fixed orientation and highlights how little is presently known about the process by which *Wolbachia* phages integrate into their host *Wolbachia* genomes, with the data presented here contributing substantially to the current knowledge base.

### sr1WOdamA1: evolution and relevance to *Wolbachia*-based disease control strategies

Modular theory predicts that phages can exchange gene sequences freely across a broad range of ecological niches (Kent et al., 2011b; Metcalf and Bordenstein, 2012). It has been proposed, however, that the normal rules of modular evolution do not apply to WOs and that, while WOs can exchange gene sequences among themselves, they do not appear commonly to exchange genes with non-*Wolbachia* phages (Kent et al., 2011b; Metcalf and Bordenstein, 2012). The BLAST searches performed with each of the sr1WOdamA1 36 predicted gene sequences, returned a best match sequence deriving from a previously characterized WO sequence. In most cases the best match sequence was from another serine recombinase WO, suggesting that there may $$even be restriction of gene flow between WO subgroups (Kent et al., 2011b; Metcalf and Bordenstein, 2012). In line with previous analysis, however, these searches did provide clear evidence of genetic exchange between sr1WOdamA1 and other WO sequences that infect arthropod-infecting *Wolbachia* (Klasson et al., 2008; Kent et al., 2011b; Wang et al., 2013).

Although evidence of frequent *Wolbachia* phages horizontally transferring between strains has recently emerged, the evidence for this has been entirely based on the minor capsid protein and, thus, the structure and biology of the bacteriophages involved in these transfers have hitherto been completely unknown (Wang G. H. et al., 2016; Wang N. et al., 2016). In this study, we have isolated three novel WO prophage sequence elements (which probably all derive from the same WO genome) from the *w*Dam *Wolbachia* genome. The *w*Dam *Wolbachia* strain is the first from outside the A and B super clades to be shown to be infected with an sr1WO group bacteriophage (Crainey et al., 2010a; Kent et al., 2011a; Ellegaard et al., 2013). This has two important implications for *Wolbachia*-based disease control strategies. Firstly, it suggests that artificially-introduced *Wolbachia*, like those being used to control *Aedes aegypti*-transmitted dengue in Australia, Brazil and elsewhere, could themselves be infected by naturally occurring phages (Hoffmann et al., 2011, 2015; Caragata et al., 2016). Given the present plans to expand the use of *Wolbachia*-based disease control techniques and the possibility that phage integrations into artificial *Wolbachia* infections could impact on vector characters of epidemiological importance, this is not a trivial observation but one that may have wide-reaching consequences (Woolfit et al., 2013; Sutton et al., 2014; Jeffries and Walker, 2015). For example, although variant strains of *w*Mel *Wolbachia* bacteria currently used for control appear to be near-identical in gene-coding regions, very minor differences in repeat-region sequences have a major impact on the longevity (and thus epidemiological importance) of *Ae. aegypti* (Woolfit et al., 2013). On the other hand, this observation suggests that if a WO can be manipulated to modify genetically *Wolbachia* (such as lambda, which is routinely used to infect *E. coli*), one phage could potentially be used to modify a broad range of *Wolbachia* stains (Tanaka et al., 2009; Kent and Bordenstein, 2010; Wang et al., 2013). In this context, our discovery that sr1WOdamA1 and WOcauB2 belong to a group of phages that may integrate into a single *Wolbachia* genomic site is particularly interesting as it suggests that they may be adapted to provide a genetic modification system for *Wolbachia*.

### The relevance of *w*Dam WOs to onchocerciasis epidemiology

Because some *Wolbachia* strains seem to be able to change radically and spontaneously the way in which they infect their insect hosts (for example, from exerting a cytoplasmic incompatibility to male-killing), WOs have long been suspected as having an important influence on these characteristics (Kent and Bordenstein, 2010; Metcalf and Bordenstein, 2012). Thus far, however, precious little evidence has been uncovered to support this hypothesis. The existence of an SpvB-like protein at the extreme terminal end of the WOcauB3 phage is regarded as the best evidence yet that these bacteriophages could influence the insect host as this gene is believed to have insecticidal properties (Kent and Bordenstein, 2010; Metcalf and Bordenstein, 2012). The impact that *w*Dam and sr1WOdamA1 might have on *S. squamosum* E is presently unknown (Crainey et al., 2010b), but the occurrence of an SpvB-like gene at the terminal end of the tail module of the WO prophage sequence elements isolated in this study suggests that the WOs of *w*Dam could be influencing *S. squamosum* E biology.

Male-killing is a common form of reproductive parasitism induced by *Wolbachia* (Zug and Hammerstein, 2015). Selective expression of insecticidal proteins such as SpvB in a male insect environment could provide a molecular mechanism for such a phenomenon (Kent and Bordenstein, 2010; LePage and Bordenstein, 2013; Metcalf and Bordenstein, 2012). Although there are presently no reports of male-killing *Wolbachia* infections in the *S. damnosum* s.l. complex, which contains the most important vectors of human onchocerciasis in Africa (including *S. squamosum* E), efforts to get the species into laboratory colonies have repeatedly failed because the entire population has become female over time (Simmons and Edman, 1982; Raybould and Boakye, 1986; Crainey et al., 2017). If the *Wolbachia* infecting *S. squamosum* E is promoting its spread by male-killing, this could be expected to increase the proportion of female flies in the onchocerciasis foci where this species occurs, and this could, in turn, be expected to increase disease transmission in areas where such infections occur. Similarly, it would suggest that antibiotic treatment might aid getting this notoriously difficult species into laboratory colonies.

## Conclusions

In this study we have shown that the genome of a genetically-distinct *Wolbachia* named here as *w*Dam harbors at least one serine recombinase *Wolbachia* prophage relic. Although the three prophage sequence elements we have characterized correspond to three distinct non-overlapping WO functional modules (and could in theory have multiple origins), we believe that they almost certainly all derive from a single WO genome that we have named sr1WOdamA1. Although this WO is unlikely to be active, its existence in the *w*Dam genome implies that active, naturally occurring bacteriophages can infect a broad range of genetically diverse *Wolbachia* strains and that naturally occurring WOs could pose a greater risk to the artificial *Wolbachia* infections currently used for disease control than previously thought. The occurrence of an sr1RRM prophage sequence in the same BAC clones in which the *FtsZ* gene is found is consistent with the notion that at least some of the sr1WO group WOs, notably the WOcauB2 phage, may have a target site preference and could be used for targeted introduction of recombinant genes into *Wolbachia* genomes. The occurrence of an SpvB gene in the genome of the *w*Dam WO prophage sequences suggests that these genes may be a more common feature of *Wolbachia* bacteriophages than hitherto realized, an observation consistent with previous proposals that WOs could be important drivers of *Wolbachia* reproductive parasitism and thus could be causing male-killing in the onchocerciasis *S. damnosum* s.l. species complex with implications for the laboratory colonization of vector species and the epidemiology of onchocerciasis.

## Author contributions

RJP, MGB, and RAC secured financing for this work. JLC, RJP, MGB, and MDW contributed to the planning of the work. JLC performed the laboratory work, except for the Sanger sequencing which was done by CEG, JLC, JH, and CPMA performed the DNA sequence analysis. RJP, RAC, and PHLL collected *S. squamosum* E. MGB, RJP, RAC, and PHLL made substantial contributions to the interpretation and editing of the manuscript. All authors read and approved the final version of the manuscript.

### Conflict of interest statement

The authors declare that the research was conducted in the absence of any commercial or financial relationships that could be construed as a potential conflict of interest.

## Abbreviations

BAC: bacterial artificial chromosome
BLAST: Basic Local Alignment Search Tool
bp: base pair
*FtsZ*: Filamenting temperature-sensitive mutant Z
gp: gene product
ITR: inverted terminal repeat
kb: kilo base
MITE: Miniature Inverted-repeat Transposable Element
nts: nucleotides
PCR: polymerase chain reaction
pgp: paralogous gene product
SpvB-like protein: *Salmonella* virulence plasmid B protein homolog
sr1RRM: group 1 serine recombinase and replication module
TcdB toxin: *Clostridium difficile* toxin B
WO: *Wolbachia* bacteriophage.
