# Supplementary material for: The Genomic Architecture of Novel Simulium damnosum Wolbachia Prophage Sequence Elements and Implications for Onchocerciasis Epidemiology
Source: Front Microbiol. 2017 May 29;8:852. doi: 10.3389/fmicb.2017.00852 (PMC5447182; doi:10.3389/fmicb.2017.00852)
Supplement: Supplementary file 1 [file DataSheet1.PDF]

The Genomic Architecture of Novel Simulium damnosum Wolbachia Prophage Sequence Elements and Implications for Onchocerciasis Epidemiology

James Lee Crainey, Jacob Hurst, Poppy H.L. Lamberton, Robert A. Cheke, Claire E. Griffin, Michael D. Wilson, Cláudia Patricia Mendes de Araújo,
María-Gloria Basañez, Rory J. Post

SUPPLEMENTARY FILE 1 | A library of Wolbachia bacteriophage (WO) protein sequences in FASTA format

```
>WP0239_wPip_WO1_CAQ54347_Putat
MANISIRYQIAQKVRSWRLKRGYTQKDLAGKIGVTYQIVLQYEKGIRKIS
IEKLYAIAKVLSVDIIDLIPVSNEKICLEDEEEEILDLVREYKKINDQEL
RKMFCLLTKFVKVSEKSSKKSEKIKIAKGMVKAGISVDIVAKTIGLSVDE
CVEEKVGSIYCQIGKKIKEWRLVRGYTQKDLAEKMSTTRDEISNYEQGRV
AIPLEKLYAIAETLSINIMDLLELTEDADDKVENELPNLIEEYKEIESQE
LRYALIKFLFESIQICEEKVKRVEKMKIAKDLVKGEISTDIILQITGLSL
GEIQQ

>WP0240_wPip_WO1_CAQ54348_CHP
MALSKFLDARNDYAFKRIFGTEKNKDILIHFLNDILGFTGLAAIHDVEFL
ATILDPEIAAKKQSIVDVLCKDSQGSRYIIEMQFTKTKGFEKRAQYYAAK
AYSSQADQGDNYHNLKEIIFIAVADCIIFPDKADYKSNHVILDQNSFEHD
LKDFYFVFIELPKFTKTKEDQLENIVEKWCYFFRYAAETREEDLDKIVGS
DVIIKRAYEEMNKFNWSEEELLAYEQMKKRIMDEIAAFAQKFDEGLRVGQ
EKGRQEGRQEEKIEVAKNSLKAGVSIDVIAQITGLSHSEISQLKEKT

>WP0241_wPip_WO1_CAQ54349_HP
MSSYISWAKNLAASTFSIIPGLPAQYNIADKNNVKSDNKNIPQSTSSVGW
NKFLNNENIALASCVADALDNTPSRRYQDLMSKGVEVVPSRRVAVEFALK
KFNSFVEDKIRNLDSKEQARIRVELKDAYPEITASLERGVEFSGNVGLDN
VLEKCKKCFCTNVLPKEVSTCLSDIGVTKLEGNLNR

>WP0242_wPip_WO1_CAQ54350__Puta
MLLPPNATKQEKALVDATDYRVDPSRIRGFKFRLEERILPWLIEEYCKLF
RGSAQ

>WP0243_wPip_WO1_CAQ54351_Basep
MQQPNIIEPLNFEEIFFRMKEELVSRDASFTGLVESDPAIKILEVAAWRE
LLLRQRMNEAVKSNLLKFAKGEDLDNLAEFYGVEREKEEEDERFRKRVKA
KIAGWSTGGSKEYYKYHALSADSRVKDALVESPIPGKVQISILSTQTGIL
SEELLEIVKKQVTRDDIRVLTDTVTVVGCNITEIDIHSRMSISPVISKEE
IKKQFIEKFELAKRLGWSVTRSWIIANLFVDGVENVELIEPKEDVVVLGN
ECANLRNLKIELN

>WP0244_wPip_WO1_CAQ54352_Basep
MKGMDAKTGKALEGIEHLKQSVVDILTTPINSRVMRRDYGSRLFELVDKP
INRDLTLEIYAATAEALGKFEKRFKLEKVKITEVKEGRVTLNLEGVYLSE
GKFININGVVV

>WP0245_wPip_WO1_CAQ54353_HP
MNKSVVRVGDHCAEATPHFCVSGSNNVFVNGKPVCRKGDNFTEGRALTEG
SKTVFANGYSIGRVGDIVSCGFKVIKGSESVFAK

>WP0246_wPip_WO1_CAQ54354_Basep
MLDHSFAISELNRKLANVIRIGVVKEIDYEKAKVRVKIGEFLTDWLPWIT
SKAGKDRDWSPPDIDEQVVILSPLGELSLGVVLPGIYQQKYSAPENKKEI
NSVKFQDGTRLLYDKEKHHLEIEVMDKVTLKAGESSIEMTKSGIKLKADK
INLN

>WP0247_wPip_WO1_CAQ54355_HP
MNWQDLHKAICTKLKEEISAIQTCEIYPAIRKELLAPAVFVELSGFEKGH
DPGTEELALKARFEARIVIDSTIENAPVIVRTLAAEVARVVNKNTWNVEN
VSPGEFISAEIDGFRPELDAYLVWVVDWVHEIHIGKSVWSETGIKPHIIE
IGNVHVGS

>WP0248_wPip_WO1_CAQ54356_Puati
```

MSVHIEVEVIESANAKRKKIELAIVRALNRTALWLKSKAAKKISEEKKSL
IRKRLRIFKAKTSRLEVLIRANLYDIRASTIGKIQKTRRGSKVGKHEFIG
GFAAVMPKGNSGMFKREGRAALPIKEVKLPLEPEASRIIENFVNYEVEKV
FEKFFHRELSYITPI

>WP0249_wPip_WO1_CAQ54357_HP
MQENIKRLLEDCFAHLGEVALYESKNKAYMVQVLKQQPDKLYEIGEGQFV
GEMLILEVSVFDILKPMVGDIFVIGNCKYKVHSLPLRDKSGMIWRIEASG
V

>WP0250_wPip_WO1_CAQ54358_HP
MQNPFTNTAFSMTALTNAMNILPINYGRVENLNLFPNRSVRFRHITIEEH
NGVLSLLPTQVPGAPATVGKRGKRKVRTFTIPHIPHDDVVLPEEVQGIRA
FGSESELKALADVITDHLQLMRNKHAITLEHLRMGALKGIILDADGSELL
NLYNEFEITPKVVNFALGTATTDVKRKCLEVLRHIEDNLSGEYMTGIHAL
ISPEFFDALTSHTKVKEAYERWQEGAALRNDMRSGFTFCGITFEEYRGQA
TDPEGTVRRFIEKDTGHCFPLGTASTFTTYFAPADFNETVNTLGQPLYAK
QEPRRFDRGTDLHTQSNPLPMCHRPGVLVKVAIA

>WP0251_wPip_WO1_CAQ54359_HP
MISITEGNNLGDLLKYEVSNLYSRDQITVAKGQNLKLGEVVAKKTEDGFI
RVLNPVGTDGTQTAIGAIVSDVNATENSKAVIITRGAILADHAVVWPAGI
TEEQKAAAIKQLEGRGIIVRKGV

>WP0252_wPip_WO1_CAQ54360_Putat
MWINKPVMVERRSFELLSLYNSKQPIFKNLKHFHINPKGIAIIRIYGVLT
KKTEAFDHILDMTSYENIHEEIESALEDKSIETILLDIDSPGGEVNGVFD
LADFIYGVRGKKRIIAIANDDAYSAAYAIASSAEKVFVCRTSGVGSIGVI
ASHIDQSGFDEKQGIKYTTIFAGKRKNDLNPHEPMTSESLESLQKEVDRL
YEMFVQLIARNRGLSIEKIRSTEAGLYFGEKAVEIGLADGVTTFFEFINK
GENTMNKQTTTDLEIDDLETDNLTKYRTEIVELIRLCNLSRMPEKIGEFI
EQGVSIEQAREVLMELLAERTKKTEILSAIPQNSQEDLMTQVAKSRCI

>WP0253_wPip_WO1_CAQ54361_Putat
MLLKTFKQLFNKPKIKSSAWDTAGSGRRFFHFQPELGSINNLLSQSLETL
RSRSRDMVRKNPYAANIIDTIVSNSIGTGIKPQSKAKNAEFRKKVQELWL
KWTDEADSSGVSDFYGLQALVCRSMIEGGECFVRLRNRKLEDGFSVPLQL
QVLESEHLDNKSNQTLANGNIIRSGIEFNRLGQREAYYLFREHPGEGSFG
ESVRVPANDVLHIYKPLRPGQIRGEPWLSNILLKLYELDQYDDAELVRKK
TAAMFAGFITRLDPEANILGEGESNEQGVALSGLEPGTMQLLDPGEDIKF
SEPSDVGGSYEAFMRQQLRAIAIGTGITYEQLTGDLTGVNYSSIRAGLIE
FRRRCAMLQHNIMVFQFCRPVWSRWLELAVLCGELRIDEKVAKAAKEEVK
WIPQGFDWVDPLKDQQAQQMAVRNGFKSRSEVVSELGYDVEEIDQEIAED
QKRADSFGLCFDSDINHKREGR

>WP0254_wPip_WO1_CAQ54362_HP
MYSEEYLTQVEKAIQKLQSGERVVSIAYGDHVVRYGEVQINDLLNLRQRI
KAELKVAGMRPKRKIVFSTSKGIL

>WP0255_wPip_WO1_CAQ54363_Phage
MIYARAFSEGLRPDPELKVSEWANEYRVLAPTAASEPGKWRTERTPYLKE
IMDSLSPSSPAEKVVFMKGAQIGGTEAGNNWIGYIIDQTPGPMLVVQPTV
EMGKRWSKGRFAPLIESTPCLKSKVKDPRSRDSGNTVQSKEFPGGIVVIT
GANSSVGLRSMPVKYLFLDEIDAYPGDSGGEGDPVLLSIARTNTFARRKI
FLVSTPTIHGISRIEKEFEATDKRYFFVPCPHCNYYQVLKWSQIKWENND
SRTAHYVCTECSGKIENHQKTEMLERGEWRATEAKEGEKKGFHLSSLYSP
VGWYSWQQAVEDFLHAKESEQLLKVWINTTLGETWVDKGEVPDWKQLFNR
REFFPVGTVPKGEVVLTAGVDVQKDRLEVEVVAWGKSRESWSIDYRVFEG
DTGGREVWGKLSELLNHHFIGENGLEYMISMMAVDAGYATQEVYNWVRGH
QGSGRVMAVKGVNKALVPLSSPSRVDITVGGQKLKRGIKLWPVGVSILKS
ELFQLLNVLKDGEEAPAGYCHFPEYAPEYFKQLTAEQLVSKVVKGYTKQE
WQKVRERNEVLDCRIYARAASIALGIDRWPESKWNSLSGKMESKKPKKVI
QSKWLGNQNVQ

>WP0256_wPip_WO1_CAQ54364_Ankyr
MSAHLSLELIKCLINQPGLDVNVRGLNGKTPLHCAIEFDELSMVDLLLTK
KNINPFVEDNDGKTSLDYAKEGKKAEILKALINNKYGSEQDSLLHLAAMV
GEVNAVRYLINRGIDVNSRNAIFHTPLHLAAGIGHVEVVKILVREGNAEI

EVFDARNQTPMHYAVNNKKLEIVKLLLELGADVNSARVGQNSMKLSPIHI
AVSNTNYDERDLCLDILKCLIREPNAQVNLQDYENKTPLHYAERLKTIEV
LLTREDIDPLVKDDSGKTPFDYAKPEIKKALISNKYGSEKNSLLHLAAQR
GEIELVESILKEEIDIDISNNKSLSPIYLAAEKGHLHVVKLLLKKGANYT
PVLHLAIKSNNLELLKVLFNEKNGALLCRDTVVNFPTLHNKYIAQREIAD
KRMKKHNNIICICITVSAVAMAAYIGLTAATISSAIIFATITGIFALVVA
IMVSEMNKRYIEKEFQKKMFMELEECSSTVNDVAIVSRCR

>WP0257_wPip_WO1_CAQ54365_HP
MKLITQTEWAREQGFSKQYVCYLVKKGIVELENGLINREQANEAVAAIRD
PSQPLRRKNYSENGEKLSTMLLKTRIKNEMERGKLLEAKAKAEIGELVAV
EEVKNEAFNVARVVRNNLLNIPNRVSALLASLSDTEKIHMELTEEITNSL
EELSNTKF

>WP0258_wPip_WO1_CAQ54366_Phage
MNLAIHYYPTRDLVEYERNPRKNDDVVNRMCASIREFGFRIPIVAKSDGT
VVDGHLRLKAARKLGMESIPVVLSDNLNEPQTKAFRLLANQSANWAKWDD
ELLKVEIQELEDLQFDLKMTGFELEKVQHFLDDLDSEKEDLSDLVVDDKK
VEITKPGDLWILGDHRIYCGDSSVVESYKALLDDKMADITVCDPPYNVDY
GSSQEREDKKILNDNQGEKYELFLYDICSHILAYTKGAIYICISSSEFST
LQKAFEEAGGKWSTFIIWAKNHFTLGRSDYQRQYEAMLYGWKSGNKREWH
GGRNQSDLWFYDKPTHNTLHPTMKPVELMERAIVNSSRPGDIVLDPFSGS
GSTLIACERTGRICRTIELDSKFVDVTIKRWQVYTGREAILSGTGKTFPE
IQEEKKEQKK

>WP0259_wPip_WO1_CAQ54367_HP
MSTLTLDLGKQTGWAILTDGVIESGSESFHTSRFSGGGMCFLNFRNWLNS
LKEISVVYFEEVRRHLGTDAAHCYGGFLAVLSAWCEEHHVPYKGVNVKTI
KRFIAGNGNASKSEVIEAVKEKGFLPRDDNESDALALIFYVMNFSKDFNT
LKIP

>WP0260_wPip_WO1_CAQ54368_HP
MTDTLTLKEGDIETTAFDNSNFTRVLHDGEWWYVITEVIAFLTGSKNPSD
YLKKIKSRDIGLSEGWGQFVTPLEIKTKGGKQNVNCTNVEGLFRILQSIP
SKRVEQFKRWLAKVGYERLQEYENPELALKRIYADYAAKGYPQEWIQKRI
ESIAVRNQLTKEWGNRNISDHNNKYIEGREYAILTDVISEGTFGVKTKHH
KEIKGLRKQPLRDHMTPIELIFNMLGEQATIDEIKDKDAQGYNKNLEAAK
EGGKNAGTAREAFEKARGVKVVSSDNFLKRIKD

>WP0261_wPip_WO1_CAQ54369_Putat
MSPLFIGRKTELKQLLELTEKNTASFVVVKGRRRIGKSRLIQEFGKYFEQ
YYSFIGLPPEKHTTMSYQLNEFSRQVARQFNTSFARYDDWSDLLWAVGER
LLSGKTLLLFDEISWMGSKDPTFLGKIKNFWDTQLKNNNKLIFVVCGSAS
SWIEKNILSSTGFVGRISLTLTLGELSLSDCNEFWPKNISAYEKFKVLAV
TGGIPKYLEEVNFKHSAEENIKRLCFTKGGFLVEEFNQIFSDLFMRKTAF
YKQIVRALSTGAKEQEEICATLNIVRHGRISEYLYELELAGFIAKDHTWS
IKTGTDSRLRRYRLQDNYLRFYLKYIEKDLGKISRDTYSIGFLPEWYTII
GLQFENLVLNNRKSIHNILGIDRIISENPFFQKRTRNSAGCQIDYMIQTK
FNTLYICEIKFSKDKIGHSIIQELQKKIDALNRPKGFSCRPVLIHVNDVS
DDVIDSGYFSHIIDFGKLLNCK

>WP0264_wPip_WO1_CAQ54372_RNA
MKSRNSYSGIDPKVVNLIKFYAKYYKSLTDQDIEDIEQDLFCEFLSCIDQ
YDKTKGSLSTFAKQVVKNRINNLVRKHSRTTPKLENNVQQESYCFEDETI
LRIDVEQIISKLPKKWQVLCEQLKHHSIAEVADMNNMSRTTLYNILKKMR
SKFAPVYGKKHKKN

>WP0265_wPip_WO1_CAQ54373_HP
MKIINSKERAKMVTGVKMVIFGPYGIGKTSLLKTLDESTTLCLDFEAGLL
AVQDWKGDSTEIRTWNEARDIACLIGGPNPALRSDQAYSQKHYEHVCSKH
KDLLSEVSKYRSIFIDSITVASRLCFSWARMQPEAFSDRSGREDKRAAYG
LLAQEMMAWLNQFQHIRDKDIIIVGTLGQYLDDCNRSTWLPQCEGAKTAS
EIPGIVDEVISMVGIKKDDGTEKRSFVCQTINTWGYPAKDRSGCLNMVEE
PHLGKLLTKIKAKAFATAA

>WP0266_wPip_WO1_CAQ54374_HP
MLQDFFTITQKIPFFSVKEYLDDQSPIPEDIIAPRILTKRGLLVLGGPPK
IGKSDFLISWLVYMAAGVSFLGMTPSKPMKIFYLQTEIEYEYMKERLQQL

QLDNELLNIAANNLIITPRVQLSFSSEEIDEIKNVIKERFKPDIIAIDPL
RNIFNSSEYGNENDNSAMLFFLQKTLERLRNVINPDSGIILTHHTKKLSK
KMLEEDPFQGLSGAGSLRGFYSTGMVMFAHDEESTVRQIVFELRNGERVA
SKLVDKIDSRWKLADQWS

>WP0267_wPip_WO1_CAQ54375_HP
MLSDFLTDFNNAKLQSNLIPKGTIVKVKMAIKPGGYENWFTKSYTTGSIY
LNAEFTVTEGPYAKRKIFQVIGIKSGKASVEGEDTWGESGRSMLRSILES
ARNIHAHDTSEKAVIARKINSIADFNGLEFTAKVGIEADRYGEKNKIATV
ITPENTELDWIPF

>WP0270_wPip_WO1_CAQ54378_HP
MKRAIIITLLIQTVGFIWWLSKLDSRVQFHDKLIEYGLMEKVLVLEERVK
NLSEELDDFKASNLKCE

>WP0271_wPip_WO1_CAQ54379_Putat
MILLTCSLLSGCVTCPKTTVCFTPRENCAEQITNAIDQAQKSILIQAYTF
TSKPIAQSLIRAKKRGVDIKVILDESQISSKYSVINELFYQKIPVYIDYK
PAISHSKIMIIDNQKIITGSFNFSDAAQKKNAENLLVITGSPSLVEQYVE
NWKDRQLQSRPYTPLIEN

>WP0272_wPip_WO1_CAQ54380_patat
MAKYILSVDGGGIRGIIPAIILAEIEKRTRRTIAEIFDLVAGTSTGGIVV
AGLCRKDERGNPQYSANDLVEFYQKYGAYIFKSSFLRRSIFSWLNCAQYP
HKNIEFVLDKYFGDSTLADATNNLMLTSYDIKNNYPFFFKNWREDRNFIK
LKDALRATTAAPTYFAPKYLKINHKEMVLVDGGVFANNPAACAYASAKRL
FPNDEIILLSIGTGRTDRSIANSRRLGKIGWIKPLLHVMFASSLDSVNYQ
LDQVIGDKYIRIQSQLKLASADMDNITSKNIKSLQQEAKAMIEDNQKVIE
KFCNIIS

>WP0273_wPip_WO1_CAQ54381_Putat
MFVSVRDISSISYKIGQKIEDCRLMQRCTQAELASKIGLAYKEVTNYENG
YIPITIEVLYVIARVLSVNVVDLLPKPITVREYEDEDEEILYLTKIYENQ
KLGKIVPSLIRFVHISEKINQEEARLEVAKNLVKEGVSVDIISQATGLSI
YEYDNTEKEVCTDSIYYRIGQRIREWRLIRRYTQKDLADKVGLTLKEIHE
YERGYTAITFDKLYEMAGALSVNIKVLLPETRESKEENRLLSLIDEYREQ
ESLVKSLSEDMKSGKEKVKKQRKSRLQKI

>WP0274_wPip_WO1_CAQ54382_Putat
MVVFVEKSLDCEVGEKVKNWRLERGYTQKDLAEKIGVKYWVILQYEKGNR
RISIERLYAITEALSISITDLIPISKSCLEDEGEEILNLVREYKKINDQE
LRKMFCLLTNFVQISEKSSKKAEKIKIAKGLVKAGVSVDIVSQAIGLSAD
ECIEEKVGSIYCQIGKKIKEWRLVREYTQKDLAEKMNTTRDEISNYEQGR
TAVPLDKLYEMAEALSINITDLLIEKDEGSTVENELPDLIKEYKEIESQE
LRNALIKSLFEGMHICEEKVRKAERIKVAKDLVKGGISIDIILQIVGLSA
DQIA

>WP0275_wPip_WO1_CAQ54383_CHP
MVNKIIVPFDDKKGGTYELNIDLDRLSKGEVLNAIIGISRVNQQSTFVSK
INKAKKSQEIAPFPGREVRENKINYGKGLDYIYGTKPASDQEDKNPFTSA
LQKIKPSASESKELSWFKSAVVEAKNVNELHKVIDKVITSGVRLNACNDG
EWSFAEYLVLGTHFHKFDKADRKKLIRKLMLSGAEFHDTLLQNKLIGEIY
NELQPEVQPQIDKQLEELEEAGESAVQEGSLIDFEIDNTTSYIEFSDDSK
VEVAKILRELGSNILKIGNDAVEVKSEKGGVRNYTDMSNGSSVMLEFPTS
IGKLNVILYHDVKKYDQVQVRVENKEMWAELQKRGEEIGKNCLFGGVKLK
EAVERGSFTRCGIWSEKYAIKEISNDEVLSSWVERVCGGSKETFREL

>WP0276_wPip_WO1_CAQ54384_Putat
MNKSKEEIEFRILESKGKALLDREIMETFLSAVHERPQAQEIAKNLVNSY
TGVGRILGREMDDLKVIEGVTDSAVAMIMCVKETLERVLREKLKSEPIMD
LQGLVEYLNVSIGHSERECVKILYLNKRRQLIGEESYIGEMEKAPVYIKE
ITRKALIKNATLVIMSHNHPGGSLEPSEEDQEVTKSLAAACSTVSVRLFD
HIIITSGGYFSFRENGLL

>WP0277_wPip_WO1_CAQ54385_Putat
MKKENKCSNFLDYKVIGQEVRNRRLAKGYTQKDLAKKIDTTYQVILQYEK
GTRRISIKKLYELAEALSTTARDLACVQGVSNEKRYEEEEVLNLVRRHKE
IKDQELRETFYLLTKFIRISEEESGKAVKIEVAKGLVKEGVSISQTTSLS

IDEYDNDEKKISIPYKVGQRIKEWRLRRGYTQEDLASKVGIINQRIYEYE
QGRSAVSLEMLDEIAKVLLINITDLLPETRENENSEVELSRLIKEYKKIK
SQELRNVLIKSLFESIQICKEKVKKVEKMKIAKNLVKEGISINIILKTVG
ISLDEI

>WP0278_wPip_WO1_CAQ54386_DNA_m
MAIILLDTKTINRIAAGEVIERPASVVKELVENAIDAGSSEIEIKIESGG
RNLITVTDNGNGIEKEDLELAFMRHATSKLSDGELIEIRHLGFRGEALPS
IAAVSRMKLSSKASGAKEAWSIRYEGGEKVREITPCSLLQGTYIEVRDLF
FATPNRLKFLKTERAETQSIVDIVNNLAMINYSIGFTLTSGNKKLLKYVK
QTSLFNRLCEIEKEFQSNSLEVKEEEEGIKLKGHICKPNVNRGNSTQIYT
FVNGRPMKDNLLVGAIRYAYHDFIPNNRYPFAVLHLEVPYDQVDVNVHPN
KSEVRFQNKRLIYEIVRRGIIKTLSTRFAAGDQGIEEELIFNDSKSQEQI
DSQEKKDQKEFYEKRPSLLENRLMKEFNAPDERRQSLPETFKYGESPPQK
GTMVLERKQIDLIADHPLGYARCQVYNTYIIAEAKGKLIIVDQHAAHERL
IYECLTSIKRQKFLLPETVEIKNQAGMEMVGMYKDRLFEMGFGIEIESED
KVRVKEIPAILGTINVKEMVMNIVDRLMEIGDTLPIEEKVNKILATIACH
GSIRAGRAMKLEEMNELMRQMEETPYAGQCNHGRPTYIEMKLSDIEKLFE
RR

>WP0279_wPip_WO1_CAQ54387_ribon
MDKKKVIIYTDGACSGNPGPGGWAAVVMYENKSVFIKKRISGGEENTTNN
KMELKAVINGLKMLKISCKVIVHTDSQYIKQGITEWINKWKTNGWKTADK
KPVKNRELWQELDEVALQHDINWKWVRAHNGNMYNEEADRLARKESKNLK
YRDCEVKKSPKNRGNSKFHRLGGVLWQ

>WP0282_wPip_WO1_CAQ54390_CHP
MPTQKELRDTMSKKLQEAIKHPDPAVVAGRKSAIKRWVGVLQDNFMEHIK
YFKGDKLKFLHNVFQDEGCWSGVRLDNAALGQRFTEEKIGGIDNPLRKYE
MACSYCVVDKIHPLFQKRFESYRNKFPPGAFDGKTETEFGKYVRNSLLDS
IKRKGPVFDFWIDRESGELKKYDAVEGFDSAVKFKWSEGVEYFYNHLKEE
DKEKKLTEAILALSRVQSVEKDAPILDFCVNKIVDKDTLLQKLSQKDKGV
YSLFAELIESCFFDTVHDLVQCWCYKEVSAGGDHSEKIFSQRDYELFLSS
LSDTMLKNPELSVQARSLIMEFWECGSLYQYRKAAVNTSNYTVPTSGVFA
ELIVNWRREDIYKTDEEKEIEKKEILDMMSFAKDCFPEKFELFKKLIIRD
LRLCGREGKRVNVDYGLFAEELFSELEKTILPPGPVGDGPCSNLRSRSKA
HGSKKTTLPVDDSPQSELGTPSVSGVSSYKKKSVFTLSGNK

>WP0283_wPip_WO1_CAQ54391_CHP
MSNGDGLIRSLVDGDLEGFRQGFESFLDQCPSFLYHVSAGRFLPVFFFSM
FSTAHDANILNANERVYFRFDNHGVNPRNGENRNTANLKVAVYRDGQQVV
RCYSISDRPNSDGLRFSTRERNALVQEIRRQNPNLREEDLNFEQYKVCMH
GKGKSQGEAIATVFEVIREKDRQGRDKFAKYSASEVHFLRQLFRNHRLTI
KEIEGRQLNQNQLRQLGRSVNFTRVEPGQQRIDNFMEMLASNQRQDVRDS
LRGDILEYVTDTYNNYRAQIENNIEGRSQKFESHGFLLGFLANFSHRYTI
GVDLDLSPRNSHVAFLVRHQVERENIPIVINLATRAPPYIALNRARSHAE
RLHVFSFIPIHTESRNTVCVGLNFNLNLDPFSVDTVGLQQDRFPLVQRLF
ECLENEGIRENIRDFLLHHLPAEIPRNAENYDRIFDCITGFAFGNSAFDR
HPLELEEEDEAPITKYIFRHGDEGLRCLTMVFHAEGSDIVILHIRAHDAQ
QQGAINLQTLNVNGNDVHVWEVSCTLNNQLELDIDLPNDLGLYHDYQNNN
ANNFLAGDLVQVPNTENVHNTLNQVVNDGWKNIAQHRGLFQEISGALMPL
VDTINVNSEDKFRSILHGTFYASDNPYKVLAMYKVGQTYSLKRGQEEEGE
RVILTRITEQRLDLLLLRQPRENDLDTHPIGYVLRLANNAEEVGQQQNDA
RQEIGRLKKQHRGFIPITSGNEVVLFPIVFNRDAHEAGNLILFPEGIGRE
EHVHRLDRHVRSSRPGGLVGPESVIDENPPEGLLSDQTRENFRRFYEEKA
PGQNSIFLLDIGDNLHVPFSYLQGTRAQVIETLKSRIRGGGTPTAQGILQ
QINAILRRNNAREIEDVHDLLALDFATDNQNYRYWLQTHDMFFAARQYTF
LDNQSHSTNDHYGFEITSVGVDGNQNDPTGRGLLSSHITNFKQKVDSGEK
DRLIAIINVGNRHWVTLVIVHQNGNYYGYYADSLGPDSGIDNNIRGALRE
CDINDDNVHNISVHQQTDGHNCGIWVYENARDINQAIDQALQGNNNFGEK
GEGIIGYIRGLLSAGIGNDTRQPRRNEQYFEDRRRDISQLLQNDPNLPSR
RSDLIQAHPGIQHEIDPLLLQFLGLQYPQRGGGGALQLGGERVISIDFGN
PQSALDKIDGVSRVYNHSNSRGSR

>WP0290_wPip_WO1_CAQ54398_Putat
MANISIRYQIAQKVRSWRLKRKYTLKVLIDKTSINYHTLLRYEQGTCGIP
IEKLKILAGALSIPIRNLFPKRKVLRENSCFDKAKEQAMYNLMQGYIQIG
ERELRKVVYALTQSIQAEKESNIKAARIRIARNLVKAGFDTEIIYRATGL

STEEYADKERYEPNGGQEIKKWRIIRGYTQSKKA

>WP0291_wPip_WO1_CAQ54399_CHP
MAFSKFLDARNDYAFKRIFGTEKNKDILIHFLNDILGFTGLAAIHDVEFL
ATILDPEIAAKKQSIVDVLCKDSQGSRYIIEMQFTKTKGFEKRAQYYAAK
AYSSQADQGDNYHNLKEIIFIAVADCIIFPDKADYKSNHVILDQNSFEHD
LKDFYFVFIELPKFTKTKEDQLENIVEKWCYFFRYAAETREEDLDKIVGS
DVIIKRAYEEMNKFNWSEEELLAYEQMKKRIMDEIAAFAQKFDEGLRVGQ
EKGRQEGIQIGHEKGKIEGKIEGKIEGKIEVAKNSLKAGVSIDVIAEITG
LSLDEIKKLI

>WP0292_wPip_WO1_CAQ54400_Ankyr
MIWQKSYGTLSKLRKSVDTNAQAIQEYKPEKITDITDKVVIISAEPGMGK
STVLTPLALKTKESLWIVRINLLDYSTELKIETEKKTKLNEAEAIKFLYR
IVGFQLFQGEKEETTEKKGKREQTVENVLHAITVKDSKISLDRKSTQDLD
LLEIELFNNFYNQGRIVLLFDGFDEISPDYTEKVIELLQVLKNSKVEKLW
ITTRPYNFIQEELEDQLSVFSYSLKPLLLEEQKGFLRKFWKEKLKLNELD
EQRSGVFIDELLGKLPKSIGDKDFMSIPLHASMVAEVFTDAFKSFYDSNK
QELSDEHKRKIEEQDLVTLYERFIDIKFYKIRFGEKKPGMNIDDPDMKGM
VEEGREKLIEDHMKLALYAIFNEDEVKKLLTGEEMDKVRGLIDDIKEGKE
STGIIEQVIDNKPRFIHRTFAEYFAASYFWERFKSVKSDKFEDFVENIIT
KNLIEDDRIQISKFLQLKAKKDFESNIDFPDKQNKLRTLLTKLLDQTTRY
DYNGNGKQSTKLLFGIVEFSLSTKEKSNVDVIENNDQLKLLCVSAEMGYI
KLVEALETKLNKEFLQKYLKRDKWFYSPLWLAAQNAHLDVLEVLVKKFSY
DPNWKDKDSETLVQRIFQKIEFDVLRSCLKLDIVDGKPYEDLNHRQELPI
LEAFQYVAPSDVIELLIEKTDSNLVNGFKYPEISSQISPAQLIAVQSLKY
GKEITELAIKKGIDFSDVINVLLFEYCYESLNSPINKLNYASNSQPSFHS
DKEFLNLFERIELLLKAGVGYNHKDIKYSKTTLQYAKQIHYIHELLDGVH
KLHNQKINKSSNPLGTILKLIIDNVGSSPDFEGYEFVESHQQGISLSLNY
KDESSKYNKRIPLLPDNIPKLLFKFYEDSNTNLVENALGKFRDYITNAKE
EIGAFKQYPDSKVYNEILTGYREMLKFNDYQHLYHQFYSLDERKEKENRY
QSLDDYEQDLLERVLNLYIRQDYEINDALKEVEEEVLRLKEIINKLKENS
GHKKYETGEILDILAIKSMDTNEELQDLISKIYECGGIQFLSETWLHLNL
IGQQKIMGTLELMFKFADQSSKILELLKKLDDSYKTLTEAIKNKDYQMIN
NILQDQDIEPDFIKAIINGQDDKYGSPLHYAAFKGDVQVTKILLESGANP
NLKMQGSGILLSTADETQKDEDLEYKYQGCTPLHVAALNKQLEVAKILVN
DWDADVNAMSTGWQVTPLHMAAESGNLDMVKFLVKEGKADVNAMSTGWQV
TPLHMAAESGNLDMVKFLVEEGKADVNAKNKDERTPLYLSARKVKLDVID
FLVEKGGDLNVKDKYGKTPLQSIDYENYSFDDFVFTVPGALNNNDEDKRN
VLILQWAAYFGNLDVVKSLVEKGADVNAKDELSRSLIYYAAYSGNLNVIE
FLVEEGADVNAKEEGGRAPLHTAVQLGYLKIVKLLLEKGAHYDVQNAQGK
TPLDLAKSGSVKNLLSDIDKLFQDVQDGKEDKVNSLLKGKDDDTLKAVLH
ARNRNQKTLLKFAIANKQQDIANLLIEKLRLIQSSDQQQEAVKQGGEVSA
KECLPSTSSGRSKREAIGEKCLFTWEDVDEFNEEKDEKRDLSKIKIDSER
FVSYIKDLPKEKQSQLIQLASEVKIGDSSQGLVSKLIGNQKVMNHLGRVA
GMTMHGMMAKNVLADFLNGNYQGAAINVGFIAGGQGFAKVAEAASLKGLK
LAEEGKLLVGQSLKAASPFLARGTSAFVVYDLVNQIKAFKNGTEEALVGV
VGDSIYLGVDAAEIGVEVAEAFGVLEGVSSVTGPIGATIGAVVFVGTDIY
MAVKRVDKIDQIIHLTGDEKFVEGLRAFIGMKPEQYIEELMEKKQLYNQL
VKQGLGYLKQHSDIQSYVFPTTDKLDSKVLLDRKRTGIRWSRARPDDLNE
GRVFCLPQGNDEPAPDYGSYLCENAIGIFANKTGDHTLINLGEGKDYAKG
FLNSPNIFVVNNGSKEYYGGNKDDIFVLQAEYIKGHLSGEGGINTLDTTL
FALQEEQLNIQLEIGEIADYFRDNWLKICEINKVIGRANKAETITVSCDG
CNSNVRLIDGQSGNEEIKDRINIIDDSCDYQMQIVVRPNTAVYNRALKGS
FDYLVPLNSSGSAEVSFIYGPERFNVNNTFWFAYQAVDIKSIDVKYINVF
NRTEHEVKFNFVESNKEFNVTISYSENPAYRLGKSGEIRIGNKGNLYMLE
SSDKSSEEVIRDYLPVANRVSKMSFFIQSVLSNETVVIGSGNHEVIHSNP
AYRSHLVGNGGENVYVIDSETTEVIIHDVDEENSIDTIDLRNVVRQVRGE
LSNQDNFQLKVLKSANDLLLEATVVEVKPTEDSSVSKMRKHEYCTVRLKD
GVNWYNKTHVIVDNAPMKINLDNNEWSLKPQPLMFERDKEIIVVTNQDIE
QGSEIITPRKGGNYKFVRSNDNDLMITNAFDLTITKNDLCTITLSKFYET
PRMKTLFIKFADKEIILKEHQEEISTARDVNVVKKEHKDQVYNDVFNPEV
MMLSDQPVTHRHRHSRHREQARHRRSTTSSSTRPTGWINDLFGWVKSSIS
GLLSSKPESTKSPISQVDAKMDVNGTIILLDMLIRKVTGQKYVSTADQSI
SPLEAQGYALNITKGFEKVVEQAGLKSGVSMHRLNIDYMGMQKEITRKVM
SGKFNEISGILSSYVEKACPDGEAGYPGKLSPKKFDKFMIEFNKGLDVTL
NQSIEHNGDGRLEVDVVKEQKTNSGPQSYLSNASVQGHLTQNKVKLIS

>WP0293_wPip_WO1_CAQ54401_Ankyr
MLKVEHSSDYLKDEGRDSQAVELLKKKQQSKRSLQPKSRLRRQYNPDDIN
IAKLFLAIREHKTGNPQLDKINEIKVLLIQIRNINDIDFNDNGNTPLHVA
VSKEHEDIVKLLLKKGAKVDIKNNIGSSPLDIAVRLDNQIIIGELQKYSE
LGSSNQQVSSVQQQPKNDDTLKELQTREPISIFQKITKEKDQSKKQNLEG
LLNKLRSSYPDDLKIGVALKTGDCFFDSVAQGLNELKDKGLVENSERFNV
KSLRKDCADYARENNNKDSWTYEAVIKDAAIGGYFAALKGPYGPIQDPKT
KINVIDKGETFRKYLKDIKNMATGTDYPAIWGRPEIEGQMISNKYGVVLC
FYSTNEENGQKITVTKITPDNNDPEVFIEGVNFDQLHKDSNEKVLRIVNY
KEHFVPLLGGLTADITENVKVPREEVYGVENKKSFDLEEDSDLHNNQAQP
FVDTFTTEGFGNCFFHAVFGDNSSGTYKAERAQDMRMEWHKFLSQFTSLS
DPKMPNPLRDQLEKVFDGFLNTPGDLTVKSEEIKGLAEQTNKKIEEADSK
IKELQEEIIKKFRDDGDFRKVIYPVIEKAITQRNERNVDSPRDQKTLLSI
EDLLKEENKERLYNDIIEDLESCALILNSSLSKKEYGNTYNPKIIKDSFL
NDEVLYETYLEAISDNAYYVFFEEVPILASLANIKITAYYDNGNRLQVFE
PNKELLGSYQSDENLWGEKEEAVIFHKGLHFSHATISHSLHSDPKQRKSS
EHGQPLSGSHQLSSSRQQESEISSQINDRGKQTPSGKSRPDHQSFSSEIK
EPGKRSKTPIPVSQSSGLANQNAASQVDRKKSSNVLGEADMRSSRIPHLK
SQAGSSQQSSNNRQSVRGTIDSAQHQQINPNSPNFIDSDDEREKNTELFS
AIKNSNLQKVQELLKAGVKVNIIDKNNKDNTPLHYAIEREKKEIAKKLLQ
KWKADINAKNNKGDTPLHVAVSKGHQDIVELLLKEGAKIDIENNAGKSPL
ILANELDNSNPNKQGIIQILETLLSSKTQIQPRQRTNIDAQGKQENRDDQ
RPGPSRQVNNPTQLHKQSSVKQHPEQSPSDNSNFYQKRSGTSGISGQLYE
TKLLMLILFRALNDEKINEFYLGTNVDGVGALDDIIFRYNLSGQEKPRVI
FIQAKHKDNSDNKKVTVGEVQKKGGDFSLHSYLDSYLRIKYKFNPESDDV
IFKGEFQNIDCDFVIYTPAKENFSEKKSIEQPNTGDVLIYTSEEEGKRFQ
FEYKDENIGLLVKIVSESHTKILAENFAKFILSSTDNMMSDGLIKSYHVV
LAQKVIRKVSDGKWKFRDEFFESKEDLLRILKKVLCEKIVYDKKSTTKPE
EIRVLVEAMLENTSPDTISKLIGSVVTFDSEESRLKFVEKGIPQEVIDLK
NKLEEKEVTQKIVNDAIWIAGKKKLQSLSLKLPSTFGNLDLALRKEKRLE
HLTLKFIDLLKDGKVTNNYVVTIDDNMVGNQKRLNKGDIAINGGIGGLVG
NLLIFDEETSMLKFNNNEESLPKNAKQLFQKLKDKIEKENGLTNKKLDSY
RFEINVKNFPRLSLIDEKHDKSLVREFLDKIKFFTNQAKEDGVEEILKQE
ISEYYLQNRESLFDIKTDAIFLDVHDKVQKWWKLPKEAPYLTKSCSYFNE
VKDRIINNPILNILRIIYFHKIKGSDIEFSSDSIKELKLEDFVSDPNKQI
LNIIAKEKLLSAVKLVQNFVTADFIDLDYLYSLGGDNDYLEKAITELKQK
GGENNILVIHCNDIEVERVDELYKRVDELYNRLSSIIQSNSNKKVILITQ
EQDPLAAKFKEDSSTKYTEKKDERNILTDLTTESQEKLLEREKVIFQGKE
VSLGTLVDDGSKHLLDGEVLSKLINKEKVKIGKALIDSKYEDIKDYYISR
TFNRQVKIKKEILKEKDSKFLVTYSSQVNRQNLKEDQDIVLISDTGEDFK
HLCDNHKNTIFIGSKRREMI

>WP0294_wPip_WO2_CAQ54402_CHP
MESGLDHNYNKILDILKGAIKGDDNQVKARKHLRVERWLRAYIQLIEDFD
EEKLIFFSDIFSDNSCWDGIKLKNKAVGERLTEEKNKNGKENPLDLADRY
YLACKYCLEDKIPGLFEQVFMRFKRSAFEEDGSDDDLRRELLENIEETSP
IEAFWSFLIDKQIGKLNEYKSVEGLQKSIQINSNKNWEEGIEFFYNKLHN
DSSISSQDKDDLLIEAALSAVKGYKEVDTIEFCLSKMDDEQKKKLLDRDY
KENTYYAVLNVLVGQYYFDSFMELSRLCSQIECERYTTFLSSLSDQVLKN
PDLSEETKKCMMNVWERIIKLKTQDRGEQSISSIFVDYSVTYTIANLIVD
PSRQGVSKEEILGKILKHVKEMSGEEMIKVKDSVLSKIQLFHGGKKLQLG
EQVFSKLAQEASKESILREAGDTLPQSSLSTTDTPYNIKSLSHSK

>WP0295_wPip_WO2_CAQ54403_CHP
MPSNVKPLELVQLLLMRNKSKDEFLDFQKRFQSFINQSPSFLHSVGKPGF
FPSFFFGMFATVLDTELATKIGIKKLHFRFDDNRTLKIAILTNEGLKCIT
MSDQVDGNMHLKFSQGELEKIAQKWKMGAEFDKLEKEEHEITITGKEVKH
GKVDPAFSKKTDYSQKGFTEIEKDRDQQDLESLISKLSNQDFEEVKKNAR
RMFNYITNVYKKYEKETLFSGKESSHHGFLAGFLINFKYRFHLKLYLELF
AGKGYADIILLVRGSDKSLSSIPIIIELKAGTGEISTVIKALKQAQDYVK
GSFSNSIRMITIANEAICVGLNFDMVHHENVKIDVENFLSREGNSVIEKL
LGTEATNAEVIRTQLEYLYYGIVWSNGGSDNINYVSRMILGQLVLISNII
KREKLGKHIFIYDQNDKMVTGSQKRPEAAKESIEDCVTTIVLTLGKKVLI
LNINEKNEFALRVPDNKGIPIENIRRIQNVNDIKIQEITCNLYSTPSNKN
PFDQYCNKNKGITVNTYDSLDKYKRGKEILQGNFTRIVENKKFKAALSKA
IESGKYDDYKKLFEEISHILHPFKSLISNEATFQAVLHGLFSSYGEDNIK
VITEFQIGGGEKLDVMLVINATDQKKEYPPVGIELKFAKKGELDKKEKDA
KDQLKRYKEGEAYKVITDAGKVKLIYAVFNKGATDEGSLIKIGNEFVEVD

VRHSSVVAFGQQPGSLQQPYVKQAGLSRAVNQ

>WP0296_wPip_WO2_CAQ54404_Ankyr
MSESTGMVKSIINLLKPKSKSELYDFEYESIIEHLSSKEHLLNELMNDMW
YSGDLPSVRNHNELPSLIDSKLGLSEQICVWFALDHDLTPSQKILNKKLL
SALKCLASNCGSFDNTEPLEEFLYDNKNNKDLKVILNLRRGESQSTVLHA
IAGANIGGFRRTGNQAVDLLLEAGADPNIQDSKGKTPLYIAAAKGHYNNA
NSLFLKGANPNITSRKGKTPQQIATNKLCYNIEELFLTDKQKKLNAELYD
LLVLDSDCTKNLKEFLSKHKRDSDLKVVLNIRQGMGESKVFSYVDRFAWD
NEGLAQELRKIFLEAGALDYDINIYRQQKKGQASKLLVNLTSNQKEKLNN
FSDKVFRAQNMAELEEIVNDAIKSGVRLNYSSSQDFFLGNEYTFTDYVMK
KISELEKNPKVASSIICQLVSKGAVFGNTVDANTLTSEFKEHKTNLKKAY
RDYISNSHKFIEIAKSATNSELKDARVDNSVFYLEYSKDSKIDIIKITDG
TRDLGLTDGDVKCGRNIVKIGKSEVEIKTEDGIRNYTDLTEGSDIVLTFY
TSLGNVDVRLYPDVQNKSKIIVEVSNREEILEKFKGREEELGNDCELGNY
WVYDAIEQGYFERSGGLMRPEVISESNNKWTKREELRRTSDPRREVSR

>WP0297_wPip_WO2_CAQ54405_site-
MLKEVRCAIYTRKSNEDGLEQKFNSLDAQRVACEKYIKSREGWVALAKRY
DDGGYSGKNLERPAIKELFEDVKAGEVDCVVVYTLDRLSRETKDSIEVTS
FFRRHRVNFIAVTQIFDNNTPMGKFVQTVLSGAAQLEREMIVERVKNKIA
TSKEQGLWMGGTLPLGYDVKDKELIINGKEAKTVKHIFERYMELKSMAEL
ARELNSQGYRTKARFDIFKKATVRRIITNPIYMGKIRHYEKEYEGKHEAI
IEEEKWQKAQELIRNQPYRKAKYEEALLRGIIKCKSCDVNMTLTYSKKEN
KRYRYYICNNHLRGKSCESVNRTVVAGKVEKEVMKRAEDLYEKCGEEWKN
LSFGKQKEVVKKLIKGVMVKEDGIEVSSEDKVEFIPIKKKGNKCTVVEPE
GKTNNALLKAVVRAHLWKRQLEEGKYANIKELSAKVNIGTRRIQQILRLN
YLAPKIKEDIVNGRQPRGLRLVDLREIPMLWGEQLEKFYGLGA

>WP0298_wPip_WO2_CAQ54406__Puta
MEKEIEKKVMNLEKKPLGEMRKIWKKVYGEEAPRHSKKYLIPRLAYRLQE
KVHGEISRKGAKRLEYLADRLEKGKRISSDKLPVAGTELILERGEETHAI
MVTDKGLIYREEFYTSLSAVAGKIMGMSYNGPLLFGMRDKSGN

>WP0299_wPip_WO2_CAQ54407_Ankyr
MIKLGKKEKSLLESSFKKKETKRELHYAVDAKTVRLLVEKGANVNAKDVE
GYTALHLAVTEKRLEIVRELIKSGGNVNAEEYGNKCTPLHLACMIGEKEI
VKELVKAGGEIEQADKFGMTAMDYAKNSKEITEILKKEIDRIEKLFMKG

>WP0300_wPip_WO2_CAQ54408_ankyr
MKFSKESFSKFCKEVSSKDINKRNEEGETILHQAVEISNYKTVRFLIKKG
AEVNARDKNGYTPLHCAVFAKSLENVKVLLREGAEVNATQYVTGCTPLHS
ACKIGGAGVEIIKELVKAGAEVNQLNKYGATPMYYIWESEKYCLCDSKES
EKASKFLREKGGITKSRELTCYGIEGLVGEIADMLNGSYMPELKIIEIGE
IRKRDKSLIKEECENLASKIMSQVNEMIDEVVKRKA

>WP0301_wPip_WO2_CAQ54409__Puta
MLLPPNATKQEQALVDAIDYKVDPSCIRRFKFSLKEETLPWIIEEYGLEE
ILRWVKDRRKAVIEGVKFQRLRGTPASLKIALKWANIEDIKIIEEPPGKH
FFELQVGIKEVPNDFFVDAVVELAKLSLPVRSRLMRIFNDYYNVQRFILD
ESLFGDLLSDYSGVKIEKDGPVLSFGRVNFFRSSGPVIRIIENYLRDHYE
RALSNDIYRLDVAVLGETEAHTKDYKGIYERSHQWYNLKALYPLSQSLLP
EIKFAKAQIVLSDSWKLGEINGCFPVSSVEEKGNKFVLGNDKLSGQRWNL
KHKPILERFSIIHRYKVENYTDQKVRKYVLAEHNIYYKNDLYSEQKDSIH
ELEKHILVFYPGVLKWHEHRHLHRSWKNSQVISIIS

>WP0302_wPip_WO2_CAQ54410_Basep
MKQLNIVEKLSYEEIFSRMKEELVKRDASFTGLVESDPAMKVLEVAAWRE
LLLRQRINEAVKGNLLKFATGEDLDSLAEFYGVERQNEEDDEHFRKRIKA
KIAGWSTGGSKEYYKYHALSADSRVKDALVESPIPGKVQISILSTQTGIL
SEELLEIVKKQVTRDDIRVLTDTVTVVGCNITEIDIHSRMSISPVISKEE
IKKQFIEKFELAKRLGWSVTRSWIIANLFVDGVENVELIEPKEDVVVLGN
ECANLRNLKIE

>WP0303_wPip_WO2_CAQ54411_Basep
MKGMDAKTGKTLEGIEHLKQSIIDILTTPINSRIMRRDYGSRLFELVDKS
INRDLTLEIYSAVAEALQRWEERFKLEKVKMTEVKEGKVTLDLEGLYLPS
GKNIHFDEIVV

>WP0304_wPip_WO2_CAQ54412__Puta
MNKGIVRLGDYCGEAIPHFCISGSNNVFVNGKSICRQGESFSEGKVMIQG
SKTVFANGLSVGRVGDIVSCGFKVIKGSESVFAK

>WP0305_wPip_WO2_CAQ54413_Basep
MLESNFAISELQRKLANIVRIGLVKEVDYEKAKVRIKIGEFLTDWLPWIT
SKAGKDRNWSPPGIDEQVVILSPLGELSLGVVLPGIYQQKYSAPENKKEV
SSLTFQDGTKLSYDKDKHHLEIEVVDKITLKAGESSIEMTKSGIKLKAER
IDLN

>WP0306_wPip_WO2_CAQ54414__Puta
MNLKDLHDKICTTLKREIPAIQTCEIYPSIRKELLAPALFVELVSLESGK
DPGTEELALKARFEARIVTDSTIENAPIIVRTLAAEVARVVNKNTWNVEN
VSPGEFISTEIDGFRPELDAYLLWLVEWSHQLHLGKSIWTENKIKPHTIT
IGENVRE

>WP0307_wPip_WO2_CAQ54415_Putat
MFHNVNQVISDIRGKEAQIKLVVAKALNKTAIWLKGQAAKEISEEKKIKL
TVMRKRLRIFKAKAGRLEVLIRANLYDVRASSIGSMRETKRGTKTGNHEF
IGAFTATMPRGYKGAFKREGRAALPIKEVKLPLEPEASRIIGNLVNYEVE
KVFTKFFERELSYKG

>WP0308_wPip_WO2_CAQ54416__Puta
MSIERLLEDCFEHLGVEATYCNKNKEGIHKIKVLMKRPETKYSLGTDGAL
TQQIASIEIRDQDVTSFSIGDYIKIEKRFYKIFEPPLKDSSSKIWKIQAV
GEGSVS

>WP0309_wPip_WO2_CAQ54417__Puta
MQNPFTNTAFSMTALTNAMNILPINYGRVENLNLFPNRSVRFRHITIEEH
NGVLSLLPTQVPGAPATVGKRGKRKVRTFTIPHIPHDDVVLPEEVQGIRA
FGSESELKALADVITDHLQLMRNKHAITLEHLRMGALKGIILDADGSELL
NLYNEFEITPKVVNFALGTATTDVKRKCLEVLRHIEDNLSGEYMTGIHAL
ISPEFFDALTSHTKVKEAYERWQEGAALRNDMRSGFTFCGITFEEYRGQA
TDPEGTVRRFIEKDTGHCFPLGTASTFTTYFAPADFNETVNTLGQPLYAK
QEPRRFDRGTDLHTQSNPLPMCHRPGVLIKITAS

>WP0310_wPip_WO2_CAQ54418__Puta
MISITEGNNLGDLLKYEVSNLYSRDQITVAKGQNLKLGEVVAKKTEDGFI
RVLNPVGTDGTQTAIGAIVSDVNATENSKAVIITRGAILADHAVVWPAGI
TEEQKAAAIKQLEGRGIIVRKGV

>WP0311_wPip_WO2_CAQ54419_Putat
MWINKPVMVERRSFELLSLYNSKQPIFKNLKHFHINPKGIAIIRIYGVLT
KKTEAFDHILDMTSYENIHEEIESALEDKSIETILLDIDSPGGEVNGVFD
LADFIYGVRGKKRIIAIANDDAYSAAYAIASSAEKVFVCRTSGVGSIGVI
ASHIDQSGFDEKQGIKYTTIFAGKRKNDLNPHEPMTSESLESLQKEVDRL
YEMFVQLIARNRGLSIEKIRSTEAGLYFGEKAVEIGLADGVTTFFEFINK
GENTMNKQTTTDLEIDDLETDNLTKYRTEIVELIRLCNLSRMPEKIGEFI
EQGVSIEQAREVLMELLAERTKKTEILSAIPQNSQEDLMTQVAKSRCI

>WP0312_wPip_WO2_CAQ54420_Putat
MLLKTFKQLFNKPKIKSSAWDTAGSGRRFFHFQPELGSINNLLSQSLETL
RSRSRDMVRKNPYAANIIDTIVSNSIGTGIKPQSKAKNAEFRKKVQELWL
KWTDEADSSGVSDFYGLQALVCRSMIEGGECFVRLRNRKLEDGFSVPLQL
QVLESEHLDNKSNQTLANGNIIRSGIEFNRLGQREAYYLFREHPGEGSFG
ESVRVPANDVLHIYKPLRPGQIRGEPWLSNILLKLYELDQYDDAELVRKK
TAAMFAGFITRLDPEANILGEGESNEQGVALSGLEPGTMQLLDPGEDIKF
SEPSDVGGSYEAFMRQQLRAIAIGTGITYEQLTGDLTGVNYSSIRAGLIE
FRRRCAMLQHNIMVFQFCRPVWSRWLELAVLCGELRIDEKVAKAAKEEVK
WIPQGFDWVDPLKDQQAQQMAVRNGFKSRSEVVSELGYDVEEIDQEIAED
QKRADSFGLCFDSDINHKREGR

>WP0313_wPip_WO2_CAQ54421__Puta
MYSEEYLTQVEKAIQKLQSGERVVSIAYGDHVVRYGEVQINDLLNLRQRI
KAELKVAGMRPKRKIVFSTSKGIL

>WP0314_wPip_WO2_CAQ54422_Phage

MIYARAFSEGLRPDPELKVSEWANEYRVLAPTAASEPGKWRTERTPYLKE
IMDSLSPSSPAEKVVFMKGAQIGGTEAGNNWIGYIIDQTPGPMLVVQPTV
EMGKRWSKGRFAPLIESTPCLKSKVKDPRSRDSGNTVQSKEFPGGIVVIT
GANSSVGLRSMPVKYLFLDEIDAYPGDSGGEGDPVLLSIARTNTFARRKI
FLVSTPTIHGISRIEKEFEATDKRYFFVPCPHCNYYQVLKWSQIKWENND
SRTAHYVCTECSGKIENHQKTEMLERGEWRATEAKEGEKKGFHLSSLYSP
VGWYSWQQAVEDFLHAKESEQLLKVWINTTLGETWVDKGEVPDWKQLFNR
REFFPVGTVPKGEVVLTAGVDVQKDRLEVEVVAWGKSRESWSIDYRVFEG
DTGGREVWGKLSELLNHHFIGENGLEYMISMMAVDAGYATQEVYNWVRGH
QGSGRVMAVKGVNKALVPLSSPSRVDITVGGQKLKRGIKLWPVGVSILKS
ELFQLLNVLKDGEEAPAGYCHFPEYAPEYFKQLTAEQLVSKVVKGYTKQE
WQKVRERNEVLDCRIYARAASIALGIDRWPESKWNSLSGKMESKKPKKVI
QSKWLGNQNVQ

>WP0315_wPip_WO2_CAQ54423_Ankyr
MSAHLSLELIKCLINQPGLDVNVRGLNGKTPLHCAIEFDELSMVDLLLTK
KNINPFVEDNDGKTSLDYAKEGKKAEILKALINNKYGSEQDSLLHLAAMV
GEVNAVRYLINRGIDVNSRNAIFHTPLHLAAGIGHVEVVKILVREGNAEI
EVFDARNQTPMHYAVNNKKLEIVKLLLELGADVNSARVGQNSMKLSPIHI
AVSNTNYDERDLCLDILKCLIREPNAQVNLQDYENKTPLHYAERLKTIEV
LLTREDIDPLVKDDSGKTPFDYAKPEIKKALISNKYGSEKNSLLHLAAQR
GEIELVESILKEEIDIDISNNKSLSPIYLAAEKGHLHVVKLLLKKGANYT
PVLHLAIKSNNLELLKVLFNEKNGALLCRDTVVNFPTLHNKYIAQREIAD
KRMKKHNNIICICITVSAVAMAAYIGLTAATISSAIIFATITGIFALVVA
IMVSEMNKRYIEKEFQKKMFMELEECSSTVNDVAIVSRCR

>WP0316_wPip_WO2_CAQ54424__Puta
MKLITQTEWAREQGFSKQYVCYLVKKGIVELENGLINREQANEAVAAIRD
PSQPLRRKNYSENGEKLSTMLLKTRIKNEMERGKLLEAKAKAEIGELVAV
EEVKNEAFNVARVVRNNLLNIPNRVSALLASLSDTEKIHMELTEEITNSL
EELSNTKF

>WP0317_wPip_WO2_CAQ54425_Phage
MNLAIHYYPTRDLVEYERNPRKNDDVVNRMCASIREFGFRIPIVAKSDGT
VVDGHLRLKAARKLGMESIPVVLSDNLNEPQTKAFRLLANQSANWAKWDD
ELLKVEIQELEDLQFDLKMTGFELEKVQHFLDDLDSEKEDLSDLVVDDKK
VEITKPGDLWILGDHRIYCGDSSVVESYKALLDDKMADITVCDPPYNVDY
GSSQEREDKKILNDNQGEKYELFLYDICSHILAYTKGAIYICISSSEFST
LQKAFEEAGGKWSTFIIWAKNHFTLGRSDYQRQYEAMLYGWKSGNKREWH
GGRNQSDLWFYDKPTHNTLHPTMKPVELMERAIVNSSRPGDIVLDPFSGS
GSTLIACERTGRICRTIELDSKFVDVTIKRWQVYTGREAILSGTGKTFPE
IQEEKKEQKK

>WP0318_wPip__WO2_CAQ54426__Put
MSTLTLDLGKQTGWAILTDGVIESGSESFHTSRFSGGGMCFLNFRNWLNS
LKEISVVYFEEVRRHLGTDAAHCYGGFLAVLSAWCEEHHVPYKGVNVKTI
KRFIAGNGNASKSEVIEAVKEKGFLPRDDNESDALALIFYVMNFSKDFNT
LKIP

>WP0319_wPip_WO2_CAQ54427_helic
MSIYGRTWWGKKWLQSFSEVDYDNRLSRGRTYANTGRAFGIQINGNTITA
KVSGSRPHPYKVKITLNELNSSAIRCIIENSPSILPKLINRQLPTLLFDK
LNDSGIKLFPSNWEEMNASCNCPDWAMPCKHIAAVIYLIAAEIDKNPFVI
FNIHNCDLLSLIDDFGNGKLENIQSILKINDLFKSSSKVKVYDQVNLNDI
DLSTIPNLASCIENILTNNPLFFEKNFHSILQTAYKHWQKYPTGKSDYFF
YSSKKKLSEEELFTSKWGSIEHWQTFQLFINDQYQVIQVSNGIANSFMLS
ESVSRNIAQFLNDIPISLLHKLNPELRFMHMISQFARMLMEKSALVPQIL
QNKRGEFIIRWIPALFNESVKEIHRKISSMCPPLLIQYQKTSIEPEEQVN
IAISLILLGYMANNFPAALDKYREKNTFPSSMDKYRDKHIFALFFTGSAY
KFSEFSNREIPQAINLWLSRLYLTDKPYKLYLMVKDYYERFELDIQVSLD
DGKSIIKLKKALSNCSTKLSILSDMGMLSEYIPELERSIDDSSRLLFSLD
DFAPLFLNILPVLRAIGVIVILPKSLEKILKPKLSLNLSAKGKIEEDRKS
FLTLEKLLKFDWKIAIGDKEISITEFKKLLKDSRGLIKIIDQYVLLDDKE
VEALLKKFDKLPERLSQAELMQAALGGELDGAKVGLDQQIKSLFEKLNTY
VPVDVPSNLTAQLRPYQKRGFSWLVQNIETGFGSIIADDMGLGKTLQVIA
AILYCKNVGFLDEDKVLIVAPTSILSNWQREMERFASELKLFIYHGQNRE
LANDYDVALTSYGLARRDKKELNRVGWFLLVIDEAQNIKNPHSEQSKAIK
AIAAKNRIAMSGTPVENRLLEYWSIFDFTNKYYLGTPKEFKTHFATPIEK

ARDKVCLEKFMKVTHPFMLRRVKSDKSIIEDLPDKIENNRYCSLTPEQTA
LYQEVVNTTMKKIERSEGIERKGLILKLINALKQVCNHPSHFGKKKRASI
EQSGKMQMLEEILIGISELAEKSLIFTQYTEMGEIIARLLEEKFESKVPF
LHGSLSRKARDKMVNDFENSFRSNILIVSLKAGGTGLNLTAANHVIHYDL
WWNPAVEAQATDRAYRIGQERNVMVYRLLSTGTFEERIDEMIQSKKELAN
LTISSGESWITEFSNNQLKDLINIRNAV

>WP0321_wPip_WO2_CAQ54429_RNA
MKSRNSYSGIDPKVVNLIKFYAKYYKSLTDQDIEDIEQDLFCEFLSCIDQ
YDKTKGSLSTFAKQVVKNRINNLVRKHSRTTPKLENNVQQESYCFEDETI
LRIDVEQIISKLPKKWQVLCEQLKHHSIAEVADMNNMSRTTLYNILKKMR
SKFAPVYGKKHKKN

>WP0322_wPip_WO2_CAQ54430_conse
MKIINSKERAKMVTGVKMVIFGPYGIGKTSLLKTLDESTTLCLDFEAGLL
AVQDWKGDSTEIRTWNEARDIACLIGGPNPALRSDQAYSQKHYEHVCSKH
KDLLSEVSKYRSIFIDSITVASRLCFSWARMQPEAFSDRSGREDKRAAYG
LLAQEMMAWLNQ

>WP0323_wPip_WO3_CAQ54431_Phage
LLKVWINTTLGETWVDKGEVPDWKQLFNRREFFPVGTVPKGEVVLTAGVD
VQKDRLEVEVVAWGKSRESWSIDYRVFEGDTGGREVWGKLSELLNHHFIG
ENGLEYMISMMAVDAGYATQEVYNWVRGHQGSGRVMAVKGVNKALVPLSS
PSRVDITVGGQKLKRGIKLWPVGVSILKSELFQLLNVLKDGEEAPAGYCH
FPEYAPEYFKQLTAEQLVSKVVKGYTKQEWQKVRERNEVLDCRIYARAAS
IALGIDRWPESKWNSLSGKMESKKPKKVIQSKWLGNQNVQ

>WP0324_wPip_WO3_CAQ54432__Puta
MYSEEYLTQVEKAIQKLQSGERVVSIAYGDHVVRYGEVQINDLLNLRQRI
KAELKVAGMRPKRKIVFSTSKGIL

>WP0325_wPip_WO3_CAQ54433_Putat
MLLKTFKQLFNKPKIKSSAWDTAGSGRRFFHFQPELGSINNLLSQSLETL
RSRSRDMVRKNPYAANIIDTIVSNSIGTGIKPQSKAKNAEFRKKVQELWL
KWTDEADSSGVSDFYGLQALVCRSMIEGGECFVRLRNRKLEDGFSVPLQL
QVLESEHLDNKSNQTLANGNIIRSGIEFNRLGQREAYYLFREHPGEGSFG
ESVRVPANDVLHIYKPLRPGQIRGEPWLSNILLKLYELDQYDDAELVRKK
TAAMFAGFITRLDPEANILGEGESNEQGVALSGLEPGTMQLLDPGEDIKF
SEPSDVGGSYEAFMRQQLRAIAIGTGITYEQLTGDLTGVNYSSIRAGLIE
FRRRCAMLQHNIMVFQFCRPVWSRWLELAVLCGELRIDEKVAKAAKEEVK
WIPQGFDWVDPLKDQQAQQMAVRNGFKSRSEVVSELGYDVEEIDQEIAED
QKRADSFGLCFDSDINHKREGR

>WP0326_wPip_WO3_CAQ54434_Putat
MWINKPVMVERRSFELLSLYNSKQPIFKNLKHFHINPKGIAIIRIYGVLT
KKTEAFDHILDMTSYENIHEEIESALEDKSIETILLDIDSPGGEVNGVFD
LADFIYGVRGKKRIIAIANDDAYSAAYAIASSAEKVFVCRTSGVGSIGVI
ASHIDQSGFDEKQGIKYTTIFAGKRKNDLNPHEPMTSESLESLQKEVDRL
YEMFVQLIARNRGLSIEKIRSTEAGLYFGEKAVEIGLADGVTTFFEFINK
GENTMNKQTTTDLEIDDLETDNLTKYRTEIVELIRLCNLSRMPEKIGEFI
EQGVSIEQAREVLMELLAERTKKTEILSAIPQNSQEDLMTQVAKSRCI

>WP0327_wPip_WO3_CAQ54435_Putat
MISITEGNNLGDLLKYEVSNLYSRDQITVAKGQNLKLGEVVAKKTEDGFI
RVLNPVGTDGTQTAIGAIVSDVNATENSKAVIITRGAILADHAVVWPAGI
TEEQKAAAIKQLEGRGIIVRKGV

>WP0328_wPip_WO3_CAQ54436__Puta
MQNPFTNTAFSMTALTNAMNILPINYGRVENLNLFPNRSVRFRHITIEEH
NGVLSLLPTQVPGAPATVGKRGKRKVRTFTIPHIPHDDVVLPEEVQGIRA
FGSESELKALADVITDHLQLMRNKHAITLEHLRMGALKGIILDADGSELL
NLYNEFEITPKVVNFALGTATTDVKRKCLEVLRHIEDNLSGEYMTGIHAL
ISPEFFDALTSHTKVKEAYERWQEGAALRNDMRSGFTFCGITFEEYRGQA
TDPEGTVRRFIEKDTGHCFPLGTASTFTTYFAPADFNETVNTLGQPLYAK
QEPRRFDRGTDLHTQSNPLPMCHRPGVLVKVAIA

>WP0329_wPip_WO3_CAQ54437__Puta
MQENIKRLLEDCFAHLGEVALYESKNKAYMVQVLKQQPDKLYEIGEGQFV

GEMLILEVSVFDILKPMVGDIFVIGNCKYKVHSLPLRDKSGMIWRIEASG
V

>WP0330_wPip_WO2_CAQ54438_Putat
MSVHIEVEVIESANAKRKKIELAIVRALNRTALWLKSKAAKKISEEKKSL
IRKRLRIFKAKTSRLEVLIRANLYDIRASTIGKIQKTRRGSKVGKHEFIG
GFAAVMPKGNSGMFKREGRAALPIKEVKLPLEPEASRIIENFVNYEVEKV
FEKFFHRELSYITPI

>WP0331_wPip_WO3_CAQ54439__Puta
MNWQDLHKAICTKLKEEISAIQTCEIYPAIRKELLAPAVFVELSGFEKGH
DPGTEELALKARFEARIVIDSTIENAPVIVRTLAAEVARVVNKNTWNVEN
VSPGEFISAEIDGFRPELDAYLVWVVDWVHEIHIGKSVWSETGIKPHIIE
IGNVHVGS

>WP0332_wPip_WO3_CAQ54440_Putat
MLDHSFAISELNRKLANVIRIGVVKEIDYEKAKVRVKIGEFLTDWLPWIT
SKAGKDRDWSPPDIDEQVVILSPLGELSLGVVLPGIYQQKYSAPENKKEI
NSVKFQDGTRLLYDKEKHHLEIEVMDKVTLKAGESSIEMTKSGIKLKADK
INLN

>WP0333_wPip_WO3_CAQ54441__Puta
MNKSVVRVGDHCAEATPHFCVSGSNNVFVNGKPVCRKGDNFTEGRALTEG
SKTVFANGYSIGRVGDIVSCGFKVIKGSESVFAK

>WP0334_wPip_WO3_CAQ54442_Basep
MKGMDAKTGKALEGIEHLKQSVVDILTTPINSRVMRRDYGSRLFELVDKP
INRDLTLEIYAATAEALGKFEKRFKLEKVKITEVKEGRVTLNLEGVYLSE
GKFININGVVV

>WP0335_wPip_WO3_CAQ54443_Basep
MQQPNIIEPLNFEEIFFRMKEELVSRDASFTGLVESDPAIKILEVAAWRE
LLLRQRMNEAVKSNLLKFAKGEDLDNLAEFYGVEREKEEEDERFRKRVKA
KIAGWSTGGSKEYYKYHALSADSRVKDALVESPIPGKVQISILSTQTGIL
SEELLEIVKKQVTRDDIRVLTDTVTVVGCNITEIDIHSRMSISPVISKEE
IKKQFIEKFELAKRLGWSVTRSWIIANLFVDGVENVELIEPKEDVVVLGN
ECANLRNLKIE

>WP0336_Pip_WO3_CAQ54444__Putat
MLLPPNATKQEQALVDAIDYKVDPSCIRRFKFSLKEETLPWIIEEYGLEE
ILRWVKDRRKAVIEGVKFQRLRGTPASLKIALKWANIEDIKIIEEPPGKH
FFELQVGIKEVPNDFFVDAVVELAKLSLPVRSRLMRIFNDYYNVQRFILD
ESLFGDLLSDYSGVKIEKDGPVLSFGRVNFFRSSGPVIRIIENYLRDHYE
RALSNDIYRLDVAVLGETEAHTKDYKGIYERSHQWYNLKALYPLSQSLLP
EIKFAKAQIVLSDSWKLGEINGCFPVSSVEEKGNKFVLGNDKLSGQRWNL
KHKPILERFSIIHRYKVENYTDQKVRKYVLAEHNIYYKNDLYSEQKDSIH
ELEKHILVFYPGVLKWHEHRHLHRSWKNSQVISIIS

>WP0337_Pip_WO3_CAQ54445_HP
MVRQEVLGEQLVKIQHKQSLANSLEQTTMLHEVTSVIELVEWTMQGTTKV
KGLSPEIPEDIEVDIFISVEDSMRDNAKASYHSLYRDLRPCHVLKWTSSE
LGRSYAFL

>WP0339_Pip_WO3_CAQ54447_ankyri
MKTNIDEPCAGNLQARFFRGSCSNKTQTRGVKLQLLPNSFSKSKFFKEVS
SKDINKRNEEGETILHQAVEISNYKTVRFLIKKGAEVNARDKNGYTPLHC
AVFAKSLENVKVLLREGAEVNATQYVTGCTPLHSACKIGGAGVEIIKELV
KAGAEVNQLNKYGATPMYYIWESEKYCSWNREENEKASKFLREQGGITRS
RELTCYGIERLVGEIADMLNGSYMPELKIIEIGEIRKRDKSLIKEECKNL
ASKIMSQVNEMIDEVAKRKV

>WP0340_Pip_WO3_CAQ54448_Ankyri
MLKLGKFNKSAKELLENSYKNIYARDENGRTALHYAINAKTVRVLVEKGV
NVNAADVGGYTALHLAVTEKRLEIVRELIKSGAEVNAEEYGNKCIPLHLA
CMVGEKEIVEELVKAGAEIEQADKFGMTGMDYAKNSKEITEVLKKETDRI
EKLFMRG

>WP0341_Pip_WO3_CAQ54449_putati

MEEETEKKVMNLEKKALAELRKIWKKVYEEEAPRYSKKYLIPRLAYRMQE
KAYGEMSRKGAKRLEYLADRLEKGKRISSDKLPVEGTELILERGEETHAV
MVTDKGLIYREEFFTSLSAVAGKIMGMSYNGPLLFGMRDKNEN

>WP0342_Pip_WO3_CAQ54450_site-s
MLKEVRCAIYTRKSNEDGLEQKFNSLDAQRVACEKYIKSREGWVALAKRY
DDGGFSGKNLERPAIKGLFEDVKTGEVDCVVVYTLDRLSRETKDSIEVTS
FFRRHRVNFVAVTQIFDNNTPMGKFVQTVLSGAAQLEREMIVERVKNKIA
TSKEQGLWMGGTLPLGYDVKDKELIINEKEAKIVGHIFERYLELKSMAEL
ARELNSQGYRTKSDIFKKATVRRIITNPIYMGKIKHYEKEYEGKHEAIIE
EEKWQKAQELIRNQPYRKAKYEEALLRGIIKCKSCDVNMTLTYAKKENKR
YRYYVCNNHLRGKGCESENRNVIAGEVEKEVMRKTEQLYGKLGEKAEEWK
NLSFGKQKEVVKKLIKGVMVREDGIEVSLEDKVEFIPIKKKGKKCTVIEP
EGKTNNALLKAVVRAHLWKRRECSKKCVKPHF

>WP0346_Pip_WO3_CAQ54454_ankyri
MLKRFANNRTLENQDIEIVKLLLSKGANPSIIQGNSELVSLLRQYLFSAF
KDRDLKRARELVKEVKDLDIKESGKPLLHCFVENACQDGNWFEFLKEVLS
KQRVFCSLFGTGMRFKMDINICDDEHNETALNYACRCNKKDVIEFLLDSG
ADVNKVNGIYAHRTPILIALKYNYYDSVRLLFEYGAKIDISSGIEQEIIN
NSREYKRETMEILLEHTSPQQNTKVLECFVNNETSRSVDIEIVKLLLNAG
ADLSIIPRGRFTECSFSTFNNKDLETAKLLIDHVDLNKVFKVLRGNKEVE
ISLSYLFLMNACLGTDWFEFYEDVLKDRKVDINAVFSHGYTALGYACKKG
KEDVIKLLLSKGAEINKGKKTPIYFALKNNDDRLASLLFEHGADINATPE
LGQEIIANSYNKRSKYTKETIKILLQHTSKEQNTEILRRFANNGTLENKD
IEIVKLLLSKGADPCFGNPNAVALATEQGNTQLKDLFSQSQLDSESQEAQ
VTLLGSEQGFEGSDNEFYSAEEDIVKGTSEDKYEGNTPQTTAEEDCNGQS
GDQKSSEPESAGSQQTEKNVLSSTKKSNAINNAGASMEETVSNDHKKGKM
HANTQQGTSSLEQNKKVSEAAKLAGGNSQLKESLNSTQHQLIEELQEQLA
SKDKGIDAANQALNAKTKEFESAKAQLERELGSVQQELDKTTEELRRKKA
DLENSKKELGNVNEKVLKLEHETLNQQASINDLTKERDQLASRTNTLTEK
LKNESKKLNDANQALDTKISEVTQLTKQNSQLEEQLRKELNNVAEKLTQE
QQKAFELGNQVTTLTKELKEKKAELERENSSLVSQVGELKGEFEGIKTKL
AGKEEELRSKIKEVAELSVTVGKLERQTEELMATQAEFETEKAKLKSELT
KQDEVLESTQAQLKQEVSSLKGQVTQLAKEKDQLAKQLNDTQQNPNPDNE
NKKLLAVSAQSRKQITYASVSFVLSGAFAVGASLTMFHLTTCISLAVVAL
TFLAVGCYCSYKANTALSNVEIDNCVNPAVVEV

>WP0347_Pip_WO3_CAQ54455_ankyri
MSDTISNEGSIIALLKEFMPGFNVNGLSEIDHYKILGVSRNATLTEIKEA
RNRLALIFHPDKIISPEEKPIYNKLLCKANNRQAFSQSEEKDWAKFQEKF
NSAKKINIAYEVLSDEKKRNDYDRKLQVDNSGLNNFKRSSKNTQSTDRET
EKNKKDIKVRLFHAFHNGDLQEAKNLVRKVDDLDITLPEYPIGSALYFFV
LRACMSEDWFKFYQDVLSKQGSNVDLNIEVHPKLGTALSAACSKERPNSK
KRDVVKLLLSYGVDVNKVSQSRERAPILYALKNNDHDLASLLFKHGAKID
VTPELESSIILYSNLRGCSKYTKKQWKFCCNILLLSKTLRC

>WP0409_wPip_WO4_CAQ54517_putat
MDAKVSHAEITKRVAACVVDAIIFLSFYLFTLFISSDKSFTEFFNYIFSD
TAIISTSIQGEVLETLLFIALETLMITKFGWTPGKLLFGIYIKDANTLKN
VALMQAVIRSTLKVLLLSCISNWFFILPILVLIFATFDQRKQTFYDKTIK
TVVIDYKPEKHHLNLNCVGIIRRAVAYIIDHFIIIGISLAFFSFVQIAFD
PIKADLLTTYLFFLLSIIFGVFMMRRFSGTPGQLLCGIHIKDANTLENIT
LVQATVRYVLFEAFNVFIPCYILSLEEFCNQHNSEKWSGALLTLTFVVII
LIFIFAILDKRKQLFYDKIAKTVAIYYKSSR

>WP0410_wPip_WO4_CAQ54518_ankyr
MHVNQQQSSNGTINKIVQLLQSGTFKQKNLSDKISHEEISRLVNDIISSS
EDNTVKLHRSKRGIGNDIYKIATYIYRWYQLSKKLGWDRSSQVVTRHLGT
QSGQQKALDTARGALGEVETLGDLIDTLKRFLYPTGVPQVTEVPQVTEVS
YTTDGQQVTTEGQLVTEKQQITEVPRVTERQQVAAEQDNLDEEGFFVAKG
KDEKGDIPLDEGSGYVDIAGYFTEKGQQVTEVPHTTEGQQVTEVSQVTRG
QQVTERQQVTTETQRVTERQQVTRRQRTTEVPQVTERQQVTRRQQVTEVP
QVTERQQTTGRQQVTTEISQTTEGQQVTEDYSNTSLHDAAEQGNLNAVKY
FVERGADVNARNKGENTPLHFAAKRDNLDIVKYLVEKGADIDAKDGWTGR
TPLYIAAERGNLEVVKYLVDKGADLNSKLNDYDKTPIHEVVFHLDMVKYF
TDKRADVKDTDGNTLLHLAARYGRLDAVEYLIENGADINAKDRYGRKTPL
HWAVWNNQLDVVKYLVKKGADINVADEHEGPLHLAAAKGHLDIVKYLIEK

GANINTEASRSGRTSLHFAAQRGSLEVVKYLINKGADLNTKDKNGEIPLH
YAVKSCHLDIVKYLVEKGADVNARNTEGETALIIAFNTQDYYCDRRLDMM
KYLIDKGADVNARNEQDRSVLCLAAGDRRWNDFDFLIENGADINAKSRCG
GNTLLHFAIMGNQYWCHRCIYPSPSSLNTIKYLVERGANANVEDNDGETP
LDIAKSRGYSQIVEFLSSYQHGDRNRRDIVPSNSWMNNSVVIWVKGLISS
TGSPALLTSRGHNSTTVTSVPEINTTVVNNTLLLGILAASLFNKTQYKQP
IHESLLSPREQLMRNIDDSSRLLEESLQKAKEEGWSSPSTDVNEVKISDN
KTGMWK

>WP0411_wPip_WO4_CAQ54519_site-
MLKEVRCAIYTRKSNEDGLEQKFNSLDAQRVACEKYIKSREGWVALAKRY
DDGGYSGKNLERPAIKELFEDVKAGEVDCVVVYTLDRLSRETKDSIEVTS
FFRRHRVNFIAVTQIFDNNTPMGKFVQTVLSGAAQLEREMIVERVKNKIA
TSKEQGLWMGGTLPLGYDVKDKELIINGKEAKTVKHIFERYMELKSMAEL
ARELNSQGYRTKARFDIFKKATVRRIITNPIYMGKIRHYEKEYEGKHEAI
IEEEKWQKAQELIRNQPYRKAKYEEALLRGIIKCKSCDVNMTLTYSKKEN
KRYRYYICNNHLRGKSCESVNRTVVAGKVEKEVMKRAEDLYEKCGEEWKN
LSFGKQKEVVKKLIKGVMVKEDGIEVSSEDKVEFIPIKKKGNKCTVVEPE
GKTNNALLKAVVRAHLWKRQLEEGKYANIKELSAKVNIGTRRIQQILRLN
YLAPKIKEDIVNGRQPRGLRLVDLREIPMLWGEQMEKFYGLNLQK

>WP0412_wPip_WO4_CAQ54520__Puta
MEKEIEKKVMNLEKKPLGEMRKIWKKVYGEEAPRHSKKYLIPRLAYRLQE
KVHGEISRKGAKRLEYLADRLEKGKRISSDKLPVAGTELILERGEETHAI
MVTDKGLIYREEFYTSLSAVAGKIMGMSYNGPLLFGMRDKSGN

>WP0413_wPip_WO4_CAQ54521_Ankyr
MIKLGKKEKSLLESSFKKKETKRELHYAVDAKTVRLLVEKGANVNAKDVE
GYTALHLAVTEKRLEIVRELIKSGGNVNAEEYGNKCTPLHLACMIGEKEI
VKELVKAGGEIEQADKFGMTAMDYAKNSKEITEILKKEIDRIEKLFMKG

>WP0414_wPip_WO4_CAQ54522_ankyr
MKFSKESFSKFCKEVSSKDINKRNEEGETILHQAVEISNYKTVRFLIKKG
AEVNARDKNGYTPLHCAVFAKSLENVKVLLREGAEVNATQYVTGCTPLHS
ACKIGGAGVEIIKELVKAGAEVNQLNKYGATPMYYIWESEKYCLCDSKES
EKASKFLREKGGITKSRELTCYGIEGLVGEIADMLNGSYMPELKIIEIGE
IRKRDKSLIKEECENLASKIMSQVNEMIDEVVKRKA

>WP0415_wPip_WO4_CAQ54523__Puta
MLLPPNATKQEQALVDAIDYKVDPSCIRRFKFSLKEETLPWIIEEYGLEE
ILRWVKDRRKAVIEGVKFQRLRGTPASLKIALKWANIEDIKIIEEPPGKH
FFELQVGIKEVPNDFFVDAVVELAKLSLPVRSRLMRIFNDYYNVQRFILD
ESLFGDLLSDYSGVKIEKDGPVLSFGRVNFFRSSGPVIRIIENYLRDHYE
RALSNDIYRLDVAVLGETEAHTKDYKGIYERSHQWYNLKALYPLSQSLLP
EIKFAKAQIVLSDSWKLGEINGCFPVSSVEEKGNKFVLGNDKLSGQRWNL
KHKPILERFSIIHRYKVENYTDQKVRKYVLAEHNIYYKNDLYSEQKDSIH
ELEKHILVFYPGVLKWHEHRHLHRSWKNSQVISIIS

>WP0416_wPip_WO4_CAQ54524_Basep
MKQLNIVEKLSYEEIFSRMKEELVKRDASFTGLVESDPAMKVLEVAAWRE
LLLRQRINEAVKGNLLKFATGEDLDSLAEFYGVERQNEEDDEHFRKRIKA
KIAGWSTGGSKEYYKYHALSADSRVKDALVESPIPGKVQISILSTQTGIL
SEELLEIVKKQVTRDDIRVLTDTVTVVGCNITEIDIHSRMSISPVISKEE
IKKQFIEKFELAKRLGWSVTRSWIIANLFVDGVENVELIEPKEDVVVLGN
ECANLRNLKIE

>WP0417_wPip_WO4_CAQ54525_Basep
MKGMDAKTGKTLEGIEHLKQSIIDILTTPINSRIMRRDYGSRLFELVDKS
INRDLTLEIYSAVAEALQRWEERFKLEKVKMTEVKEGKVTLDLEGLYLPS
GKNIHFDEIVV

>WP0418_wPip_WO4_CAQ54526__Puta
MNKGIVRLGDYCGEAIPHFCISGSNNVFVNGKSICRQGESFSEGKVMIQG
SKTVFANGLSVGRVGDIVSCGFKVIKGSESVFAK

>WP0419_wPip_WO4_CAQ54527_Basep
MLESNFAISELQRKLANIVRIGLVKEVDYEKAKVRIKIGEFLTDWLPWIT
SKAGKDRNWSPPGIDEQVVILSPLGELSLGVVLPGIYQQKYSAPENKKEV

SSLTFQDGTKLSYDKDKHHLEIEVVDKITLKAGESSIEMTKSGIKLKAER
IDLN

>WP0420_wPip_WO4_CAQ54528__Puta
MNLKDLHDKICTTLKREIPAIQTCEIYPSIRKELLAPALFVELVSLESGK
DPGTEELALKARFEARIVTDSTIENAPIIVRTLAAEVARVVNKNTWNVEN
VSPGEFISTEIDGFRPELDAYLLWLVEWSHQLHLGKSIWTENKIKPHTIT
IGENVRE

>WP0421_wPip_WO4_CAQ54529_Putat
MSVHIEVEVIESVNAKRKKIELAIVRALNKTALWLKAQAAKEISEEKKSL
IRKRLRIFKAKTSRLEVLIRANLYDIRASAIGKIQKTKRGSKVGKHEFIG
GFAAVMPKGNSGMFKREGRAAFPIKEVKLPLEPEASRIIGNLVNYEVEKV
FTKFFERELSYKG

>WP0422_wPip_WO4_CAQ54530__Puta
MKNISKLFKDCFEHLGQQALYESKDKSYMVQVLKQEPDKLYEIGEGQFVG
EMLFLEVSIFDILRPIVGDIFVIGDCKYKIHTPPLRDKSGMIWRIEASGV

>WP0423_wPip_WO4_CAQ54531_glyox
MTELLGKIIPILIIKDATSAIELYKQALNAKVLEIHFDENNKNMVMHAAI
QIGDSLVYIDDVQSNSIAGGNVLQAAKLCVYVENADELFNKAVSKGMQVE
RSLENMHWGYRFGVVKDEYGIQWCFSSPLKK

>WP0424_wPip_WO4_CAQ54532__Puta
MQNPFTNTAFSMTALTNAMNILPINYGRVENLNLFPSRSVRFRHITIEEH
NGVLSLLPTQVPGAPATVGKRGKRKVRTFTIPHIPHDDVVLPEEVQGIRA
FGSESELKALADVITDHLQLMRNKHAITLEHLRMGALKGIILDADGSELL
NLYNEFEITPKVVNFALGTATTDVKRKCLEVLRHIEDNLSGEYMTGIHAL
ISPEFFDALTSHTKVKEAYERWQEGAALRNDMRSGFTFCGITFEEYRGQA
TDPEGTVRRFIEKDTGHCFPLGTASTFTTYFAPADFNETVNTLGQPLYAK
QEPRRFDRGTDLHTQSNPLPMCHRPAVLVKITV

>WP0425_wPip_WO4_CAQ54533__Puta
MISITEGNNLGDLLKYEVSNLYSRDQITVAKGQNLKLGTVVGYDTKDGFI
KALNPTATDGTQTAIGVIASDVNAKENSKGVIIARGAILADHAVVWPAGI
TEEQKAAAIKQLEGRGIIVRKGV

>WP0426_wPip_WO4_CAQ54534_putat
MEILQTNWLCRPMMIEQKSFDLLSLYKGKQPILKNIKHTVNQNIEKTAVI
AIHGILTKKPGAFDVFLGMTSYEQIEEQITQALADSSIETIILEIDSPGG
EVNGIFDLADFIYESRAKKRIIAIANDDAYSAAYAIASSAEKVFVSRTSG
VGSIGVIASHIDQSGFDEKCGIKYTTVFAGSRKNDLNPHEPMTSESLESL
QKEVDRLYEMFVQLIARNRNLSIEAIKSTEAGLYFGEKAVEIGLADGMTI
LSSINKNRSITMNEQTTNDLETDNLTKYRTEVLELIRLCNLSKMPEKIGE
FIEQSVSVEQAREVLMELLAERTKKTEILSAIPQNSGEELMMQVAKSRAQ
SNI

>WP0427_wPip_WO4_CAQ54535_Putat
MKITQTEWAREIGVSKQYVCYLVKKGIVELEDGLIDREQANRAIETIRDP
SQPLRRKNYSESGEKLSTMLLKTRIKNEMERGKLLEAKAKAEIGELVAVE
EVKSEAFNVARIEFNKLGQREAYYLFKEHPGENTFGESVRVPANDVLHIY
KPLRPGQIRGEPWLSNVLLKLYELDQYDDAELVRKKTAAMFAGFITRLDP
EANIMGEGEASEQGVALSGLEPGTMQLLDPGEDIKFSEPSDVGGSYEAFM
RQQLRAIAIGTGITYEQLTGDLTGVNYSSIRAGLIEFRRRCAMLQHNIMV
FQFCRPVWSRWLELAVLCGELRIDEKVAKAAKEEVKWIPQGFDWVDPLKD
QQAQQMAVRNGFKSRSEVVSEMGYDVEEIDQEIAEDQKRADSLNLFFDSD
VRTQTK

>WP0428_wPip_WO4_CAQ54536_Putat
MVVVILIYWYIPYFNEFSVTNNFGLNVVYLMNQEKTLAGVVTSQTNNEAS
FSEEVENNVSNSTLSVKERIELFEKGTKQEPDLNTECKLASLNVISNSSD
SSITAKIDEAGADFSVSCSSLVFDDGKDIEDEFNAVCEANGIEYNRPYSL
DQLEAASNTSSNPKIDNTKEMQKEEEDKELGDKPKPLPRNGMLEKDNIRN
VAPPSPRTAENHRLSIIQDIAPPKLPHSSFQLNGEKCDEQEAIGEPMYAT
IDKNKKFSKRGLDGEINDQSPEMEKSNSEETSIKKGSIDEPIDLNTKFLK
KSLEGSDSGIDNRSAEMTRSNSQVTEMSEFCGNQGWENMSLNHDSPVTLS
TSSSEKSDNEETPLIKKEKKTNRILPRVKYAVQQKEFVIFGIAAVALSAS

AALVYLQDKGQFIAFFANSPLYITMPMIAITSLLAISTIFCGIKQFRDTE
ECQIGGKNANETLNQVLKYQPKDKVIKSFKLEYSNGTHSHFTLNAWKGKN
NFINIDDKVISRRNKVESVIKDRPLFTALLSSLVAANIVLPLLLYVEGGI
HSVQKFYQNALINNVGLSVLVGSSILALSVIFLGVHYYRKTNCTNLVYCE
ERIEPEKVNGKFIEETKEARTKVLEENHSKDAKCSSLTLEQVVVESHNYK
DVIYSIS

>WP0429_wPip_WO4_CAQ54537_Phage
MNLAIHYYPTRDLVEYERNPRKNDDVVNRMCASIREFGFRIPIVAKSDGT
VVDGHLRLKAARKLGMESIPVVLSDNLNEPQTKAFRLLANQSANWAKWDD
ELLKVEIQELEDLQFDLKMTGFELEKVQHFLDDLDSEKEDLSDLVVDDKK
VEITKPGDLWILGDHRIYCGDSSVVESYKALLDDKMADITVCDPPYNVDY
GSSQEREDKKILNDNQGEKYELFLYDICSHILAYTKGAIYICISSSEFST
LQKAFEEAGGKWSTFIIWAKNHFTLGRSDYQRQYEAMLYGWKSGNKREWH
GGRNQSDLWFYDKPTHNTLHPTMKPVELMERAIVNSSRPGDIVLDPFSGS
GSTLIACERTGRICRTIELDSKFVDVTIKRWQVYTGREAILSGAGKTFSE
IQEEKQ

>WP0430_wPip_WO4_CAQ54538__Puta
MSILTLDLGKQTGWAILTDGVIESGSESFHTSRFSGGGMCFLNFRNWLNS
LKEISVVYLEEVRRHLGTDAAHCYGGFLAVLSAWCEEHHVPYKGVNVKTI
KRFIAGNGNASKSEVIEAVKEKGFLPRDDNESDALALIFYVMNFSKDFNT
LKIP

>WP0431_wPip_WO4_CAQ54539_Putat
MDESKIIFYTTPGGNIEIEVLCRNENLWLTQKKMAELFDVQLPAISKHIK
NIFESGELQEKEVISTFDVTAADGKNYPTQFYNVDTIIAVGYRVNSKKAT
SFRVWATKILRDFIIKGFALDSERLKNGPKFGKDYFNELLEKIREIRASE
RRFYQKITDIYAECSADYDPNSEITKQFYAKVQNKLYWAIYGLTAAELIC
SRADHKKLHMGLTTWKDGPGNKIHKSDVSTAKNYLTKEELSELNHIVSMY
LDYAELQARKHRLMKMQNWVEKLDAFLLFNDYEVLKDAGKVSAEVAKALA
EGEYEKYRVIQDKLHESDFDELIKASKESEKITTTKTSL

>WP0433_wPip_WO4_CAQ54541__Puta
MHDKQALFERAAEVKAELLSRIESSLSYLLPNGTFHGGKFYVGNIRGDKG
KSLVVETRGERAGLWHDFATGEGGDILNLWWDVTSQTKFLDTIEDIEKWL
GHSTNHKEEFSEHLIACWDYHDENNQVIAKIYRYDSTSGKKRYSCFDVKN
SSSTAPDPRPLYNVPGIIKSDKIVFVEGEKCAESLISKGITATTMMFGAN
SPVNKTDWAPLKGKHITIWPDNDSSGQKYAENVAKKLLDIGVASLSILKI
PQDKPKAWDAADCIKEGVDVKRFLTSTVSLPYTKNVTSFPVLQYLNDKSP
MPEDIIAPRVLTPSGLLVFAGAPKVGKSDFLISWLFHMASGTPFFDMVPK
RPLRIFYLQTEIGYHYMRERLQQMKFSEELIKLAANNLVITPQTKLLLNE
NSIKDIVTEAERNFDLNTIDIIAVDPLRNVFDADEYGNENDNNAMIFFLQ
ERVEKLRSLINPDAGVILVHHTKKMQKKLLEEDPFQSFSGASSLRGFYTT
GMIMFRPDEKNSYRQLMFELRNGNSVMPKFVDKINGHWYEVDAESQRLIN
KDYGEKLDAERSRRYDIILDLICAEGRKGHAYTINQFCQAFENKEGLGST
HSIRDRLDVLATKGYVKFNKEGKNAARNKYGVLCVEGMEFGQKIFDPEKN
KKVTVYHRLLPTHYKSPHYGSVIPVENPNTWVYHN

>WP0434_wPip_WO4_CAQ54542_RNA_p
MHSKNSYTGINPKVVKHIRHYAKLLKRGKAFAYKEIEDIEQDLLLDCLPG
LNELEGHFIKQYVKCRALNLKEKELCKKRTINFVDEALYEESDESLEYTS
AVRIDVNEAIAKLPRELRKICKLLAEDHSICEISRRTGIPKSTLYDTINK
LRKEFSHLKTYLKRDF

>WP0435_wPip_WO4_CAQ54543__Puta
MKIINSKERAKMVTGVKMVIFGPYGIGKTSLLKTLDESTTLCLDFEAGLL
AVQDWKGDSTEIRTWNEARDIACLIGGPNPALRSDQAYSQKHYEHVCSKH
KDLLSEVSKYRSIFIDSITVASRLCFSWARMQPEAFSDRSGREDKRAAYG
LLAQEMMAWLNQFQHIRDKDIIIVGTLGQYLDDCNRSTWLPQCEGAKTAS
EIPGIVDEVISMVGIKKDDGTEKRSFVCQTINTWGYPAKDRSGCLNMVEE
PHLGKLLTKIKAKAFATAA

>WP0436_wPip_WO4_CAQ54544__Puta
MLQDFFTITQKIPFFSVKEYLDDQSPIPEDIIAPRILTKRGLLVLGGPPK
IGKSDFLISWLVYMAAGVSFLGMTPSKPMKIFYLQTEIEYEYMKERLQQL
QLDNELLNIAANNLIITPRVQLSFSSEEIDEIKNVIKERFKPDIIAIDPL
RNIFNSSEYGNENDNSAMLFFLQKTLERLRNVINPDSGIILTHHTKKLSK

KMLEEDPFQGLSGAGSLRGFYSTGMVMFAHDEESTVRQIVFELRNGERVA
SKLVDKIDSRWKLADQWS

>WP0437_wPip_WO4_CAQ54545__Puta
MLSDFLTDFNNAKLQSNLIPKGTIVKVKMAIKPGGYENWFTKSYTTGSIY
LNAEFTVTEGQYAKRKIFQVIGIKSGKASVEGEDTWGESGRSMLRSILES
ARNIHAHDTSEKAVIARKINSIADFNGLEFTAKVGIEADRYGEKNKIATV
ITPEQNKNVENDWVVPF

>WP0438_wPip_WO4_CAQ54546_Putat
MSILTQSGRAAIAASIKEQSLHLAWGSGDANWESSHQVEKVFVEDEIALD
HHTIKDVRVFTGQTTYQPSIDYIVDSSSGVIKRTENSSITANSAVTIEYT
ESTPPELITSEKLLNELGRRTADEVLFCTGDENGELITPSGRFKPSNVPT
NNLYLKFTFDFTNAANQVIRELGVMVGTKVKEKLPPGQRYFEPQDIEDPG
ILLVLEHTVPLIRTSATRETFSFVVTF

>WP0441_wPip_WO4_CAQ54549__Puta
MLTANNQGKLQGKVKVPANIPAGTKLVQFYGDKGSYGEATYTGKKTITIE
ERRRVIAARRVDPLAQTFTLNESRHIGGVELWFVNKGKKRVVVQIRETAV
GVPSQTVIAESYIEPKDIKIDGTATRIEWSPVFCHAGEEYAIVLLTDDSG
TAVKIAELGKYDAVNSRWVTSQPYQVGVLLSSSNASTWTPHQNLDLTFRL
LAAKFSENSHVIDLGKVTATNTSDLIVLTNVEKVAFDTNVEFILTDEEGK
ENFLSDNLPLALRERLSGKLTVKANLKGSKEKSPVLYPGLQLVLGNLLES
GDYVTRSITAGANTKITITYDALIPGTADVKAYVQKNVDWQLVNLTSGKP
IGENLVERTHVLSNFNGNDTRIKLVLSGTVVYRPKVKNLRIIIT

>WP0442_wPip_WO4_CAQ54550__Puta
MSDDKTSRGYSLPHPENIAVQDVVRIRTTIEKIDEDITEREDEHNQLKNN
FKRFRFETFLNFWE

>WP0443_wPip_WO4_CAQ54551__Puta
MKEAIYQRIKDLAANSTPDQLAYLAKSLELIADKKAISDIVQMAKIKEII
DALQKRLKDLAENSTPDQLAYLAKALESIIDKSAVSEIVQMTDGKLKELL
DSAKKHLTDLDNKKASSLAVISESEKQLLKRIDEKGTTNLSLLDTRKNAN
IAAINSVGNDHKDGLKGLVNNFRTVNDVPSGSSIMKEVRNRHMIEPGALP
FLFGVLSRKNNYFGHGTFTTELGQWSSDVTKTDYMLQLLAGTHTYESYVS
FYRPRQLSFLEGSKGTFIYGESYPRFMYDGFSEQFNMLKYPYAALGLIFV
KNTSNVDISKTLNFVGSAMWYKDEVNYGGAGLFIGTPDKTNTRKSEISRI
TWTRIYQHEINSPEFIASGNILIPAGKTVAVLLYTSSNLHSEGERVGETP
EFFASEHTYSTIYGQFIQWGIYNFRSNFLTTGLEVDVERTLRAWQCPGLD
ATHKIWN

>WP0444_wPip_WO4_CAQ54552__Puta
MPIYIRFEHNKQVETTTLESKPIGNDWYEAPKNFDWQKSYQLTEGGEIAQ
RSQEDIELELLQNAKFSALSNLRAYYDNYTNQYTGVSHQKSKSYQIQEKA
AKSILADQDEKDKEIIEPLAKVRGITVIEMARIIEEKAKKAVKEIIKCEE
LEDITKKKIAEAKSKNELENLLNDFKQKIQRNG

>WP0445_wPip_WO4_CAQ54553_Phage
MAEEFLHGVNVIEVTSGAKTVRTAKSSVIGVIGTAPEADGQKFPLNKPVL
IAGSLKEAAKLGKSGSLPSAVNGIFSQIGVTVIVIRVEESENSDPKLKEE
ETLKNIIGGVDKETGEYQGIEAFLNSESIVHVAPRILIAPQFTHQLPESK
NPVVAALIGVAEKLRSIIVADGPNTNDEEVIKWRKSVGSSRVYVVDPWVK
VFIEGKEEILPVSPFVAGLIAKVDSEQGFWHSPSNKEINGIVGTSRPIDF
TLGNTNCRANHLNENEVTTIIHQNGYRLWGNRTCSNDSKWAFLSVRRTAD
LINDSLLRAHLWAVDRNITKTYIDDVIEGVNSYLANLKAQGAIISGKCYA
TPELNTPANIASGKVYFDFEFTPPYPAEQITFRSHLVSGTIL

>WP0446_wPip_WO4_CAQ54554_Phage
MLPKILKNFNVFVDGRGYAGKIDEITLPKLTIKTEEYRAGGMDIPINIDM
GMEKLEAEFTFAEYDTELFRLFGLINGNSVSLTLRGGMQGSGSNDIEAVV
INLRGVFREFDFGSWKPAEKATLKCTVAAHYYKLTIGGNELIEIDAENMV
RKINGVDQMALLQTVLGI

>WP0447_wPip_WO4_CAQ54555__Puta
MHTIILNNPITVDGISVSELSMREPKVRDLLAIERIEGEALKEVALIANL
ASVPKEVVEDLCIKDYVEIQKVLKGFLSPLEQRS

>WP0448_wPip_WO4_CAQ54556_Phage
MSMLSIKIGATLDGSFNSAMTGSAAKLSKLGDSIKQLDSSMKSVSKFKQL
NHDALEAMKNWKEAEKKSKESAAAIAKEKKEKKEPSKALKNEFEKLKASA
SKAKEAYIKKRDALHTLNEEVRKSGKDIKSLVRDQTKLGSSIEVLKGKYS
KLGSVIQKQQNALAKKAHYRSQVMETIGLGLSLAAPIKVAIDFESAMADV
KKVVKFQYDEEMNEFSENIKKLSREIPLSAAELAQIAASGGQLGIDKNKL
LGFTTTVAKMATAFDMSAEQAGDSIAKLSNVYGIDVSEMEHVGNVINHLS
DNSAAKAKDMVEALAIVGGTAKQFGLDIKETSSLVNAFVSLGKQPAKAAT
AINALLSKLQTAEGQGKEFKAALESIGITAEEMSQKIAQNGQDALLYFFE
TLEKVDKQERSQILLNLFGQEYQDDIALIVGSLKKYEDAIAFVADKEKYK
HSMQEEFDNRARTTANNLQLLKNTIAELGMNLGSIMLPPLNWISKILRSI
STGIAWFAEKCPILTTGVMSIISALIIGKIAVVGFGYAFALAGGGILTFK
AILQGTLLPVLTSLSARVIPAVIMGLKALTLTNPIGAAIAGLSFGAALVI
ANWQKVKDFFSSLWKSITKSIENWIGIGKFFNDSPIKALEKPVEAKIGSA
TSENNVFSKGNPLLNNNAFSGVSNSRTNIIESVITEKSSVADNSEKVLKD
CEKCEQKIFNQTFTFNISIKAEPNQDVRSLADAVIKRIREKSRDVLFDSI
EPIY

>WP0449_wPip_WO4_CAQ54557_phage
MLSLGQHKLSPTSVRYSKENRWSTIECIGKIPSLQNIGQGTENIDLEGVI
YFHNLNDFNRLKSMKEAEKNQEPSILVDNLGNVLGRFVIVRLEEKQTSYF
PCGLPKKVEFSLSLRSYV

>WP0450_wPip_WO4_CAQ54558_Phage
MTVHYIARENEMLDYICFKHYGHSSGAVELVLKENPGLAEYGSFLPAGLK
IKLPIIQEQLKKSKLKVWE

>WP0451_wPip_WO4_CAQ54559_phage
MKPEFSIEGIKDHVISVHLTDESGTIDDVAEVCVDYGNENVEVPNELNIA
LGYKETGIFPMGIYTVNEVTIQGPPKTLLIKAHATNLRISLKAKVSKEWH
QITIENLVKEIAQKHGYGHKVAEEFKNVLIPHINQADESDISLLTKIATE
REAMAKLAGGYILFISKNMAKSATGKALGTTTIIPQDTINWKVHFTVRDK
YNSVVAKWYSYEKGETIKETVGSGEPSYIMLELYSNAESALSAANAKLKQ
LKRNNETLDITMPGNPELFAEAKLNLIGFNQAVDSEWIVNRAEHTLNSSG
YLTMLSASLSK

>WP0452_wPip_WO4_CAQ54560_ankyr
MRNMKKIKSNEKYKLHFAWFALLIVCLVITYCYQKSKATDNYKTILRIAS
ENCNLDVVKFSVKNLLSINTQIPRLTALHCAAEGKCLELVKFLVNEGVDI
NDTGRYKGWTVLHSAAYGGNLEIVKFLLERGANPNTRDTDGKNPRDVAVI
ESRHNKDKPYREIIKLLANAEEQHKSK

>WP0453_wPip_WO4_CAQ54561__HP
MQKQKIPITVIVTMLIQTVATIWWLAKLDLRVHMHDKFIEQNNGLNVTVY
RLEERVKNLSEELDEFKADLK

>WP0454_wPip_WO4_CAQ54562_Putat
MYFFMIRYLLILLTCSLLSGCVSCPSTTVCFTPGENCTNLIIDSVEHAKG
SVLVQAYQFTSKPIAESLIQAKKRGVEVKIILDKSQIHSKYSVINELFEQ
GIKIYIDFKPAIAHNKVMIIDNQKIITGSFNFSDAAQKKNAENLLIITGD
PPLVEQYIKNWKDRQSQSDAYTPKVEESLL

>WP0455_wPip_WO4_CAQ54563_patat
MKKYILSVDGGGIRGIIPAIILAEIEKRTRKRIAEIFHLMAGTSTGGIVI
AGLCKKDKQGNPQYSANDLVEFYQKYGAYIFKSSFLRRSIFSWLNCAQYP
HKNIEFVLDKYFGEDILKNTLSKVLITSYDINNNYPFFFKSWREDRNFIK
LKDALRATTAAPTYFAPKYLKINQKEMVLVDGGVFANNPAACAYASGKRL
FPNDEIILLSIGTGRTDRSIANSRRFGKIGWIKPLLNVMFASSLDAVNYQ
LDQVIADKYIRIQSQLKIASPDMDNITSKNIKSLQQEAKAMVEGNQKVID
KFCDIVS

>WP0456_wPip_WO4_CAQ54564_HP
MVSHERKHTVAFCISAIAFCVGISLIAAVLTTIAPLIAIIVSTIIGAITI
LSACQAIESYSVIKNSNKVTSSLHEIKEAFTGKESELFIEKVFNHLCGFF
SSEKESSTQR

>WP0457_wPip_WO4_CAQ54565_Putat
MFISVKIVNNNTKNISPARYKIAQNVRSWRLKQKYALKDLVDKTGIKYHT

LLRYEQGACGIPIEKLKVIAEGLSIHVRNLFPRRKALRESGCFDESKIEW
MYNFIAKTRGRKAIYALTKSIGAEEENNVKAARIKIAKNILKAGFTNDII
YRAVGLSTEEYSDKESIHYTSQNGGQEIKKWRIIRGYTQEELAKKLNVGP
SQIHHYEQGSVKILSERLWEIAEKLSVNAEDLIREKDCYEDDEGEKELLS
LARELKKINNQESRDELDIWVEFLSQRKQVYKEKIYKAEAIKVANNLRML
DVSIDAISKITDLSIEELENYAFKT

>WP0458_wPip_WO4_CAQ54566__HP
MTTKKTVSFDDTDGNTYELIIDSKKLPKGLYSAIRRTSKKNQQLILVDET
DPKIFDDKSRFENPEGVPSPSDVSGKSINLGEGREVYNKKSSNRQNPFIP
ALSKIEPSASENAELRWFKSRVVKAKSVDELNKTIDQALSTGIRINAYSE
GQKSFADTVISKMPLIKCKENEKEDIICELMLNGAIFSYDLLQDKGISEM
HNKLYQEIRPQIDEWLKELREIGESAIENEGIIEDVEMDNQTFFMKFSEN
SKVNIARVLEGTKNLGLTTGEVKLGGDVIEIGDSKVEVRSEKEGERNYVD
VSDNSAFEITFPTSIGSLKIVIYHNAENDHEIEVRVADKEMWSELQKKGE
NIGKGCFFGGMTVTEAVEKGSFPRSGKWSKEKVKEEIKAISNNEALSWVD
RVCGGSKETSRKL

>WP0459_wPip_WO4_CAQ54567_Putat
MNNGKKLTEEIERVLQSGGGSALFDREIVSVLLGAVHDRKQAQDVTKTLM
DNCPGIGEILGREIDDLKMIEGMTEHAAAAIECVKEAYKRALREGLKRGP
VMDSQEKLIEYVRVNIGFSAKEAVLIIYLDKQNRLIRDEVYFGTIDEVHL
SERKVVKKALSMEAASIILSHNHPGGSLEPSEDDKVMTRELVSACQGVGI
ELEDHIIMTDKGYCSFKERELL

>WP0461_>wPip_WO4_CAQ54569_HP
MALSKFLDPKLDLTFKKIFGTEKNKNILIHFLNDILGFTEINTIQEVEFL
STIMDPEIASDKQSIVDVLCRDSTGARYVIEMQLARDKGFEKRAQLYAAK
AYSRQADKGGKYIDLKKVFFIAISNCNLFPDKLDYISSHTIRDEKTNEHD
LKDFQFIFIELPKFPKSREEQLENVVDRWLFFFKYAEETTDEDLRKIAEK
SPIIKLAYDELDKFHWNEKDLVAYEERVMDLQKEAAILEQKLDDAKHEGR
QEGIQIGHEKGKIEGKIEVAKNLLKAGISIDVVSQTTGLSFDEIKQLQAE
KTS

>WP0462_wPip_WO4_CAQ54570_ankyr
MLKARDYLKSEVTDPVKEEQNAEQSTKSFHPKLRVRRQYNPNDNNLRLLS
LISLYKNYPALINDIKDLLNDVDINYVDKDGDTFLHAAVSEGHQDIVELL
LNVPDIDPKIKNNKGDTPLDIAVRSGNQIIIGELQKYSELGSSNQQVSSV
EQQPRSDDTLKELQTREPVSIFQEINKAKDQSKKRNLEGLLNKLRSSYSD
DLKIGVALKAGDCFFDSVAQGLNELKDKGLVENSERFNVKSLRKDCADYA
RENNKGETFRKYLKDIKNMAKGTDSPAIWERPEIEGQMISNKYDVVLYFY
STNEENGQKITVTKITPGSDDPEVFIEGENLDQLHIDSNEKVVRIVNYKK
HFVPLLSRLEKDIEKAIEVSREEVYGVENKKSYSLIQDIGNFNPEENNDP
NISEQAESQAQHPKIAEQQLDPLEGRAKRADNEPGPSSQIGQDSQSSGQQ
TRTRSKKPRSELIEMFLSEVTKEFDDSFEKLLKEVPSGHPAPSYDEFFIY
FFLGSTITIPDRLIAKIGIKSVHFNLNFTDKGSKIIKLLFEINKDPQDRR
LVLRTIIADGVKTLSGQYYSNDNIQEIVEKLNLAGDDANSYKVDARIFHI
TLSKDTGRVLRTSEDQKTKQDLEKALQKIQANVIEGVELQDKAKEFESIE
LQNKDDSSKFENALENLSKKINAVEEGVKELFKGFIEVCKKNKKHFPKVA
GRNKNDHNRYKESRFHGFIYGALVLNFKSKYYLNCYAERSSGRGGGADLV
LISRKDEYKNRNLGAVPVIVELKVDQSASTTDVKKEVESNIFSNVRTFSD
EAIVACASLWIEKSKTRLGESELIAKRYNIPKVEGLITLLLENLNDSDLD
NIIKEELERLHHSIPSMGKINGNPNYLSRLILGEILTNNDKLRKFAFIYK
DKEKFTTFLLQRDGVDQIIILNVVESVLIKLEKTVTFNDKVVISDSGYIY
QIDIGINPEQTKKFFDEKSSTEKYLQLLQIRKWNFQEYKNEQKYNGELIE
FGSVNYVNLNKEVELKVIEDEQLTNNLNEALFPIRELITLVDGNQDWSKI
SKNKAGNIDKTTKSNLMAVIHGLFIGKQNIAKKSNQIIKVLAKTSLAYKR
DLDLAISIASKDRNTYQENEVVAICTSEPDTFIERGLRSLIQKMRAKVQV
VSFNVKANSKNDLINIKSKNYDIEHTSIGEEDIIEDFFDAIYEGKVNELV
DLLKNRVTSERIKLRKREYNDAFKLTLELEEFEMSDLLLKYAIDDKQKHL
MLNGADKNGITLLDNAAENDKWNIVEYLINKGGESDYVLEPYMFRLIEEQ
KWEKVKFLIDKEVDVNATDGKDMTPLHHAVRDNNAKMVSFLIDKGADVDA
EDKGKNTPLHYACRDGNYDIVYLLLDRDAKSISNEDGKEPDYYLDYDNPE
SEQIRELFEERFSNSDSQNPSSDEESHAPEEYHSSSDEIDSDDSQAYFEH
LFNKEPDPDSDLTPSSSDELDIEYDSVRCLSSDSGRKKREAESECLFTWE
DVDEFNEEKDKKRDLSKIKIDSERFVSYIKDLPEGKQSQLIQLAGETGNA
QGLVSKLISNQKIMNHLSRVGRVSGMTMHGMMAKNVLADFLNSNYQGVAI
NVGFIAGGQGFAKVAEAASLKGLKLAEEGKLLVGQSLKAASPFLARGTSA

FVVYDLVNQIKAFKNGTEEALVGVVGDSIYLGVDAAEIGVEVAEAFGVLE
GVSSVTGPIGATIGAVVFVGTDIYMAVKRVDKIDQIIHLTGDEKFVEGLR
AFIGMKPEQYIEELMEKKQLYNQLVKQGLGYLKQHSDIQSYVFPTTDKLD
SKVLLDRKRTGIRWSRARPDDLNEGRVFCLPQGNDEPAPDYGSYLCENAI
GIFANKTGDHTLINLGEGKDYAKGFLNSPNIFVVNNGSKEYYGGNKDDIF
VLQAEYIKGHLSGEGGINTLDTTLFALQEEQLNIQLEIGEIADYFRDNWL
KICEINKVIGRANKAETITVSCDGCNSNVRLIDGQSGNEEIKDRINIIDD
SCDYQMQIVVRPNTAVYNRALKGSFDYLVPLNSSGSAEVSFIYGPERFNV
NNTFWFAYQAVDIKSIDVKYINVFNRTEHEVKFNFVESNKEFNVTISYSE
NPAYRLGKSGEIRIGNKGNLYMLESSDKSSEEVIRDYLPVANRVSKMSFF
IQSVLSNETVVIGSGNHEVIHSNPAYRSHLVGNGGENVYVIDSETTEVII
HDVDEENSIDTIDLRNVVRQVRGELSNQDNFQLKVLKSANDLLLEATVVE
VKPTEDSSVSKMRKHEYCTVRLKDGVNWYNKTHVIVDNAPMKINLDNNEW
SLKPQPLMFERDKEIIVVTNQDIEQGSEIITPRKGGNYKFVRSNDNDLMI
TNAFDLTITKNDLCTITLSKFYETPRMKTLFIKFADKEIILKEHQEEIST
ARDVNVVKKEHKDQVYNDVFNPEVMMLSDQPVTHRHRHSRHREQARHRRS
TTSSSTRPTGWINDLFGWVKSSISGLLSSKPESTKSPISQVDAKMDVNGT
IILLDMLIRKVTGQKYVSTADQSISPLEAQGYALNITKGFEKVLNKTAIK
SGISLTNLNFDPVVVQSAIIGKIINGKFSEIAKTLYSFAKEACPEFKQTD
KFLDHLKSSLKEEKETVMLQQKVEKPSKDLSQEVSRKVELSKKPNTFLNG
TSVVRGISRAIN

>WP1291_wPip_WO5_CAQ55399__HP
MQGTRAQVIETLKSRIRGGGTPTAQGILQQINAILRRNNAREIEDVHDLL
ALDFATDNQNYRYWLQTHDMFFAARQYTFLDNQSHSTNDHYGFEITSVGV
DGNQNDPTGRGLLSSHITNFKQKVDSGEKDRLIAIINVGNRHWVTLVIVH
QNGNYYGYYADSLGPDSGIDNNIRGALRECDINDDNVHNISVHQQTDGHN
CGIWVYENARDINQAIDQALQGNNNFGEKGEGIIGYIRGLLSAGIGNDTR
QPRRNEQYFEDRRRDISQLLQNDPNLPSRRSDLIQAHPGIQHEIDPLLLQ
FLGLQYPQRGGGGALQLGGERVISIDFGNPQSALDKIDGVSRVYNHSNSR
GSR

>wPip_WO5_CAQ55400__HP_WP1292
MQNIINIIRDFLGRDHPNRDRNYFQNMRVGISQLFRNDPGFQGVQLRAYI
QNRGQIDPLLRTYLELGYHRVGGGCGPVEAPISLSVSDYSNFGGLPQSSL
EGPEASRVSDLSRGKGGR

>wPip_WO5_CAQ55401_ankyrin_repe
MVETKEFVEHIIKLTNRQSDQTNQQEPKWKPSTGQRWSSTDDQELEITQL
PTSSKDDYYELIKQSLFWINMNLMDNTSNWSLLEKEWVIKAHRSELTKNQ
EKLIQKLFHIINSRDTSTLQQFLQKTKSNPDLKTVLDLKVGASELTLLHI
LPTILYSFDSCTLVDCLLKAGAEPNIQDYKGKTPLHYYAATYPVEKKYVD
SLVSYGADLNIKDNNGKTPLQIAIDKGTLDVAECFFNLEQKKLYEELNKL
IEGTSKLENGSSKYNVQGVEDLKKFLDKNKNNEDLKMILSIRDEENNLEI
LKDYMYTEVINLLSATQSQLMVTEQETEETYVPHQCLLDICLTDQLRQLN
QFVSEIPKAANIAELQKVVDKAIMSGVGLNLTKDGGQCFTDYILERIGQL
EKNHEVASNIVCTLISKGARLSDWNSLNVIDEIELEFKEHKANMIKAHEQ
CVDHTHKFLKIAKNATNGKLNCAKMDNATFYLEYSEDSIIDVAQITDGTR
DLGLTHGDVKCGRNIVKIGNSEVEIKTEDGIRNYTDLTEGSSIVLTFYTS
LGELEVRLCPHEENKNWIKVEVSEEGLLLLDEIDSYNEAIEDCNEGIENY
EKIGQNCLLGGLSVIEAIDRGVFARSGGLMHPEVISESNNKWTEREELRR
TSDPRREVSR

>wPip_WO5_CAQ55403_putative_pha
MFFQDKNKPSVILTKSKVMNMDITTSLLGDVSNLIDRAKNHLSVQFNSTL
VLLNWQIGSRIDQDILKHKRADYGKQIISQLAKELQIKYGRGFDRASLFR
MVQFSKFFPDQEIVATLSQQLSWSHFVEIIAISDELKRNYYIEMCRIERW
SVRTCT

>wPip_WO5_CAQ55404_Putative_pha
MNKSVVRVGDHCAEATSHFCISGSNNVFVNGKSVCRKGDNFTESRVLTEG
SKTVFTNGYGIGRVGDSISCGSKVMSGSNNVFSG

>wPip_WO5_CAQ55405_baseplate_as
MLENNFAIAELQRKVANIIRIGVVKEVDYEKAKVRVKIGELLTDFLPWIT
SRAGEERSWLPPSINEQVVILSPLGELSLGVVLAGIYQQKYSAPENKKEV
SSLTFQDGTKLSYDKDKHHLEIEVVDKITLKAGESSIEMTKSGIKLKADK
INLN

>wPip_WO5_CAQ55406_Putative_pha
MNFKDLHNAICTTLKREIPAIQTCEIYPSIRKELLAPALFVELVSLESGK
DPGTEELALRAKFEARIVVDGTVEDSSVVVRSLAAEVARVVNKNTWNVKN
ISPAEFLSAEPDGFRPELDAYLVWMVDWSHQLHLGKSVWEEGKIKPHKIA
VGEMYVGK

>wPip_WO5_CAQ55407_Putative_min
MSVHIEVEVIENVNAKRKKIELAIVRALNRTALWLKLKTAKEISEEKKSL
IRKRLRIFKAKTSRLEVLIRANLYDIRASTIGKIQKTRRGSKVGKHEFIG
GFAAVMPKGNSGIFKREGRTALPIKEVKLPLEPEASRIIGNLVNYEVEEV
FTKFFERDITESV

>wPip_WO5_CAQ55408_Putative_pha
MQENIKRLLKDCFAHLGEVALYKSKDKSYMVQVLKQQPDKLYEIGEGQFV
EETLALEVSAFDVLKPIVGDVFVIGDRRYKVHSPPLRDKSGITWKIKASG
V

>wPip_WO5_CAQ55409_Putative_pha
MQNPFTNTAFSMTALTNAMNILPINYGRVENLNLFPSRSVRFRHITIEEH
NGVLSLLPTQVPGAPATVGKRGKRKVRTFTIPHIPHDDVVLPEEVQGIRA
FGSESELKALADVITDHLQLMRNKHAITLEHLRMGALKGIILDADGSELL
NLYNEFEITPKVVNFALGTATTDVKRKCMEVLRHIEDNLSGEYMTGVHAL
VNPEFFDALTSHSKVKEAYERWQEGAALRNDMRSGFTFCGITFEEYRGQA
TDPEGTVRRFIEKDTGHCFPLGTASTFTTYFAPADFNETVNTLGQPLYAK
QEPRRFDRGTDLHTQSNPLPMCHRPGTLVKVVAA

>wPip_WO5_CAQ55410_putative_pha
MTCITEQNNLGDLLKYEASSLYSRDQITVAKGQNLKLGTVVGYDTKDNLI
KALNPTATDGTQTAIGVITSDVNAKENSKGVIIARGAMLADYAVVWPAGI
TEEQKAAAIKQLEGRGIIVRKGV

>wPip_WO5_CAQ55411_Putative_min
MEILQTNWLCRPMMIEQKSFDLLSLYNGKQPIFKKIKHAVNQNIEKTAVI
EIHGILTKKPGAFDVFLGMTSYEQIEEQITQALADSSIETIILEIDSPGG
EVNGIFDLADFIYESRAKKRIIAIANDDAYSAAYAIASSAEKVFVSRTSG
VGSIGVIASHIDQSGFDEKCGIKYTTVFAGSRKNDLNPHEPMTSESLESL
QKEVDRLYEMFVQLIARNRNLSIEAIKSTEAGLYFGEKAVEIGLADGVTT
FFEFINNHRSVSMTTNELTEENCRREILEIIRLCNISKMPEKIGEFIEQG
SSVEQAREVLMELLAERTKKTEILSAIPQNAGEELMMQVAKSRCI

>wPip_WO5_CAQ55412_Putative
MLLKSFKQLFSKPKIKSSAWDAAGSGRRFFHFQPELGSINNLLSQSLETL
RSRSRDMVRKNPYAANIIDTIVSNSIGTGIKPKSKAKDGEFRKKVQELWL
KWTDEADSCGISDFYGLQALVCRSMIEGGECFVRLRTRKLEDRFSVPLQL
QVLESEHLDNKTNQTLGNGNVIRNGIEFNRLGQREAYYLFREHPGEGSFG
ESVRVPANDVLHIYKPLRPGQIRGEPWLSSILLKLYELDQYDDAELVRKK
TAAMFAGFITRLDPEANILGEAQANEQGVALSGLEPGTMQLLDPGEDIKF
SEPSDVGGSYEAFMRQQLRAIAIGTGITYEQLTGDLTGVNYSSIRAGLIE
FRRRCAMLQHNIMVFQFCRPVWNRWLELAVLCGELRIDEKVAKAAKEEVK
WIPQGFDWVDPLKDQQAQQMAVRNGFKSRSEVVSEMGYDVEEIDQEIAED
QKRADSLNLFFDSDVRTQTK

>wPip_WO5_CAQ55413_phage_unchar
MTKIIAKEYTEFLEQLKEQIAISRYKAARTVNKELLLLYHYIGTQILEKQ
KSQGWGSKVIEQLSRDLKAEFPEMKGFSTRNLKYMRQFAGEYQDIEFVQQ
LVAQLPWGHNVFLMDLVRDKEARLFYIKEAIEHGWSRNIMVMQIELGLHK
RQGKAITNFKEKLPSPQSDLAHYTLKDPYIFDFLSIGKDAHEREVEKGLV
GHMEKFLLELGEGFAFVGRQFHLDVGDEDFFVDLLFYHLKLRCFIVIELK
DNKFKPEYAGKMNFYLSAVDDLLKHETDQPSVGLILCKSKNDVLAKYTLK
DMTKPIGLAEYRITESLPENIKTALPTIEELEAELSKVSDKEK

>wPip_WO5_CAQ55414_putative_pha
MYSEEYLTQVEKAIQKLQSGERVVSIAYGDHVVRYGEVQINDLLNLRQRI
KAELKVAGMRPKRKIVFSTSKGII

>wPip_WO5_CAQ55415_Phage_termin
MIYARAFSEGLRPDPELKVSEWANEYRVLAPTAASEPGKWRTERTPYLKE

IMDSLSPSSPAEKVVFMKGAQIGGTEAGNNWIGYIIDQTPGPMLVVQPTV
EMGKRWSKGRFAPLIESTPCLKSKVKDPRSRDSGNTVQSKEFPGGIVVIT
GANSSVGLRSMPVKYLFLDEIDAYPGDSGGEGDPVLLSIARTNTFARRKI
FLVSTPTIHGISRIEKEFEATDKRYFFVPCPHCNYYQVLKWSQIKWENND
SRTAHYVCTECSGKIENHQKTEMLERGEWRATEAKEGEKKGFHLSSLYSP
VGWYSWQQAVEDFLHAKESEQLLKVWINTTLGETWVDKGEVPDWKQLFNR
REFFPVGTVPKGEVVLTAGVDVQKDRLEVEVVAWGKSRESWSIDYRVFEG
DTGGREVWGKLSELLNHHFIGENGLEYMISMMAVDAGYATQEVYNWVRGH
QGSGRVMAVKGVNKALVPLSSPSRVDITVGGQKLKRGIKLWPVGVSILKS
ELFQLLNVLKDGEEAPAGYCHFPEYAPEYFKQLTAEQLVSKVVKGYTKQE
WQKVRERNEVLDCRIYARAASIALGIDRWPESKWNSLSGKMESKKPKKVI
QSKWLGNQNVQ

>wPip_WO5_CAQ55416_ankyrin_repe
MSAHLSLELIKCLINQPGLDVNVRGLNGKTPLHCAIEFDELSMVDLLLTK
KNINPFVEDNDGKTSLDYAKEGKKAEILKALINNKYGSEQDSLLHLAAMV
GEVNAVRYLINRGIDVNSRNAIFHTPLHLAAGIGHVEVVKILVREGNAEI
EVFDARNQTPMHYAVNNKKLEIVKLLLELGADVNSARVGQNSMKLSPIHI
AVSNTNYDERDLCLDILKCLIREPNAQVNLQDYENKTPLHYAERLKTIEV
LLTREDIDPLVKDDSGKTPFDYAKPEIKKALISNKYGSEKNSLLHLAAQR
GEIELVESILKEEIDIDISNNKSLSPIYLAAEKGHLHVVKLLLKKGANYT
PVLHLAIKSNNLELLKVLFNEKNGALLCRDTVVNFPTLHNKYIAQREIAD
KRMKKHNNIICICITVSAVAMAAYIGLTAATISSAIIFATITGIFALVVA
IMVSEMNKRYIEKEFQKKMFMELEECSSTVNDVAIVSRCR

>wPip_WO5_CAQ55417_Putative_pha
MKLITQTEWAREQGFSKQYVCYLVKKGIVELENGLINREQANEAVAAIRD
PSQPLRRKNYSENGEKLSTMLLKTRIKNEMERGKLLEAKAKAEIGELVAV
EEVKNEAFNVARVVRNNLLNIPNRVSALLASLSDTEKIHMELTEEITNSL
EELSNTKF

>wPip_WO5_CAQ55418_Phage_relate
MNLAIHYYPTRDLVEYERNPRKNDDVVNRMCASIREFGFRIPIVAKSDGT
VVDGHLRLKAARKLGMESIPVVLSDNLNEPQTKAFRLLANQSANWAKWDD
ELLKVEIQELEDLQFDLKMTGFELEKVQHFLDDLDSEKEDLSDLVVDDKK
VEITKPGDLWILGDHRIYCGDSSVVESYKALLDDKMADITVCDPPYNVDY
GSSQEREDKKILNDNQGEKYELFLYDICSHILAYTKGAIYICISSSEFST
LQKAFEEAGGKWSTFIIWAKNHFTLGRSDYQRQYEAMLYGWKSGNKREWH
GGRNQSDLWFYDKPTHNTLHPTMKPVELMERAIVNSSRPGDIVLDPFSGS
GSTLIACERTGRICRTIELDSKFVDVTIKRWQVYTGREAILSGTGKTFPE
IQEEKKEQKK

>wPip_WO5_CAQ55419_putative_pha
MSILTLDLGKQTGWAILTDGVIESGSESFHTSRFSGGGMCFLNFRNWLNS
LKEISVVYFEEVRRHLGTDAAHCYGGFLAVLSAWCEEHHVPYKGVNVKTI
KRFIAGNGNASKSEVIEAVKEKGFLPRDDNESDALALIFYVMNFSKDFNT
LKIP

>wPip_WO5_CAQ55420_putative_pha
MRKQKKSLISFNGEFMNISCDFNNTISQSYLPSSEETANIKLRLLADLRS
CLSYLLPNGVFHGNRFYVGDVHGNRGKSLVVELTGSKAGLWKDFATNQGG
DIIDLWAEVHSKTEFHEIMASIAEWLGYNKKYTEKQYIDEDFEKFITYSW
NYYDENGQVIVKVYRSDFPGKKKVYKPFNVKTLSYKAPDTRPLYNIPGIL
KSDKIILVEGEKCAEALIEKGITATTTMSGANADADKTDWSQLKGKHIVI
WPDNDEPGKQYAENATTKLLSLGIASLSIVKIPEGKPKGWDAADYVQENT
NVSDFIENNLKKVIIRPPLNILDWSADRFVGPVPEQKFLVEGIFPLGVTS
IVAAMGDTGKGMLLLDLALKVASDTDQICGFGSPVTEHGSVVIFSAEDDA
GEVHRRLERLDPECKRLKFKDKLFIVPLPNVRGSLTILRSIRGKIIEVSP
EFESIIKQLDKIKDLKLVVFDPLASFIHADINADPAVGDYLMCLLSDLAC
STGASIVTAHHLRKPKGEKPILTAEQARDAIRGTSALVNGVRCSYAFWPV
EGTAKPEIFQSIGEAPRQNALFYGAVVKANGLADRTVRTYLRNQETGLLE
DITVQLKGKNASEKDLKMYLVDAIARSAVAGHPFTHTGNTGVYKQRHRLP
DILHGLGRDRLERIVQELLQTKKLVKGMSTGSREDKWLDVREGPFARGVG
KFIHGAEGIIPNLIANKTN

>wPip_WO5_CAQ55421_Putative_pha
MVYKRSLVQGSIIDLKVVESFKNAIVSHLFFFIQFKYLSNIFVVFLLSLT
FYTVCNLLLFIITFIYYSYILYNNIRVLGT

>wPip_WO5_CAQ55422_putative_pha
MQSKNKYPGIDPSIIKQIRKRARQIKSYKCCVNSDIEDIKQQLLCETWSD
LSQYNGEESGRNAFIHSVIKNHAISLLRKQSCAKRGSKAGVAFVDITDLD
DVIDENSNFADKVAASVDINEAISKLPKLWQDICYQLESHSISEIAEIHG
ISRTTIYDIREQIRAKLAHLKIYL

>wPip_WO5_CAQ55423_putative_pha
MTLKIINSEQRVKMVTGVKIVIFGPYGIGKTSLLKTLNEPTLCLDFEAGL
LAVQDWQGDSISIRTWNQARDIACLIGGPNPALKSDSAYSQRHYEHVFSK
YKDLEFSKYSCIFIDSITVASRLCLLWAKMQPEAFSDRNGKQDMRAAYGL
LAQEMMAWLNQFQHIPNKDIITVGTLGQYLDDFNRPTWLPQCEGAKTASE
IPGIVDEVISMVSIKKDDGTEVRSFVCHTINSWGYPAKDRSGCLDMVEEP
HLGKLLTKIKTKTFSTSTQFMPHN

>wPip_WO5_CAQ55424_putative_pha
MLQDFLTTGQKIPFFSMKEYLNDQSSIPKDIVSPRILTQRSLLVLGGPPK
IGKSDFLISWLVHMAAGVSFLGMTPSRPLKIFYMQTEIEYEYMKERLQCL
QLDPELLAIAANNLIITPKVHLSFCHEEINYIKEIAKERFKPDILAVDPL
RNIFNSEYGNENDNSAMLFFLQKTLEKLRNAINPNAGIILTHHTKKLSKK
MLEEDPFQGLSGAGSLRGFYTTGMVMVAYDEESTRRQIVFELRNGERVPN
KLIDKINGSWQLLDQWNN

>wPip_WO5_CAQ55425_putative_pha
MLQDFLTDFNNAKLQSSLIPKGTIVKVKMAIKPGGYENWFTKSYDTGSIY
LNAEFTVIEGPYAKCKIYQIIGIKSGKSEGKEDTWGESGRSMIRSILESA
RNIHTNDTSEKAVLARKINSIADLNGLEFTAKVGIEANNTYGDRNRIATV
ITPEQEKNSDADWIPF

>wPip_WO5_CAQ55426_putative_pha
MEFCITESRQEIKMLLAHNIKECLSYLLPGGKFGYGFYSGNINKDHLMVV
AEGKQTGTWYNCDKKTNGDIIELWSIITNKTDFSEITSLITKWLSERIIA
KYSYLDKNNEAIAYVYLYKNNYRHIWNLRTSRNGLKPLYNIPGIIKSDEV
IIVKGENRADSLIKRGFTATTAIDDDIEKTDWSMLTDKHIIIWPEKDEQY
VKKVAVELDNLGMSCENPESLGISSENRESLSVLSEKKAFPASHYLNDKS
PSPEDIILPRILTPGGLLLLGGAPKVGKTDLLLSWLAHLSAGLPFLSMVP
IRPLKIFYLQTDLEYPYIKERLQQLKFDDKSLELISKNLIITPKTSLLLN
SQGVEEVKDIIAERLDVKTVDIIAIDTLRGVFDFNQYKGENSNSSMFCFL
KDRVEKLRSITNPSCGIILTHNTNKVSKKSLVEEPFQNFSGASALRSFYT
SGIMMIKPNGEQNTRQLIFELRNGKSILPMQIEKVNDSWQTVSEKPTVKQ
RRKKSYDIILRLINERAAKGKFYTKSQFCYLFEDTFGLKSCHSIRRYVNA
LVASGYIKFYEKEIPERVKVMKVAEHLTRRDI

>wPip_WO5_CAQ55427_putative_pha
MDLPFCQKQNPTFYRQIVTNLLRWSDSYDTPSRDYLEVAQYLSSLGFVNL
REYYFIICANDEDEFDFHVINPFCNNRLEIVSDYDEDYDNPIMCDLCERD
ILPDTYKKQRYFSLEVKVNHLKVIEWFEKQLASLKITCNKVATGVYYVIV
DTSLISLIIPECCPDNSYSAVDKLKTTPTALITFNKESLKPPLNLHIVPI
ADLICEDQSLNEVLHQTVEKGVPELLPNVSFQAFNCYSYIPLQQTKSTPA
EKTFQLHIKGNDICVNGIGVIETQSKSGRIFFIFLDQFFHDFKSGISPEQ
YKTLNVGEIANRLENIHDVEQQIRKPINRMQKTIAEKLAITLGLNVKKDD
IIQTLPWSGIGTKEYGYRLNPFTIVLKK

>wPip_WO5_CAQ55428_putative_pha
MIANQEFWEDDLEVPATELLLSFQNPDLCKSWINSLNNKQLNVILKQHFN
YQQHLFDNSPYYDYRSVQQKRKFLIDSNLDYLPNYYLISYFSRLKSDSAI
IEIAKPILSGDVKYDKKSILFTLFLIDHNLLKHVFLFNKVQKRSFSSFIL
NNPPRQRQSSFKDFLSKEVLREILTQHDSLANDNFESKFKGFFYYQDRIY
LFIRRASDSDLVLSSNQVIHGYKPDWIILDFSLNSNQVNLCTKNLKRGLE
IANSIASRYFQRECSFINLRNQNKVAQVRTFLTDCVHELIKDIKLIELKF
TSPEPSTYFTLNTNSIEKWLKVLEPSIGSIIYNISLVQHIKVIFRNKKVT
LSFHANTDYIAINYSEHVLDKKEREDFKLMFSNTYGLTILSKAKSNFLQA
NSY

>wPip_WO5_CAQ55429_putative_pha
MSILTQSGRAAIAASIKEQSLHLAWGSGDANWESSHQVEKVFVEDEIALD
HHTIKDVRVFTGQTTYQPSIDYIVDSSSGVIKRTENSSITANSAVTIEYT
ESTPPELITSEKLLNELGRRTADEVLFCTGDENGELITPSGRFKPSNVPT

NNLYLKFTFDFTNAANQVIRELGVMVGTKVKEKLPPGQRYFEPQDIEDPG
ILLVLEHTVPLIRTSATRETFSFVVTF

>wPip_WO5_CAQ55430_putative_pha
MTLNAYYNRFNPDKEYEKSLFLAGRGLQSAELNETQEYALSKLKGIGDAI
FRDGDVITGSNCIIDRETGKVTLEGGKIYLRGAVRKVEKEEFTIPLSTIV
RIGVYYVESTITELEDENLRDPAIGTRNYQEVGAARLKVSTIWSYQAEGV
TTNASGEFYPIYNIENGVLIEHSPPPQANIVTTALARYDKEANGSYVVNG
LEVMFLQKKEGEGGKKIFVINEGKAHVDGYEIELPHSIRVSFDEDPDIKS
VESEPHTFQPNSQRVMELKVNDFPISEIKKVDITVQKTITVTHGSYSGAI
DPIPDSAVLEIIQVKQGNVIYENSIDYKLNAGNVDWSLPGKEPAPGSSYQ
ITYRCRTHVSPEDISEQGCKVKGAVDNSLVLIDYTWKMPRYDLITIDSKG
VVRRIKGISHPWRPSMPRAPSGQLLLCYIHQTWKKGEGVKIVNNAIHAVP
MNELEAMKKGINDLYALVAYERLRSDANSREPTTKKGVFVDSFFDDDMRD
QGISQSAAIVNKELILPIDVEIIDVEKSTKPYLLPYELEPVLEQLLQTKG
EKINPYQAFDPVPAKVTMNKNIDHWTEVTTNWKSPVTREFNTRETTELLS
STSYEAEFMREAVQNFEIEGFGPSEKLKEVKFDGIVIQPTA

>wPip_WO5_CAQ55431_Putative_pha
MLTANNQGKLQGKVKVPANIPAGTKLVQFYGDKGSYGEATYTGKKTITIE
ERRRVIAARRVDPLAQTFTLNESRHIGGVELWFVNKGKKRVVVQIRETAV
GVPSQTVIAESYIEPKDIKIDGTATRIEWSPVFCHAGEEYAIVLLTDDSG
TAVKIAELGKYDAVNSRWVTSQPYQVGVLLSSSNASTWTPHQNLDLTFRL
LAAKFSENSHVIDLGKVTATNTSDLIVLTNVEKVAFDTNVEFILTDEEGK
ENFLSDNLPLALRERLSGKLTVKANLKGSKEKSPVLYPGLQLVLGNLLES
GDYVTRSITAGANTKITITYDALIPGTADVKAYVQKNVDWQLVNLTSGKP
IGENLVERTHVLSNFNGNDTRIKLVLSGTVVYRPKVKNLRIIIT

>wPip_WO5_CAQ55432_Putative_pha
MSDDKTSRGYSLPHPENIAVQDVVRIRTTIEKIDEDITEREDEHNQLKNN
FKRFRFETFLNFWE

>wPip_WO5_CAQ55433_Putative_pha
MKEAIYQRIKDLAANSTPDQLAYLAKSLELIADKKAISDIVQMAKIKEII
DALQKRLKDLAENSTPDQLAYLAKALESIIDKSAVSEIVQMTDGKLKELL
DSAKKHLTDLDNKKASSLAVISESEKQLLKRIDEKGTTNLSLLDTRKNAN
IAAINSVGNDHKDGLKGLVNNFRTVNDVPSGSSIMKEVRNRHMIEPGALP
FLFGVLSRKNNYFGHGTFTTELGQWSSDVTKTDYMLQLLAGTHTYESYVS
FYRPRQLSFLEGSKGTFIYGESYPRFMYDGFSEQFNMLKYPYAALGLIFV
KNTSNVDISKTLNFVGSAMWYKDEVNYGGAGLFIGTPDKTNTRKSEISRI
TWTRIYQHEINSPEFIASGNILIPAGKTVAVLLYTSSNLHSEGERVGETP
EFFASEHTYSTIYGQFIQWGIYNFRSNFLTTGLEVDVERTLRAWQCPGLD
ATHKIWN

>wPip_WO5_CAQ55434_Putative_pha
MPIYIRFEHNKQVETTTLESKPIGNDWYEAPKNFDWQKSYQLTEGGEIAQ
RSQEDIELELLQNAKFSALSNLRAYYDNYTNQYTGVSHQKSKSYQIQEKA
AKSILADQDEKDKEIIEPLAKVRGITVIEMARIIEEKAKKAVKEIIKCEE
LEDITKKKIAEAKSKNELENLLNDFKQKIQRNG

>wPip_WO5_CAQ55435_Phage_major_
MAEEFLHGVNVIEVTSGAKTVRTAKSSVIGVIGTAPEADGQKFPLNKPVL
IAGSLKEAAKLGKSGSLPSAVNGIFSQIGVTVIVIRVEESENSDPKLKEE
ETLKNIIGGVDKETGEYQGIEAFLNSESIVHVAPRILIAPQFTHQLPESK
NPVVAALIGVAEKLRSIIVADGPNTNDEEVIKWRKSVGSSRVYVVDPWVK
VFIEGKEEILPVSPFVAGLIAKVDSEQGFWHSPSNKEINGIVGTSRPIDF
TLGNTNCRANHLNENEVTTIIHQNGYRLWGNRTCSNDSKWAFLSVRRTAD
LINDSLLRAHLWAVDRNITKTYIDDVIEGVNSYLANLKAQGAIISGKCYA
TPELNTPANIASGKVYFDFEFTPPYPAEQITFRSHLVSGTIL

>wPip_W05_CAQ55436_phage_tail_t
MLPKILKNFNVFVDGRGYAGKIDEITLPKLTIKTEEYRAGGMDIPINIDM
GMEKLEAEFTFAEYDTELFRLFGLINGNSVSLTLRGGMQGSGSNDIEAVV
INLRGVFREFDFGSWKPAEKATLKCTVAAHYYKLTIGGNELIEIDAENMV
RKINGVDQMALLQTILGI

>wPip_WO5_CAQ55437_Putative_pha
MPSGIKPYNIDYSESVIKKDIPALPAKVKLMIKKAIMERLTVDPIGLGKP

LKHNLSGQRSLRVSTYRILYYIDVPEHTVVITAIEHRKDSYQN

>wPip_WO5_CAQ55438__Putative_ph
MQAITLNNPITVDGISVSELTVRRPKVRDYLAIERLNGSDLSKEVTLTAN
LTSVAKEAIEELDIADYVKVQEVLKDFFSPIIQKT

>wPip_WO5_CAQ55439__Putative_ph
MRLEILVLGSIIGGGVEHILDMEISEFISWSKSAKELKCQRYL

>wPip_WO5_CAQ55440_phage_tail_t
MSTLSIKIGAVLDGSFNTVIKGSSSQLSHLGENIRRLDSSLKSVSKFKQL
GSDVLTSKRSWKDFEDQVKSLAKQMKEVEKPSKTLKAEFDRAKFSATKAK
EAYLKKRDVLHSFNEEVRKSGRNIKSLVSDQYKLGSSIEVLKGKYGKLGS
AIRSHQSFLASKSHFKSQIIETIGLGLTLAAPIKVAIDFESAMADVTKVV
DFKKGTDEATKFAKKLKEMSRTIPLSAAELAQIAASGGQLGIKKEDLFMF
TETVAKMSTAFDMSAEQAGDSIAKLSNVYGIDVSKMEHVGNVINHLSDNT
AAKARDMVETLAIVGGTAKQFGLDIEKTSSLANAFISLGKTPEKAATAIN
ALLSKLQTAEEQGGDFKAALEQMGITAEEIVQRISENGEEALLYFFQTLK
KMDNQERSTILMKLFGQEYQDDIALLAGSFNKYEDAIRLLADTEEYKSSL
QKEFQNRVDTTASKLRLLRNAIAEVGMNLGSVMLPTLKSIAEFLQEKTRS
IALFAEKYPTLTKAIMGTIAALISLKILVVGLGYGFTFLASTVLGLRAKI
IATFSFLSATVFPAVITGLRAVTLAVMSNPIGFLIASLVTGAALVVTNWQ
KVKDFFSSFWKSLIKPIGEAFSWIGESVFGKVLGNSTLKELKKRKTEVKA
VHTPLKSNIFNSGNPLLGNSIIKEFSKRNKNIFRVKSLIEEKKSTENDKI
LADFARSKFENKEQNKTQNITNNYTISIKAEPNQDVRSLADEVIKRIREK
SRDVLFDTVETFY

>wPip_WO5_CAQ55441_phage_tail_p
MLSLGPYKFAPTSLKYSRENRWSTIECIENMPLLQNIGQGVENIDLEGTI
YLHNLNGLNQLKSVKEAEKPHILVDSLGNILGQFVITRLEEKQMYFLPNG
LPRKVEFSLSLKSYR

>wPip_WO5_CAQ55442_phage_tail_p
MTVYYVSKENEMLDLICWKHYGFTDGVVELVLAENLGLAEYGSFLPAGLK
IKLPTIKKIVQKSKLKVWE

>wPip_WO5_CAQ55443_phage_late_c
MQPDFIIDKCESIKDRVISLHLTDESGIIDDVVEVCVDYRDEDIDIPNEL
NIALGYKEIGIFPMGIYTVNEVTIQGPPKTLLIKAHATNLRISLKAKVSK
EWHQITIENLVKEIAQKHGYGHKVAEEFKNVLIPHINQADESDISLLTKI
ATEREAMAKLAGGYILFISKNMAKSATGKALGTTTIRPQDTINWKVHFTV
RDKYNSVVAKWHSYEKGETIKETVGSGEPSYIMLEMYSNAESALSAANAK
LKQLKRNNAVLDITIPGNPELFAEAKLNLIAFNQAVDGEWIVNRAEHTLN
SSGYLTMLSASLSK

>wPip_WO5_CAQ55444_ankyrin_repe
MSQLKCNEKEKIHFVWFIILMVCVVITYCYQKSKATDNYNKTLQAAASNC
NLEIVKLLVKDMAPNLSETALHCAARKGCLDIIRFLILEEKVNINVIDRN
AFKRTALHHAAGEGHLGIVRFLLEKGANPNIKDNDGKGARKIAVMASRHD
KNKPYREIIKLLANAEEQHKSK

>wPip_WO5_CAQ55445__HP_WP1338
MQKQKIPITLIITLLIQTVAFIWWLSKLDSRVQFHDKLIEYGLMEKVLVL
EERVKNLSEELDEFKDNLKSGV

>wPip_WO5_CAQ55446_Putative
MYFAMVRYLLILLTCSLLSGCVSCPKATVCFTPGENCTNLIIDSVGHAKK
SVLVQAYQFTSKPIAESLVQAKKRGVDIKVILDESQTSSKHSVINELFEH
KIPIWIDFKPAIAHSKVIIIDEQKIITGSFNFSDAAQQRNAENLLIITGD
SPLVEQYVKNWKNRQSQSKHYTPDFKLSLNSSNSSERFFTLSSSTNTFSI
KPYSISLS

>wPip_WO5_CAQ55447_patatin
MAKYILSVDGGGIRGIIPAIILAEIEKRTRRTIAEIFDLMAGTSTGGIIV
AGLCKSNKLQYSANDLVELYQEYGAYIFQSSFWRKSIASWLSGSQYSYRN
MEFILNKYFGESTMADVASNLLLTSYDIHNSCEFFFKSWKEKNIKLKDAL
RATTAAPTYFTPKRLKISQTERVLIDGGVFANNPAACAYASAKRLFPNDE
IILLSIGTGRTDRSIKYANSRKFGKIGWVKPLLNVMFASGLDCVDYQLEQ

VIDDKYIRIQSQLKIASPDMDNITSKNIKSLQQEAKAMIEDNQKVIEKFC
IDILNI

>wPip_WO5_CAQ55448_putative

MFISVKIVNNNTKNISPARYKIAQNVRSWRLKQKYALKDLVDKTGIKYHT
LLRYEQGACGIPIEKLKVIAEGLSIHVRNLFPRRKALRESGCFDESKIEW
MYNFIAKTRGRKAIYALTKSIGAEEENNVKAARIKIAKNILKAGFTNDII
YRAVGLSTEEYSDKESIHYTSQNGGQEIKKWRIIRGYTQEELAKKLNVGP
SQIHHYEQGSVKILSERLWEIAEKLSVNAEDLIREKDCYEDDEGEKELLS
LARELKKINNQESRDELDIWVEFLSQRKQVYKEKIYKAEAIKVANNLRML
DVSIDAISKITDLSIEELENYAFKT

>wPip_WO5_CAQ55449_HP_WP1342
MTTKKTVSFDDTDGNTYELIIDSKKLPKGLYSAIRRTSKKNQQLILVDET
DPKIFDDKSRFENPEGVPSPSDVSGKSINLGEGREVYNKKSSNRQNPFIP
ALSKIEPSASENAELRWFKSRVVKAKSVDELNKTIDQALSTGIRINAYSE
GQKSFADTVISKMPLIKCKENEKEDIICELMLNGAIFSYDLLQDKGISEM
HNKLYQEIRPQIDEWLKELREIGESAIENEGIIEDVEMDNQTFFMKFSEN
SKVNIARVLEGTKNLGLTTGEVKLGGDVIEIGDSKVEVRSEKEGERNYVD
VSDNSAFEITFPTSIGSLKIVIYHNAENDHEIEVRVADKEMWSELQKKGE
NIGKGCFFGGMTVTEAVEKGSFPRSGKWSKEKVKEEIKAISNNEALSWVD
RVCGGSKETSRKL

>wPip_WO5_CAQ55450_Putative
MNNGKKLTEEIERVLQSGGGSALFDREIVSVLLGAVHDRKQAQDVTKTLM
DNCPGIGEILGREIDDLKMIEGMTEHAAAAIECVKEAYKRALREGLKRGP
VMDSQEKLIEYVRVNIGFSAKEAVLIIYLDKQNRLIRDEVYFGTIDEVHL
SERKVVKKALSMEAASIILSHNHPGGSLEPSEDDKVMTRELVSACQGVGI
ELEDHIIMTDKGYCSFKERELL

>wPip_WO5_CAQ55452_conserved_HP
MALSKFLDARNDYAFKRIFGTEKNKDILIHFLNDILGFTGLAAIHDVEFL
ATILDPEIAAKKQSIVDVLCKDSQGSRYIIEMQFTKTKGFEKRAQYYAAK
AYSSQADQGDNYHNLKEIIFIAVADCIIFPDKADYKSNHVILDQNSFEHD
LKDFYFVFIELPKFTKTKEDQLENIVEKWCYFFRYAAETREEDLDKIVGS
DVIIKRAYEEMNKFNWSEEELLAYEQMKKRIMDEIAAFAQKFDEGLRVGQ
EKGRQEGIQIGHEKGKAEGEKQAKIAVAKNSLKASVSIDVIAEITGLSLD
DIKKLI

>989wPip_WO4_CAQ55453_HP_WP1346
MKDELLAGEYPIWPLDTNLSILGNYSQADFFLSDLQRKGQENVIMRNREG
LNIYEFNQRNGLEHLTQVPNGAKLGNSEEKLFFPDLTGQSYKDIVALNSS
GLFIYQYGQNNYRSIHYNPLFSKIKGWNTEHINSVQFEDIDLDGRQDMLF
TGPRGIDILTFDNHTSQWRSLLDNSQLTISERHSNVIKVISSNPHVIEHP
IIFTQYKDQLRWANIVEAEKSLEEEVGIQERNETSSQSVPPIVPQLLQLQ
SEEEKPILLLREQLDCSSVINAVDKNTGKPKFKLPLIDLSNLSSDIKLDF
FYDSNSKASDILGLGWSLPRNFIMMDHQSSIFPEDEKYYVILQDLPQQLI
FDSSNSTDTVYCFKLSTDQPDLQIHYHKNEERWEINNSGIKQIYGRTDSV
RSDSINWELAWENWRSVGSSSTGQKKLATGWYLAEVRDENDNVVRYTYDI
INVSVPNGESFTQEIYLKTVGDNQGNEVIFNYSDKEESEYNLLPLIDKDG
NLNTRKVETRYLSGYKVSTPGYQQDVDFVYRVEDGKRLLTEIRQRGDITN
QPILQFTYKEFSAYMLEKVTLPIGASVEFSYRSIGTASHITEELGRRYDV
QKSHRVDYSNDDVLISYIDEQGQVALRMFNQEMIEELYSSVSSSISSRFP
LLGRGLVKAHEIVRAEDFFAVILYYNTDKELCLFRKEEGKWLPTPKYYSF
NEDTMIRFGADFTVVASTNSSVEIIDWNKASKEWTKKPFFNKDNTSLLLA
ASGRMFLIYDDQGLLIGYRNHDNEWQSKVIKEIPGVASSIQKTLEKFNLS
NNVHKHLLSYFRNNALQLNNNLLLLSTWEAEGPQLYSIINLFVLDRTYQI
VQEQQYRIDQDNINVFSREVEDSSNNKYTLAYRINGDKYKFTVKDFSGPI
NDELENSKKEALDEIEEKKCKALAQIDAARGDLQIKAEEARIDLQNRAYE
VRRDFQDNATQARASLDARVKTEKDVVSSRIRKVKTDLENKVKGVHDDIQ
SAVQKAKADMQVKVEKVKSEAFAEITKQGRKFLAEVQSKEERDEIHRLMQ
KEKDSVLSKLSEEVSKVNAQLARESNEAYRKLEEKVNETYEQLKKSTNEA
YQKFEESKNEVYGQLERRLREALEQLEQKIHEAYERLEEQISQAYQQLEE
GEKEVYKNVKDSRVRVYEQIEDIKAKICKNVASLWEKEFFLLDSVKYSAT
LNSQIAVCSHRKFTFTGDKWKEGTISETELRQENFSIPLGNKLVLDKENS
RSSLKLYSQGNNREKQGNSLLDLEVKQLNQTSIRNMYPAYLAYQEKDKQV
GVVEFSSDGTIDKTYKLPQEEKLSSWSSYQTLVTHVNNLDSGESNTLVFR

QQLGIRRLLPNPVITKTAVTLENGSKRITGYEYDVSSAKALGNSVYYEKT
SIIPGNNKTFFGWIEEMKDFDNYSNGEKKVFNAQEVLIKTLKLKEEQAST
NDTIINRENLYLNTTLLDKTGKLEVAQFFPYEIADEEVSHYGFENYEINK
IGKNVTLPEKRWLFNETDVVKQGFSFTGQSYLSLSRSTLEGTFQPNNQNQ
EYVASCWMRPQSRVFGLGAVTPYLKAVVYAENGDEIFGLLSEVKFQSGDW
FYLEVVIDLLHAKQIYKGLQKSSNSTLTEQPDINLKTSIIVSPGTNTTID
VDHVRFSPLNSHSKVNVYDTKTKQVREVIQANGLVEKHVYDEYQNEIASI
NEYGQLEKLSSYTKASSIGRVVQLKSSIQIQPETGFYEDFSPYSFKERWK
IDNADVWKISLAQLQHFTNHLHSLQLNPYDINSASHGMRVCFSLQSESSI
IKFNDDLKLMRTGNRANIIFPGKRLSVPLNGEILVAVEHGRLLVWIDGGL
YFDGLSNGSLFNLGVSGKVRISDVMVFSEPSIQVTYFNALDEKLQEVVLK
GENTAIVTEYLYDELGRQTITTQPARIERSSSQSVLAYYPNFVTNSNPYS
ANSVWKIEQLQGDVGRIVGEHSYSQTKYDNNPLDERCAVGLPGREFSVSG
PYAKRFASSSENLFINNLFPTSQNYSYQVEYKPGGVEDISVFDNKNNRVA
QYTRVPGSRDLLSTYEYDENDKLVKSLPPLYHEKVRTFHKLNSQLQSLST
EEKALQDFLGAHTSYDGKGNAIIKTTPDSGKVEHLYDNNGLLKFAVHYSQ
LNGEEVENVIYFDYDQLGRLISTGKLTSPLPKKEELPTLAISSNNIEEYQ
QFYHSDFEREPILRGKVRRTITFNNKEPLIEESILNIDKETLSKRILIPI
KDNDEPLLIAINKRYTAGKLREIEYPIDVQGNSFKLTYSYNKLGKVTGIG
VPGKQNLFVNFAHNSAGQVISEQHLPESARNFTRQYSYNAPGFLTKLEDK
FLTEKVYYTDRSYGGHGYGDGTVTRTEFKATWHEHCDNRELGLNEQSFVS
ENITPKESELCFHKLKETGYLNEHNHQAKAYYPALEPKLPIVCSDGITGR
QIAKTLGEKGFPTEYGHSYDYGNHQELTKAKYFVGNEAPLPLQPDTFAKE
IRGINSTVSHDIWQILKSAGYLREDNAKVDISLSHAKRGKPFIRPTLFSD
LKSINANYGGYKLPLERLLATAFSQGQNLSLLKSNLQNAFIKWNGGVGSN
ELQQTASKIIEMLERKQHLDNPLNEEFSNLLRKYESFIPDIVRLLSEHFA
RQLGEAEFDVESYDIDANGNHKHYYTGFDRYELSYSNNTNQVNSVKFKSY
SSSKTEQKFAIKHDSRGNIIQALHKGIEQIDYDSVSNHATKIRLTDGRAL
IFYYDAQGERILKRVTNSQGETTKEIHYIRDEFGRALVERQITYISQDLP
PDILITAYIYGPRGLLGFIRRDEFYSVITDHEGSIRLVVKGDEVVAAYDY
LPYGNLMREYGGNPEAHIAYRYTGQELDEETGLYNYHARFYDPSIGRFYQ
IDPQGQYFSPYKYAGNSPVSIIDPDGEFAFLIPLIIAGAVAGGYLGGAAA
NKEWNPGKWKWDSGKTWLGIVGGGVGGALLPIGFGASVSAFTSIGLSSTA
SIAATTSLGGAGIYFSTAAANKEWDPSKWDFSSPETWNAAFQGFAIGSGA
VGGIRSAGTFYKGLSSTWGKGAFIVGSTGTGGGLFVLNGFAANWDFSKPG
VYFGFVDAITGAPDLAMFLRGAGRFLGKNGRNIVSTFDSFKSLNNQKGRY
FGGTVFSNLNQAFQGIDASEALKRLASKQTWSNTATFLGDLSSHQFSKSV
GKIAGGVAMAGSSAYLMGSAVSENGWDLTSAYTYHAIINGMLTGGQLASS
GRAYYRGKIVKNQLKLPKEMERIAQNYAKEALTIYQEILNKGGQIIIGGH
ISSKVITIDGKDFTILDPGNKINKKSIKDIESIDRDNGVKDWMIKNGEEK
FIVATTKNGWVTIDPKGEIQGISRLHPIRTGQDPISYSRKVKVQIKKGEV
PNTAISVAVTENLAVVTLSGTDSPGLVIFIDRKQGVTYTKPLARNKEFAG
KEVTIDQSIENAINALRESGEDFPPWLPSNCAEPSALVALSSSKEAFPQS
LNSRSYEIDDIDFLATYKVPKPFPDNIDFKKPLDPKFPDLVPFERCNYCK
VTTGSVRNVITDPAWLKKIDDLIGLNEKLDYSKQRLYFLIPYHDITNRIV
KMNTTNNGNRSLSNRQKRSSDKRIMVEVEHSAIDHSINETGRWSEPNTVK
NKKITSSATSSSARTGSKINNLFSWIKSSIGGLLSSKSVGTSNTTSSISQ
VDAQIDVNSTIMLLDLLIRKVTGRKYISTADQSISPLEAQGYALNITKGF
EKVVEQAGLKSGVSMHRLNIDYMGMQKEITRKVMSGKFNEISGILSSYVE
KALPGREAGYPGKLSEKRFNEFMIKFNKGLDVTLNQSIEHNGDGRLEVDV
VKEQKTNSGPQSYLSNASVQGHLTQNKVKLIS

>WD0251_AAS13993_conserved_HP
MLFNSIALVVSLLLFFYIIVKKLSEKKANLEGNLCKLEQELQETKQTLLT
KNEEIVDLRVKKAELEVTLQKEREEKKKEIELLTKAEERLTNTFKALSLD
ALQTNNNNFLNLAKEVIDSKLKETESDFKKRQATINEVVTPIKEKLEKFD
NEIRELEKERVGAYEGLKEQIGALMNQTSSLANALRKPHIRGKWGEMQLK
RVVEMAGMIEYCDFFTQPSVIDKNEDNLLRPDLIIKMPSGKQIIIDAKVP
LDSYMDAISQNDLQIQKEKLKNHSLAIKKHINDLGKKEYWNQFENTPELV
VLFLTGEGVFSAALEYEPALIEIGVEKKVIIATPITLIALLRAIAYGWKQ
EMIAENAKKISELGHILYERICTMGENFDNLRRSLKSAVDHYNKTAGSLE
ARVFPAAREFNKLGIHAKNKSLSAAKELESLPRSLHTEELKVD

>WD0254_wMel_WOA_AAS13996_trans
MEVYVFVSASNVSYGIGQKIENCRLMRGHTQIGLAGQVGLTYQEVNSYEN
GYTPIPIGVLYVIARVLSVNAVDLLPKLITVREYKDEDEEILYLTKIYEN
QKLGKIVPSLVRFVHISEKINQEEARLEVAKNLVKEGVSVDIISQATGLS
IYEYDSTEREICTDSIYYRIGQRIREWRLIKRYTQEDLANKVGLTLKEIQ

GTFKEVCQTEKKVIN

>WD0255_wMel_WOA_AAS13997_trans
MVVFVEKSLDCKVGEKVKNWRLERGYTQKDLAEKIGVKYWVILQYEKGNR
GISIKRLYAIAEALSVSITNLIPASKEKIGFKNEEGEILNLVREYKKIND
HELRRMFCLLTKFVQVSEKSSRKSEKIKIANGLVKAGISVDIVSKTIGLS
ADECIEEKVGSIYYKIGKKIKEWRLVREYTQKDLGEKMSTTRHEVSNYEQ
GRTAVPLDKLYEMAEALSINITDLLIEKDEGSRVENELPNLIKEYKEIES
QELRNALIKSLFEGIRICEEKVREIERIKVAKDLVKGGISIDIILQAVGL
PVDIVLDR

>WD0256_wMel_WOA_AAS13998_HP
MSNKIIVPFDDQEGGTYELNIDLDRLSKGEVLNAIVGIGRTKEQPTFVSK
INKAKKPEEIAPFPGREVRENKINYGKGFDYVYGTKPVSDQEDKNPFASA
LKKIKPSAEKLSWFKSAVVRAKSPNELHKVVDEALAAGARLNACNDGEWS
LAEYVILGTHFHKFDKADRKKLIRKLMLNGAEFHDTLLENKLIGEIYNEL
QPEVQPQIDERLEELEKAGESAVQEGELIDVEIDNTTSYIEFSGDSKVEV
AKILEANRKLGSNILKIGNDAVEVKSEKGGIRNYIDMSDESSIILQFPTS
IGQLNIILYHDVKKYDQVQVRVENKEMWAELQKRGEEIGKNCLFGGVKLQ
EAVEKGNFTRCGIWNEKYAIKEISNDEVLSSWVNRVCGGSKETFREL

>WD0257_wMel_WOA_AAS13999_DNA_r
MKIIYFDQKCRLKGEEVFTGTVDKVPFYIREVTRKALIRKATSIIISHNH
PEGRLKPSDEDEAVTKDLAKACQTIGIRLLDHIIITSVGYFSFKEQGLL

>WD0259_wMel_WOA_AAS14000_HP
MDENKIVFYTTPGGNIEIEVLCRDENLWLTQKRMAELFDVQLPAISKHIK
NIFESGELQEKEVVSTFEVTAADGKNYPTQLYNLDTIIAVGYRVNSKKAT
LFRVWATKVLKDFIIKGFALDSERLKNGKKFGKDYFNELLEKIREIRASE
RRFYQKITDIYAECSADYDPNSEITKQFYAKVQNKLYWAIYGLTAAELIC
SRVDHKKPHMGLTTWKDGPGKKIHKSDVSIAKNYLTEEELSELNHIVSMY
LDYAELQAKKNRLMKMQDWAEKLDAFLLFNDYEVLKDAGKVSAEVAKALA
EGEYEKYRVIQDKLHESDFDELIKASKESEKITI

>WD0261_wMel_WOA_AAS14001_HP
MSILTLDLGKQTGWAILTDGVIQSGSESFHGNRFSGGGMALLHRYL

>WD0262_wMel_WOA_AAS14002_CHP_i
MRERFSDIEAVYFEEVRRHLGTDAAHCYGGFLAHLTAWCEENNIPYQGVP
VKTIKRFITGKGNASKSEVIEAVKGKGFIPQDDNEGDALALMFYVNNFSK
DFNMLDSA

>WD0263_wMel_WOA_AAS14003_proph
MNLATHYYPIENLVEYDRNPRKNDDVVNRMCASIREFGFCIPIVAKSNGT
VVDGYLRLKAARKLGMESIPVVLSDNLSEAQTKAFRLLANQSANWAKWDD
DLLKLEIQELEDLQFDLKMTGFELEKVQRFLDDLDGENSEKEDFSDLAGD
SKKVKITKPGDLWILGGHRIYCGDSCLVESFKAVLDDKMADITVCDPPYN
VAYGDSQEREDKKILNDDQGEKYELFLYDICSHVLAYTKGAIYICASSSE
LATLQKVFEEAGGRWSTFIIWAKNHFTLGRSDYQRQYETILYGWKNGNKR
EWHGGRNQSDLWFYDKPTYNSLHPTMKPVELMERAIVNSSRPGDIVLDPF
SGSGSTLIACERTGRICRTIELDPTFVDVTIKRWQVYTGREAILSNTGKT
FAQIQEEKR

>WD0264_wMel_WOA_AAS14004_cHP
MEGGKITQTEWARELGVSKQYVCYLVKKGIVELEDGLIDREQANEAVAAI
RDPSQPLRRKNPEGEEVGNNKLSMMLLKTRIKNEMERGRLLEAKAKAEIG
ELISVEEVKTEAFNVARVVRNNLLNIPDRVSALLASINDTEKIHETLTEE
IRTALEELTQSVF

>WD0265_wMel_WOA_AAS14005._prop
MIYSSSFYTGLKPDPLLKVSEWADRNRQLSTIASSEPGKWRTERTPYLKE
IMDSLSSSSPAEKVVFMKGAQIGGTEAGNNWIGYIIDQTPGPMLVVQPTV
EMGKRWSKGRFAPLIEDMPCLRSKIKDPRSRDSGNTVQSKEFPGGTVVVT
GANSPVGLRSMPVRYLFLDEVDAYPGDSGGEGDPVLLSIARTNTFTRRKI
FLVSTPTVHGISRIEKEFESSDKRYFFVPCPHCDHYQVLKWSQIKWKDKD
PSTAHYVCIEYSGKIENHQKTEMLARGEWRATREEGKKEAKIGFHLSSLY
SPVGWYSWKQAVEDYLHAKENEQLLKVWINTTLGETWVDKGEVPDWKQLF
ERRENFPIGMVPKGGKIVLTAGVDVQKDRLEAEVVAWGMGRESWSIDYQV

LEGDTGSGEVWGKLSELLNHHFIGENGLEYMISMMAVDAGYATQEVYNWV
RSHQGSGRVMAVKGVNKALVPLNSPSRVDVTVGGQKLKRGIKLWPVGVSI
LKSELFQLLNVLTEGAPGYCHFPEYPPEYFKQLTAEQLITKVVKGYTKQE
WQKIRDRNEVLDCRVYARAASIALGIDRWPESKWESLVGEKAKKSKKVRR
SQWLSEKS

>WD0266_wMel_WOA_AAS14006_HP
MMYNQEYLTQVKEAIKKLQSGERVVSIAYGDHVVRYGEVQINDLLSLRQR
IKAELKVAGVKRKIVISTNKGIDK

>WD0267_wMel_WOA_AAS14007_HP
MIGNVERLQRELLEEMGSISLGSSNTRALEIVNQLGEDHINDTIMTLDGR
QFTALDCAIMFDSQGNNPRINERPRINNNAFRELEGAIRSAGGRTSEELG
RRQDIPYDRESFNEQVSTFLTDVTIEHVMAGRLHLMV

>WD0268_wMel_WOA_AAS14008_HP
MVNSRVNVTFNPKTSQHLIELAKATKQSVQKLTEKLIKEAIELEAEDIAF
SKIVRELDADDSEEVEDSEDIWK

>WD0269_wMel_WOA_AAS14009_cHP
MKTSGNKRYTIKYLKHVLKRNLPSLPEAIKPKIKDAIREYLATDPIGNGV
LLRNRLKGHRRIRVDDYRVVYRVNTAERKVTIVSIGHRDNIYKQAILDLL
KH

>WD0273_wMel_WOA_AAS14010_CHP
MSCITEQNNLGDLLKYEASNLYSRDQITVTKGQNLSLGTVVGCNIEDNVI
KIINPTATDGTQTAIGVIASDVNVKENTKAVIITRIAMLADHAVVWPANI
TEEQKAAAIKQLEARGIIIRKGV

>WD0274_wMel_WOA_AAS14011_CHP
MQNPFTNSAFSMTELTKAINILPINYGRTESLNLFPSRSVRFRHITIEEQ
NGVLSLLPTQVPGAPATVGKRGKRKIRTFTIPHIPHDDVVLPEEVQGIRA
FGSENELKALANVVTDHLELMRNKHAITLEHLRMGALKGIILDADGSELL
NLYNEFEITPKVVNFALGIATTDVKRKCMEVLRHIEDNLSGEYMTGVHAL
VSPEFFDALTSHSKVKEAYERWQEGAALRNDMRSGFTFCGITFEEYRGQA
TDPEGTVRRFIEKDTGHCFPLGTASTFTTYFAPADFNETVNTLGQPLYAK
QEPRRFDRGTDLHTQSNPLPMCHRPGILVKLAA

>WD0275_wMel_WOA_AAS14012_CHP_d
MFRDIKRLFADCFAHLGQQALYESKDKSYMVQILKQQPDKLYEIGEGQFV
GEILILEVSIFDVLQPVVDTVLQVNKKITKIVQAVS

>WD0277_wMel_WOA_AAS14014_CHP_t
MLKLKTSDAFDKIQGDIFIIDKRKYKVYSPPLRDNSGMLWKIQASGV

>WD0278_wMel_WOA_AAS14015_proph
MRINIEVTGSIIQSIDAERKKVEKATVRALNKTALWVRSQTVKQVSEEKQ
IPKKAMRKKLSVDKANRKRLWSVVKLSSQWIGVAKLGSIKQTKIGAKVGS
RTYEGAFIATMKNGHIGIFRRRYTTSLPIDEIKINTHARETMKELVSNEV
ERVFEKYFDHELNFI

>WD0279_wMel_WOA_AAS14016_CHP
MFWRDLHQKICKTLKEEIPAIQTCEVYPAVRREIIAPAAFVELASLEPGK
DPGTEELALRARFEVRVVVDGTVEDSQVVVRSLAAEIARVVNKNTWDVEN
VSAGEFISAGGDDFKPELDAYLVWLVEWVHELHTGQSMWLETGIIPHTIS
VGENVRA

>WD0281_wMel_WOA_AAS14017_HP
MAKAVVCIGDYCSGIPAHVCMSGSSDVFVNGRSVCRKGDILTLGEKLTQG
SNSVFVNDIGITRTGDLVSCGFHVMGDSKNVFAE

>WD0282_wMel_WOA_AAS14018_proph
MNASTGKELEGLNHLKQSIVDILTTPIGSRVMRRDYGSRLLELVDKPINR
DLTLEIYAAVAEALQKLEQRFKLEKVKITEVKEGKVNISLDGIYLPNGKN
IHFDGIMV

>WD0283_wMel_WOA_AAS14019_proph
MEQPNIIEPLNFEEIFSRMKEELVSRDESFTALVESDPAIKILEVAAWRE

LLLRERINEAARSNLLKFARGEELDNLAEFYGAERQDGEEDERFRKRIKA
KIKGWSTGGSKEHYRYHALSADRRVKDALVESKVPGSVEISILSTELSTN
GIASEELLDIVRKRVTRDDIRVLTDTITVVGCNIIEIDIHSKISIKRPDI
IETVKKKFIEKFEATKRLGWKVTKSWIIANLFVEGVENVELIEPRKDVVV
LGNECVCLGKLEIELMGKS

>WD0284_wMel_WOA_AAS14020_CHP
MRLFRQARNRINGQKLMLLPPNSTKEERAIVEAIDYKVDPSRIKGFKFNS
KDEILPWIIEEYGLGEILRWVKDKGKAIREGIKFQRLRGTPASLKIALKW
ANIEDITIIEEPPGKHFFELQVGIRDVPNDFFVDAVVELAKLSLPARSRL
MRLFNDHYNIGRFILDESFFGSLLSDYSGTKIEKDGPVLSFGRVNFFRSS
GPVVRIIENYLRNHYEQALSNDIYRLDVAILGETEPHTKNYNGIYERNHV
WYNFKALYPLPQSLLPAIKFAKAQIVLSDSWNLGEINACFPVTSIEEEGD
KFLLGSSKLSEQLWNLKYKPVLERFSVTHHYKVEDFTNQKVIRFGLAEHN
VHFESDLDSEQKDSIHELERYILVFYPGVLTWHEHRHLNRSWEEKQVVCL
MS

>WD0285_wMel_WOA_AAS14021_proph
MWISQEERDDFNELFQELLAGKLSKKHINRKNENGETMLHRAAKMSTRKK
VNWLIWEGADVDARDNEGYTPLHSAALGMRLENVKELIEARADINATEED
GNTALHLACMVGGKKIVEELIKAGAEINLVSISGFSPMYYASDEETREVL
KKKGGKIVNKQRELMEKISKVSEKQVVNGGKKLNDVEERAVDVCRLFVRK

>WD0286_wMel_WOA_AAS14022_proph
MVKFSKKEREEFNKSWKEVLDNSIENINKKDTKGRTILHYAVGMPDPKKV
KLLIKKGADVDAADAGKYRPLHLAVMGQRLENTKELIKAGVDVNAVERSS
KFAALHLACMVAEIKIVEELVKAGGNVEQKDKFGKTPMDYVRNNKEIKEV
LENVKMANKQREFIERIRVVSESTAAGVIVAKEEVKELETMDEKVL

>WD0287_wMel_WOA_AAS14023_CHP
MEGIEKKVLNLEKKALEELRKIWRKVFVEEAPRNSKKYLIPRLAYRLQEK
AYGEISRKGAKRLKYLADRLEQGKRISSDKLPAVGTEWILERGEETHAVM
VTDKGLIYREEFFTSLSAVAGKIMGMSYNGPLLFGMRDKKVS

>WD0288_wMel_WOA_AAS14024_proph
MLKEVRCAIYTRKSNEDGLEQKFNSLDAQRVACKKYIKSREGWVALAKRY
DDGGYSGSNLNRPAIKELFEDVKAGEVDCVVVYTLDRLSRETKDCIEVTS
FFRRHRISFVAVTQIFDNNTPMGKFVQTVLSGAAQLEREMIVERVKNKIA
TSKEEGLWMGGNVPLGYDVKEKELIINEKEAKVIKHIFERYMELRSMAEL
ARELNREGYRTKAKSDIFKKATVRRIITNPIYMGKIRHYEKQYKGKHEAI
IEEEKWQKAQELIKNQPYRGKKYEEALLKGMIRCRCCKVNMTLTYSKKEN
KRYRYYICNNHLRGKGCKSMNRTVVAGEVEKEVMKRAECLYENWEKGAKK
EKWENLSFGKQKEAVKKLIKTVWVREDGIEVCSESEEKFIPMNLKKKGNK
CTVVEPEGKTNNALLKAVVRAHLWKRQLEEGKYRSVKELSIKINIGTRRI
QQILRLNYLAPKIKEDIVNGRQPSNLRLVDLREIPMLWSEQMEKFYKLAS

>WD0289_wMel_WOA_AAS14025_HP
MPVKKKERTDKLCGLTEKEKGKFDTLKQEIENLGKKKVLYVLEKDEK

>WD0290_wMel_WOA_AAS14026_HP
MFWRRTKNENSIAEKFNDDTLQKILTTKIPYSEEGRKGVFTLPCYAISLK
NNQAFETFLQEAAKRKFLKDLLNDELNGEILGVQKTTYTVLAYCISEKNS
KAVRAVLEVSKENGILKEILDQKMIKEKANSQKISYTLIDYANKQNCKEI
VATLKEFKESLNIEVKPVSGNAETSCEDINKDCEDLEIHEPMLASSEEAK
NILTKDDIDQTQSSSISTTTFTEVVKRDQTQSCSHSKSRSAENGKSLTEI
KHKRKLIKTDTESREKLTDNLTWESKATAQLRGDINKDSELIFETSEKAQ
NALTEEEPDIPSPTELYFDSKGNSVDEKSSKKAIQPIVAGVVGAVLLASG
VALYIMKMPVIAAVVGIAGLVCLSFALYNVLKPSTKLEKVESVEQPIQSS
LNLA

>WD0291_wMel_WOA_AAS14027_proph
MHTSKPTAAEKDLNSKLFYAVEQNNLDKVKELIRNGADIHAREISSKKTM
HIAVKKGNKNIVEFFLNEGISVNDTNNSGWTPLHYAAFGGELEIAKLLVA
NGANVRAENAYGQKPIDLIHYGKDDGYKGIMELLLNKGGGKVNDIDKEGW
TLLHYAAFNGNLETVKFLIDKGASIHTKNNGRETPLDLAREGGSTEVVNM
LSSINTKVTDVSVSQLSIPRKRTV

>WD0292_wMel_WOA_AAS14028_proph

MLTRDISRPEQVFSGAEISEKKELEEKSNTRQGQSEIGGSFDDQYELIIE
YLLQIPPSNDRLEILRKQGHSQSDIDEMFSQGDSWYTQSTISSFRSYNSP
PSLMDSKYSLSKQVSVWSALDHNLTESQKKLNIELLNVLKYLEWHDEGAL
FDYDNGQVDELEEFLKNNRGNPDLKAVLNVKRGESGSTVLHAIAGAHIGA
SYRQEDRTINLLLEAGASPNIQDDKGETPLHRASAMGYDKNIYSLLRGNA
DPNICDGQGKTPQQTAVDNHNYHVERCFFTDNQKKLRKELNNILNRHRYK
EGNLNYIVTLTLFQFFEDLERFLHEHKNNKDLKVVLNTRNIAGKSEVLEH
VKNAFSGFQAADEIKKLLLEAGAKEFTYERKKCLPKSGVLWGNIIPAQEE
KLSRSLGILSEIQDINQLEKFVKIAIKSGVRLNYRFTVPSPFEGYSFTDY
VIKRISELEKCPKVASGIICQLVSKGAVFGSPESIDVIDELGLECKDHKA
NMIKAFEGYINDAHRFIKVAKSATTSKLNDIAIGNTTLYLEYSEESKIDI
AKITDGARSLWLNYENAGYERNIVKIGESEVEIITQNGKRHYTDLTANSN
IALIFCTSFGELEVRLYSDKQNENRIRVEARDQGMLKKLKDCGEEIGKNC
SLGYHSVYDAIERGYFEKPASSSRIVQEEKLQNGKWADQVRYTRKNGAQI
R

>WD0293_wMel_WOA_AAS14029_HP
MSKLWVHGRKKLTLPKRRRKKNFHNKSVFLEGAVMTQGNWDSDKNFAG

>WD0294_wMel_WOA_AAS14030_ankyr
MKDLVNVPRNRQGWTSLHYAVKNGNVGKINDLIKGGKNVDAQDEQGWTPL
HLAATGSYTKVVNAQMYGDDIHARETGSEEPIYIKACKNIIESFLDKLLN
IKVVGALIKGKAEINAKDNQGMAPLHWAVKVGHINVVNGLIKGKAEINAK
DNQGRTPLHWAALIDRTSAVKALIKGKAEINAKDNQERTPLHLSIQIGRT
DVVNTLIDKKAEINAKDRQGRTPLHWAASKGGIEVVNALIEKGADVNAVN
KYGDAPLRFAARDGHIDIVKALIQGGANVNARNSDGTPLHTAYGHEEIVK
LLIEKGADVNAVNSNGDTPLRFADRNGHIDTVKALINYVTKLEAADLYVS
QKNLEEKNRLIGDLHDLHSSYPQHLQNCKKEVKKIEKESQELHSFLKKSD
INELISVWERNADIQNQIDNHDNLKEQYPEYAHILINKANEVKKEIFLHN
HQPLIDALSAHYKCDIKTMTFAGIENFFKVVHRDDFKEKLGNGGITLKNF
VDLKNVEKRDDVPKLGVIAFKHGCQLSEPRVTRVIDQALSL

>WD0295_wMel_WOA_AAS14031_HP
MKDYHQLRPLHLRHQYSITKEAKCDLSYDNASDNSSQSDEIGYNKIGATS
GASKPSSWIDAVKGVFQFISSPLRAALPSFSENSSSRSGTAGSSSNHSDT
VRNPNIQYTSQFSREVCASNNASLMFFLLQAFLDKKCPLPKFSDVNPVKA
LFYTSDIIKLFKEVLEKQAESKGGIKMHRLDINFEKLFEKINEKIRSGEF
NEISGILKSYAEEALPGREPGDPGRLSPKKFDKFMAEFNRRIDPVINQLM
QQMLSRLEVSDVKEQQISLEPKSYLNNTSVQCHLTQARGQGKVLIP

>WD0296_wMel_WOA_AAS14032_CHP
MTFSKFLDPKNDVSFKRIFGTEKNRDILIHFLNDILGFTGKNEIKDIEFL
STIQDLDIAAKKQSIVDVLCRDSTGAQYICEMQVAKTKGFEKRAQYYAAK
AYSRQADKGDQYHNLKEIIFIAIADCVLFPNKSEYKSKHTIRDEDTNEHD
LKDFYFIFIELPKFPKNKEDQLENIVEKWVYFFRYADETSEEELEKIIGS
DVIIKKAYEELNRFNWSEKEFIAYGQEIKRILDEQAVLAQKLDDATEKGR
EEGKEEGIQIGHEKGRKAEKIEVAKNSLKAGVSIDVIAQITGLSIDEIQK
LRN

>WD0297_wMel_WOA_AAS14033_HP
MNNKNKSKDRKKEIEFRIDLTQKKWRERKEFFRVK

>WD0299_wMel_WOA_AAS14034_HP
MVGYLLCTKISAFFLTLSRKSGEKVWNVFHKENDESKCSGMQ

>WD0300_wMel_WOA_AAS14035_cytoc
MVKLFALLIIFYSNISVASAPTSWQFGFPAPATEVMEAVVRSHSFVMLVM
VTIMLFVWALLAYIAFRFRKSKVTNISKTTHSVPLEIIWFVIPTIIVGIL
AFENAKLLKLQEEIPKADITLKVIGHQWYWSYQYPEYQGVSFDSYIKGKE
DFIEGDLKLFSVDNNIILPVNTNVRLQVTAGDVIHSWGVPAFGVKIDAIP
GRLNEAWFNIKKPGVYYGQCYELCGQGHGFMPIVVEAVSKEDFNKWIENR
KLVS

>WD0581_wMel_WOB_AAS14286_HP
MSILTQSGRAAIAASIKKQSIHLAWGTGDSSWESSHKVEKTFVKGEIKFD
HQPIFDRQPIKDVKVFTGQTTYQPSIDYTVNGSTGVIKLTENSSIPVSDK
VTVEYSESTPPELITSEKLLNELGRRTADEVLFCTGDENGELITPSGRFR
PSNVPTNNLYLKFTFDFTDAANQVIRELGVMVGTKIKEELPEGQRYFEPQ

DVEEHGILLILEHTVPLIRTAATRETFSFVVTF

>WD0582_wMel_WOB_AAS14287_regul
MDIYCSQRNGHYKLLGKKEQLRTQLLSNIRSCLSYLLPRGTFRGDKFYVG
DLQGNKGQSMIVELTGSKAGLWHDFSTGEGGDIFDLWAAVTGKNQFTDTI
EDIAKWIGYSEKNNTLGQPTASWNYYDESDQVIVTVYRYNTDSGKRYLPF
DVKRSSFTLPETRPLYNIPGIVKSDKVILVEGEKCADALIEQGMTATTAM
LGANAPIEKTDWSPLKGKHVIIWPDNDEPGKQYAEKVVKKLTFLGVLSLT
LLEIPENKPKGWDSADGIEEKMNIPEFIENNSKKIIVKQLLNIQEWSVER
FIGPVPEQKFLVEGLFPLGVTSILAAMGDTGKGMLLLDLALKVASRKDQP
CGFGPLVTEHGSVVIFSAEDDMSEIHRRLERLDSQCERLKYKDRLFIVPL
PNVSGSLTIIKSVSGKVIETSPEFELITKQLNKIKNLKLIVFDPLASFVH
ADLNTDPAVGDYLISLLSDLAGSTGASIITAHHMRKPKSEKPISTVEQAR
DAIRGTSALVNGVRCSYAFWSVEDAAKPTIFKSIGESVRQNALFYGAIVK
ANGLADRTLRTYLRNEETGLLEDITERLRIKNMNEKDSKQHLITAITRAA
ISGHPFTHTGSTGVYKQRHRLPEEFHSIGRDRMERIVQELLQTRKLVKGM
ATGSKEDKWLDIPSGPFARGVGKFSCGAELFS

>WD0583_wMel_WOB_AAS14288_HP
MPQDIIGPRILTPGGLLVIGGTPKIGKSHFLLSLLVHLVAGRSFLGMKPT
RPLKIFYLQNEMEYDYIRERIQQLNKLPHLATENLVVTNKMNLTLNEEGI
EKIKNMVEEKFDLVVIDSPYNYVNYGLQHAIEKLRSKINTMAGIIITNHT
KKVSTSTLEKNPFQALVGANALRSFYTSGIVIFQPNKRTNILQVVYELRN
GRSIPTKFINKINGCWKSANVIART

>WD0584_wMel_WOB_AAS14289_HP
MVAILKYDEGKLWAAVITRAIQDAAGKNPKLKKEACQWIRSRSFETVCEL
ANLDFKRLKNMLNKFGINKENFMDFSSKNALKIKSLLINNIKDCLSYLLP
EGKSYQNKFYIGSRNGRRKCRKLVQPDRRKERKYNRPLDFS

>WD0585_wMel_WOB_AS14290_CHP
MLQDFLTDFNNAKLQSSLIPKGTIVKVKMAIKPGGYENWFTKNYTTGSIY
LNVEFTVTEGPYAKRKIFQIIGIKNGKAEGEEDIWGESGRSMLRSILESS
RNIHANDTSEKAVTARRINSIADFNDLEFTAKVGVETNNTYGDRNRITTV
ITPELQKNFQDDWLPF

>WD0586_wMel_WOB_AAS14291_HP
MNLLVSGQPLTHHTKKLSKKMLEEDPFQGLSGAGSLRGFYSTGMVMFAHD
EESSARQIVFELRNGERVPNKLVDKINGHWQLME

>WD0589_wMel_WOB_AAS14294_HP
MLQNFLTIGQKIPFFSVKEYLDDRSPIPEDIISPRILTKRGLLVLGGPPK
IGKSDFLISWLVHMAAGVPFLGMVSNRPLKIFYMQTEIEYMKERLQQLDN
ELLNVAANNLIITPKVHLSFNHDEISGIKEIVNERFKPDVLAIDPLRNIF
SSEYGNENDNSAMLFFLQKTLEKLRSAVNPDACIVLRGCPETSKRL

>WD0590_wMel_WOB_AAS14295_CHP
MKIMNSNERLKVQTGIKMVIFGPYGIGKTSLLKTINEPTLCLDFEAGLLA
VQDWQGDSVSIRTGNEARDIACLIGGPNPALKSDSAYSQRHYEHVSSKYK
ELASEFLKYRCVFINSITVASRLCLLWSKMQPEAFSERSGKQDMRAAYGL
LAQEMMAWLNQFQHIPNKDIITVGTLGQYLDDFNRSTWLPQCEGAKTASE
IPGIVDEVISMVGIKKDDGTEVRSFVCQTINSWGYPAKDRSGCLNMVEEP
HLGKLLTKIKTKAFAIQPVTPNI

>WD0591_wMel_WOB_AAS14296_CHP
MKNSYQGIDSTIVKHVRFQASRLKSKSCFINESLEDIEQELFCQVWQYLN
QHDEKRSSFSTFVARLANYCARNMLRNQLCLKRNITFDDINDDIPDHRNS
EIEAIIRTDINNIISTLSEKDSNLCQLLKVFTITEVSVITKIPKTTIYRT
LERIRNKFSVLEKL

>WD0594_wMel_WOB_AAS14297_proph
MNLAIHYYPVENLVEYKRNPRKNDDVVNRMYASIREFGFRIPIVAKSDGT
VVDGHLRLKAARKLGMESIPVVLSDNLNEPQTKAFRLLANQSANWAKWDD
ELLKVEIQELEDLQFDLKMTGFELEKVQRFLDDFDGKEEVERGVLELIDK
KVEIAKPGDLWILGDHRIYCGDSCVVESFKAVLDDKMADITVCDPPYNVD
YGSSQEREDKKILNDNQGEKYELFLYDICSNILAYTKGAIYICISSSEFS
TLQKAFEEAGGKWSTFIIWAKNHFTLGRSDYQRQYEAMLYGWKSGNKREW
HGGRNQSDLWFYDKPIHNSLHPTMKPVELMEKAIVNSSRPGDTVLDPFSG

SGSTLIACERTGRICRTIELDSKFVDVTIKRWQVYTGREAILSGTGKTFA
QIQEEKQQ

>WD0595_wMel_WOB_AAS14298_CHP
MEKITQTEWAREIGVSNQYVCYLVKKGIIELENGLVNREQANEAVAAIRD
PSQPLRRKNPENENTSNLSTMLLKTRIKNEMERGKLLEAKAKAEIGELVA
VEEVKRDAFNVARVVRNNLLNIPNRVSALLASLSDTEKIHMALTEGITNS
LQELSNIKF

>WD0596_wMel_WOB_AAS14299_proph
MSANLSLELIKCLINQPGVDVNVRGLNGKTPLHYAVEINELSMVALLLNR
KNINPLITDDNGKSALDCAREEILQALINHKYGLEKDSLLHLAAMLNEAN
AVRFLLDKGTNVNEQNALLHTPLHLAAGAGHEQIVEILIREGNADKDVLD
ARNHAAIHYAVNNKKLGVVKLLSNLGANVNVVGSGRNAMKLSSLHVAISS
SNYDERDLCLDIVRCLINVPNAGVNLQDYENKTPLHYAERLKTIEVLLTR
EDIDPLIKDDNGKTPFCYAKEANRLDIVKILVSNRYGADKNSLLHLAARK
GYEDLIDGILGEGVEIDAVDESGKTGIYVAVKHGHFNVVKLLLKRGADAT
DVFQYAIITNNAKLIKLLSKEKEIVLFGRQKNFPTFHLLSNKYFEERKIA
DKKIKKYINIVCVSITVCAIVIVGIYPNIIAAVMVGIVALIAAIAMSNLT
QKYIEEALEKKMFIELESEKTSECSSILSDVEVSSNDGEELAI

>WD0597_wMel_WOB_AAS14300_proph
MIYATSFSEGLKPDPLLKVSEWANEYRVLAPTAASEPGKWRTERTPYLKE
IMDSLSPSSPAEKVVFMKGAQIGGTEAGNNWIGYIIDQTPGPMLVVQPTV
EMGKRWSKGRFAPLIESTPCLKSKVKDPRSRDSGNTVQSKEFPGGIVVIT
GANSSVALRSMPVKYLFLDEIDAYPGDSGGEGDPVLLSIARTNTFARRKI
FLVSTPTIHGISRIEKEFEATDKQYFFVPCPYCNYYQVLKWSQIKWENND
SRTAHYVCTECSGKIENHQKTEMLERGEWRPTNRVKGEKKGFHLSSLYSP
VGWYSWTQAVEDFLHAKESEQLLKVWINTTLGETWVDKGEVPDWKQLFNR
REFFPVGTVPRREVVLTAGVDVQKDRLEVEVVAWGKSRESWSIEYRVFEG
DTGRGEVWGKLSELLNHHFIGENGLEYMISMMAVDAGYATQEVYNWVRGH
QGSGRVMAVKGVNKALVPLSSPSRVDITVGGQKLKRGIKLWPVGVSILKS
ELFQLLNVLKDGEEAPAGYCHFPEYAPEYFKQLTAEQLVSKVVKGYTKQE
WQKVRERNEVLDCRIYARTASIALGIDRWPESKWNSLSEKPESKKSKKIV
KSKWISE

>WD0598_wMel_WOB_AAS14301_HP
MYNEDYLIQVEEAIKKLQNGERVVSIAYGDHVVRYAEVDINDLLSLRQRI
KAELKIAGMKPKRKIVFSTNKGVDKIM

>WD0599_wMel_WOB_AAS14302_HP
MNNMDQSQGRLNVNVNFEGEFAQYLTEMAKVQNRTIEEIVKDLVEEELEV
SKLSEGDEMDEGEIALLELAAKRNVPGAKIIRHEDIKWE

>WD0600_wMel_WOB_AAS14303_CHP
MGIIYQIGYLEGVDTEDLPSLPKTIRLRVQKAIEKRLTIIPDKVGEALSH
KWVGYFRLRVGDYRVIYLIDNSEHMVKIAAIGHRKEIYKRSPE

>WD0602_wMel_WOB_AAS14304_proph
MKNQAIWLNRCVMVEPRSFELLSLQTGKQPIFKNIKHAVRNSERGIIPIH
GILTKKSEVFDDVLGMTSYEKISEEIEEALIDKEVETIILDIDSPGGEVN
GLFDLSDFIYQARRKKRIVAIANDDAYSAAYAIASSAEKVLVTRTSGVGS
IGVIASHIDQSGFDEKQGIKYTTVFAGSRKNDLNPHEPITSESLESLKSE
VNRLYGMLVELIARNRNLSVEAIKSTEAGLYFGEKAVEIGLADGITILSE
FKSINKKGDITMNEKTTNDLETDNLTKYRTEVLELIRLCNLSRMPEKIGE
FIEQSVSVEQAREVLMELLAERTKKTEILSAIPQNSGEELMMQVAKSRAQ
SNI

>WD0603_wMel_WOB_AAS14305_CHP
MISITEGNNLGDLLKYEVSNLYSRDQITVAKGQNLKLGKVVGYDTKDGFI
KALNPTATDGTQTAIGVIASDVNAKENSKGVIIARGAILADHAVVWPANI
IEEQKTAAIKHLEGRGIIIRKGA

>WD0604_wMel_WOB_AAS14306_CHP
MQNPFTNTAFSMTALTNAINILPINYGRVENLNLFPSRSVRFRHITIEEQ
NGVLSLLPTHLPGAPATVGKRGKRKIRTFTIPHIPHDDVVLPEEVQGIRA
FGSESELKALADVITDHLQLMRNKHAITLEHLRMGALKGIILDADGSELL
NLYNEFEITPKVVNFALGTATTDVKRKCLEVLRHVEDNLSGEYMTGIHAL

VSPEFFDALISHSKVKEAYERWQEGAALRNDMRSGFTFCGITFEEYRGQA
TDPEGTVRRFIERESGNCFPLGTASTFTTYFAPADFNETVNTLGQPLYAK
QEPRRFDRGTDLHTQSNSLPMCHRPGVLVKVVVT

>WD0605_wMel_WOB_AAS14307_HP
MQENIKRLLQDCFAHLGKVALYESKDKSYMVQVLKQQPDKLYEIGEGQFV
GEILLLEVSVFDVLKPMVGDTFSIGSCRYRVHTPPLRDNSGMVWKIQASG
V

>WD0607_wMel_WOB_AAS14308_HP
MLFVSILSSNQILRASVSSSMAVTSNFFPLASSTIC

>WD0608_wMel_WOB_AAS14309_HP
MYFSLNVNILIIFCGSLSGLLSFCCLSYCTLFVFYYYFILSIIIYYYYIY
IIYKGFRNAESAIMADFRHIITRNTFRNRGQKVRIQAIFSFLVSHYLFSN
QGIRNIIQPFDVLSLFN

>WD0622_wMel_AAS14323_transcrip
MFVSVSDISSISYKIGQKIEDSRLMRGHTQVELASEIGLTYQEVNSYENG
YIPIPIEVLYVIARVLSVNAIDLLPEPVIVREDSYEDEEILYLTKIYENQ
KLGKIVPSLVRFVHISEKINQEEARLEIAKNLVKEGVSVDIISQATGLSI
YEYDNTEREICTDSIYYRIGQRIREWRLIRRYTQKDLADKVGVTLKEIHE
YERGYTTILFDKLYEIAGALSVNIKVLLPETRESKKLLSLINEYREPESL
DALVKSLSEDMKSGKEKVKKAEKIRVAKNLAKAGVAIDIIVRASGLTADE
CEN

>WD0623_wMel_AAS14324_transcrip
MVLFVEKSLDYKVGEKLKSWRLERGYTQKDLAEKLGVKYWVILQYEKGNR
RISIERLYAITEALSISITDLIPISKSCLEDEGEEILNLVREYKKINDQE
LRKMFCLLTNFVQVSEKSSKKAEKIKIAKGLVKAGVSVDIVAKTIGLSAD
ECVEEKGGSIYCQIGKKIKEWRLVREYTQKDLVEKMSTTRDEISNYEQGR
TAVPLDKLYEMAEALSINITDLLIKEGSKVKNELPDLIKEYKEIESQELR
HALIKSLFEGIRICEEKVREIERIKVAKDLVKGGISIDIILQIIGLSVDQ
IA

>WD0625_wMel_AAS14325_DNA_repai
MNKSKEEIEFRILESKGKALLDREIMETFLSAVHESPQAQEIAKNLVNTY
TGVGRILGREMDDLKVIEGVTDSAVAMIMCVKETLERVLKEKLKSEPIMD
LQGIVEYLNVSIGHSERECVKILYLNKRRQLIGEESYIGEMEKAPVYIKE
ITRKALMKNATSIIMSHNHPGGSLEPSEEDQAVTKSLAAACSTVSVKLFD
HIIITSGGYFSFRENGLL

>WD0626_wMel_AAS14326_transcrip
MANISIRYKIAQKVRSWRLKRGYTQKDLAGKIGVTYQVVLQYEKGTRKIS
IEKLYAIAEVLSVGIIDLIPVSSEKICLKNEEEEILNLVRKYKTINDQEL
RKVFYLLTKFTRVGEKSSKKAEKVKIAKGMVKAGISVDIVSQAIGLSANE
CVEEKTGSIYYQIGKKIKEWRLVREYTQKDLAEKMDTTRDEISNYEQGRV
AIPLEKLYAIAETLSISITDLLIEEDEIVESELPDLIKEYKKIESQELRY
ALIKSLFESIQICEEKVKRAEKMKIAKDLVKGGISTDIILQITGLSLGEI
QQI

>WD0627_AAS14327_CHP
MALSKFLDPKNDISFKRIFGTEKNKDILIHFLNDILGFAGTNAIQDIEFL
STIQDPDIASKKQSIVDVLCRDKNGLQVIVEMQVAKTKGFEKRAQYYAAK
AYSRQADKGDQYHDLKEIIFIAIADCILFPDKSEYKSKHTIRDEDTNEHD
LKDFYFIFIELPKFPKTKEDQLSSIVEKWVYFFKYADETSEEELEKIIGS
DLIIKKAYEELNRFNWSEKEFIAYEQEIKRILDEQAVLAQKLDDATAKGI
LIGHEKGRKEREIEVAKNLLKAGVSIDIIAQTTGLTANEIKDLSSLDV

>WD0628_AAS14328_HP
MNIFVLDENPEIAAKMLCDKHIVKMLLETAQLLSNVFSIALKVPNPLVSI
TDQNIEVPYKLTHKNHPCSLWARQSKGNFFWLIKYGRELCKEYTWRYKRK
HRSEEVIDWCDSNKNLLVFQSTDIKPFVQALPDQYKSSHSVKAYREYYLK
EKMRFAKWEKGREVPDWLLSRIVRLKSRK

>WD0630_AAS14329_HP
MTLTKCVLTSPFSSVQQYLLVLGKVMVVNTKSDQQRAEDDLYTVLRFKEK
GDFERRIRELLVNDYKKLKKPLSKLLGKLESKIRTDKENNQAARNVYDQI

KKSKTNEDKLSNLLNFKKQLKAFLKNQDDCSIVLNAIDSASVIPVIEVNG
VHPKDSEIKKLKKAQPLTSNRQDFEEALGYIESQEKVMGITLEEIMEYKQ
HSVKIFLLNRVHSCKNGHSQLHPDVKEAIAILSNDKLRAFYDEEVKKKTL
QPILSYKECEQLYDKINYEKNESHIIIEREELGENVRRVMGVKRFTNQSS
REDQKNGISPTGTSTNYRQEQVASSGDRTLYNSDIPSLGRSIKSSRSALF
MPFGASVDTSTTIKPANPSNLVTTGENYTSNADLSDELNVSEVTKIVERK
ELLSNSLKKIQEIVEKLGNTTLSEEVIVHERHFKKFMDFVGEILVDIGTR
LPQSGSKSMYLERKLNGLEKMNESLDNSVQELEKKVDKLEKENKDFLAEN
DELKEKLATAKEEKELLFNKKEKMQEVINGLNEDNQRLMQKINGLEDDYK
YLLRAEKTKRETEDSCIQVEDADIPEGKAELCKDYEIKLVKLEQEIDAKG
KQCEELLLENSILRGNIERLKEENKRLEGIEESFSIDLSHFCSVKNLHDE
LILANEQPENIDISIQDEMDSKVATESVGVNVKQGYSKRTSNLSRVSEQS
SAVKSQFSEARAQSRKQIIYASASLILSGVFAVGTSLTMSHLGISISLAL
TALTFLTLGCYCSYKASTTLRNIELDRTFKMADHEAVFMVPSL

>WD0631_AAS14330_HP
MPIETKRQAEVLKKLQDVIKHTDRDIAAGRKLAIKRWVETYIEYIKLFKD
DKLEFLYNVFRDEGCWLGTRLNNTVLGQKLTEEKIGEIDNPLPRYGMASR
YCITGKIGDFFNKQFVLSRGQFTSEEVDSQGNPISDQYVRNILLSSMKRN
GPVFDFWIDRESGELKKYDAVEGFDSTVKLKWSEGVEYFYNQLEEKDKEK
KLTEAIVALSRPQSVKRDAPILDFCVRNIGDKDTLLQKLLQKDKGVYFLL
AELIESCFFDTVHDLVQCWCYKGVSAGGDCSDKIFSQQDYELFLYSLSNV
MLKNPELSVQARSLIMEIWKCERFAEYRETSVNTSNYTVPIKSVLGGLII
NWKREDVCKPDREIEKEEILDMISFAKGCFPEKFDLFKEVMIENLRICGR
EGKRKGVDYGKFAEELFLQLEKVTLPSVGDGPWNNLRSQSKVSLPLDGSG
DGPQSEFEAPSVSGISGSHKKRRI

>WD0632_AAS14331_HP
MDGDLDGFRQEFESFLDQCPFFLYHVSTGRFLPVFFFSMFATAHDANILK
ANERVYFRFDNHGIDTGGRNRNTGNLKVAVYHDGQQVVRCYSISDRLNSD
GLRFSTRERNALVREIRGQNPNLREEDLNFEQYKVCMHGKGKSQGEAIAT
VFEVIREKDSQGRDRFAKYSASEISLLRHIERNRLNGINAPAPRSLLTVK
EIGSIRLNQDQRVQLGHLVNFVQVAPGQQGIFSFMEVLASNQKINIERGI
NEGILPYITRIYRSYLGSLQNDIQNRSQKFESHGFFLGLLANFIHLYTID
IDLDLSPGNSYVAFLICHQAERENIPIVINVTRWRTSSDIALNRARADAK
RLHVSSFISIHTESRNAVCIGLNFNLNIDPFSIDTVEFLENRFPLVQRLF
ECLEDEGIRENIRDFLLQHLPNEIPRNAENYNRIFDCITGFAFGNSILEE
FRLVNAVQQRVRKYIFRYGDENHALTMVFHTQGSDIVILHIRDNNAVQQG
AINLQDLNVDGNNVHVREVSCTLNNQLGLNIHTDNLGLYHNYQNNNANNF
LGGNLVQVPNAGNVHNALNQVMNDGWQDRFQHQELFRNISAVLMPEDTHG
NMIIDVNSKDKFRSILHGTFYASDNPYKVLAMYKVGQTYSLKRWQEEEGE
RVILTRVTEQRLGLLLLRQPTADTHPIGYVLGFADNAEEVEQEQDEARYK
ITELMSKQRGYLPITSGNEVVLSYAVFNRGAQRAEDFISLPQQAVYVHRL
DRRGHDSRPEVLVGPESVIDENPPENLLSDQTRENFRRFYMEKRPGQNSI
FLLDIDDNLHVPFSYLQGTRAQAIETLRSRIRGGGTSTAQGILQQINTIL
RRNNAREIEDVHNLLALDFATENQNFRYWLQTHDMFFAARQYTFHDDRSN
PTNDRHDFAITSVGVDGNQNDPTGRDLLSSNIDNFKQKVDSGEKDRLTAI
INVGNRHWVTLVIVHQNGNYYGYYADSLGPDSRIDNNIRGALRECDISDD
NVHDVSVHQQTDGHNCGIWAYENARDINQAIDQALQGNSNFGEKGEGIIG
YIRGLLSAGIGNDTRQPQRNEQYFRNRRRNISQLFQNDSLSSPRGRLIQG
RPGIQHEIDPLLLQFLELQYPQRGGGGALQLGGERVISIDFGPQSVLDEI
DGVNRVYDHSNGRGSR

>WD0633_AAS14332_prophage_Lambd
MDTGSVKYIAESISEQSKHTDEQKLKCDLSAGQEFSEIGSPKTEATGLPV
DSYYEFIKRDLFWADRSLIDDKKYLEDLIDEWFIHAPNLGLEESQKKLNQ
KLLNSIIKDFNMGNYDSFSNLKQFLESNEKNKDLKYVLNLKRGHLGTTIL
NVFLEYDEIIPSLLKAGADLNMQDNKGKTLLHDTAIYSSGYEDLGYLLDA
KADPNIQDEKGNTPLHYYAAKCSDQSRKTMDLLISKGADLSIKNNDGKTP
LQVAIDNDNIIGCLLTNSQKKLREQLGKMLLATSSDEDWDNAYDPEVENL
RKFLNQYENDNDLKIVLNVKDDSSVLLDSPSRRFPNVKALLLKAGAADFI
GKKQDNYEKCDSFLSEIYQINYLAKRNEFLSKVVKAKSMIELQEVVNEII
ASGMRLNFAKDKDYYFADHVLEKIAQLEGSYGIASDIVCTLISRGAKLKR
SESLKVIDTIELKFKAHKANMISAHLEYVSNTEEFFRIAKAATSGQLYDG
KIDNNVFYLEYSEDSIIDVARITDRTRNLELIQESYRRDIIKIGKSKMEI
ITENGIRYYTDLTEGSDIVLTFYTSLGNIDLRLYPDIQDKSKIIVEVSNR
EEILEKFKGREEELGNDCALGGYSVYNAIEQGYFERSRKLMRPEVISESN
NKWTEREELRRDSMEEIARRHKLLQDLRNIESNIVQKEKNFDIKTYLIDI

FKTLSRFYEEKGDISKTDLAKAAEKESKKLGLEGKYNWSKIFGLEEEIIE
KVEKQGDKEQKSNIPDDFYLGHAINNGSCFFDSFRQSLEQQKGIKVTVEQ
LRNECKRFAQDNPPEWFISKIGNDFDEVESELVNRGITCNQYINSIGKNE
FWGRSDIEGRVLCDKYGVKLHVAESNPLHTIDKQQDPFLHQLIDSSRSKA
GKIDYSHNSALHMVNGGHDHFQPLLYRNKTLAKQTQEQKDSLCYSSLPSC
SMDELKIEKANIRHCL

>WD0634_AAS14333_prophage_Lambd
MLKEVRCAIYTRKSNEDGLEQKFNSLDAQRVVCEKYIKSREGWVALAKKY
DDGGFSGSNLNRPAIKELFEDVKVGEVDCVVVYTLDRLSRETKDCIEVTS
FFRRHRISFVAVTQIFDNNTPMGKFVQTVLSGAAQLEREMIVERVKNKIA
TSKEQGLWMGGNPPLGYDVKEKELIINEKEAKIIKHIFERYMELKSMAEL
ARELNREGYRTKAKSDIFKKATVRRIITNPIYMGKIRHYEKQYKGKHEAI
IEEEKWQKAQELISNQPHRKAKYEEALLKGIIKCKSCDVNMTLTYSKKEN
KRYRYYVCNNHLRGKNCESVNRTIVAGEIEKEVMKRAECLYGDGENLSFR
EQKEAMKKLIKGVMVKEDGIEVCSESEEKFIPMKKKGNKCIVIEPEGKTN
NALLKAVVRAHSWKRQLEEGKYRSVKELSKKINVGTRRIQQILRLNYLAP
KIKEDIVNGRQPRGLKLVDLKEIPMLWSEQREKFYGLDL

>WD0635_AAS14334_CHP
MERKEEIEKKVMNLEKKALEEVRKIWKKVYGEGAPRYSKKYLIPRLAYRM
QEKAYGEISRKGAKRLEYLADRLEKGKRISSDKLPVEGTELILERGEETH
AVKVTDKGLIYKEEFYTSLSAVAGKIMGMSYNGPLLFGMRDKEKEC

>WD0636_AAS14335_prophage_Lambd
MSKKEKEDELKELLENSGKNIKGTKKELHYAVDGKTVSLLVEKGANVNAA
DVEGYTALHLAITEKRLETVRELIKSGGNVNAEEYGSKCTPLHLACMVGK
VEIVEELVKAGAEIEQADKFGMTAMDYAKNSKEVTEVLKKETDRIEKLFE
KL

>WD0637_AAS14336_prophage_Lambd
MHHAVENSDHKTVRLLIEKGAEINARDKNGYTPLHCAVFAKSLENVKVLL
RSGAEINATQYVSGCTPLHSACKIGGAGVEIIKELVKAGSEVNQLNKYGS
TPMYYIWESEKYYLCDREESEKASKFLRAKGGVTKSRKLTCYGIEGLVGE
IADKLDRSYMPELKIMEIEEIRKRDKSLIKKECQNLATKIMCQVNEMIDE
VVRIKKGT

>WD0638_AAS14337_CHP
MLLPPNATKKEKALVDAIDYKVDPGCIRGFKFSQGEKVLPWIIEEYGLGE
ILHWVKDKRKAIKKGIKFQRLRGTPASLKIALKWANIEDITIIEEPSGKH
FFELQIGIKDVLNDFFVDAVVELAKLSLPARSRLMRIFNDYYNISRFILD
ESFFGSLLSDYSGVKIEKDGPVLSFGRVNFFRSHGPVIRIVENYLRDHYE
RALSNDIYRLDVAILGETEPHTKNYNGIYERNHVWYNFKALYPLPQSLLP
KIKFAKAQIVLSDSWNLGEINACFPVTSGEEKGSKFLLGSDKLSEQLWNL
KHKPILERFSVTHHYKVEDFTNQKVIIFGVAEHNIHFERDLNSEQKDSIH
EPENYVLVFYPGVLTWHEHRHLNRGWEEKQVICLIY

>WD0639_AAS14338_prophage_Lambd
MQQTNIIEPLNFEEIFSRMKEELISRDESFTALVESDSAMKVLEVAAWRE
LLLRQRINEAVKSNLLKFATGEDLDNLAEFYGVERQKEEDDERFRKIIKA
KIIGWSTGGSKEHYRYHALSADSRVKDALVESPIPGKVQVSILSTELSTL
QEELLEIVKNQLNREDVRILTDTVEVVSCNMIEIDIHSKISIKRPDIIET
MKKKFIEKFGTTKRLGWKVTRSWIIANLFMEGVENVELIEPREDIVVKGN
ECAFLKSLNVELN

>WD0640_AAS14339_prophage_Lambd
MDGKTGKALEGIEHLKQSIIDILTTPIGSRIMMRNYGSRLFELLDKPVNR
DLTLEIYAATAEALEKWEKRFKLEKVKMTEVKEGKVTLDLEGLYVPMGKK
IRFDEVVV

>WD0641_AAS14340_HP
MNKSVVRVGDYCGEATPHFCISGSNNVFVNDFPVCRKGDNFNEGKVMIQG
SKTVFANGHGVGRVGDSVSCGLKVIKGSKSVFAK

>WD0642_AAS14341_prophage_Lambd
MLESNFAISELQRKLANIIRIGLVKEVDYEKARVKVQIGEFLTDWLPWIT
SKAGKDRNWSPPDIDEQVIILSALGELSLGVVLAGIYQEKYPAPENKKEI
NSVKIQDGTRLLYDKEKHHLEIEVVDKITLKAGESSLEMTRSEIKLKADR

IELN

>WD0643_AAS14342_CHP
MILIELHKAICRMLKKEIPAIQTCEIYPMIRREIVAPAVFVELSSLEQGK
DSGTEELALKARFEARIVIDSTIENAAIIVRELAAEVARVVNKNTWNVKN
VSPGEFISAEIDGFRPELDAYLVWMVDWVHEVHLGRSIWEEGKFMPHKIE
IGEINVGK

>WD0644_AAS14343_prophage_Lambd
MEKAAVRALNKTALWLKTQAAKEISEEKKIKLTVMRKRLRIFKAKTSRLD
VLMRANLYDIRVSAIGKMRQTRKGAKVGKHKFIGGFMATMPRGNSGVFRR
EGRTALPIQEVKLGLEPEASKIIKELVNYETEGIFEKYFERELNYIVKV

>WD0649_AAS14347_secretion_prot
MISKLKKLSTGFSLTGLIGHSDNINMQGAKNKKKKYPKFLDKTFALIDAM
INFILKRESNNVNEVLRVTWGPLFFGLVVILIFFGIGGIWSAIAPIDGAV
HASGEVIVSSNRKIVQHLGGGIISKILVKESQAVKKDEPLVLLSDVNEKA
NLSIIKEKLLSFLATEARLLAIRVDLDTLEFPDEVKKLSHDELVNKAIKN
QVKLFNSQRKSILGKTDILQQRIKQLNDELAGLNFQLNAAHKQYDLITEE
LETKRQLLDSGHISKPHILALEKQFAEIEGRVGHYRSAISQVQQKIGENE
LEIINVRNDSQERANAELKEVSTSIADLKERLMVAEDSLARTIIKSPQDG
IVTDIRYHTEGGVIQSGVPIMSVVPSDDDLIIDAKIQTRNIEEVLSAQKK
DSNIVSIDGLEGLKVKVRLSAYSARRLSLISGIVSHISPDALDDPRLGRY
YSVRVVIPKPELAQFKNVYLYPGMPAEVYIVTQSRTLLSFLFTPIIATVD
RSFIER

>WD0650_AAS14348_3-oxoacyl_acyl
MFGLESRKFLITGASGGIGQAIVKIMHKAGATLCVSGTKKEVLEEVAKQY
EKNMHVLPCDLSNAEEVNQLVNRASELMEGFDGLVCNAGITQDSLLLRMT
DETWQKVIDINLSSTFKLSREACRKLIKNNWGRIINISSIIGLTGNAGQA
NYAASKAGIIAMSKSIAKEVASRNITVNCIAPGFIDTKMTEVLNEVQKGK
ILDNIPMKRMGTGEEIAAGVLFLASDEAKYITGHVLNINGGLFM

>WD0565_wMel_pyocin_AAS14271_pa
MVKYILSVDGGGIRGIIPAIILAEIEQRTRKTIAEIFDLMAGTSTGGIVV
AGLCKKDKPQYSANDLVEFYREYGPYIFKSSFFRRSILSWFNCAQYPHKN
IESVLDKYFGEDILKNTLSNVLITSYDIQNNCPFFFKSWKEGNIKLKDAL
RAATAAPTYFAPKYLKVNQKEMVLVDGGVFANNPAACAYASGKRLFPNDD
ILLLSIGTGRTDRSIANSRRFGKIGWIKPLLNVMFASSLDAVNYQLDQVI
ADKYIRIQSQLKIASPDMDNITSKNIKSLQQEANAMIEDNQELIDKFCDV
LS

>WD0566_wMel_pyocin_AAS14272_an
MKKIKYNERDKLHFVWFILLIVCVVITYCYQKSKATDNYNKTLQVATSNC
NLGIVKLLVKDMAPNLSGTTLHCAARKGCLDIIRFLIEEEKVNINALDRN
AFKRIALHHAAGEGHLEVIKFLLEKGANPNIRDIDGKNPRDVAVLRSRHN
KDKPYDEIIHLLYNAEKEHESEQ

>WD0567_wMel_pyocin_AAS14273_pr
MQPDFIIDKCESIKDRVISLHLTDESGIIDDVVEVCVDYRDEDIDIPNEL
NIALGYKEFGIFPMGIYTVNEVTIQGPPKTLLIKAHATNLRISLKAKVSK
EWRQITIENLVKEIAQKHGYGYKVAEEFKDVLIPHINQADESDISLLTKI
ATEREAMAKLASGYILFISKNMAKSATGKALGTTTIRPQDTINWKVHFTV
RDKYNSVVAKWHSYEKGETIKETVGSGEPSYIMLELYSNAESALSAANAK
LKQLKLNNETLDVTMPGNPQIFAEAKLNLLGFNQALDGEWIINRAEHTLN
SSGYLTMLSASLSK

>WD0568_wMel_pyocin_AAS14274_pr
MTIYYVSKENEMLDLICWKHYGFTDGVVELVLAENLGLAEYGSFLPAGLK
IKLPTIKKIVQKSKLKVWE

>WD0569_wMel_pyocin_AAS14275_pr
MMLSLGPYKFAPTSLKYSRENRWSTIECIENMPLLQNIGQGVENIDLEGM
IYLHNLNVLNQLKSVKETEKPHILVDSLGNILGQFVITRLEEKQMYFLPN
GLPRKIEFSLSLKSYR

>WD0570_wMel_pyocin_AAS14276_pr
MKAIEKPSKTLKAEFDKAKTSAIKAKEAYLKKRDALHSFNEEVRKSGRNI

KYLVSDQHKLGSSIEVLKGKYGKLGSAIRSHQSFLASKAHFKSQIIETIG
LGLTFAAPVKVAIDFESAMADVTKVVDFKKGTDEATKFAKKLKEMSRTIP
LSAAELAQIAASGGQLGIKKEDLFMFTETVAKMSTAFDMSAEQAGDSIAK
LSNVYGIDVSKMEYVGNVINHLSDNTAAKAKDMVEALAIVGGTAKQFGLD
IKETSSLVNAFVSLGKQPAKAATAINALLSKLQTAEEQGGDFKAALEQMG
ITAEEIVQRISENGEEALLYFFQALKKMDNQERSTILMKLFGQEYQDDIA
LLAGSFNKYEDAIRLLSDTEEYKSSLQKEFQNRVDTTASKLRLLRNAIAE
VGMNLGSVMLPTLKSIAEFLQEKTRSIALFAEKYPTLTKAIMGTVAALIS
LKVVAVGLGYGFTLLGSTIFSLKANLLGVFSFLSATVFPAVVTGLRAVTL
ANPIGLLITGLVTGAALVITNWQKVKDFFSSFWKSIIKPIEEAFSWIGES
IFGKVLGNSPLKEFEKRKTEVKAVHTPLKSNILNSGNPVLGNSIIKEFSK
RNKNSFRVKSLIEETESDKIFARSKFENKEQNKTQNITNNYTISIKAEPN
QDVRSLADEVIKRIREKSRDVLFDTVETFY

>WD0571_wMel_pyocin_AAS14277_HP
MSTLSIKIGAVLDGSFSTVIKGSSSQLTHLGENIRKLDSSLKSVSKFKQL
GHDVLTSKRSWKGFEDQVKS

>WD0572_wMel_pyocin_AAS14278_CH
MQAITLNNPITVDGISVSELTVRRPKVRDYLAIESLNGSDLSKEVTLTAN
LTSVAKEAIEELDIADYVKIQEVLKDFFSPIIQKT

>WD0573_wMel_pyocin_AAS14279_HP
MDSKAHTNSIEQLTGLLVSVAQSQDKSVLKLLTELMEEEIEDMELSKIAD
ERYKEKRVKHQDAFWD

>WD0574_wMel_pyocin_AAS14280_pr
MLPKILRNFNVFVDGRGYAGKIDEITLPKLTIKTEEYRAGGMDIPINIDM
GMEKLEADFTFAEYDSELFRLFGLIDGNSVSLTLRGGMQGNDIEAVIINL
RGIFKEFDFGNWKPAEKATLKCTVAAHYYKLTIGGNELIEIDAENMIRKI
NGVDQMALLQTILGI

>B2gp1_putative_DNA_recombinase
MTTVSLYARVSSRQQAQENTIESQIVELERRISSDGHELLDEHKFVDNGY
SGSNLERPGLENLRDRVAEGKIDKIYIHSPDRLSRKFSYQMILLEEFKKA
GSEIVFLNHKFDDNPDSHLFLQIQGAIAEYERAKIMERNRRGKLHAAKAG
CISVMGRAPYGYRYIAKHVGEGSAQFEVDEEEANIVRKIFSRVGQERASI
GEVVHELNKIPVITRTGKRYWKRSTIWNMLKNPAYIGQAAYGKTRTCSKP
QVKKSKKGTCGKLKSGRFNSDKENWTYIPVPKIINEHLFDSVQAQLTENR
QRARVRQRRETYLLQGLMVCQRCQYTYCGTNHVHKKSTYYYYRCSGTNSS
KFNGNKICDNKSIRTDILDGVIWEEVKSILKEPDRIANEYQRRLSENKKP
LHNQTREKQESKLRLSIKKFIDSYAKGFISQEEFEPRITTMKQHLKEIEE
EKERTLDQKKLQQELSLVTDSLKNFSSSVESKLDQVDWQTKQNIIRMLIH
QIEINHNHLYIVFRIKSLANFDQNSHNRIMQCCTGSQYCWTAG

>B2gp2_HP
MEIYPSHKFWESDLEVPVNLLLDRFQDSNIRQSWLDSLSGKQLSIIFQHC
FKNHLNGQLFQDGDYDDRSTQQKRKILASYSDSLFNYYLISYFDRTKLEA
TVSEVARFALTQELMRSYLIKNNTKYDKRSLLFLLFHINCELLKSVYHFD
KVQKRGFVSFALQKSPRQINTSFKEFMSQEAVEQILKFENQLQGFFHHQD
RIYMFVRRGSDMDLLLNSNKVVHGHKPEWMILDFSIDGTKVNLCAKNTNK
AVEIANSIVSGYFNCECTFVNIQDKNFLLQVHKFLQACIEGSDPNICIFE
LNFKSDYFKNSNTYLTLSVKPYDPIAPELHILKPSIGNILQSIQSAKVMF
QNKKVTFSFKSSGEIYYSEHPLNKKEREDLKKHMEQSYGLKILSRANC

>B2gp3_HP
MVLKSFHEQIADLLSSNSSWVNPSQNHIKAARYLFDLGLIKMQERYYIVC
SREEDHLDWPNVIDPSCSNEIFIDPDFDEACDDAICENCSRHILPNTYKK
QRFHLLSIYLNIEKIIDWFETKLNDSRLMWEKVEKGVYYICNQGRIVNLI
ILDFCTDAIFLTIDKLRVHPTVLITLKKDIPNLLLNLYVVPMVKLFCQLK
TVTEIFQEADKRGVPEVVENTSLQVLPASYISLKRVEPIIPTKLLELQVV
KGMVYVNSIEVINKKAASCLNIFRILFKQFLHDCEKELPPEKHTLLNINQ
LEKLLGLSLEADLEHQIRKPLNKMQKTIKATLAEKLGLSIERDDVIQTLG
WQRSSHGYRINPFTLTIKK

>B2gp4_putative_regulatory_prot
MLELKTQLLQNIRSCLFHLLPRGTFRGDKFYVGDVQGNKGKSTVIELTGE
RAGLWKDFATGEGGDIIDLWATVHGKNARIEFPEVMLSISEWLGKTNFKE

QRNLEQYLTCSWNYYDENNQLIVIVYRYDPPLEKKQFKPFDVKKQRFKEP
EIRPLYNIPGILKSDKVVLVEGEKCAEALIEKGITATTAMFGANAPIDKT
DWTPLKGKHIIIWPDNDEAGNKYTKNAEKRLLELGVASLATLKIPPNKPK
SWDAADCVLEGINIEEFIEKNSRKIIIKPPLDIFSWSVERFVGPVPKQRF
LVEGLFPLGVTSILAAMGDTGKGMLLLDLALKVAGSIDQVCGFGPLVTEH
GSVVVFSAEDDTNEIHRRLERLDPKCERLKYKGRLFIVPLPNIGGSLTIL
KNVRGKVVEISPEFESVMKQLSRIKDLKLIIFDPLASFIHADLNADPAVG
DYLMCLLSDLACSTGASIITAHHMRKLKGEKPISTVEQARDAIRGTSALV
NGVRCSFAFWPVEDMTKPMIFRSIRKIPRQNALFYGAVVKANGLADRTIR
TYLRNEETGLLEDITEQLKAQNISDKDLKIYLIDSIARAAISGHPFTHTG
SAGIHKQRHRLPEVFHSMGRDRLERIVQELLQAKQIVKGMANGSKEDKWL
DIKTGPFARGVGKFIHGAEKNYF

>B2gp5_HP
MKYDEEKLWSAVVTRGIQDAVGKNPKLREEAINWLNSKSFETVCELANLN
FTRMKNMYGNFMSKKQKELKMLLIDNIKECVSYLLPNGEFYREKTYIGDL
NGNTITVKIVGKEAGDWRNFTEGTGGDIIDLWILIKGDIYSARKWLNKKS
KSGEKKGRKREEKIFSVKQYLSDQSPIPEDIIAPRILTPGGLLVIGGTPK
VGKSYFLLSLLAHLAAGVSFLKMKSARPLKIIYLQNEMEYNYIRERIQQI
ITNQRLPNLAEENLIVTTKMRLTLNDEGIERIKDIIGEKFKTIDLIVLDS
LDYENMFSGLQSRVEKLRSVINPMAGIIITRHTRKVSTATLAKSPFQALI
GANALRSFYTSGMVMFQPNKRTNVLQVVYELRNGKSIPAKFISRVNGRWQ
NSKVIATA

>B2gp6_putative_phage_related_p
MLQNLLTDFNTVKPQSRLIPKGTIVKVKMTIKPGGYEHWFTKSPTTGSIY
LNTEFTVIEGPYAKHKIYQIIGIKSSKAEDTWGEMGRSMLRSILESARDI
HPHDNSENAILARKLNSVAELNGLEFTVKVGVITDEYGEKNKIASVVTPE
YRKNYEIDRVPF

>B2gp7_HP
MVLKILNNNERLQTISTVKMVIFGPYGIGKTSLLKTVDEPTLCLDFEAGL
LAVQDWQGDSISLRTWNEARDIACLIGGPNPALKSDQAYSQRHHEHVSGK
YKDLFPEFSKYRCIFVDSITVASRLCLLWAKMQPEAFSERSGKQDMRAAY
GLLAQEMMAWLNQFQHIRDKDIIIVGTLGQYLDDFNRPIWLPQCEGAKTA
SEIPGIVDEVISMIGIKKNDGTEKRSFVCHTLNPWGYPAKDRSGRLSMVE
EPHLGKLLTKIKSKF

>B2gp8_sigma-70_region_2_domain
MKLKNIAIIVKNVKYQAYRLKLAKCFIDENNEDLEQELFCEIWPCLDQYD
EDKSSFNTFVARLTENKAINMLKKQQCAKRNINNYISIDVTELLEGEITK
RIDVDYMISVLPKEWQNICEQLKFFNLHEVAKMNNVSRTTLNNIIKKIRA
KLSPIYYEGKKKN

>B2gp9_HP
MPLCRTGAKINNTDAIFNLLIFYCFFLRIILIQFKIIQFRFLEPILNGFQ
YF

>B2gp10_HP
MLSITLSSVPNYWLPKSGIRKQESRISLYGGFSPCVPKTVPRNGISEVRQ
YGGF

>B2gp11_HP
MLTLDLGKQTGWTILNDGIVQSGSKSFHVSRFSGGGMQFLNFRNWLNSLK
YKFPGIEVVYFEEVRRHLGTDAAHIYGGFLAHLSAWCEESNIPYQGVSVK
TIKRFITGKGNASKADVIEAVQEKGFCPTDDNEADSLALMFYVMNFSKDF
NMLEIS

>B2gp12_HP
MRITQAEWAREKGFSRQYVCSLVKKGIVELEDGLIDREQANEAVAAIRDP
SQPLRRKERGETLSTILLKTRIKNETERGKLLEAKVKAEIGKFVSIEEVK
TEAFNVARVVRNNLLNIPNRVSALLASLSDTEKIHMALTEEITNSLQELS
NTKFQI

>B2gp13_ankyrin_motif_protein
MTDNLSLELIKCLINQPGLDVNVRGLNGKTPLHCAIEFDELSMVDLLLTK
KNINPFVEDNEGKTSLDYAKEGKKAEILQALINNKYGSEQDSLLHLAAMI
GEVNAVRYLIRKGIDVNVRNALHHTPLHLAAGIGHENVVKILVKEGNAEI

DVFDARNQTPMHYAVNNKKLEIVKLLLRLGADVNSARMGQNSMKLSPVHI
AVSNTNYDERDLCLDILKCLIKEPNAQVNLQDYENKTPLHYAERLKTIEV
LLTREDIDPLVKDDSGKTPFDYAKPEIKKALMSNKYGSEKNSLLHLAAQR
GEIEIVDAILKEEINIDIVNNKGHSPIYLAAERGHLHVVKLLLKKGANYT
PVLHLAIKSNNLELLKVLFTEKNGSLLCRDTVINFPTLHNKYIAQREIAD
KRTKKHNNIICIYTTFSAIAVAVYIGLITTTISSAIIFATITGIFAFVIA
IMVSEMSKRYIENEFQKKMFMELEECGGINSTVNNIEIEPIMSRCRQ

>B2gp14_phage_terminase_large_s
MIYATSFSEGLKPDPELKVSEWANEYRVLAPTAASEPGKWRTERTPYLKE
IMDSLSPSSQAEKVVFMKGAQIGGTEAGNNWIGYIIDQTPGPMLVVQPTV
EMGKRWSKGRFAPLIESTPCLKSKVKDPRSRDSGNTVQSKEFPGGIVVIT
GANSSVGLRSMPVKYLFLDEIDAYPGDSGGEGDPVLLSIARTNTFARRKI
FLVSTPTIHGISRIEKEFEVTDKRYFFVPCPHCNYYQILKWSQIKWEDKN
PNTAHYICIECGKKIENHQKTGILERGEWRATNPIKGEKKGFHLSSLYSP
VGWYSWTQAVEDFLHAKESEQLLKVWINTTLGETWVDKGEVPDWKQLFNR
REFFQIGTVPKGEVVLTAGVDVQKDRLEVEVVAWGKGRENWSIDYRVFEG
DTGGREVWGKLSELLNHHFIGENGLEYMISMMAVDAGYATQEVYNWVRSH
QGCGRVMAVKGVNKALVPLSSPSRVDITVGGQKLKRGIKLWPVGVSILKS
ELFQLLNILKEEEGKALPGYCHFPEYAPEYFKQLTAEQLVSKVVKGYTKQ
EWQKVRERNEVLDCRIYARAASIALGIDRWQENKWNSLSGKMESKKPKKV
RQSKWLENV

>B2gp15_HP
MYNEEYLVQIEEAIKKLQSGERVVSIAYGDHVVRYAEVQINDLLNLRQRI
KAELKIVGMKPKRKIVFSTSKGIL

>B2gp16_phage_portal_protein_la
MLLKTFKQLFNKPKIKSSAWDAAGSGRRFFHFQPELGSINNLLSQNLETL
RSRSRDMVRKNPYAANIIDTIVSNSIGTGIKPQSKARDGEFRKKVQELWL
RWTDEADSNGVSDFYGLQALVCRSMIEGGECFVRLRNRKLEDGFSVPLQL
QVLESEHLDNKTNQTLGNGNVIRNGIEFNRLGQREAYYLFKEHPGESMFG
ESVRVPANDVLHIYRPLRPGQIRGEPWLSNILLKLYELDQYDDAELVRKK
TAAMFAGFITRLDPEANILGESESNEQGVALSGLEPGTMQLLDPGEDIKF
SEPSDVGGSYEAFMKQQLRAIAIGTGITYEQLTGDLTGVNYSSIRAGLIE
FRRRCTMLQHNIMVFQFCRPVWDRWLELALLSGELDIGEEWTKGKEGAKK
EVKWIPQGFDWVDPLKDQQAQQMAVRNGFKSRAEVVSEMGYDVEEIDQEI
AEDQRRASELGLSFDSDVTANQEVI

>B2gp17_putative_minor_capsid_p
MVEPRSFELLSLQTGKQPIFKNIKHAVRNSERGIIPIHGILTKKPGAFDE
MLGMTSYEQIEEQITQALADSSIETIILEIDSPGGEVNGIFDLADFIYES
RGKKRIIAIANDDAYSAAYAIASSAEKIFLTRTSGVGSIGVIASHIDQSG
FDEKCGIKYTTVFAGSRKNDLNPHEPITSESLENLKSEVNRLYEMLVELI
ARNRNLSVEAIKSTEAGLYFGEKAVEIGLADGITILSEFKYINKNRSITM
NEQTITDLKEETNNLTKYRTEVLELIRLCNISKMPEKIGEFIEQGVSVEQ
AREVLMELLAEQTKKTEILSAIPQNSGEELMMQVAKSRRHLKYITKGEKQ
NEYYNRTK

>B2gp18_HP
MSTITEQNNLGDLLKYEASSLYSRDSIAVAKGQNLKLGTVVGRDKDNMIK
VINPTATDGTQTAIGVITSDVNSKDGDTKAVIITRIALLADHAIVWPANI
TEEQKAEAIKQLEVRGIIIRKGV

>B2gp19_HP
MQNPFTNTAFSMTALTNAMNILPINYGRVENLNLFPSRSVRFRHITIEEH
NGVLSLLPTQVPGAPATVGKRGKRKVRTFTIPHIPHDDVVLPEEVQGIRA
FGSESELKALADVITDHLQLMRNKHAITLEHLRMGALKGIILDADGSELL
NLYNEFEITPKVVNFALGTATTDVKRKCLEVLRHIEDNLSGEYMTGIHAL
VSPEFFDALTSHTKVKEAYERWQEGAALRNDMRSGFTFCGITFEEYRGQA
TDPEGTVRRFIDKDTGHCFPVGTASTFTTYFAPADFNETVNTLGQPLYAK
QEPRRFDRGTDLHTQSNPLPMCHRPGVLVRVVAT

>B2gp20_HP
MQENIKRLFKDCFAHLGEVALYKSKDKSYIVQVLKQQPDKLYEIGEGQFV
GEMLFLEVNVFDVLRPMVGDIFVIDGRKYKVYSPPLRDNSGIVWNIKCTV
LGEKDV

>B2gp21_HP
MFNIEVNENIERIIHNIDANKAKIELAAVRALNKTALWLKSQAVREISEE
KQIRLKVIRKRLRIIKARKSTLKVLIRAYLYDINIKQAKIRTAFNDAFMA
TMPRGYRGIFKRVGRTALPIQEVKLPLEPEASRIIENLVNYEVERIFEKY
FTHKLFYDGILD

>B2gp22_HP
MTAFWTNLHEAICNTLKAEIPAIQTCEVYPAIRKELAAPAVFIELSSLEQ
GKDPGTEELALRAKFEARIVIDSTIENAPIIVRSLAAEVARVVNKNTWNV
KNVSPGEFISGGGDDFRPELDAYLVWMVDWAHELHVGRSIWEVSKIKPHL
IDVNVGK

>B2gp23_baseplate_assembly_prot
MLESNFAISELRRKLANIVRIGIVEKIDYEKAKVKVKIGELVTDFLPWIT
NRAESWSPPNIDEQVIVLSPLGELSLGVVLPAIYQEKYPPPENKKEVNNV
KFQDGTRFTYDKEKHHLEIEVVDKITLNVGESSVEITKSEIKLKAKRVNL
N

>B2gp24_HP
MNKSVIRLGDHCEGALLHFCISGSRDVFINGRPVCRQGDNFSEGKVMTQG
SKAVFVNGKAIARTGDLISCGAIAEEGSDNVFAG

>B2gp25_baseplate_assembly_prot
MRGMSSETGKTISGIEHLKQSIVDILTTPIGTRVMRRDYGSRLFELIDHP
IVPGFAQELYAAVAEALEKWERRFKLKRVQITEIKEGKVTLILEGTYLPN
GEPIRLDGIIV

>B2gp26_baseplate_J-like_protei
MKTPNIVETLKFEEIFSRMKEELVRRNASFSALTESDPAIKILEVAAWRE
LLLRQRIDDAAQANLLAFARGNDLDHLAEFYGVLRKESENDESLRKRVKA
KIVGWSTAGSKEHYRYHALSADTRVKDAQVVSPIPGSVQISILSTENGGV
PSEELLEIVRNHVIRDDIRVLTDTLTVIGCGIIPITIHAKVHIYPTASKD
VIEAAKEQFIKDLESAKGLGWNVTRSWIIAHLFIEGMQNIELTEPIEDIV
VQDNECVTLNNLSVR

>B2gp27_HP
MILPPNASKQEQILTDIINYSFDANSLRGFKFNPQTEMLPWLVAEYGLGE
ILSWGKKATKSVLKEWVLAKQNGIRFQRLRGTPQSLKMALKWANIDNITI
QEEPPGEHFAEFQIGISDVPNDFFVDSVIALAKLSAPVRSRLMRIFNDYY
NAQRFMLDESIFGDLLSDYSGVKIAKEGPVLSFGRKNSFELKIGNPRFKF
GTFRSHYDRSYSNDLYRLDVATLGETEPHTKNYNGTYERNHEWYNLEALY
PLPQSLLPEIKFAKALIVLSDSWNLGDINACFASTTYEEIEHTFHLSEDK
LSEQIWNFQRTPILERFSITYYYEAKNFTDQKIIESNLVEYHVDCKNDSD
SKQKDPIHELENYVAAFYLGMVTWHEHRHLHRPWKDFDPIIAKKLDVFST
DLVYT

>B2gp28_HP
MSVLTQSGRAAIAASIKEQPIHLAWGSGDSTWESSYKVEKTFAAGGKISL
DHHTIKDVKVYTGQTIYQSSIDYIVDSSSGTIERVENGAIIADSVITIEY
TQDTPPELITSATLIKEVGRRVVDEILFCTGDDDGELLTPTGRFKPSNVP
TNNLYLKFTFDFNDAANQVIRELGVMVGTKVKEGLPIGQRYFEPKDVENP
GILLVLEHTVPLIRTAATRETFSFVVTF

>B2gp29_HP
MTLNAYYNRFNPDKEYEKSLFLAGRGLQSAELNETQEYALSKLKGIGDAI
FRDGDVITGSNCIIDRETGKVTLESGKIYLRGAVRKVEKEEFIIPLSTIV
RIGVYYLESTITELEDENLRDPAVGTRNYQEVGAARLKVSTIWGYQAEGL
SPRFSEGEFYPIYNIENGVLIEHSPPPQANIVTTALARYDKEANGSYVVN
GLEVMFLQKEEGEGGKKIFVINEGKAHVDGYEIELPHSIRVSFDEDPDIK
SVESEPHTFQPNSQRVMELKVNDFPISEIKKVDITVQKTITITHGSYSGA
IDPIPDSAVLEIIQIKQGNVIYENSIDYKLNAGNVDWSLPGKEPAPGSSY
QITYRCRTHVSPEDISEEGCKVRGAVDNSLVLIDYTWKMPRYDLITIDSK
GAVRRIKGIAHPWRPSMPKAPSGQLLLCYIHQTWKNGEKEGVKIVNNAIH
AVPMNELEAMKKGINDLYALVAQERLRNDANSREPTTKKGVFVDSFFDDD
MRDQGISQSAAIVNKELILPIDVTIADIDGGEKTYLLPYELEPVLEQLLQ
TKGEKINPYQAFDPVPAQITLNKNIDHWTEVKTNWKSPVTRVFNVKETTE
LLSSTSYETEFMREAVQNFEIEGFEPDEKLKEIKFDGISIIPMA

>B2gp30_HP
MEKRANNQGKLKGKVKVPANIPAGTKLVQFYGDKGSYGEATYTGKKTITI
EERRRVIAARRVDPLAQTFTLNESRHIGGVELWFVNKGKKRVVVQIRETA
VGMPSQTVIAESYIEPKDIKVDGTATRIEWSPVFCHAGEEYAIVLLTDDS
GTAVKIAELGKYDAVNSRWVTSQPYQVGVLLSSSNASTWTPHQNLDLTFR
LLAAKFSEVSHIIDLGKVTANNVSDLIVLTNVEKVAFDTNVEFILTDEEG
KENFLSDNLPLALRERLSGELTVKANLKGSKEKSPVLYPGLQLVMGNIGE
KGDYVTRSITAGANTKITITYDALIPGTADVKAYVQKNVDWQLVNLTSGK
PIGENLVERTHVLSNFNGNDTRIKLVLSGTVVYRPKVKNLRIIIT

>B2gp31_HP
MSDDKTSRGYSLPHPENIAVQDVVRIRTTIEKIDEDITEREDEHNQLKSN
FERFNFETFLNFWK

>B2gp32_HP
MKEAIYQRIKDLAANSTPDQLAYLAKSLELIADKKAISNVVQMTEVKGII
DALQKRLKDLAANSTPDQLAYLAKALESIVDKSAVSEIVQMTDGKLKELL
DSAKKHLTDLDNKKASSLAVISESEKQLLKRIDEKGTTNLSLLDTRKNAN
IAAINSISNSHKDGLKGLVEDFRAVNNVPPGSSIIGEIETRIENISNEVK
TRDEQLKVSLINEIRKRNMVEPGSLPFLFGVLGRKNNYFGHGTFTTELGK
WSSDITKTDYMLQLLAGSHTYNTDYVSFYRPRQLSFIEGSKGTFIYGELC
TKSFSGSYDEIYYYPYAALGVVFVKNTTNVNINKTIEFVGSSYSSTEYGG
AGLFVGTPDNTNSNKSSISKIVWKNVYQYMSSDSKLAGSGNVEIPAGKTV
AILLYTSSYLYSRTQVSQGMLASNYVHNYGQFIQWGIYNIRSNVLTTGLE
VDVERTLKAWQCPGLSNTYEIWR

>B2gp33_HP
MSIYIRFENDKQVETTTLESKPTENDWYEAPEDFDWQKSYCLTEGGKIAQ
RNQEDIELELLQNAKFSALSNLHAYYDNYTHQYSGYSHQKSKSYEIQAKA
AENILAAPESIDKKDAEIIEPLAKVRGISVVEMARIIQEKAKRAKKAIIK
CEELVDIAEREIGEAKSKEELQTLLDDCKQKMQES

>B2gp34_HP
MTEQFLHGVNVIEVTSGARTVRTAKSSVIGVIGTAPDADGQKFPLNKPIL
IAGSLKEAAKLGQSGTLPSAINAIFSQIGATVVVIRVEEKVEETLSNVIG
GVNEETGEYQGIQAFLSSESIVHVAPRILIAPQFTHQLPEIDGVNPVVSA
LISIAEKLRAIIVADGPNTNDEEAIKWRKSVGSSRVYVVDPWIKLFIEGK
EKILPSSPFVAGLIAKIDSEQGFWHSPSNKEINGIVGTSRPIDFTLGNTN
CRANHLNENEVTTIIHQNCKLKM

>B2gp36_phage_major_tail_sheath
MRRTADLINDSLLRAHLWAVDRNITKTYIDDVIEGVNSYLANLKAQGAII
SGKCYATPELNTPTNIASGKVSFDFEFTPPYPAEQITFRSHLVSGAIL

>B2gp37_major_tail_tube_protein
MLPKILKNFNVFVDGRGYAGKIDEVTLPKLTIKTEEYRAGGMDIPINIDM
GMEKLEADFTFSEYDSELFRLFGLINNNAVSLTLRGGLQGSSDAESVVIN
LRGLFKELDFGNWKAAEKATLKCIIAANYYKLTIDGRELIEIDAENMIRK
IDGVDQMTSMRTALGI

>B2gp38_HP_
MQKIKLTEPIKIDGILVSELTLRRPKVRDRLAVERMGNSDAEKEVALIAN
LASISREAVEEFDLADYNKIQETLQGFLSQQKN

>B2gp39_phage_tail_tape_measure
MKAISIVIGATLQSSFNSTIAGSTKQLSRIGSTIKQLESSSKSVFKFKQL
SHDALLARRSWNELEIKTKSLAQQIKNTGAPSKSLQNEFTRSKTAALKAK
TAYLQKRSALHHLRTEFSKSGRDIKSLIGDQVKLGSSIEKLKNNYTALNS
VMQKRKGVLAQRANFKAQMMDAVALGLTLAAPLKAAISFESAMADVKKVV
KFEDTDVNGLTKLGETLKEMSRTIPLSAAELAQITASGGQLGIKAKDLTT
FTDTVAKMATAFDMSAEEAGDAIAKLSNIYQIEIGEMKGIGDAINHISDN
TAAKAKDIVPALNRIGGTARQFGLTAVEAGALASSFISLGKTPEKAGTAI
NAMLSKLQTASKQGGNFQKAFQQLKINAKEFEKAIGKNAQGTLVKFLETI
AKLGKQERSSVLFDLFGLEYQDDIALLIGSLDEYKKSLRLINGEYKGSMQ
REFENRANTTANNLQLLKNSIAEVGMNLGSVLLPPLNFTVNLLRSATTQV
ALYAKEYPVLTTVIMSTTTALISIKIAAISLGYAWTFVKGSLFAFLSTWK
MFGSVITLVKIGLSAVFPAIIVGFKALTIALMSNPMGLVIGGLAVAATLI
ITKWQVVKNFFVTIWESVKIVWKSFSDWVGKFWNNITQPFRTISNLWNKR

NESKLEVKSVNNVISDSLKYPIAAHSKAVENKTQNNHFSINIHSTPGQDT
RSIADEVMERIREQFSGALYDIN

>B2gp40_F_protein_
MLSLGPYRFSLTERGFTRRNEYRWPALERIGEKPLLQSIGPGEDVIELAG
VIYSGGLGQINNMRNLSKPLLLIDGQGNILGHFVIVKIEETQRYFFPNGE
ARKIEFHLSLKHYDNVLSDGSLNNPVDDIQLDNDILQN

>B2gp41_tail_protein_X_
MLDLICWNYYGFSSGAVEIVLRANPGLSEYELLPAGLIIKLPVIQKETQK
QVVKLWD

>B2gp42_late_control_gene_d_pro
MTPDFSISVEGILITELIKSRLVSMHITDEAGVISDTAVIHLDDRDSLFE
IPRTGAKLNILLGYKETGIVPMGDYIVNEITLQGPPQGLKIKSHAADLKE
SLKEQVFNEWHQITLNDLVKKIADKHGYQAKVAAEFVNIMISHIDQTAES
DMHFLTRLAQIYGAIAKPAGGYLLFVSKGKAKSVTGKTLSTITLTPRDIT
NWKVKFNERNQYGSVIAYWYDYEKAETITEKVGDQEPSYILRDIYASSDL
AQSAASAKLNQLMSNAATLNVTMPGNPELFAEAKINLSGFRKGVYGKWII
NRAEHVIDNLGYRTIVTGIIDSWKVN

>B2gp43_ankyrin_motif_protein_
MKSSLFWKLLTSSIIPIICVIVLYQHFSTDQLSKDVPIKSKASGNDLETL
STAAEKCNLDTVRSLIKNVVDINATHHFGATSLHYATSGGCLEVVKFLIE
EGIDVNTTDAFSWTALHYAVRKGHLEIAKFLLKNGANPSAKNKDKKTPLD
LAVEELNNNKEDIYEEIINLLSNYQLFP

>B2gp44_HP_
MTNTGKIPVAVIATMVIQTIALIWWLAKLDLRVHMHDKFIEQNQRTTEII
YRLEERVKNLAEEVNELEQRQK

>B2gp45_patatin-like_protein_
MNLSRDKNKKVAKYILSVDGGGIRGIIPAIILAEIESRTKKPISQIFDLM
AGTSTGGIIVAGLCKSNKPQYSANDLVGLYQEYGAYIFQSSFWRKSIASW
LSGSQYSYRNMEFILNKYFGESTMADVASNLLLTSYDIHNSCEFFFKSWK
EKNIKLKDALRATTAAPTYFTPKRLKISQTERVLIDGGVFANNPAACAYA
SGKRLFPNDEIILLSIGTGGTNRSIKYANSRRFGKIGWIKPLLNVMFASG
LDCVDYQLEQVIDDKYIRIQSQLKVASTEMDNITLKNIKFLQQEASKMIE
DNQKVIDKFCKQNPKL

>B2gp46_ankyrin_motif_protein_
MFTNRISNNLDQLQFFVEFLNENYEIQKHLSLTPLHLAAGNGQLDLVNTL
LGEGLDINSEIKYDGFTPLYFSIAKNRLEMVNFLIAHGADVNHKTILGFT
PLSFASQQGYLDIVNTLIANGADLSTKTDKLNTPLHLAAENGHLDIVNVF
IEKGLDVNAVNNDRARPLHSAVQNGNLEVVKALISQGSNINAGSSGIGNH
KVDANITPLHLGTQTGRLDIVKVLLEAGANVNAKTDDKITPLHLASQNGF
LELVDILLKAKSNVNAKDYENLTPLHLAAERNHFGVVKSLLLVRGIDVNA
KDHDNSTALHIGSQNGHLEVVKLLIEKKANVNAKKNEGFTPLHLAIQQSH
FEVSDFLIKNGANINTVDDQNWTPLHNAAYNGFSLKIVESLIAKGANINA
KMDDGRRALHLAAEHNHLEIMNFLIENGADINALDNRSWTPLHCAAYDGN
LEVAKSLLDKGADINAKTVKSTTPLHFAVDHDHLEVVELLLEKEADINAL
DHTNWTPLHFAAEKGYDQIATVLLKHGADVNVKENQNKGTALHLAAQYGH
PKVVKTLIINGADVNAKMDKNATPLHLGAQIGNLDIVRSLLMSGAYFNAR
AEGGRYVLPLHFAERRGNPEVIKLLKLVEKLFKAIEDNNYLGIESSIRDG
AIIDSKNVDGRTPLHYAVNNGHIKVVNILLANGADATQVTNKGNTPLHTA
ASKGHKEIIEALLQRVSHNKLSDFINAKTTSSGTTSLHVAAKGGSLEVVK
SLLKHGAIYNIKNKEGKAPLDLSRDQNITNLLKLVEELFENAKNGNVEII
SKLKAIKPDERVAVTNARNDQDKSLIQVAVINKHSNLASRLLEILKSPDQ
SLQDVSVENRVKSLKLBGPANKYRINMTIFPRTEINMLNNNINKELEKAV
YERNLEKVKALLSAGANANLRVERNRDTLLHTTSSVEITKELLLGGAEVN
SRNMYSETPLHSAITLGKELELVRELLKHGADVNATNRKGNTALNHAVKK
SGKNLEVVKELLEYGADINIRNRERRLYDNCSTSLDNAVSDLACAKLLIK
FTLVRNFNKDYGKIIDLSPYKGCSNYSKLSSYLDDCVCEILQMKTDEIKD
SLPLYEFVTNNFNIHVLYNNQLSVRLAKIDYSIRYPIYNDIILDKVKPFL
ERADLLNKLSKIQFYTKLDVADQDAKEKKIILDHDSTYNITEYLSNDNLL
NFIVAFDDSNKRTFQSLSSLNNLVEPGTRLYETSISNDTKRARLE

>B3gp1_putative_DNA_recombinase

MVTVSLYARVSSRQQAQENTIESQIVELERRIGSDGHELLDEHKFIDNGY
SGSSLERPGLESLRDRVAEGKIDKIYIHSPDRLSRKFSYQMILLEEFKKA
GSEIVFLNHKFDDNPDSHLFLQIQGAIAEYERAKIMERNRRGKLHAAKAG
CISVMGRAPYGYRYIAKHVGEGSAQFEVDEEEANIVRKIFSRVGQERASI
GEVVHELNKIPVITRTGKRYWKRSTIWNMLKNPAYIGQAAYGKTRTCSKP
QVKKSKKGTCGKLKSGRFNSDKENWTYIPVPKIINEHLFDSVQAQLTENR
QRARVRQRRETYLLQGLMVCQRCQYTYCGTNHVHKKSTYYYYRCSGTNSS
KFNGNKICDNKSIRTDILDGVIWEEVKSILKEPDRIANEYQRRLLENKKP
LHNQIREKQESKLRLSIKKFIDSYAKGFISQEEFEPRITTMKQHLKEIEE
EQERTLDQKKLQQELSLVTDSLQNFSSSIESKLDLVDWQTKQNIIRMLIH
QIEINHNHLYIVFRIKSLANFDQNSHNRIMQCCTSSQNEQLLIKFTVSIQ
Q

>B3gp2_HP
MNVHPSYEFWESDLEVPVNLLLDRFQDSNIRQSWLDSLSGKQLSIIFQHC
FKNHLNGHLFQDGDYDDRSTQQKRKILTSYSDSLFNYYLISYFDRTKLEA
TVSEVARFALTQELMRSYLIKNNTKYDKRSLLFLLFHINCELLKSVYHFD
KVQKKGFVSFALQKPPRQINTSFKAFMSQKAVEQILKFENQLQGFFHHQD
RIYMFVRQGSDMDLLLNSNKVVHGHKPDWMILDFSLDGTQVNLCAKNTNK
AVEIANSIVSCYFDCECTFVNIQDKNFPLQVHKFLQACIDGSDSNICIFE
LNFKSDYFKNSNTYLTLSVKPYDPIAPELHILKPSIGNILQSIQSAKVMF
QNKKVTFSFKSSGEIYYSEHPLNKKEREDLKKHMEQSYGLKILSRANC

>B3gp3_HP
MVLKFFHEQIADLLSSNSSWVNPSQNHIKVARYLFDLGLIKMQERYYIVC
SREEDHLDWPNVIDPSCSNEIFIDPDFDEACDDAICENCSRHILPNTYKK
QRFHLLSIYLNIEKIIDWFETKLNDSRLMWGKMEKGVYYICDQGHIINLI
ILDFCTDAIFLTIDKLRVHPTVLITLKKDIPNLLLSLYVVRMVELFCQYK
TVTEIFQEAAKKGVPEVVENTSLQVLPASYISLKRVEPIVPTKLLELQVV
KGMVYVNSVEVINKKAASCLNIFRILFKQFLHDCEKELPPEKHTLLNINQ
LEKLLGLSLEADLEHQIRKPLNKMQKTIKATLAEKLGLSIERDDVIQTLG
WQRSSHGYRINPFTLTIKK

>B3gp4
MLELKTQLLQNIRSCLFHLLPRGTFRGDKFYVGDVQGNKGKSTVIELTGE
RAGLWKDFATGEGGDIIDLWATVHGKNARIEFPEVMLSISEWLGKTNFKE
QRNLEQYLTCSWNYYDENNQLIVIVYRYDPPLEKKQFKPFDVKKQRFKEP
EIRPLYNIPGILRSDKVVLVEGEKCAEALIEKGITATTAMFGANAPIDRT
DWTPLRGKHVIIWPDNDEAGNKYAKNAEKKLLELGVASLATLKIPSDKSR
SWDAADCVLEGINIEEFIEKNSRKIIIKPPLDIFSWSVERFVGPVPKQRF
LVEGLFPLGVTSILATMGDTGKGMLLLDLALKVASSIDQVCGFGPPVTEH
GSVVVFSAEDDTNEIHRRLERLDPKCERLKYKDRLFIVPLPNIGGSLTIL
KNVRGKVVEISPEFESVMKQLSRIKDLKLIIFDPLASFIHADLNADPAVG
DYLMCLLSDLACSTGASIITAHHMRKLKGEKPISTVEQARDAIRGTSALV
NGVRCSFAFWPVEDMTKPMIFRSIGKIPRQNALFYGAVVKANGLADRTVR
TYLRNEETGLLEDITEQLKAQNISDKDLKIYLIDSIARAAISGHPFTHTG
SAGIHKQRHRLPEIFHSMGRDRLERIVQELLQAKQIVKGMANGSKEDKWL
DIKTGPFARGVGKFIHGAEKNYF

>B3gp5
MKYDEEKLWSAVVTRGIQDAVGKNPKLRKEAINWLNSKSFETVCELANLN
FKRIKNMYGSFMSKKQEKIKALLIDNIKECVSYLLPNGEFYRDKTYIGDL
NGNTITVKIVGKEAGDWRNFTEGTSGDIIDLWILIKGDIYSARKWLNKKS
KSEEKNGGKREEKIFSVKQYLSDQSPIPEDIIAPRILTPGGLLVIGGTPK
VGKSYFLLSLLAHLAAGVSFLKMKSARPLKIVYLQNEMEYNYIRERIQQI
ITNQRLPNLAEENLIVTTKMRLILNDEGIERIKDIIGEKFKTIDLIVLDS
LDYENIFSDLQNRIEKLRSIINPMAGIIITRHTRKVSTATLAKSPFQALI
GANALRSFYTSGIVMFQPNKRTNVLQVVYELRNGKSIPAKFISRVNGRWQ
NSKVIATA

>B3gp6_phage_related_protein
MLQNLLTDFNTVKPQSRLIPKGTIVKVKMTIKPGGYEHWFTKSPTTGSIY
LNTEFTVIEGLYEKHKIYQIIGIKSSKAEDTWGEMGRSMLRSILESARDI
HPHDNSENAILARKLNSVAELNGLEFTVKVGVITDEYGEKNKIASVEYRK
NYEIDRVPF

>B3gp7_HP
MRFFNHTQASTAVFSCQGFKVWQTKERFSVPSSFLIPIIFINYSRNLTKS

LRIEVASLDG

>B3gp8_HP
MVLKILNNNERLQTISTVKMVIFGPYGIGKTSLLKTVDEPTLCLDFEAGL
LAVQDWQGSLRTWNEARDIACLIGAALKSDQAYSQRHHEHVSGKYKDLFS
EFSKYRCIFVDSITVASRLCLLWATEASSERSGKQDMRAAYGLLAQEMMA

>B3gp9_sigma-70_region_2_domain
MIKNIAIIVKNVKYQAYRLKLAKCFIDENNEDLEQELFCEIWPCLDQYDE
DKSSFNTFVARLTENKAINMLKKQQCAKRNINNHISIDVTELFESEITKR
IDVDYMISVLPKKWQNICEQLKFFNLHEVAKMNNVSRTTLNNIIKKIRAK
LSPIYYEGKKKN

>B3gp10_HP
MPLCRTGAKINNTDAIFNLLIFCYFFLRIILMQFKIIQFRFLEPILNGFQ
RMGSSDFLKLY

>B3gp11_HP
MLSITLSSVPNYWLPKSGIRNQESRISLYGGFSPCVPKTVPRNGISEVRQ
YGGF

>B3gp12_HP
MLTLDLGKQTGWTILNDGIVQSGSKSFHVSRFSGGGMQFLNFRNWLNSLK
YKFPGIEVVYFEEVRRHLGAHIYGGFLAHLSAWCEESNIPYQGVSVKTIK
RFITGKGNASKADVIEAVQEKGFCPTDDNEADSLALMFYVMNFSKDFNTL
EIS

>B3gp13_HP
MRITQAEWAREKGFSRQYVCSLVKKGIVELEDGLIDREQANEAVAAIRDP
SQPLRRKERGETLSTILLKTRIKNETERGKLLEAKVKAEIGKFVSIEEVK
TEAFNVARVVRNNLLNIPNRVSALLASLSETEKIHMALTEEITNSLEELS
NTNIKKILNIQ

>B3gp14_ankyrin_motif_protein
MTDNLSLELIKCLINQPGLDVNVRGLNGKTPLHCAIEFDELSMVDLLLTK
KNINPFVEDNDGKTSLDYAKEEKKAEILQALINNKYGSEQDSLLHLAAMI
GEVNAVRYLIRKGIGVNVRNALHHTPLHLAAGIGHAEVVKILIREGNAEI
EVFDARNQTPMHYAVNNKKLEIVKLLLELGANVNSARVGQNSMKLSPIHI
AVSNTNYDERDLCLDILKCLIKEPNAQVNLQDYENKTPLHYAERLKTIEV
LLTREDIDPLVKDDSGKTPFDYAKPEIKKILMNNKYGPEKNSLLHLAAQK
GVVDAILKEEIDINILNNKGHSPIYLAAEKGHLHVVKLLLKKGANYTPVL
HLAIKSNNLELLKVLFTEKNGALLCRDTVVNFPTLHNKYIAQREIADKRT
KKHNNIICIYTTVSAIAVAVYIGLITTTIGSAIIFATITGVIALIIALMI
SEMSKRYIEKEFQKKMFMELEECGGINSTVNNIEIEPIMSRCRQ

>B3gp15_phage_terminase_large_s
MIYARAFSEGLRPDPELKVSEWANEYRVLAATAASEPGKWRTERTPYLKE
IMDSLSPSSPIEKVVFMKGAQIGGTEAGNNWIGYIIDQTPGPMLVVQPTV
EMGKRWSKGRFAPLIESTPCLKSKVKDPRSRDSGNTVQSKEFPGGIVVIT
GANSSVGLRSMPVKYLFLDEIDAYPGDSGGEGDPVLLSIARTNTFARRKI
FLVSTPTIHGISRIEKEFEATDKRYFFVPCPHCNYYQVLKWSQIKWENND
SRTAHYVCTECSGKIENHQKTEMLERGEWRATEAKEGEKKGFHLSSLYSP
VGWYSWQQAVENFLHAKESEQLLKVWINTTLGETWVDKGEVPDWKQLFNR
REFFQIGTVPKGEVVLTAGVDVQKDRLEVEVVAWGKGRENWSIDYRVFEG
DTGSGEVWGKLSELLNHHFIGENGLEYMISMMAVDAGYATQEVYNWVRSH
QGSGRVMAVKGVNKALVPLSSPSRVDITVGGQKLKRGIKLWPVGVSILKS
ELFQLLNISKGEEGKALPGYCHFPEYAPEYFKQLTAEQLVSKVVKGYTKQ
EWQKVRERNEVLDCRIYARAASIALGIDRWPESKWNSLSEKPESKKPKKV
RQSKWLGNQNVQ

>B3gp16_HP
MYSEKYLTQVEKAIQKLQSGERVVSIAYGDHVVRYGEVQIKDLLELRQRI
KAGLKVAGMRPKRKIVFSTSKGIL

>B3gp17_phage_portal_protein_la
MLLKTFKQLFNKPKIKSSAWDAAGSGRRFFHFQPELGSINNLLSQSLGHL
RSRSRDMVRKNPYAANIIDTIVSNSIGTGIKPQSKAKNAEFRKKVQELWL
RWTDEADSSGVSDFYGLQALVCRSMIEGGECFVRLRTRKLEDRFSVPLQL

QVLESEHLDNKTNQTLANGNVIRNGIEFNRLGQREAYYLFKEHPGESMFG
ESVRVPANDVLHIYRPLRPGQIRGEPWLSNILLKLYELDQYDDAELVRKK
TAAMFAGFITRLDPEANIMGESESNEQGVALSGLEPGTMQLLDPGEDIKF
SEPSDVGGSYEAFIRQQLRAIAIGTGITYEQLTGDLTGVNYSSIRAGLIE
FRRRCAMLQHNIMVFQFCRPVWDRWLELALLSGELDIGEEWTKGKEGAKK
EVKWIPQGFDWVDPLKDQQAQQMAVRNGFKSRSEVVSEMGYDVEEIDQEI
AEDQRRASELGLSFDSDVTANQEVL

>B3gp18_putative_minor_capsid_p
MVEPRSFELLSLQAGKQLIFKNIKHAVRNSERGIIPIHGILTKKSETFDG
LLGMTSYEKIHEEIESALEDKSIETILLDIDSPGGEVNGVFDLADFIYES
RAKKRIIAIANDDAYSAAYAIASSAEKIFLTRTSGVGSIGVIASHIDQSG
FDEKQGIKYTTVFAGSRKNDLNPHEPITSESLENLKSEVNRLYGMLVELI
ARNRSLSVEAIKNTEAGLYFGEKAIEMGLADGMTILSSINKNRSITMNEQ
TTNDLETDNLTKYRTEVLELIRLCNISKMPEKIGEFIEQSVSVEQAREVL
MELLAERTKKTEILSAVPQNSGEELMMQVAKSRRS

>B3gp19_HP
MSCISEQNNLGDLLKYEVSSLYSRDQITVAKGQNIKLGAVVAKKTEDGLI
RVLNPAGTDGTQTAIGVIVSDVNAIENTKAVIITCVAILADHAVVWPANI
TEEQKAEAIKQLEGRGIIIRKGV

>B3gp20_HP
MQNPFTNTAFSMTALTNAMNILPINYGRVENLNLFPSRSVRFRHITIEEQ
NGVLSLLPTQVPGAPATVGKRGKRKVRTFTIPHIPHDDVVLPEEVQGIRA
FGSESELKALADVITDHLQLMRNKHAITLEHLRMGALKGIILDADGSELL
NLYNEFEITPKVVNFALGTATTDVKRKCLEVLRHIEDNLSGEYMTGIHAL
VSPEFFDALTSHTKVKEAYERWQEGAALRNDMRSGFAFCGITFEEYRGQA
TDPEGTVRRFIDKDTGHCFPLGTASTFTTYFAPADFNETVNTLGQPLYAK
QEPRRFDRGTDLHTQSNPLPMCHRPAILVKIVAT

>B3gp21_HP
MQENIKRLFKDCFAHLGKVALYESKDKSYMVQVLKQQPDKLYEIGEGQFV
GEMLFLEVSVFDVLRPMVGDIFVIDGCKYKVYSPPLRGNSGIVWNINCTV
LGEKDV

>B3gp22_HP
MFKIEVNENIERITHDIKAKVELAAVRALNKTALWLKSQAVREISEEKQI
RLKVIRKRLRIIKARKSTLKVLIRAYLYDINIKQAKIRTAFNDEFMATMP
RGYRGIFKRVGRTALPIQEVKLPLEPEASRIIENLVNYEVERIFEKYFTH
ELFYDGILD

>B3gp23_HP
MTAFWTNLHEAICNTLKAEIPAIQTCEVYPAIRKELIAPAVFIELSGFEK
GHDPGTEELALRAKFEARIVIDSTIENAPIIVRALAAEVARVVNKNTWKV
EKISPGEFISGGGDDFRPELDAYLVWMVEWVHEVHLGDSVWAESSIPPST
VYIGFTPDIGVANKHKYVKVGKNDE

>B3gp24_baseplate_assembly_prot
MNSFEDANLLIKLNNLIRVGTVEEVGCGTAKVRVKMIGNILTDWLPWVTA
RAGEDRSWSAPSIGEQVIVLSPSGEIAKGIVLPAIYYKKYPIPEGSKEEV
SGFVFKDGTVIYYDRNGHKFTISVKEEGALVFAVKETQMEVTSKEVILKT
GKSSSNITDEKIVLQVGNSSLEITESGIKLNAKKIDLN

>B3gp25_HP
MIGDIEYTSQTSELSGYESGCEDESRREEETRGSLLGQKRLSESDKDLSL
AKQGKVVRFGDMCNQHGDYLQSPGIGGSSNVFVNGRPVHRQGDEWSQHAS
TDPYKPPHTSQSLLEGSDSVFVNGKAIARTGDLISCGAVAEQGSNDVFAG

>B3gp26_baseplate_assembly_prot
MRGMNSETGKTISGIEHLKQSIVDILTTPIGARVMRRDYGSRLFELIDHP
IVPGFAQELYAAVAEALEKWERRFKLKRVQITEIKEGKVTLILEGIYLPN
GKPIRLDGIIV

>B3gp27_baseplate_J-like_protei
MKTPNIVETLKFEEIFSRMKEELVRRNASFSALTESDPAIKILEVAAWRE
LLLRQRINDAAQANLLAFARGNDLDHLAEFYGVLRKESENDESLRKRVKA
KIVGWSTAGSKEHYRYHALSADTRVKDAQVVSPIPGSVQISILSTENGGV

PSEELLEIVRNHVIRDDIRVLTDTLTVIGCGIIPITIHAKVHIYPTASKD
VIEAAKEQFIKGLESTKGLGWNVTRSWIIAHLFTEGIQNIELTEPIEDII
VQNNECVALSDLSVR

>B3gp28_HP
MILPPNASKQEQILTDIINYSFDANSLRGFKFNPQTEMLPWLVAEYGLGE
ILSWGKKATKSALKEWVSAKQNGIRFQRLRGTPQSLKMALKWANIDNITI
QEEPPGEHFAEFQIGISDVPNDFFVDSVIALAKLSAPARSRLMRIFNDYY
NAQRFMLDESIFGDLLSDYSGVKIAKEGPVLSFGRKNSFELKIGNPRFKF
GTFRSHYDRSYSNDLYRLDVATLGETEPHTKNYNGTYERNHEWYNLEALY
PLPQSLLPEIKFAKALIVLSDSWNLGDINACFASTTYEEIEHTFHLSEDK
LSEQIWNFQRTPILERFSITYYYEAKNFTDQKIIESNLVEYHVDCQNDSD
SKQKDPIHELENYVAAFYSGMVTWHEHRHLNRPWKDFDPIIAKKLDVFST
DLVYT

>B3gp29_HP
MSVLTQSGRAAIAASIKEQSIHLAWGSGDANWESSHKVEKVFVEDEIALD
HHTIKDVRVFTGQTTYQPSIDYIVDSSSGVIKRTENSSIRANSAVTIEYS
ESTPPEPITSQKLLNELGRRTADEVLFCTGDENGELITPSGRFRPSNVPT
NNLYLKFTFDFTDAANQVIRELGVMVGTKVKEGLPIGQRYFEPQDIENPG
ILLILEHTVPLIRTAATRETFSFVVTF

>B3gp30_HP
MTLNTYYNRFNPDKEYEKSLFLAGRGLQSAELNETQEYALSKLKGIGDAI
FRDGDVITGSNCIIDRDTGKATLEGGKIYLRGAVRKVEKEEFVIPLSTTV
RIGVYYVESTITELEDENLRDPAVGTRNYQEVGAARLKVSTIWGYQVESV
TVNSTNGEFYPIYNIENGVLIEHSPPPQANIVTTALARYDKEANGSYVVN
GLEVMFLHKEEKGEEGVKKGKKEIFVINEGKAHVDGYEIELPHSIRVSFD
EDPDIKSVESEPHTFQPNSQRVMELKVNDFPINEIKKVDITVQKTITVTH
GSYSGAIDPIPDSAVLEIIQIKQGNVIYENSIDYKLNAGNVDWSLPGKEP
APGSSYLITYRCRTHVSPEDISEQGCKVKGAVDNSLVLIDYTWKMPRYDL
ITIDSKGAVRRIKGIAHPWRPSMPKAPSGQLLLCYIHQTWKNGEKEGVKI
VNNAIHAVPMNELEAMKKGINDLYALVAEERLRSDANSREPTTKKGVFVD
SFFDDDMRDQGISQSAAIVNRELVLPIDVEVADIEKGGERYLLPYKLEPV
LEQLLQTKGEKINPYQAFDPVPAKVTLNKNIDHWMEVKTNWKSPITRAFS
VKETTELLSSNSYEAEFMREAVQDFEIEGFEPNEKLKEVKFDGIFIQPTA

>B3gp31_HP
MLTANNQGKLKGKVKVPANIPAGTKLVQFYGDKGSYGEATYTGKKTITIE
ERRRVIAARRVDPLAQTFTLNESRHIGGVELWFVNKGKKRVVVQIRETAV
GVPSQTVIAESYIEPKDIKIDGTATRITWSPVFCHAGEEYAIVLLTDDPG
TAVKIAELGKYDAVNSRWVTSQPYQVGVLLSSSNASTWTPHQNLDLTFRL
LAAKFSENSHVIDLGKVTANNTSDLIVLTNVEKVAFDTNVEFILTDEEGK
ENFLSDNLPLALRERLSGKLTVKANLKGSKEKSPVLYPGLQLVMGNLSEF
GDYVTRSITAGSNTKITITYDALIPGTADIKTYVEKNMGEWQLVNLTSGK
PIGENWVERTHVLSNFNANETRVKLVLSGTVVYRPKVKNLRVIVT

>B3gp32_HP
MTFDQTKRGYPLPYPENIAVEDVVRIRTTIKKIDEDIAKRENEHNQLKKA
FERLNFETFLNFWNDHC

>B3gp33_HP
MTIANEAIDALHQRIKDLAANSTPDQLAYLAKSLESMIGKKAISDIAQMT
KVKEIIDALQERLKDLAANSTPDQLAYLAKALESIIDKSAVSEIVQMTDG
KLKELLDSAKKHLTDLDNKKASSLAVISESEKQLLKRIDEKGTTNLSLLD
TRKNANIAAINSISNSHKDGLKGLVEDFRAVNNVPPGSSIIGEIETRIEN
ISNEVKTRDEQLKASLINEIRKRNMVEPGSLPFLFGVLGRKNNYFGHGTF
TTELGKWSSDITKTDCMLQLLAGSHTYDTDYVSFYRPRQLYFIEGNKGTF
IYGELCTKSFSGSYDEIYYYPYAALGVVFVKNTTNVNINKTMEFVGSSYS
STEYGGAGLFVGTPDNTNSNKSSISKIVWKNVYQYTSSDSKLAGSGNVEI
PAGKTVAILLYTSSYLYSRTQVSQGMLASNYVHNYGQFIQWGIYNIRSNF
LTTGLEVDVERTLRAWQCPGLDATHKIWN

>B3gp34_HP
MPIYIRFEHNKQVETTTLKSKPAGNDWYEAPENFEWQKSYCLTEGGEIAQ
RTKEDIQKELLDNAKLCAFDNIRTHFNNFTSRFSGHSHQKAKSYEIQAKT
AENILTTQESIDEKDAAIIEPLARVRGISVVEMAKIIEEKAEKAVKAIIK
CEELEDLAKRKIEEAKSEEELEDLLNNFKQKMQRNG

>B3gp35_HP
MAEEFLHGVNVIEVTSGAKTVRTAKSSVIGVIGTAPEADEQKFPLNSPVL
IAGSLKEAAKLGKSGTLPSAINGIFSQIGATVVVIRVEESEAIQNIIGGV
DEETGKYQGIQAFLSSESIIHVAPRILIAPQFTHQLPESENPKNPVVAAL
IGVAEKLRAIIVADGPNTNDEEAIKWRKSVGSSRVYVVDPWVKVFIEGKE
EILPSSPFVAGLIAKVDSEQGFWHSPSNKEINGIVGTSRPIDFTLGDRSS
RANYLNENEVTTIIHQNGYRLWGNRTCSNDSKWTFLSVKRTADLINDSLL
RAHLWAVDRNITKTYIDDVIEGVNSYLANLKAQGAIISGKCYATPELNTP
TNIASGKVYFDFEFTPPYPAEQITFRSHLVSGTIL

>B3gp36_phage_major_tail_tube_p
MLPKILKNFNVFVDGRGYAGKIDEITLPKLTIKTEEYRAGGMDIPINIDM
GMEKLEAELTFAEYDSELFRLFGLIDNNAVSLTLRGGLQGSNSEAESVVV
NLRGLFKELDFGSWKPAEKATLKCTVAANYYKLTIDGRELIEIDAENMIR
KIDGVDQMTSMRTALGI

>B3gp37_HP
MEKIKLTEPIKIDGILVSEITLRRPKVRDRLAVERMGNSDAEKEVALIAN
LASISREAVEELDLADYSKIQEVLQGFLSQQKS

>B3gp38_phage_tail_tape_measure
MKAISIVIGATLQSSFNSTIAGSTKQLFRIGSTIKQLESSSKSVSKFKEL
SHDALLARRSWNELEIKTKSLAKQIKNTEAPSKRLQNEFTRSKAAALKAK
TAYLQKRSALHLLRTEFNKSGRDIKSLVGDQAKLGSSIEKLKNNYTALNS
VMQKRKGVLAQRANFKAQMMDAVALGLTLAAPLKAAISFESAMADVKKVV
KFEDTDVNGLTKLGETLKEMSRTIPLSAAGLAQITASGGQLGIKAKDLTT
FTDTVAKMATAFDMSAEEAGDAIAKLSNIYQIEIGEMKGIGDAINHISDN
TAAKAKDIVPALNRIGGTARQFGLTAVEAGALASSFISLGKAPEKAGTAI
NAMLSKLQTASKQGGNFQKAFQMLKINANEFEKAIGKNAQGTLVKFLETI
AKLGKQERSSVLFDLFGLEYQDDIALLIGSLDEYKKSLRLINGEYKGSMQ
REFENRANTTANNLQLLKNSIAEVGMNLGSVLLPPLNFTVNLLKSATTQV
ALCAKEYPVLTTVVISTIATLISIKITAISLGYAWTFVKGSLFALLSTWR
VFASVITLVKIGLSAVFPAVIAGFKALTIALMSNPIGLIIGGLVIAATLI
ITKWQVVKNFFVTIWESVKIVWKSFSDWVGKFWNNITQPFKTINNLWSKK
NEARLEVRSVNNVISDSLKHPIAEHNKTIENKTHNNHFNINIHPSSGQDV
RSIADEVMERIREQFSGALYDIN

>B3gp39_F_protein
MLSLGPYRFSLTERGFTRRSEYRWPALERIGEKPLLQSIGPGEDVIELAG
VIYSHFSGGLKQINNMRNSSKPFLLIDGQGNILGHFVIVKIEETQKYFFP
NGEPKKTEFHLSLKHYDNALSDGSLNNPVDDIQLDDDVLQN

>B3gp40_phage_tail_protein_X
MLDLICWNYYGFSSGAVEIVLRANPGLAEYELLPAGLIIKLPVIQRTIQK
QVIKLWD

>B3gp41_phage_late_control_gene
MTPDFSISVEGISITELIKSRLVSMHITDEAGVISDTAVIHLDDRDSLFE
IPRTGAKLNILLGYKETGIMPMGDYIVNEITIQGPPQALKIKSHAADLKE
SLKEQVFNEWHQITLNDLVKKIASKHGYQAKVAAEFVNIMISHIDQTAES
DMHFLTRLAQIYGAIAKPAGGYLLFVSKGKAKSVTGKTLSTITLTPRDIT
NWKVKFNERNQYGSVIAYWYDYEKAETITEKVGDQEPSYILRDIYASSDL
AQSAASAKLNQLMSNAATLNVTMPGNPELFAEAKINLSGFRKGVYGK

>B3gp42_ankyrin_motif_protein
MKSSLFWKLLTSSIIPIICVIVLYQHFSTDQLSKDVPIKSKASGNDLETL
STAAEKCNLDTVRSLIKNVVDINATHHFGATSLHYATSGGCLEVVKFLIE
EGIDVNTTDAFSWTALHYAARKGHLEIAKFLLKNGANPSAKNKDKKTPLD
LAVEELNNNKEDIYEEIINLLSNYQLFP

>B3gp43_HP
MTNTGKIPVAVIATMVIQTIALIWWLAKLDLRVHMHDKFIEQNQRTTEII
YRLEERVKNLAEEVNELEQRQK

>B3gp44_patatin-like_protein"
MTKYILSVDGGGIRGIIPAIILAEIESRTKKPISQIFDLMAGTSTGGIVV
AGLCKSNKLQYSANDLVELYQEYGPYIFQASLWRKSIASWLSGSQYSYKN

MEFILNKYFGESTMADVASNLLLTSYDIHNSCEFFFKSWKEKNIKLKDAL
RATTAAPTYFTPKRLKISQTERVLIDGGVFANNPAACAYASGKRLFPNDE
IILLSIGTGGTDRSIKYANSRRFGKIGWVKPLLNVMFASGLDCVDYQLEQ
VMGDKYIRIQSQLKVASFEMDNITSKNIKCLQQEAKAIIESHQRVINDFC
AIKII

>B3gp45_SpvB-motif_protein
MEHNKDTSQKSTIEAKKLELPKGGGAIKGISEAFEANSFTGTGSFSIPIP
LPTCRNFSPSLSVDYSSGGGNGVFGVGFNVSIASIIRKTSKKIPTYDDED
IFVLTGADDLVAINQTSNSEYDITYYKPRVEGLFAKIERLISKNDNRVYW
QVTSKDGSIAIYGKNEQAQIYDPKSNRRIAEWLLEELSDVYGNKISYYYR
SSKVEQGSGRVIDRITYGHNKELKYNHFEVLFDYGENDFTSPIDKKYNEE
RKAIIRPDPFTSHITGFAVRTELLCRAIRIYHRFDAEPILVNGTYFDYDE
RPTITYLRAVKQVGYYEREGKRFTKTTPILVFSHTSDDSTYISTVSSVTL
SDGSKPSKELLQILKEQDFAFSEQNAEISEENSNWKIKDSEKTLVIKEKG
NFLDLYNSNNKNSFKPVKIQGFAGYLNDRRFQSVDLYQEGIEGILYNDGN
TILYFRPNGDGEFAQAKLLERFPSLLRKTTYSLTSLEGNGELQLYVNESG
FQGYHDFDSNSKWSEFKPLESQVINLSSEIQEMIDVTGDGRTDIVVFEGE
SVRVYPSKGKKGYKKAIYSKAPKDLPNQRASSESTLVRFADMFGDGKSHL
VKIENGKVECWPNLGYGKFGEKVTFKNAPYFRDGFNAKRLYFADIDGSGT
TDLVYANHNSLDIYYNNSGNSFSKPVTLKLPVDYNHIGRIEFADILGNGT
NCLLISYLDQSLKLQHLYYDFYNGVKPHLLKQIDNNMGNITRLHYAASTK
FYLEDRKENRPWVTSLPFPVQVIEKIETIDNIVGSKLTNKYRYHHGFYDY
TEKEFRGFGFVETWDTEDFSSSDQTEREHYVAPIYTKTWYHTGAYKKAED
LVESYRKEFYQGDREALKITPQVFDENIISIEDKRQAYRAIQGQMLRQEV
YGLDENPDLYQHPYSVTESSACVKLLQSSTNSSHKVFKYGIFFVNLQETI
NYHYERNCCDPRIQHDFVLEVDEYGNVIKSASITYPRRIEPNNYSEQQKL
HATLERNFYVNNTKEFYLLGALYKSQAFEIGGLRVAVSGYTNINDLRTHV
TQSLQNTIKFEENLNYTSPQVRLLSESHNYFWNKEQTKNLALGLVTQQAL
LHHTEAMAFSDSQVKEVFAEKIASDQMLKDLGYKKTSDFWWVPSPTQYYY
DSDKYYLPEKVIDVFGSETAVIYDQYNLLPIKTIVKATESSNYETTVEYD
YQRLTPVKLTDHNDNVSEVILNPLGVVVATSLYGSEKGEKKGDKPLLEYQ
VRKNPNFEDVLGLADRANGPEYYLQNATSFFYYDLDAWRKNGQPVHVINL
QRETHVSEGSFTRIRQIVTHIDGFGRNLETKMLTDSESEKWLASGRVVYN
NKGTEVKKYEPFYSNSPFYDLEESVRNQGVSDTLYYDPLLRNVHIDTAKG
FFSKVEFDSWSTKNFDLNDTVKDSTYYQEFTERWNSATEEEKIALKDEKD
ALSKAEKHYNTPTIEHLDSLGRKFLQIEILSNNEQSNVELKSHVKLDIQG
NELESVDSRFYEQNQGKSENEKIKNFVHIYSMAGLLKSISIDAGEAWNLA
NVAGSSVYNWDGKGSLTYTEYDKLQRPIKVKVTDQDLNLNGNIVEKFFYS
DDIQDKATNRYGTLVAHYDQAGLTEAKENDFKGQTLKSSYILRKNYKREA
NWQNVESITTTYADLEREIFNSSVKYNAIGEIIEKTDSQNNTHSPIYDIA
GRVKQIKLKKQEEAIKTYVSDISYNPKGQRTSITYGNGVVTSYSYDSKTF
RLIKLESVKEQKKLQSINYTYDPVGNITTMNDESYKTIFPNNKNIDTKSD
YHYDSLYRLTEATGKEHPALSGKEEIIAPIPHLNNQNAISNYTEYYDFDY
GSNITKIRHNGGNNSTKEFYISNKSNRSLPIIDRRSVNESNIDESHDERG
NLLKLHGSQNLHWNYRDNIAYVDIITRPSGKNDSEYYVYDSGGQRVRKVT
ETYQNNETSSTRVEKIYFGDIEVTRTYQESGLSKEKYTVHVMDDKSRVAL
HHYWTKGTSDETRESQSRYQLSNHLDSVALELNDNAEIMTYEEYLPFGGT
ALIAGKTVREVTEKEYRYSGKERDESTGLYYYGARYYAPWLARWINPDPA
GTVDGLNLYQFVGNNPINYVDSIGETKKRDRYNVKFNQQSKTSIAVSRRK
VGANFSLFEEVHESGGKGEKKLHAETSYITGQRSEGDLKDNIDKEIWKLD
RSPCQNCTREIIEYVKDRLEAQPNFKLSLGMENIYTKAEGIEKGKGSMEQ
ARRINATSLAAMKVIFPVALNLKYARMYENVENKENTKSGNPHPLVKQRQ
EFANDNLAQGHAENMKLVIDPLKTLAGKIEEIANGQPGDNIESIRGAIES
MVKSDNILKNLLQGKEGVMIDTWKSQGMTTVGSVKSTMKAILEGADNLIK
YSVPANDNQASSSNKKRKRAPGT

>B3gp46_HP
MNKNTANDSQLRLEKMGIENVEITEKLLSFKNENDEYLHDSAFKITDLCE
DVFIERVKDVITEQEAKDIYHKALARREFVSRYIENAREMTEPHYRALRV
CNIDHDTAEKCDAIIHSLQGE
