# Supplementary material for: The Genomic Architecture of Novel Simulium damnosum Wolbachia Prophage Sequence Elements and Implications for Onchocerciasis Epidemiology
Source: Front Microbiol. 2017 May 29;8:852. doi: 10.3389/fmicb.2017.00852 (PMC5447182; doi:10.3389/fmicb.2017.00852)
Supplement: Supplementary file 2 [file DataSheet2.PDF]

The Genomic Architecture of Novel *Simulium damnosum Wolbachia* Prophage Sequence Elements and Implications for Onchocerciasis Epidemiology

James Lee Crainey, Jacob Hurst, Poppy H.L. Lamberton, Robert A. Cheke, Claire E. Griffin, Michael D. Wilson, Cláudia Patricia Mendes de Araújo, María-Gloria Basañez, Rory J. Post

SUPPLEMENTARY FILE 2 | Primers used in gap-closing experiments

GAP1_gp1_int_f1:GGCCGtATAAGCGTAATGAG

GAP2_gp1_2_sOri_f1:gAAGAGAAAGAAAGAACGCTTG

GAP2_gp1_2_dOri_f1:TGTAGCCATTATCGACAAACC

GAP3_gp3_int_f1:CTTTCACGAGCAAATTGCTG

GAP4_gp4_int_f1:TGCTtCTTCTTGACCTAGCC

GAP5_gp5_int_f1:GCTCCACGAATTCTCACTCC

GAP6_gp6_7_f1:tcAAcaCATCAgAGaCAAaGaC

GAP7_gp7_8_f1:GATACGTGCCAAACTTTCTCC

GAP8_gp8_10_f1:TGCCATTCCTTTGGTAAAACC

GAP9_gp11_int_f1:CAACAACTGAAAGGAAATGCTC

GAP10_gp12_14_f1:GACAAGTAACCTTTCCACGATG

GAP11_gp14_16_f1:GCTCTAGTACcAcTTAGCAGTC

GAP12_gp16_int_f1:ACAATGCAGCTTTTAGACCC

GAP13_gp16_gp18_f1:TGGAGGAGAGGTCAATGGAG

GAP14_gp19_gp21_f1:GAACAGCAAGCACATTTACAAC

GAP15_gp23_gp24_f1:ACCATtGGGAGAaTTGTCTTTG

GAP16_gp25_gp26_f1:CAATTGTACCTGGTTTTGCAC

GAP17_gp27_gp28_f1:GGGATATCAATGCTTGTTTTGC

GAP18_gp28_gp29_f1:CAAGACACACCACCAGAAC

GAP19_gp29_int_f1:AAAAGGAAGAGGGGGAAGTAG

GAP20_gp31_gp32_f1:TTGTCACGTAGCAGAATGTC

GAP1_gp1_int_f2:AGCGTAATGAGTAATGCACc

GAP2_gp1_2_sOri_f2:CGTGAAAAGCAAGAAaGTAAGC

GAP2_gp1_2_dOri_f2:CGCTCCAATTCTACAATCTGAC

GAP3_gp3_int_f2:GTCTTAAAATCCTTTCACGAGC

GAP4_gp4_int_f2:CACCGAaTAAACCAaGgaG

GAP5_gp5_int_f2:ACTCCAGGTGGTCTTTTAGTC

GAP6_gp6_7_f2:CGGGAAAaCaGGACATGAG

GAP7_gp7_8_f2:GAACAACGTTTCAAGAACCAC

GAP8_gp8_10_f2:CGTTGTTCATTTTGGCAACTTC

GAP9_gp11_int_f2:GGTTTAGTGGTGGTGGAGTG

GAP10_gp12_14_f2:ATGGACTCATTGACCAAGAAC

GAP11_gp14_16_f2:AGCAGTCcAAGCaGAGTAG

GAP12_gp16_int_f2:CAGAACCATTGGATGTTGGAG

GAP13_gp16_18_f2:TGTTGGATATAGACAGTCCTGG

GAP14_gp19_gp21_f2:TTGCCCTAAACATCAGCAAATC

GAP15_gp23_gp24_f2:GGAAAAaTaCCCTCcTCCAGAG

GAP16_gp25_gp26_f2:CAATTGTACCTGGTTTTGCAC

GAP17_gp27_gp28_f2:CTCTACCACAGAGTTTATTGCC

GAP18_gp28_gp29_f2:AACCCTTCTAATGTGCCAAC

GAP19_gp29_int_f2:TTCCTCACAGTATTCGTGTTTC

GAP20_gp31_gp32_f2:TATCCTTTGCCACACCCAG

GAP1_gp1_int_r1:GCCAGAACAACGGTAGTAAG

GAP2_gp1_2_sOri_r1:TCTATCGTCATAATCTCCGTCC

GAP2_gp1_2_dOri_r1:TCTATCGTCATAATCTCCGTCC

GAP3_gp3_int_r1:GTTCTGCCATTCGCACTAC

GAP4_gp4_int_r1:AGCAAAATAACACCTGACTCC

GAP5_gp5_int_r1:GcCAACGcCCAtTTactCTG

GAP6_gp6_7_r1:CAAGATACTGCGAAGCATAGAG

GAP7_gp7_8_r1:GCTCTCATGTCCtGtTTTCCC

GAP8_gp8_10_r1:CACCATGCGTTCCTAAAACC

GAP9_gp11_int_r1:CCTGTACTGCCTCAATAACATC

GAP10_gp12_14_r1:CCTTCTCCTCCTGAATCTCC

GAP11_gp14_16_r1:GCTCAtTACtTTCACCTTCTCC

GAP12_gp16_int_r1:TCTTCAGCAATTTCCTGCTC

GAP13_gp16_18_r1:CCTATCGCTGTTTGTGTGTC

GAP14_gp19_gp21_r1:TTTCTTCACTGATTTCTCGGAC

GAP15_gp23_gp24_r1:CCATAATCTCGGCGCATAAC

GAP16_gp25_gp26_r1:ACCACTTGCACATCTTTTACTC

GAP17_gp27_gp28_r1:CTACACGCTCAATAGTGCC

GAP18_gp28_gp29_r1:AATATTGCATCGCCTATGCC

GAP19_gp29_int_r1:TCATACGGCAACAAATGAGG

GAP20_gp31_gp32_r1:ACGCTTTTAACTGTTCATCCC

GAP1_gp1_int_r2:ATTGTCACATACTCTGTTGCC

GAP2_gp1_2_sOri_r2:CTACCAGAAAGAGAATCAAGCC

GAP2_gp1_2_dOri_r2:CTACCAGAAAGAGAATCAAGCC

GAP3_gp3_int_R2:TGAGAACTGTTGGATGGACTC

GAP4_gp4_int_r2:CATTTACGAGAGCAGAAGTACC

GAP5_gp5_int_r2:TAGAGTTTTGcCAACGcCC

GAP6_gp6_7_r2:TTACGAGCAAGGATTGCAC

GAP7_gp7_8_r2:GAGCaAGTaATCCATAGGCAG

GAP8_gp8_10_r2:TGCGTTCCTAAAACCGTTC

GAP9_gp11_int_r2:TGGACAAAAACCCTTTTCCTG

GAP10_gp12_14_r2:TGCTACACTGCTATTTGCTC

GAP11_gp14_16_r2:AACCAGATAGTGCCACTCC

GAP12_gp16_int_r2:CGTCAGAATCAAAACTTAAGCC

GAP13_gp16_18_r2:CCATACAACTGCATGATCGG

GAP14_gp19_gp21_r2:GCTCTTACTGCTGCCAGTTC

GAP15_gp23_gp24_r2:GCAACTGCTGCATAAAGTTC

GAP16_gp25_gp26_r2:ACCACTTGCACATCTTTTACTC

GAP17_gp27_gp28_r2:TGCTATCTGCTATAATTGCACC

GAP18_gp28_gp29_r2:TTCGACTTTTCTAACTGCTCC

GAP19_gp29_int_r2:TTTCCAATTCGTGGTAACCTC

GAP20_gp31_gp32_r2:ACTACTCCACCTTTCAAGCTC
