# Supplementary material for: The Genomic Architecture of Novel Simulium damnosum Wolbachia Prophage Sequence Elements and Implications for Onchocerciasis Epidemiology
Source: Front Microbiol. 2017 May 29;8:852. doi: 10.3389/fmicb.2017.00852 (PMC5447182; doi:10.3389/fmicb.2017.00852)
Supplement: Supplementary file 3 [file DataSheet3.PDF]

The Genomic Architecture of Novel Simulium damnosum Wolbachia Prophage Sequence Elements and Implications for Onchocerciasis Epidemiology

James Lee Crainey, Jacob Hurst, Poppy H.L. Lamberton, Robert A. Cheke, Claire E. Griffin, Michael D. Wilson, Cláudia Patricia Mendes de Araújo,
María-Gloria Basañez, Rory J. Post

SUPPLEMENTARY FILE 3| CLUSTAL W (1.60) multiple sequence alignment of WO sernine recombinase/integrase aminoacid sequences

```
WOcauB2         SLYARVSSRQ-QAQE-NTIESQIVELERRISSDGHELLDEHKFVDNG-YSGSNLERPGLE
WOcauB3         SLYARVSSRQ-QAQE-NTIESQIVELERRIGSDGHELLDEHKFIDNG-YSGSSLERPGLE
WOdamA1         SLYARVSSRQ-QAQE-NTIESQIVELERRISSDGHELLDEHRFVDNG-YSGSNLERPGLE
WOHaA1          -LYARVSSKS-QAQN-NTIESQIAELKHRIAADKHELLNEYEFKDDG-LSGWSLEREGLD
WOMelA          AIYTRKSNEDGLEQKFNSLDAQRVACKKYIKSREGWVALAKRY-DDGGYSGSNLNRPAIK
WOMelB          AIYTRKSNEDGLEQKFNSLDAQRVVCEKYIKSREGWVALAKKY-DDGGFSGSNLNRPAIK
WOPip2          AIYTRKSNEDGLEQKFNSLDAQRVACEKYIKSREGWVALAKRY-DDGGYSGKNLERPAIK
WOPip3          AIYTRKSNEDGLEQKFNSLDAQRVACEKYIKSREGWVALAKRY-DDGGFSGKNLERPAIK
WOPip4          AIYTRKSNEDGLEQKFNSLDAQRVACEKYIKSREGWVALAKRY-DDGGYSGKNLERPAIK
WORiA1          AIYTRKSNEDGLEQKFNSLDAQRVVCEKYIKSREGWVALAKKY-DDGGFSGSNLNRPAIK
WORiC           ALYARVSSKK-QAQN-NTIESQIAELKCRIAADKHELLDEYEFKDNG-ISGWILGREGLE
WORiB           SLYARVSSRQ-QAQE-NTIESQIVELERRISSDGHELLDEHRFVDNG-YSGSNLERPGLE
WOVitA1         ALYARVSSKS-QAQN-NTIESQIAELKHRIAADKHELLNEYEFKDDG-LSGWSLEREGLD
WOVitA2         SLYARVSSRQ-QAQE-NTIESQIVELERRISSDGHELLDEHKFVDNG-YSGSNLERPGLE
WOVitA4         AIYTRKSNEDGLEQKFNSLDAQRVACEKYIKSREGWVALAKKY-DDGGFSGKNLERPAIK


WOcauB2         NLRDRVAEGKIDKIYIHSPDRLSRKFSYQMILLEEFKKAGSEIVFLNHKFDDN-PDSHL-
WOcauB3         SLRDRVAEGKIDKIYIHSPDRLSRKFSYQMILLEEFKKAGSEIVFLNHKFDDN-PDSHL-
WOdamA1         SLRDRVAEGKIDKIYIHSPDRLSRKFSYQMILLEEFKKAGSEIVFLNHKFDDN-PDSHL-
WOHaA1          ALRDKVGEDQIDKIYIHSPDRLSRKSAHQMILLDEFEKAGVEVIFLNHKTENN-PESKLL
WOMelA          ELFEDVKAGEVDCVVVYTLDRLSRETKDCIEVTSFFRRHRISFVAVTQIFDNNTPMGK--
WOMelB          ELFEDVKVGEVDCVVVYTLDRLSRETKDCIEVTSFFRRHRISFVAVTQIFDNNTPMGK--
WOPip2          ELFEDVKAGEVDCVVVYTLDRLSRETKDSIEVTSFFRRHRVNFIAVTQIFDNNTPMGK--
WOPip3          GLFEDVKTGEVDCVVVYTLDRLSRETKDSIEVTSFFRRHRVNFVAVTQIFDNNTPMGK--
WOPip4          ELFEDVKAGEVDCVVVYTLDRLSRETKDSIEVTSFFRRHRVNFIAVTQIFDNNTPMGK--
WORiA1          ELFEDVKVGEVDCVVVYTLDRLSRETKDCIEVTSFFRRHRISFVAVTQIFDNNTPMGK--
WORiC           ALRDKVAEGEIDKIYIHSPDRLSRKSAHQVVLLEEFEKAGVEVIFSNHKIENN-PESKLL
WORiB           SLRDRVAEGKIDKIYIHSPDRLSRKFSYQMILLEEFKKAGSEIVFLNHKFDDN-PDSHL-
WOVitA1         ALRDKVGEDQIDKIYIHSPDRLSRKSAHQMILLDEFEKAGVEVIFLNHKTENN-PESKLL
WOVitA2         NLRDRVAEGKIDKIYIHSPDRLSRKFSYQMILLEEFKKAGSEIVFLNHKFDDN-PDSHL-
WOVitA4         ELFEDVKKGEVDCAVVYTLDRLSRETKDSIEVTSFFRRHRVNFVAVTQIFDNNTPMGK--


WOcauB2         -FLQ--IQGAIAEYERAKIMERNRRGKLHAAKAGCISVMGRA--PYGYRYIAKHVGEGSA
WOcauB3         -FLQ--IQGAIAEYERAKIMERNRRGKLHAAKAGCISVMGRA--PYGYRYIAKHVGEGSA
WOdamA1         -FLQ--IQGAIAEYERAKIMERNRRGKLHAAKAGCISVMGRA--PYGYRYIAKHVGEGSA
WOHaA1          LGMQGLV----AEYECTKIMERSRRGKLHRAKKGCVSVIGIA--PFGYNRI-KHVDREKT
WOMelA          -FVQTVLSGAAQ-LEREMIVERVKN-KIATSKEEG-LWMG-GNVPLGYD-V-KEKELIIN
WOMelB          -FVQTVLSGAAQ-LEREMIVERVKN-KIATSKEQG-LWMG-GNPPLGYD-V-KEKELIIN
WOPip2          -FVQTVLSGAAQ-LEREMIVERVKN-KIATSKEQG-LWMG-GTLPLGYD-V-KDKELIIN
WOPip3          -FVQTVLSGAAQ-LEREMIVERVKN-KIATSKEQG-LWMG-GTLPLGYD-V-KDKELIIN
WOPip4          -FVQTVLSGAAQ-LEREMIVERVKN-KIATSKEQG-LWMG-GTLPLGYD-V-KDKELIIN
WORiA1          -FVQTVLSGAAQ-LEREMIVERVKN-KIATSKEQG-LWMG-GTLPLGYD-V-KDKELIIN
WORiC           LGMQGLL----SEYECTKTMERSRRGKRHRARKGCISVIGIA--PFGYKRM-KHVDREKT
WORiB           -FLQ--IQGAIAEYERAKIMERNRRGKLHAAKAGCISVMGRA--PYGYRYIAKHVGEGSA
WOVitA1         LGMQGLV----AEYECTKIMERSRRGKLHRAKKGCVSVIGIA--PFGYNRI-KHVDREKT
WOVitA2         -FLQ--IQGAIAEYERAKIMERNRRGKLHAAKAGCISVMGRA--PYGYRYIAKHVGEGSA
WOVitA4         -FVQTVLSGAAQ-LEREMIVERVKN-KIATSKEQG-LWMG-GTLPLGYD-V-KDKELIIN


WOcauB2         QFEVDEEEANIVRKIFSRVGQERASIGEVVHELNKIPVITRTGK-RYWKRSTIWNMLKNP
WOcauB3         QFEVDEEEANIVRKIFSRVGQERASIGEVVHELNKIPVITRTGK-RYWKRSTIWNMLKNP
WOdamA1         QFEVDEEEANIVRKIFSRVGQERASIGEVVHELNKIPIITRTGK-RYWKRSTIWNMLKNP
```

```
WOHaA1          KFEINEEEAKIVKQIFMWVGQERISIREVIRRLRDKSIRTRTGK-KVWCPIIIWKVLRNP
WOMelA          EKE-----AKVIKHIFERY-MELRSMAELARELNREGYRTK-AKSDIFKKATVRRIITNP
WOMelB          EKE-----AKIIKHIFERY-MELKSMAELARELNREGYRTK-AKSDIFKKATVRRIITNP
WOPip2          GKE-----AKTVKHIFERY-MELKSMAELARELNSQGYRTK-ARFDIFKKATVRRIITNP
WOPip3          EKE-----AKIVGHIFERY-LELKSMAELARELNSQGYRTK---SDIFKKATVRRIITNP
WOPip4          GKE-----AKTVKHIFERY-MELKSMAELARELNSQGYRTK-ARFDIFKKATVRRIITNP
WORiA1          EKE-----AKTVKHIFERY-VELKSMAELARELNREGYRTK-AKSDIFKKATVRRIITNP
WORiC           SFEINEEEAKIVRQIFMWIGQERISIREAIRRLKDMSVRTRTEK-KVWRPIIIWKMLRNP
WORiB           QFEVDEEEANIVRKIFSRVGQERASIGEVVHELNKIPIITRTGK-RYWKRSTIWNMLKNP
WOVitA1         KFEINEEEAKIVKQMFMWVGQERISIREVVRRLRDKSIRTRTGK-KVWCPIIIWKLLRNP
WOVitA2         QFEVDEEEANIVRKIFSRVGQERASIGEVVHELNKIPVITRTGK-RYWKRSTIWNMLKNP
WOVitA4         EKE-----AKTVKHIFERY-LVLKSMAELARELNREGYRTK-AKSDIFKKATVRRIITNP


WOcauB2         AYIGQAAYGKTRTCSKPQVKKSKKGTCGKLKSGRFNSDKENWTYIPVPKIINEHLFDSVQ
WOcauB3         AYIGQAAYGKTRTCSKPQVKKSKKGTCGKLKSGRFNSDKENWTYIPVPKIINEHLFDSVQ
WOdamA1         AYIGQAAYGKTRTCSKPQVKKSKKGTCGKLKSGRFNSDKENWTYIPVPKIINENLFDSVQ
WOHaA1          AYKGQAAFGKLKRVE--KRERNKQKVS------ISRTDEDSWIYIPVPKIVDEGLFNKVQ
WOMelA          IYM-----GKIRHYE--KQYKGKHEAIIEEEKWQKAQELIKNQPYRGKKY-EEAL-LK--
WOMelB          IYM-----GKIRHYE--KQYKGKHEAIIEEEKWQKAQELISNQPHRKAKY-EEAL-LK--
WOPip2          IYM-----GKIRHYE--KEYEGKHEAIIEEEKWQKAQELIRNQPYRKAKY-EEAL-LR--
WOPip3          IYM-----GKIKHYE--KEYEGKHEAIIEEEKWQKAQELIRNQPYRKAKY-EEAL-LR--
WOPip4          IYM-----GKIRHYE--KEYEGKHEAIIEEEKWQKAQELIRNQPYRKAKY-EEAL-LR--
WORiA1          IYM-----GKIRHYD--KQYEGKHEAIIEEEKWQKAQELIRNQPYKKAKY-EEAL-LR--
WORiC           TYKGQAAFGKLKRVERISRKKSKQKVKNVY---VYRTDEESWIYIPVPKIVDEGLFNKVQ
WORiB           AYIGQAAYGKTRTCSKPQVKKSKKGTCGKLKSGRFNSDKENWTYIPVPKIINENLFDSVQ
WOVitA1         AYKGQAAFGKLKRVE--RRERNKQKVS------ICRTDEDSWIYIPVPKIVDEGLFNKVQ
WOVitA2         AYIGQAAYGKTRTCSKPQVKKSKKGTCGKLKSGRFNSDKENWTYIPVPKIINEHLFDSVQ
WOVitA4         IYM-----GKIRHYE--KQYEGKHEAIIEEEKWQKAQELIKKQPHRGAKY-EEAL-LR--


WOcauB2         AQLTENRQR-ARVRQR-----RETYLLQGLMVCQRCQYTYCGTNHVHKKSTYYYYRCSGT
WOcauB3         AQLTENRQR-ARVRQR-----RETYLLQGLMVCQRCQYTYCGTNHVHKKSTYYYYRCSGT
WOdamA1         TQLAENRQR-ARVRQR-----RETYLLQGLMVCQRCQYTYCGTNHVHKKSTYYYYRCSGT
WOHaA1          KQLDENRKR-ARMQR--EGGKKK-YLLQGLVVCQNCGYAYSGAQCGVEGKKFSYYRCS-S
WOMelA          GMI---RCRCCKVNMTLTYSKKE-NKRYRYYICNNHLRGKGCKSMNRTVVAGEVEKEVMK
WOMelB          GII---KCKSCDVNMTLTYSKKE-NKRYRYYVCNNHLRGKNCESVNRTIVAGEIEKEVMK
WOPip2          GII---KCKSCDVNMTLTYSKKE-NKRYRYYICNNHLRGKSCESVNRTVVAGKVEKEVMK
WOPip3          GII---KCKSCDVNMTLTYAKKE-NKRYRYYVCNNHLRGKGCESENRNVIAGEVEKEVMR
WOPip4          GII---KCKSCDVNMTLTYSKKE-NKRYRYYICNNHLRGKSCESVNRTVVAGKVEKEVMK
WORiA1          GII---KCKSCDVNMTLTYAKKE-NKRYRYYVCNNHLRGKNCESVNRTIVAGEVEKEVMR
WORiC           KQLDENRKR-ARMQRR----EEIN-LLQGLTICQNCKSTYSGVHHRDGEKTYSYYRCS-S
WORiB           TQLAENRQR-ARVRQR-----RETYLLQGLMVCQRCQYTYCGTNHVHKKSTYYYYRCSGT
WOVitA1         KQLDENRKR-ARIQR--EGGKKK-YLLQGLVVCQNCGYAYSGAQCGVEGKKFSYYRCS-S
WOVitA2         AQLTENRQR-ARVRQR-----RETYLLQGLMVCQRCQYTYCGTNHVHKKSTYYYYRCSGT
WOVitA4         GII---KCKSCEVNMTLTYSKKE-NKRYRYYICNNHLRGKNCESINRTIVAGEVEKEVMK


WOcauB2         NSSKFNGNKICDNKSIRTDILDGVIWEEVKSILKEPDRIANEYQRRLSENK-------KP
WOcauB3         NSSKFNGNKICDNKSIRTDILDGVIWEEVKSILKEPDRIANEYQRRLLENK-------KP
WOdamA1         NSSKFNGNKICDNKSIRTDVLDTVVWEEVKSILKEPDRIANEYQRRLSENK-------KP
WOHaA1          TIRITDGREKCTNKLVRTDMLETAIWEKVKNLLKNPEIIKNEYHRRTAENKNDESSDKKF
WOMelA          RAECLYENWEKGAKKEKWENLSFG---KQKEAVK--KLIKTVWVREDGIEV-CSESEEK-
WOMelB          RAECLYG---------DGENLSFR---EQKEAMK--KLIKGVMVKEDGIEV-CSESEEK-
WOPip2          RAEDLYEKCG-----EEWKNLSFG---KQKEVVK--KLIKGVMVKEDGIEV---SSEDKV
WOPip3          KTEQLYGKLGEKA--EEWKNLSFG---KQKEVVK--KLIKGVMVREDGIEV---SLEDKV
WOPip4          RAEDLYEKCG-----EEWKNLSFG---KQKEVVK--KLIKGVMVKEDGIEV---SSEDKV
WORiA1          KTEQLYENWKEKT-EEEWKNLSFG---KQKEVVK--KLIKGVMVKGDGIEV---SSEDKI
WORiC           IVRITDDEEKCNNKLVRADMLEIAVWEKVKDVLKNPEMIKKEYQRRVLENKNDESSEKKF
WORiB           NSSKFNGNKICDNKSIRTDVLDTVVWEEVKSILKEPDRIANEYQRRLSENK-------KP
WOVitA1         TIRITDGREKCTNKLVRTDMLETAIWEKVKNLLKNPEIIKNEYHRRIAENKNDESSDKKF
WOVitA2         NSSKFNGNKICDNKSIRTDILDGVIWEEVKSILKEPDRIANEYQRRLSENK-------KP
WOVitA4         KAEQLYENWKEKR-EEKWKNLSFG---KQKEVVR--KLIKGVMVKEDGIEV---SSESKV


WOcauB2         LHNQTREKQESKLRLSIKKFIDSYA--KGFISQEEFEPRITTMKQHLKEIEEEK------
WOcauB3         LHNQIREKQESKLRLSIKKFIDSYA--KGFISQEEFEPRITTMKQHLKEIEEEQ------
WOdamA1         LHNQTREKQESKLRLSIKKFIDSYA--KGFISQEEFEPRITTMKQHLKEIEEEK------
```

```
WOHaA1          ARRENQIKQGIEKLMEDYYSQENVG-DKGYISEEEFKQTMKRMRERLRGIEEEK------
WOMelA          --FIPMKKKGNKCTVVEPEGKTNNALLKAVV----------RAHLWKRQLEEGKYRSVKE
WOMelB          --FIPMKKKGNKCIVIEPEGKTNNALLKAVV----------RAHSWKRQLEEGKYRSVKE
WOPip2          -EFIPIKKKGNKCTVVEPEGKTNNALLKAVV----------RAHLWKRQLEEGKYANIKE
WOPip3          -EFIPIKKKGKKCTVIEPEGKTNNALLKAVV----------RAHLWKRRECSKKCVKPHF
WOPip4          -EFIPIKKKGNKCTVVEPEGKTNNALLKAVV----------RAHLWKRQLEEGKYANIKE
WORiA1          -EFIPIKKKGNKCVVMEPEGKTNNALLKAVV----------RAHLWKRQLEEGKYGSIKE
WORiC           ARRKNQIKEGIEKLMEDYYSQENAG-EKGYISEEEFKQTMKKMKERLQGIEEEK------
WORiB           LHNQTREKQESKLRLSIKKFIDSYA--KGFISQEEFEPRITTMKQHLKEIEEEK------
WOVitA1         ARRENQIKQGIEKLMEDYYSQENVG-DKGYISKEEFKQTMKRMRERLRGIEEEK------
WOVitA2         LHNQTREKQESKLRLSIKKFIDSYA--KGFISQEEFEPRITTMKQHLKEIEEEK------
WOVitA4         -EFIPIKKKGNKCTVIEPEGRTNNALLKAVV----------RAHLWKRQLEEGKYRSVKE


WOcauB2         ---ERTLDQKKLQQELSL-
WOcauB3         ---ERTLDQKKLQQELSL-
WOdamA1         ---EKVLDQKKLQQELSL-
WOHaA1          ---KKVVDQKAIEKGMNL-
WOMelA          LSIKINIGTRRIQQILRL-
WOMelB          LSKKINVGTRRIQQILRL-
WOPip2          LSAKVNIGTRRIQQILRL-
WOPip3          L------------------
WOPip4          LSAKVNIGTRRIQQILRL-
WORiA1          LSAKINIGARRIQQILRL-
WORiC           ---KKVVDQKAVEKGISL-
WORiB           ---EKVLDQKKLQQELSL-
WOVitA1         ---KKVADQKAIEKGMNL-
WOVitA2         ---ERTLDQKKLQQELSL-
WOVitA4         LSAKINIGTRRIQQILRL-
```
