# Supplementary material for: The Genomic Architecture of Novel Simulium damnosum Wolbachia Prophage Sequence Elements and Implications for Onchocerciasis Epidemiology
Source: Front Microbiol. 2017 May 29;8:852. doi: 10.3389/fmicb.2017.00852 (PMC5447182; doi:10.3389/fmicb.2017.00852)
Supplement: Supplementary file 4 [file DataSheet4.PDF]

Supplementary File 4_Final.txt

The Genomic Architecture of Novel Simulium damnosum Wolbachia Prophage Sequence Elements and Implications for Onchocerciasis Epidemiology

James Lee Crainey, Jacob Hurst, Poppy H.L. Lamberton, Robert A. Cheke, Claire E. Griffin, Michael D. Wilson, Cláudia Patricia Mendes de Araújo, María-Gloria Basañez, Rory J. Post

SUPPLEMENTARY FILE 4| CLUSTAL X (1.83) multiple sequence alignment of WO minor capsid gene sequences

```
WOcauB2         ATGGTAGAACCAAGGAGTTTTGAGTTACTGTCACTACAAACAGGAAAGCAGCCTATCTTT
WOcauB3         ATGGTAGAACCAAGGAGTTTTGAATTACTGTCACTACAAGCAGGAAAGCAGCTTATCTTT
WOdamA1         ATGGTAGAACCAAGAAGTTTTGAATTACTGTCACTACAAACAGGAAAGCAGCCTATCTTT
WOHaA1          ATGCTTGAACCAAGGAGTTTTGAGCTATTGTCATTAAACAATCAAAAACATCCCACGTTT
WOMelA          ATGATAGAGCAAAGGAGTTTTGAATTATTGTCATTACATGCTGGAAAACACCCTACGTTT
WOMelB          ATGGTAGAACCAAGGAGTTTTGAATTACTGTCACTACAAACAGGAAAGCAGCCTATCTTT
WOPip1          ATGGTAGAGAGAAGAAGCTTTGAACTATTATCATTATATAATAGCAAACAACCTATCTTT
WOPip2          ATGGTAGAGAGAAGAAGCTTTGAACTATTATCATTATATAATAGCAAACAACCTATCTTT
WOPip3          ATGGTAGAGAGAAGAAGCTTTGAACTATTATCATTATATAATAGCAAACAACCTATCTTT
WOPip4          ATGATAGAGCAAAAAAGCTTTGACCTGTTGTCACTATATAAGGGAAAACAACCTATCTTG
WOPip5          ATGATAGAGCAAAAAAGCTTTGACCTGTTGTCACTATATAACGGAAAACAACCTATCTTT
WORiA           ATGGTAGAACCAAGGAGTTTTGAATTACTGTCACTACAAACAGGAAAGCAGCCTATCTTT
WORiB           ATGGTAGAACCAAGGAGTTTTGAATTACTGTCACTACAAGCAGGAAAGCAGCCTATCTTT
WORiC           ATGATAGAGCAAAGAAGCTTTGATTTATTGTCATTACATGCAGAGAAGCATCCAACGTTT
WOVitA1         ATGGTAGAGAGAAGAAGCTTTGAACTACTATCATTATATAACAGCAAACAACCTATCTTT
WOVitA2         ATGGTAGAGAGAAGAAGCTTTGAACTACTATCATTATATAACAGCAAACAACCTATCTTT
WOVitA4         ATGGTAGAACCAAGGAGTTTTGAGTTACTGTCACTACAAACAGGAAAGCAGCCTATCTTT
                *** * **   **  ** *****  *  * *** ** *       ** ** *  *  **

WOcauB2         AAAAATATAAAACATGCAGTAAGAAATAGTGAAAGAGGAA------TAATACCGATACAT
WOcauB3         AAAAATATAAAACATGCAGTAAGAAATAGTGAAAGAGGAA------TAATACCGATACAT
WOdamA1         AAAAATATAAAACATGCAGTAAGAAATAGTAAAAGAGGAA------TAATACCGATACAT
WOHaA1          AAAAACATAAAGCACTCCATCAAAAGTAACGTAGAAAGAACTGCAATTATAGCAATACAT
WOMelA          AAAAACATAAAACACACTGCAGAAAACAACGTAGAAAAAATAGCAATAATACCAATACAT
WOMelB          AAAAATATAAAACATGCGGTAAGAAATAGTGAAAGAGGAA------TAATACCAATACAT
WOPip1          AAGAACTTAAAGCATTT--TCATATA-AACCCAAAAGGAATAGCAATAATACGTATTTAT
WOPip2          AAGAACTTAAAGCATTT--TCATATA-AACCCAAAAGGAATAGCAATAATACGTATTTAT
WOPip3          AAGAACTTAAAGCATTT--TCATATA-AACCCAAAAGGAATAGCAATAATACGTATTTAT
WOPip4          AAAAATATAAAACATACAGTAAATCAGAATATAGAAAAAACAGCAGTAATAGCAATACAT
WOPip5          AAAAAGATAAAACATGCAGTAAATCAGAATATAGAAAAAACAGCAGTAATAGAAATACAT
WORiA           AAAAATATAAAACATGCGGTAAGAAATAGTGAAAGAGGAA------TAATACCAATACAT
WORiB           AAAAATATAAAACATGCAGTAAGAAATAGTGAAAGAGGAA------TAATACCGATACAT
WORiC           AAAAATATAAAACATGCAGTAAGAAATAGTGAAAGGGGAATAAAAGTAATACCAATACAT
WOVitA1         AAGAACTCAAAGTATTT--TCATATA-AACCCAAAAGGAATAGCAATAATACGTATTTAT
WOVitA2         AAGAACTTAAAGCATTT--TCATATA-AACCCAAAAGGAATAGCAATAATACGTATTTAT
WOVitA4         AAAAATATAAAACATGCAGTAAGAAATAGTGAAAGAGGAA------TAATACCGATACAT
                ** **   ***  *             *    *     **      * ***   **  **

WOcauB2         GGCATTTTAACTAAAAAACCTGGTGCATTTGATGAAATGCTCGGAATGACATCATATGAG
WOcauB3         GGAATCTTGACAAAAAAGTCAGAAACTTTTGATGGTTTACTGGGTATGACATCATATGAA
WOdamA1         GGAATCTTGACAAAAAAGTCAGAAACTTTTGATGATATTTTTGGAATGACTTCCTATAGT
WOHaA1          GGAATCTTGACTAAAAAACCAGGTGCGTTTGATGAAATGCTCGGGATGACATCATATGAG
WOMelA          GGAATTTTGACCAAGAAACCAGGAGCTTTTGATGACATGTTGGGAATGACATCATATGAG
WOMelB          GGAATCTTGACGAAAAAGTCAGAAGTTTTTGATGATGTATTGGGGATGACATCGTATGAG
WOPip1          GGAGTTTTGACAAAAAAAAACAGAAGCTTTTGATCATATTTTAGATATGACTTCGTATGAA
WOPip2          GGAGTTTTGACAAAAAAAAACAGAAGCTTTTGATCATATTTTAGATATGACTTCGTATGAA
WOPip3          GGAGTTTTGACAAAAAAAAACAGAAGCTTTTGATCATATTTTAGATATGACTTCGTATGAA
WOPip4          GGAATTTTGACCAAAAAACCAGGAGCTTTTGACGTCTTTCTGGGAATGACATCATATGAG
WOPip5          GGAATTTTGACCAAAAAACCAGGAGCTTTTGACGTCTTTCTGGGAATGACATCATATGAG
WORiA           GGAATCTTGACGAAAAAGTCAGAAGTTTTTGATGATGTATTGGGGATGACATCGTATGAG
WORiB           GGAATCTTGACAAAAAAGTCAGAAACTTTTGATGGTTTACTGGGTATGACATCATATGAA
WORiC           GGAATCTTGACGAAGAAGTCAGAGGCTTTTGATGATGTGTTGGGGATGACATCGTATGAG
WOVitA1         GGAGTTTTGACTAAAAAAACAGAAGCTTTTGATCATATTTTAGACATGACTTCGTATGAA
WOVitA2         GGAGTTTTGACAAAAAAAAACAGAAGCTTTTGATCATATTTTAGATATGACTTCGTATGAA
WOVitA4         GGCATTTTAACTAAAAAACCTGGTGCATTTGATGAAATGCTCGGAATGACATCATATGAG
                **  * ** ** ** **  * *     *****     *  * *  ***** ** ***
```

```
WOcauB2         CAAATAGAAGAACAAATTACACAAGCATTAGCAGATAGTAGCATAGAGACAATTATACTG
WOcauB3         AAAATTCATGAAGAGATAGAGAGCGCTTTAGAAGATAAAAGCATAGAGACGATTCTACTT
WOdamA1         AAAATAGAAGCACAAATTACACAAGCAATAGAAGATAGTGATATAGAAACAATCCTGTTG
WOHaA1          CAAATAGAAGAACAAATTAAACAAGCATTAGCAGATAGTAGCATAGAGACAATTATGCTG
WOMelA          AAGATACGTGAAGAGATAGAAAAAGCTTTAATAGATGAAGAAGTAGAAACAATAATTTTG
WOMelB          AAGATAAGTGAAGAGATAGAAGAAGCTTTAATAGATAAAGAAGTAGAAACAATAATTTTG
WOPip1          AATATTCATGAAGAGATAGAGAGTGCTTTAGAAGATAAAAGCATAGAGACGATTCTACTT
WOPip2          AATATTCATGAAGAGATAGAGAGTGCTTTAGAAGATAAAAGCATAGAGACGATTCTACTT
WOPip3          AATATTCATGAAGAGATAGAGAGTGCTTTAGAAGATAAAAGCATAGAGACGATTCTACTT
WOPip4          CAAATAGAAGAACAAATTACACAAGCATTAGCAGATAGTAGCATAGAGACAATTATACTG
WOPip5          CAAATAGAAGAACAAATTACACAAGCATTAGCAGATAGTAGCATAGAGACAATTATACTG
WORiA           AAGATAAGTGAAGAGATAGAAGAAGCTTTAATAGATAAAGAAGTAGAAACAATAATTTTG
WORiB           AAAATTCATGAAGAGATAGAGAGAGCTTTAGAAGATAAAAGCATAGAGACGATTCTACTT
WORiC           AAGATACGTGAAGAGATAGAAGAAGCTTTAATAGATGAAGAAGTAGAAACAATAATTTTG
WOVitA1         AATATTCATGAAGAGATAGAGAGTGCTTTAGAAGATAAAAGCATAGAGACGATTCTACTT
WOVitA2         AATATTCATGAAGAGATAGAGAGCGCTTTAGAAGATAAAAGCATAGAGACGATTCTACTT
WOVitA4         CAAATAGAAGAACAAATTACACAAGCATTAGCAGATAGTAGCATAGAGACAATTATACTG
                 * **    * * * **       **  **  ****       **** ** **  *  *

WOcauB2         GAAATAGATAGCCCCGGAGGAGAGGTAAACGGTATATTTGACCTAGCTGACTTTATTTAT
WOcauB3         GATATAGATAGCCCAGGAGGAGAAGTAAATGGAGTGTTTGACCTTGCTGATTTTATTTAT
WOdamA1         GATATAGACAGTCCTGGAGGAGAGGTCAATGGAGTGTTTGACCTTGCCGATTTTATTTAC
WOHaA1          GAAATAGATAGCCCCGGAGGAGAGGTAAACGGTATATTTGACCTAGCTGACTTTATTTAT
WOMelA          GACATAGACAGCCCTGGAGGGGAAGTAAACGGTTTATTCGACCTTTCTGATTTTATTTAC
WOMelB          GACATAGACAGCCCCGGAGGAGAAGTAAACGGTTTATTCGACCTTTCCGACTTTATTTAC
WOPip1          GACATAGATAGTCCAGGTGGAGAAGTAAATGGAGTGTTTGATCTAGCTGATTTTATCTAC
WOPip2          GACATAGATAGTCCAGGTGGAGAAGTAAATGGAGTGTTTGATCTAGCTGATTTTATCTAC
WOPip3          GACATAGATAGTCCAGGTGGAGAAGTAAATGGAGTGTTTGATCTAGCTGATTTTATCTAC
WOPip4          GAAATAGACAGCCCAGGAGGAGAGGTAAACGGTATATTTGACCTGGCTGATTTTATTTAT
WOPip5          GAAATAGACAGCCCCGGAGGAGAGGTAAACGGTATATTTGACCTAGCTGACTTTATTTAT
WORiA           GACATAGACAGCCCCGGAGGAGAAGTAAACGGTTTATTCGACCTTTCCGACTTTATTTAC
WORiB           GATATAGATAGCCCAGGAGGAGAAGTAAATGGAGTGTTTGACCTTGCTGATTTTATTTAT
WORiC           GACATAGACAGCCCCGGAGGAGAAGTAAACAGTTTATTCGATCTTTCTGACTTTATTTAC
WOVitA1         GATATAGACAGTCCGGGAGGAGAAGTAAATGGAGTATTTGACTTAGCTGATTTTATCTAT
WOVitA2         GACATAGACAGTCCAGGAGGGGAAGTAAACGGTGTCTTTGACCTAGCTGATTTTATTTAT
WOVitA4         GAAATAGATAGCCCCGGAGGAGAGGTAAACGGTATATTTGACCTAGCTGACTTTATTTAT
                ** ***** ** ** ** ** ** ** **  *  * ** **  *  * ** ***** **

WOcauB2         GAATCAAGAGGAAAAAAGAGGATAATAGCGATAGCAAATGATGATGCATATTCTGCTGCG
WOcauB3         GAATCAAGAGCAAAAAAGAGAATTATTGCAATAGCAAATGACGATGCGTATTCTGCTGCG
WOdamA1         AGTGCAAGGGGAAAAAAGAGGATAATAGCGATAGCAAATGATGATGCATATTCTGCAGCG
WOHaA1          GAATCAAGAGCAAAAAAGAGGATAATAGCGATAGCAAATGATGATGCATACTCTGCTGCA
WOMelA          GAAGCAAGGGGATTAAAAAAGATCGTAGCAATAGCAAATGATGATGCGTATTCTGCGGCG
WOMelB          CAAGCAAGGAGAAAAAAGAGGATTGTGGCAATAGCAAATGATGATGCGTATTCTGCGGCG
WOPip1          GGTGTAAGAGGAAAAAAGAGGATAATAGCGATAGCAAATGATGATGCGTACTCTGCTGCA
WOPip2          GGTGTAAGAGGAAAAAAGAGGATAATAGCGATAGCAAATGATGATGCGTACTCTGCTGCA
WOPip3          GGTGTAAGAGGAAAAAAGAGGATAATAGCGATAGCAAATGATGATGCGTACTCTGCTGCA
WOPip4          GAATCAAGAGCAAAAAAGAGGATAATAGCGATAGCAAATGATGATGCATACTCTGCTGCA
WOPip5          GAATCAAGAGCAAAAAAGAGGATAATAGCGATAGCAAATGATGATGCATACTCTGCTGCA
WORiA           CAAGCAAGGAGAAAAAAGAGGATTGTGGCAATAGCAAATGATGATGCGTATTCTGCGGCG
WORiB           GAATCAAGAGCAAAAAAGAGAATTATTGCAATAGCAAATGACGATGCGTATTCTGCTGCG
WORiC           GAAGCAAGGGGATTAAAAAAGATCGTAGCAATAGCAAATGATGATGCGTATTCTGCGGCG
WOVitA1         AGTGCAAGAGGAAAAAAGAGGATAATAGCGATAGCAAATGATGATGCATACTCTGCTGCA
WOVitA2         GAATCAAGAACAAAAAAGAGAATTATTGCAATAGCAAATGATGATGCATATTCTGCTGCG
WOVitA4         GAATCAAGAGCAAAAAAGAGGATAATAGCGATAGCAAATGATGATGCATATTCTGCTGCG
                     ***   *  *** *  **  * ** *********** ***** ** ***** **

WOcauB2         TACGCTATAGCTTCTAGCGCTGAAAAGATTTTTCTCACCCGCACTTCAGGAGTTGGGAGT
WOcauB3         TATGCAATTGCTTCTAGTGCTGAAAAGATTTTTCTCACCCGCACTTCAGGAGTTGGGAGT
WOdamA1         TACGCTATAGCTTCTAGCGCTGAAAAGGTATTTGTGAGTAGAACTTCAGGAGTAGGAAGC
WOHaA1          TATGCTATAGCTTCAAGCGCTGAGAAGGTATTTGTGAGAAGAACTTCTGGTGTTGGCAGT
WOMelA          TATGCAATAGCGTCAAGTGCTGAAAAAGTATTGGTAACGAGGACTTCAGGAGTTGGAAGC
WOMelB          TATGCAATTGCATCAAGTGCTGAAAAAGTATTGGTAACTAGAACTTCAGGAGTTGGAAGC
WOPip1          TATGCTATAGCTTCAAGCGCTGAAAAGGTTTTTGTGTGTAGAACCTCTGGTGTTGGAAGT
WOPip2          TATGCTATAGCTTCAAGCGCTGAAAAGGTTTTTGTGTGTAGAACCTCTGGTGTTGGAAGT
WOPip3          TATGCTATAGCTTCAAGCGCTGAAAAGGTTTTTGTGTGTAGAACCTCTGGTGTTGGAAGT
WOPip4          TATGCTATAGCCTCTAGCGCTGAGAAGGTATTTGTGAGCAGAACTTCTGGTGTTGGCAGT
```

```
WOPip5          TATGCTATAGCCTCTAGCGCTGAGAAGGTATTTGTGAGCAGAACTTCTGGTGTTGGCAGT
WORiA           TATGCAATTGCATCAAGTGCTGAAAAAGTATTGGTAACTAGAACTTCAGGAGTTGGAAGC
WORiB           TATGCAATTGCTTCTAGTGCTGAAAAGATTTTTCTCACCCGCACTTCAGGAGTTGGGAGT
WORiC           TATGCAATAGCGTCAAGTGCTGAAAAAGTATTGGTAACGAGAACTTCAGGAGTTGGAAGC
WOVitA1         TATGCAATAGCTTCTAGCGCTGAGAAGGTATTTGTGAGCAGAACTTCTGGTGTTGGCAGT
WOVitA2         TACGCTATAGCCTCTAGCGCTGAAAAGGTATTTGTGAGTAGAACTTCAGGAGTAGGAAGT
WOVitA4         TACGCTATAGCCTCTAGCGCTGAAAAGGTATTTGTGAGCAGAACCTCAGGAGTAGGAAGC
                ** ** ** ** ** ** ***** **  * **  *     * ** ** ** ** ** **

WOcauB2         ATAGGAGTAATAGCAAGTCATATAGATCAAAGTGGATTTGATGAAAAATGTGGAATAAAA
WOcauB3         ATAGGGGTAATCGCAAGTCATATAGATCAAAGTGGGTTTGATGAAAAACAGGGGATAAAA
WOdamA1         ATAGGAGTAATAGCAAATCATATAGATCAAAGTGGGTTTGATGAAAAACAGGGAATAAAA
WOHaA1          ATTGGAGTAATAGCAAGTCATATAGATCAAAGTGGGTTTGATGAAAAACAGGGAATAAAA
WOMelA          ATAGGAGTAATAGCAAGTCATATAGATCAAAGTGGGTTTGATGAAAAACAGGGGATAAAA
WOMelB          ATAGGAGTAATAGCAAGTCATATAGATCAAAGTGGGTTTGATGAAAAACAGGGAATAAAA
WOPip1          ATAGGTGTTATTGCAAGTCACATTGACCAAAGCGGTTTTGATGAAAAGCAAGGAATAAAA
WOPip2          ATAGGTGTTATTGCAAGTCACATTGACCAAAGCGGTTTTGATGAAAAGCAAGGAATAAAA
WOPip3          ATAGGTGTTATTGCAAGTCACATTGACCAAAGCGGTTTTGATGAAAAGCAAGGAATAAAA
WOPip4          ATAGGAGTAATAGCAAGTCATATAGATCAAAGTGGGTTTGATGAAAAATGTGGAATAAAA
WOPip5          ATAGGAGTAATAGCAAGTCATATAGATCAAAGTGGGTTTGATGAAAAATGTGGAATAAAA
WORiA           ATAGGAGTAATAGCAAGTCATATAGATCAAAGTGGGTTTGATGAAAAACAGGGAATAAAA
WORiB           ATAGGGGTAATCGCAAGTCATATAGATCAAAGTGGGTTTGATGAAAAACAGGGGATAAAA
WORiC           ATAGGAGTAATAGCAAGTCACATTGATCAAAGTGGATTTGATGAAAAGCAAGGGATAAAA
WOVitA1         ATTGGAGTAATAGCAAGTCATATAGATCAAAGTAGGTTTGATGAAAAACAAGGTATTAAG
WOVitA2         ATAGGAGTAATAGCAAGTCACATAGATCAAAGTAGGTTTGATGAAAGGCAAGGTATTAAG
WOVitA4         ATAGGGGTAATTGCAAGTCATATAGATCAAAGTGGATTTAATGAAAAATGTGGAATAAAA
                ** ** ** ** **** *** ** ** *****  * *** ******     ** ** **

WOcauB2         TATACCACAGTGTTTGCAGGAAGTAGAAAAAAATGATTTAAATCCACATGAGCCAATAACT
WOcauB3         TATACTACAGTATTTGCTGGAAGTAGAAAAAAATGATTTAAATCCACATGAGCCAATAACT
WOdamA1         TATACAACAGTATTTGCAGGAAGTAGAAAAAAATGATTTAAATCCACATGAGCCAATAACT
WOHaA1          TACACCACAATCTTTGCTGGCAGTCGAAAGAATGATTTAAATCCGCATGAGCCAATAACG
WOMelA          TATACCACAGTATTTGCAGGAAAGAGAAAAAACGATTTAAATCCGCATGAGCCGATAACA
WOMelB          TATACGACAGTATTTGCAGGAAGTAGAAAAAAATGATTTAAATCCACATGAGCCAATAACT
WOPip1          TACACAACTATTTTTGCAGGCAAGAGAAAGAATGATTTAAATCCACATGAGCCAATGACG
WOPip2          TACACAACTATTTTTGCAGGCAAGAGAAAGAATGATTTAAATCCACATGAGCCAATGACG
WOPip3          TACACAACTATTTTTGCAGGCAAGAGAAAGAATGATTTAAATCCACATGAGCCAATGACG
WOPip4          TATACCACAGTATTTGCAGGAAGTAGAAAAAAATGATTTAAATCCACATGAGCCAATGACG
WOPip5          TATACCACAGTATTTGCAGGAAGTAGAAAAAAATGATTTAAATCCACATGAGCCAATGACG
WORiA           TATACGACAGTATTTGCAGGAAGTAGAAAAAAATGATTTAAATCCACATGAGCCAATAACT
WORiB           TATACTACAGTATTTGCTGGAAGTAGAAAAAAATGATTTAAATCCACATGAGCCAATAACT
WORiC           TATACAACTGTTTTTGCTGGAAGTCGAAAAAACGATTTAAACCCGCATGAGCCAATAACG
WOVitA1         TACACCACAATCTTTGCTGGTAGTCGAAAGAATGATTTAAACCCACATGAGCAAATGACG
WOVitA2         TATACCACAATCTTTGCTGGTAGTCGAAAGAATGATTTAAATCCGCATGAGCCAATGACG
WOVitA4         TATACCACAGTATTTGCAGGAAGTAGAAAAAAATGATTTAAATCCACATGAGCCAATGACG
                ** ** **  * ***** ** *   **** ** ******** ** *******  ** **

WOcauB2         TCTGAGAGTTTAGAAAATCTAAAAAGCGAAGTGAATCGTTTATATGAAATGCTGGTTGAG
WOcauB3         TCTGAGAGTTTAGAAAATCTAAAAAGCGAAGTGAATCGTTTATATGGAATGCTGGTTGAG
WOdamA1         TCTGAGAGTTTAGAAAACCTAAAAAGCGAAGTGAATCGTTTATATGGAATGCTGGTTGAG
WOHaA1          TCTGAAAGTTTAGAAAGCTTACAAAAAGAAGTAGGACGACTATATGAAATGTTTTTGCAG
WOMelA          TCAGAAAGTGTGGAAAGCTTACAAGATGAAGTAGACCGCCTATATGAGATGTTTGTCGAG
WOMelB          TCTGAGAGTTTAGAAAGCCTAAAAAGCGAAGTGAATCGTTTATATGGAATGCTGGTTGAG
WOPip1          TCTGAAAGTCTGGAAAGCTTACAAAAAGAAGTAGACCGACTATATGAAATGTTTGTGCAG
WOPip2          TCTGAAAGTCTGGAAAGCTTACAAAAAGAAGTAGACCGACTATATGAAATGTTTGTGCAG
WOPip3          TCTGAAAGTCTGGAAAGCTTACAAAAAGAAGTAGACCGACTATATGAAATGTTTGTGCAG
WOPip4          TCTGAAAGTCTGGAAAGCTTACAAAAAGAAGTAGACCGACTATATGAAATGTTTGTGCAG
WOPip5          TCTGAAAGTCTGGAAAGCTTACAAAAAGAAGTAGACCGACTATATGAAATGTTTGTGCAG
WORiA           TCTGAGAGTTTAGAAAGCCTAAAAAGCGAAGTGAATCGTTTATATGGAATGCTGGTTGAG
WORiB           TCTGAGAGTTTAGAAAATCTAAAAAGCGAAGTGAATCGTTTATATGGAATGCTGGTTGAG
WORiC           TCTGAAAGTCTGGAAAGCTTACAAGAGGAAGTAGGCCGCCTGTATGAGATGTTTGTCGAG
WOVitA1         TCTGAAAGTCTGGAAAGCTTACAAAAAGAAGTAGACCGACTATATGAAATGTTCGTCCAG
WOVitA2         TCTGAAAGTCTGGAAAGCTTACAAAAAGAAGTAGACCGACTATATGAAATGTTTTTGCAG
WOVitA4         TCTGAAAGTCTGGAAAGCTTACAAAAAGAAGTAGACCGACTATATGAAATGTTTGTGCAG
                ** ** *** * ****   ** **   *****    **  * ****  *** *  *  **

WOcauB2         CTAATAGCACGCAATAGAAACCTTTCTGTAGAGGCAATCAAATCAACAGAAGCAGGGCTA
WOcauB3         CTAATAGCGCGGAATAGAAGCCTCTCTGTAGAGGCAATAAAAAATACTGAAGCAGGGCTA
```

```
WOdamA1         CTAATAGCACGCAATAGAAACCTTTCTGTAGAGGCAATAAAATCAACAGAAGCAGGGCTA
WOHaA1          CTAATAGCAAGGAACAGAGGTCTTTCAATTGAAAAGATTCGATCAACAGAGGCAGGGCTA
WOMelA          CTTGTAGCAAGAAATAGGAATCTTTCCACGGAAAAAATCAAATCAACGGAAGCAGAGCTA
WOMelB          CTAATAGCACGCAATAGAAACCTTTCTGTAGAGGCAATAAAATCAACAGAAGCAGGGCTA
WOPip1          CTAATAGCAAGAAACAGAGGTCTTTCAATTGAAAAGATTCGATCAACAGAAGCAGGTCTA
WOPip2          CTAATAGCAAGAAACAGAGGTCTTTCAATTGAAAAGATTCGATCAACAGAAGCAGGTCTA
WOPip3          CTAATAGCAAGAAACAGAGGTCTTTCAATTGAAAAGATTCGATCAACAGAAGCAGGTCTA
WOPip4          CTAATAGCACGAAATCGAAACCTTTCTATAGAAGCAATCAAATCAACGGAAGCAGGGCTA
WOPip5          CTAATAGCACGAAATCGAAACCTTTCTATAGAAGCAATCAAATCAACGGAAGCAGGGCTA
WORiA           CTAATAGCACGCAATAGAAACCTTTCTGTAGAGGCAATAAAATCAACAGAAGCAGGGCTA
WORiB           CTAATAGCGCGGAATAGAAGCCTCTCTGTAGAGGCAATAAAAAATACTGAAGCAGGGCTA
WORiC           CTTGTAGCAAGAAATAGGAATCTTTCCACGGAAACAATCAAATCAACGGAAGCAGGGCTA
WOVitA1         CTAATAGCACGTAATCGCAATCTTTCTATACAAGCAATCAAATCAACGGAAGCAGGGCTA
WOVitA2         CTAATAGCAAGGAACAGAGGTCTTTCAATTGAAAAGATTCGATCAACAGAAGCAGGGCTA
WOVitA4         CTAATAGCGAGGAACAGAGGTCTTTCAATTGAAAAGATTCGATCAACGGAGGCAGGGCTA
                **  ****  * **  *    ** **     *    **   *   ** ** ****  ***

WOcauB2         TATTTTGGCGAGAAAGCAGTAGAAATAGGTCTTGCAGATGGAATTACAATTCTTTCAGAG
WOcauB3         TATTTTGGCGAGAAAGCAATAGAGATGGGTCTTGCAGATGGAATGACAATTCTTTC----
WOdamA1         TATTTTGGCGAGAAAGCAGTAGAAATAGGTCTTGCAGATGGAATTACAATTCTTTCAGAG
WOHaA1          TATTTTGGGGAGAAAGCAGTAGAAATAGGCCTTGCAGACGGAATGACAATTCTTTC----
WOMelA          TATTTTGGTGAGAAAGCAATAGAGATTGGACTTGCTGATGAAGTGATAACATATTCTGAA
WOMelB          TATTTTGGCGAGAAAGCAGTAGAAATAGGTCTTGCAGATGGAATTACAATTCTTTCAGAG
WOPip1          TATTTTGGCGAGAAAGCAGTAGAAATAGGTCTTGCAGATGGAGTTACAACATTTTTTGA-
WOPip2          TATTTTGGCGAGAAAGCAGTAGAAATAGGTCTTGCAGATGGAGTTACAACATTTTTTGA-
WOPip3          TATTTTGGCGAGAAAGCAGTAGAAATAGGTCTTGCAGATGGAGTTACAACATTTTTTGA-
WOPip4          TATTTTGGGGAGAAAGCAGTAGAAATAGGTCTTGCAGACGGAATGACAATTCTTTC----
WOPip5          TATTTTGGGGAGAAAGCAGTAGAAATAGGTCTTGCAGATGGAGTTACAACATTTTTTGA-
WORiA           TATTTTGGCGAGAAAGCAGTAGAAATAGGTCTTGCAGATGGAATTACAATTCTTTCAGAG
WORiB           TATTTTGGCGAGAAAGCAATAGAGATGGGTCTTGCAGATGGAATGACAATTCTTTC----
WORiC           TATTTTGGTGAGAAAGCAATAGAGATTGGACTTGCTGATGAAGTAATAACATATTCTGAA
WOVitA1         TATTTTGGCGAGAAAGCAGTAGAAATAGGTCTTGCAGATGGAGTTACAACATTTTTTGA-
WOVitA2         TATTTTGGCGAGAAAGCAGTAGAAATAGGTGTTGCAGATGGAGTTACAACATTTTTTGA-
WOVitA4         TATTTTGGCGAGAGAGCAATAGAAATAGGTCTTGCAGATGGAATTACAATTCTTTC----
                ******** **** **** **** ** **  **** ** * * * * **    **

WOcauB2         TTTAAATATATTAATAAAAACAGGAGTA----TTACTATGAACGAACAAACTATA--ACT
WOcauB3         -----ATCTATTAATAAAAACAGGAGTA----TTACTATGAATGAACAAACTACA--AAT
WOdamA1         TTTAAATCTATTAATAAAAACAGGAGTA----TTACTATGAGCGAACAAACTACA--ACT
WOHaA1          -----ATCTATTAATAAAAACAGGAGTA----TTACTATGAATGAACAAACTACA--AAT
WOMelA          TTTATAGATAGGAGAAACAATATGAGTAAAGCTGAATTTAACTATAAGGGCCACACCATC
WOMelB          TTTAAATCTATTAATAAAAAAGGGGATA----TTACTATGAATGAAAAAACTACA--AAT
WOPip1          -----ATTTATTAACAAAGGAGAAAATA----CTA---TGAATAAACAAACTACA--ACT
WOPip2          -----ATTTATTAACAAAGGAGAAAATA----CTA---TGAATAAACAAACTACA--ACT
WOPip3          -----ATTTATTAACAAAGGAGAAAATA----CTA---TGAATAAACAAACTACA--ACT
WOPip4          -----ATCTATTAATAAAAATAGGAGTA----TTACTATGAATGAACAAACTACA--AAT
WOPip5          -----ATTTATCAATAATCATAGGAGTG----TTAGTATGA---------CAACT--AAT
WORiA           TTTAAATCTATTAATAAAAAAGGGGATA----TTACTATGAATGAAAAAACTACA--AAT
WORiB           -----ATCTATTAATAAAAACAGGAGTA----TTACTATGAATGAACAAACTACA--AAT
WORiC           TTTATAGATAGGAGAAACAATATGAGTAAAGCTGAATTTAACTATAAGGGCCACACCATC
WOVitA1         -----ATTTATCAACAATCATAAAAGTA----G------GAGTGTTAGTATGACA--ACT
WOVitA2         -----ATTTATCAATAATCATAAAAGTA----G------GAGTGTTAGTATGACA--ACT
WOVitA4         -----ATCTATTAATCAAAACAGGAGTA----TTATTATGAATGAACAAACTACA--AAT
                     *  **  *   *         *             *           *    *

WOcauB2         GACCTAAAAGAAGAAACTAATAA---------------TTTAACTAAGTATCGTACTGAA
WOcauB3         GACCTA------GAAACTGATAA---------------TTTAACCAAGTATCGTACTGAA
WOdamA1         GACCTA------GAAACTGATAA---------------TTTAACTAAGTATCGTACTGAA
WOHaA1          GACCTA------GAAACTGATAA---------------TTTAACCAAGTATCGTACTGAA
WOMelA          GATGCTGATGAGTTAATGGAAGAATCAAAACGAATAGGTTATGAGAGGTGCCGCAAGGAA
WOMelB          GACCTA------GAAACTGACAA---------------TTTAACTAAGTATCGTACTGAA
WOPip1          GACCTA------GAAATTGATGACCTAGAAACTGATAATTTAACTAAGTATCGTACTGAA
WOPip2          GACCTA------GAAATTGATGACCTAGAAACTGATAATTTAACTAAGTATCGTACTGAA
WOPip3          GACCTA------GAAATTGATGACCTAGAAACTGATAATTTAACTAAGTATCGTACTGAA
WOPip4          GACCTA------GAAACTGATAA---------------TTTAACCAAGTATCGTACTGAA
WOPip5          GAGTT---------AACTGAGGA---------------G------AACTGTCGTAGAGAA
WORiA           GACCTA------GAAACTGACAA---------------TTTAACTAAGTATCGTACTGAA
WORiB           GACCTA------GAAACTGATAA---------------TTTAACCAAGTATCGTACTGAA
```

```
WORiC           GATGCTGATGAGTTAATGGAAGAATCAAAACGAATAGGTTATGAGAGGTGCCGCAAGGAA
WOVitA1         GATGAG------TTAATTGAGGA---------------------GAACTATCGTAGAGAA
WOVitA2         AATGAG------TTAACTGAGGAA------------GGCTACGAGAACTGTCGTAGAGAA
WOVitA4         GACCTA------AAAACTAATAA---------------TTTAACTAAGTATCGTACTGAA
                 *            **   *  *                      *  *  ** *  ***

WOcauB2         GTTCTTGAATTAATACGTTTATGTAATATATCAAAGATGCCAGAAAAGATAGGAGAATTT
WOcauB3         GTTCTTGAATTAATACGATTATGTAATATATCGAAGATGCCAGAAAAGATAGGAGAATTT
WOdamA1         GTTCTTGAATTAATACGATTATGTAATATATCGAAGATGCCAGAAAAGATAGGAGAATTT
WOHaA1          GTTCTTGAATTAATACGTTTATGTAATTTATCGAAGATGCCAGAAAAGATAGGAGAATTT
WOMelA          GTATGAGAGGTAATAAGATTGTGTAATTTGTCAAAGATGCCAGAGAAGATAGGAGAATTT
WOMelB          GTTCTTGAATTAATACGTTTATGTAACTTATCACGAATGCCAGAAAAGATAGGAGAATTT
WOPip1          ATTGTTGAATTAATACGTTTATGTAACTTATCACGAATGCCAGAGAAAATAGGAGAATTT
WOPip2          ATTGTTGAATTAATACGTTTATGTAACTTATCACGAATGCCAGAGAAAATAGGAGAATTT
WOPip3          ATTGTTGAATTAATACGTTTATGTAACTTATCACGAATGCCAGAGAAAATAGGAGAATTT
WOPip4          GTTCTTGAATTAATACGTTTATGTAATTTATCGAAGATGCCAGAAAAGATAGGAGAATTT
WOPip5          ATTTTAGAGATAATAAGATTATGTAATATATCAAAGATGCCAGAAAAGATAGGAGAATTT
WORiA           GTTCTTGAATTAATACGTTTATGTAACTTATCACGAATGCCAGAAAAGATAGGAGAATTT
WORiB           GTTCTTGAATTAATACGATTATGTAATATATCGAAGATGCCAGAAAAGATAGGAGAATTT
WORiC           GTATTAGAGGTAATACGATTATGTAATTTATCAAAGATGCCAGAAAAGATAGGAGAATTT
WOVitA1         ATTTTAGAGATAATAAGATTATGTAATGTATCAAAGATGCCAGAGAAGATAGGAGAATTT
WOVitA2         ATTTTAGAGATAATAAGATTATGTAATATATCAAAGATGCCAGAAAAGATAGGAGAATTT
WOVitA4         GTTGTTGAATTAATACGTTTGTGTAACTTATCACGAATGCCAGATAAAATAGGAGAATTT
                 *    **  ***** * ** *****  * **    ******** ** ************

WOcauB2         ATTGAGCAGGGTGTAAGTGTTGAGCAAGCAAGGGAGGTTTTAATGGAATTACTTGCAGAG
WOcauB3         ATTGAGCAAAGCGTAAGTGTTGAGCAAGCAAGGGAGGTTTTAATGGAATTACTTGCAGAA
WOdamA1         ATTGAGCAGGGCGTAAGTGTTGAGCAAGCAAGGTAGGTTTTAATGGAATTACTTGCAGAG
WOHaA1          ATTGAGCAAAGTGTAAGTGTTGAGCAAGCTAGGGAAGTTTTAATGGAATTACTTGCAGAG
WOMelA          ATTGAGCAGGATGTAAACGCAAAACAAGCCCAAGAGATATTAATGTCGATACTGGCGGAG
WOMelB          ATTGAGCAAAGCGTAAGTGTTGAGCAAGCCAGGGAAGTTTTAATGGAGTTACTTGCAGAG
WOPip1          ATTGAGCAGGGTGTAAGTATTGAGCAAGCGAGGGAAGTTTTAATGGAATTACTTGCAGAG
WOPip2          ATTGAGCAGGGTGTAAGTATTGAGCAAGCGAGGGAAGTTTTAATGGAATTACTTGCAGAG
WOPip3          ATTGAGCAGGGTGTAAGTATTGAGCAAGCGAGGGAAGTTTTAATGGAATTACTTGCAGAG
WOPip4          ATTGAGCAAAGTGTAAGTGTTGAGCAAGCAAGGGAGGTTTTAATGGAATTACTTGCAGAG
WOPip5          ATAGAGCAAGGCTCAAGTGTTGAGCAAGCCAGGGAAGTTTTAATGGAGTTACTTGCAGAG
WORiA           ATTGAGCAAAGCGTAAGTGTTGAGCAAGCCAGGGAAGTTTTAATGGAGTTACTTGCAGAG
WORiB           ATTGAGCAAAGCGTAAGTGTTGAGCAAGCAAGGGAGGTTTTAATGGAATTACTTGCAGAA
WORiC           ATTGAGCAGGATGTAAATGCAAAACAAGCCCAAGAAATATTAATGTCGATACTAGCAGAG
WOVitA1         ATAGAGCAAGGAGTAAGTATTGAGAAAGCACGGGAAGTTTTAATGGAGTTACTTGCAGAG
WOVitA2         ATAGAGCAAAGCGTGAGTGTTGAGCAAGCCAGGGAAGTTTTAATGGAATTACTTGCAGAG
WOVitA4         ATTGAGCAGGGTGTAAGTGTTGAGCAAGCAAGGGAGGTTTTAATGGAATTACTTGCAGAA
                ** *****       *      *  ****     *  * ******    **** ** **

WOcauB2         CAAACGAAAAAGACAGAGATACTGAGTGCAATACCACAGAATTCAGGAGAAGAGTTGATG
WOcauB3         AGAACGAAGAAGACAGAGATACTGAGTGCAGTACCACAGAATTCAGGAGAAGAATTGATG
WOdamA1         CGAACGAAAAAGACAGAGATACTGAGTGCAATACCACAGAATTCAGGAGAAGAGTTGATG
WOHaA1          AGAACAAAGAAGACAGAGATACTGAGTGCAATACCACAGAATTCAGGAGAAGAATTGATG
WOMelA          AGAACGAAGAAGACAGAGATACTGAGCACAATACCACAAAGTTCATCAGAAGATTTAATG
WOMelB          CGAACGAAGAAGACAGAAATACTAAGTGCAATACCACAGAATTCAGGAGAAGAGTTGATG
WOPip1          AGAACGAAAAAGACAGAGATACTGAGTGCAATACCACAGAATTCGCAGGAAGATTTGATG
WOPip2          AGAACGAAAAAGACAGAGATACTGAGTGCAATACCACAGAATTCGCAGGAAGATTTGATG
WOPip3          AGAACGAAAAAGACAGAGATACTGAGTGCAATACCACAGAATTCGCAGGAAGATTTGATG
WOPip4          CGAACGAAAAAGACAGAGATACTGAGTGCAATACCACAGAATTCAGGAGAAGAGTTGATG
WOPip5          CGAACGAAAAAAACAGAGATACTGAGTGCAATACCACAGAATGCAGGAGAAGAATTGATG
WORiA           CGAACGAAGAAGACAGAAATACTAAGTGCAATACCACAGAATTCAGGAGAAGAGTTGATG
WORiB           AGAACGAAGAAGACAGAGATACTGAGTGCAGTACCACAGAATTCAGGAGAAGAATTGATG
WORiC           AGAACAAAGAAGACAGAGATTTTGAGTACAATACCGCAAAGTACACCGGAAGACTTGATG
WOVitA1         CGAACGAAAAAGACAGAGATACTGAGTGCAATACCACAGAATTCAGGAGAAGAGTTGATG
WOVitA2         CGAACGAAAAAGACAGAGATACTGAGTGCAATACCACAGAATTCAGGAGAAGATTTAATG
WOVitA4         AGAACAAAGAAGACAGAGATACTGAGTGCAATACCGCAGAATTCAGGAGAAGAGTTGATG
                  *** ** ** ***** **  * **  ** **** ** * * *    ***** ** ***

WOcauB2         ATGCAGGTAGC
WOcauB3         ATGCAGGTAGC
WOdamA1         ATGCAGGTAGC
WOHaA1          ATGCAGGTAGC
WOMelA          ATGCAGGTAGC
```

```
WOMelB          ATGCAGGTAGC
WOPip1          ACACAGGTAGC
WOPip2          ACACAGGTAGC
WOPip3          ACACAGGTAGC
WOPip4          ATGCAGGTAGC
WOPip5          ATGCAGGTAGC
WORiA           ATGCAGGTAGC
WORiB           ATGCAGGTAGC
WORiC           ATGCAGGTAGC
WOVitA1         ATGCAGGTAGC
WOVitA2         ATACAGGTAGC
WOVitA4         ATGGAGGTAGC
                *   *******
```

1
